# On the Broader Significance of Maternal Sensitivity: Mothers’ Early and Later Sensitive Parenting Matter to Children's Language, Executive Function, Academics, and Self‐Reliance

**DOI:** 10.1111/desc.13594

**Published:** 2024-12-16

**Authors:** Joan E. Foley, Thomas M. Olino, Marsha Weinraub

**Affiliations:** ^1^ Psychology and Neuroscience Temple University Philadelphia Pennsylvania USA

**Keywords:** academics, executive function, language, middle childhood, parenting, self‐reliance

## Abstract

Researchers have demonstrated the important contribution of mothers’ sensitive parenting to children's developing cognition over the first 5 years of life, yet studies examining sensitivity beyond the early years, controlling for earlier effects, are limited. In this exploratory study, we examined the developmental pathways through which mothers’ early and later sensitive parenting transacted with children's language, executive function, academics, and self‐reliance to predict child outcomes from infancy to adolescence. To a national longitudinal dataset (*n *= 1364; 52% male; 80% white), we applied random intercept cross‐lagged panel modeling to examine between‐person and within‐person associations for maternal sensitivity and child outcomes. Our findings show that over the first 15 years of life relations between maternal sensitivity and these child outcomes are best characterized by stable, trait‐like associations that persist over time with limited state‐like time‐varying associations. Importantly, we found that maternal sensitivity at both early and later developmental stages is associated with these between‐person differences. Given the nature of these associations over four developmental stages, we extend prior research by demonstrating that mothers’ sensitivity is *enduring* because of its *consistency* both early and later in development.

## 1 Introduction

Researchers differ on the role of early versus later experience in predicting children's cognitive outcomes. Even though it is widely recognized that maternal sensitivity over the first 5 years has concurrent and predictive relations with children's early cognitive outcomes (e.g., Bernier, McMahon, and Perrier 2017; Bornstein et al. 2020), investigations into the role of later maternal sensitivity, controlling for earlier sensitivity, are limited. This leaves open the important question about the role of later maternal sensitivity in children's cognitive development after considering early effects (Vandell and Gülseven 2023).

**Summary**

- Over the first 15 years of life, relations between maternal sensitivity and children's cognition and achievement reflect stable, trait‐like associations with limited state‐like time‐varying associations.
- Maternal sensitivity at early and later developmental stages is associated with these stable, trait‐like associations.
- This study's findings extend prior research by demonstrating that mothers’ sensitivity is *enduring* because of its *consistency* both early and later in development.
- State‐like, time‐varying associations in middle childhood show math problem‐solving as the leading indicator of reading comprehension, and that math and classroom self‐reliance are reciprocally related.

Some theorists argue that the effects of early experience endure throughout development (e.g., Fraley, Roisman, and Haltigan 2013) and are more important than later influences. Other theorists claim that early experience sets the course, but subsequent experiences build on and override earlier experiences to influence development (e.g., Lewis 1998). Alternatively, some theorists claim that patterns of adaptation established early in development set in motion a chain of experiences with each impacting the next subsequent phase (Bowlby 1982; Sameroff 2009; Sroufe, Coffino, and Carlson 2010). Rather than asking, does early experience makes a difference and is that early effect enduring, many developmental psychologists have come to believe that the more valuable approach to understanding the role of experience is to examine the processes through which both early and later experiences and the individual's constitution all combine and interact to influence the life course (Andersen, Steinberg, and Belsky 2021; Marshall and Kenney 2009). Sroufe, Coffino, and Carlson (2010) advocated for a comprehensive model of development capable of mapping transactional effects that interact across time to reveal patterns of associations during development. Fraley, Roisman, and Haltigan (2013) argued for longitudinal multi‐wave studies covering successive periods of development to understand the role of early experience and its continuing significance over time. Vandell and Gülseven (2023) further argued for extending the work of Fraley et al. using the same dataset to identify the developmental pathways by which functioning in early childhood and middle childhood are carried forward.

Much of what we know about the effects of maternal sensitivity on children's cognitive development stems from research covering the first 5 years of life (see review by Deans 2020) while examining one or two domains of cognition—for example, language, cognitive or behavioral control, or academic performance (e.g., Bornstein et al. 2020; Madigan et al. 2019; Wade et al. 2018). However, researchers do not always agree on the sequence of development when one or two cognitive domains are studied together with maternal sensitivity. For example, when researchers examined the effects of early maternal sensitivity with language and executive function (EF) separately, they found direct effects of maternal sensitivity on each domain (e.g., Bernier, Carlson, and Whipple 2010; Bindman, Pomerantz, and Roisman 2015; Conway 2020). However, when researchers examined the effects of early maternal sensitivity, language, and EF together, some researchers reported direct effects of sensitivity on EF (Frick, Forslund, and Brocki 2019) while others reported mediation through language (Matte‐Gagné and Bernier 2011). Lastly, one study found no evidence of longitudinal associations between maternal sensitivity in infancy and preschool EF (Jónsdóttir et al. 2024). Such equivocal findings leave open questions about how sensitive parenting predicts the sequence of cognitive development.

The effects of later sensitivity on cognition and academics, after controlling for early effects, have received less attention in school‐age children and adolescents (Bradley and Pennar 2011). Fraley, Roisman, and Haltigan (2013) and Raby et al. (2015), for example, concluded that early maternal sensitivity has enduring effects on academic outcomes across childhood and adolescence. Yet the transactional model of development (Sameroff 2009) argues that proximal interactions between a mother and her child foster development suggesting a continuing role for mothers’ sensitive parenting supporting children's achievement later in development. Here, we build on prior research to understand how early and later sensitivity and children's progressive changes in cognition predict academic achievement and self‐reliance in middle childhood and adolescence.

### 1.1 Developmental Pathways and Mother–Child Transactions: Theoretical Foundation

#### 1.1.1 The Mother–Child Dyad

This study is guided by the transactional model of development (Sameroff 2009) in which proximal social *processes* and *experiences* involving the mother–child dyad reflect ongoing transactions in which each member of the dyad influences the other contributing to change over time. This phenomenon is clearly present in Vygotsky's (1978) theory of scaffolded learning in which a more knowledgeable person (parent) provides structured support to the child in concept or task mastery. As the parent observes the child's increasing proficiency, support is adjusted accordingly offering the child more autonomy—an important attribute of sensitive parenting. Reciprocal influences between mother and child, such as those described here, and the processes through which change occurs suggest that the child shapes and is shaped by their environment. We draw from the tenets of the transactional model to understand the relative contribution of early and later maternal sensitivity and the temporal processes that predict language development, EF, academic achievement, and self‐reliance.

#### 1.1.2 Early Experience and Brain Development

Growing evidence suggests that associations between maternal sensitivity and child outcomes stem, in part, from the *proximal* effects of mother–child interactions on infants’ neural structures (Bernier et al. 2019; Bick and Nelson 2016). Individual differences in sensitive parenting have been linked to hippocampal (Blankenship et al. 2019) and amygdala (Lyons‐Ruth et al. 2016) volumes in the first 6 months of life as well as longer‐term (Ilyka, Johnson, and Lloyd‐Fox 2021). The hippocampus is known for its role in learning and memory (Rubin et al. 2014), and the amygdala regulates emotion and memory to support social information processing (Phelps and LeDoux 2005). Both brain regions grow rapidly in infancy (e.g., Gilmore, Knickmeyer, and Gao 2018) with early environmental factors influencing growth (Tottenham 2013). These findings confirm that, at least to some extent, early experience and sensitive parenting support infant brain maturation in areas important to learning and social functioning (Belsky and de Haan 2011). Consequently, this period of rapid growth has been viewed as a window of opportunity (Andersen 2003). Nevertheless, because the brain is malleable (Buttelmann and Karbach 2017), the effects of later sensitivity warrant investigation.

#### 1.1.3 Mother–Child Interactions and Language Development

Sociocultural theories of language development, following the tenets of transactional models, argue that social understanding and interaction with expert partners facilitate the emergence of child language (Vygotsky 1978). Parental responsiveness promotes social interaction that may serve as a plasticity trigger for the “gate” that initiates early language learning (Kuhl 2011). These social cues between parent and child contribute to language development (Hirsh‐Pasek and Burchinal 2006; Madigan et al. 2019; Vallotton et al. 2017) and include the child's ability to follow adult gaze direction, joint attention between parent and child, and the parent's contingent responsiveness to child behavior (Masek et al. 2021; Tamis‐LeMonda, Kuchirko, and Song 2014). Children who reach the vocabulary spurt and simple sentence construction milestones sooner than other children have more verbally responsive mothers (Bornstein et al. 2020).

Individual differences in children's language skills are evident in the first few years of life, and they demonstrate considerable stability over time (Bornstein et al. 2018). Consequently, it is important to understand how maternal sensitivity not only is associated with language growth in the first 2 years of life but also how these two variables—maternal sensitivity and language ability—operate together to influence subsequent development.

#### 1.1.4 Mother–Child Interactions, Language, and EF

Because social interactions act as experience‐expectant input to guide infants’ attention and sense of control over the environment (Fay‐Stammbach, Hawes, and Meredith 2014; Thompson and Steinbeis 2020), early social interaction with expert others fosters joint attention in support of early language learning (Kuhl 2011). As children's language develops and they begin to use private speech to aid in attention control, extrinsic regulation becomes intrinsic (Diaz and Berk 2016; Vygotsky 1987). According to Zelazo et al. (2003), early language complexity acts as a precursor to later EF. Thus, the sequence of higher‐order EF development may begin in infancy with sensitive parenting guiding attention control and early language development.

Evidence exists for parenting, attention, and language learning to support EF development, yet the timing of assessment and selection of measures may be factors in equivocal findings concerning the sequence of development. Frick, Forslund, and Brocki (2019) reported that maternal sensitivity in infancy directly predicted toddlers’ receptive and expressive language and preschool working memory, set‐shifting, and delay of gratification. Other researchers have reported that early language mediates relations between infant/toddler sensitive parenting and preschool EF (e.g., Matte‐Gagné and Bernier 2011) but more research is needed here.

#### 1.1.5 Academic Achievement and Self‐Reliance: A Role for Later Sensitivity?

According to Bernier, Beauchamp, and Cimon‐Paquet (2020, 136), early maternal behaviors likely “constitute the beginning of the developmental chain” that predict EF in support of school success. Mediation models of sensitivity and academics have demonstrated that child cognitive factors mediate relations between the two domains (e.g., Bernier, Beauchamp, and Cimon‐Paquet 2020; Bindman, Pomerantz, and Roisman 2015; Cimon‐Paquet et al. 2020; Fenesy and Lee 2018). If the early effects of mothers’ sensitive parenting on academic achievement are mediated by early cognitive factors, is there a role for mothers’ later sensitive parenting to influence these same domains?

The influence of proximal processes in fostering development, an element of the transactional model, would suggest that mother–child interactions during middle childhood and adolescence have effects on children's achievement during this later developmental stage. With the foundations of early cognitive development in place, later sensitive parenting may inspire children's relational and motivational frameworks that, in turn, influence school success (e.g., Bridgewater and Yates 2020; Rowe, Ramani, and Pomerantz 2016). Research has shown that parents who are more sensitive have children who experience less student‐teacher relational conflict over time and that has been found to predict higher academic performance (Bridgewater and Yates 2020; Magro et al. 2022). Parents who showed sensitive behaviors during homework assistance had children who demonstrated higher academic performance compared to the children of parents who were intrusive and non‐supportive during homework. Self‐determination theory (Ryan and Deci 2000) and empirical evidence (e.g., Silinskas and Kikas 2019) suggest that sensitive parenting in middle childhood has positive effects on children's intrinsic motivation and self‐reliance, and these self‐reliant children strive for higher academic achievement when positively supported in their work.

Self‐reliance, a form of cognitive autonomy, includes behaviors associated with personal initiative, self‐regulation, autonomy, persistence, and engagement (Ryan and Deci 2000). Children's autonomous behaviors and parents’ supportive behaviors are thought to coexist and are bidirectionally related. As the child engages in more autonomous and self‐reliant behavior, the parent responds by enabling more independent decision‐making and control over the self. In turn, the child gains increased control over choices and decisions in the classroom with implications for academic outcomes (Steinberg 1990).

Although evidence suggests that self‐reliance mediates the effects of supportive parenting on academics (e.g., NICHD Early Child Care Research Network, NICHD‐ECCRN 2008), studies have yet to examine mothers’ sensitive parenting together with self‐reliance and early cognition to understand the patterns of association across extended developmental periods. This approach could lead to alternative conclusions about the role of self‐reliance in children's outcomes. It is also unknown whether children's autonomous behaviors and sensitive parenting are reciprocally related because studies have been limited in their ability to capture these effects.

### 1.2 The Present Study

Using a transactional framework of development (Sameroff 2009; Masten and Cicchetti 2010), this study extends prior research by examining the developmental pathways by which both early and later maternal sensitivity and children's cognition and academic achievement transact to predict child outcomes from infancy to adolescence. By exploring the temporal sequence of development for mothers’ sensitivity and children's language, EF, reading and math achievement, and self‐reliance, we hope not only to assess the contributions of early and later sensitivity but also to determine whether these effects demonstrate stable, trait‐like associations, state‐like, time‐varying associations, or a combination of both associations. Additionally, we examine whether maternal sensitivity in the infancy/toddler years has enduring indirect effects on reading, math, and children's self‐reliance through age 15 via early cognition. We also examine the effects of later maternal sensitivity—during early childhood and middle childhood—on these same outcomes after controlling for maternal sensitivity in the preceding period. To address these research interests, we applied random intercept cross‐lagged panel modeling—an approach that disaggregates between‐ and within‐person effects—to data from the NICHD Study of Early Child Care and Youth Development, a longitudinal study following mother–child dyads from infancy through adolescence.

**FIGURE 1:**
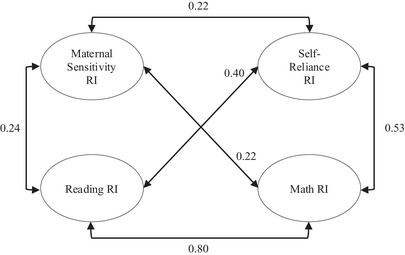
Correlations between random intercepts for maternal sensitivity, reading, math, and self‐reliance. Correlations significant at *p* < 0.05.

**FIGURE 2:**
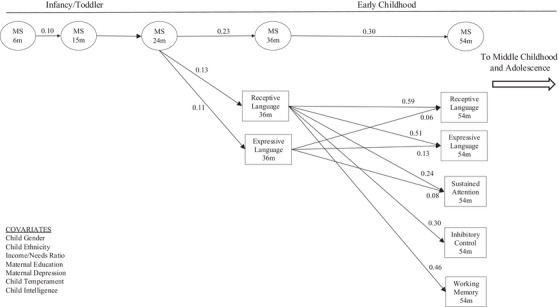
Early maternal sensitivity: Developmental pathways to language and executive function across the first 5 years of life. Standardized beta coefficients shown. *p* < 0.05.

Based on prior research, we hypothesized that children's early language and EF would mediate the effects of infant/toddler maternal sensitivity on academic achievement and self‐reliance. With this early cognitive foundation in place, we hypothesized that later maternal sensitivity, after controlling for earlier effects, would contribute to children's academic achievement and self‐reliance by supporting children's self‐motivation and autonomous behaviors. We also hypothesized that mothers’ sensitivity and children's cognition and achievement would be reciprocally related over time. Our hypotheses are drawn from existing research that has not distinguished stable between‐person differences from within‐person variations over time. Thus, there is reason to consider that our findings may not support these hypotheses.

Previous research involving relations between mothers’ sensitivity and child outcomes frequently used methods combining between‐person effects and within‐person effects. These methods have been criticized for failing to distinguish stable between‐person differences from within‐person variations over time. Conflation of within‐person and between‐person variability is likely to result in a distorted view of the dynamics that characterize these processes (Berry and Willoughby 2016; Hamaker, Kuiper, and Grasman 2015; Lucas 2023). In this study, we use the random intercept cross‐lagged panel model (RI‐CLPM), a method that is capable of disaggregating between‐ and within‐person associations. According to Hamaker (2023, 2), “a common feature of this approach is that it accounts for stable factors—such as traits, trends, random intercepts, and unit effects—that represent lasting characteristics of individuals, thereby allowing for the investigation of within‐in person cross‐lagged effects that are not contaminated by stable between‐person differences”. Because we are not aware of research investigating maternal sensitivity and children's cognition and achievement using RI‐CLPM or similar methods to examine between‐ versus within‐person associations, we consider this study exploratory.

## 2 Method

### 2.1 Sample

With a large and diverse sample of families, the National Institute of Child Health and Development Study of Early Child Care and Youth Development (NICHD‐SECCYD), a publicly available longitudinal data set, was ideal for testing our model (Vandell and Gülseven 2023). Data from birth through age 15 formed the basis for analyses.

Researchers recruited 1364 families in 1991 through hospital visits with mothers giving birth (*n* = 8986) during selected 24‐h intervals in 10 locations in the United States. Study recruitment and protocols are described in https://www.icpsr.umich.edu/web/ICPSR/studies/21940. Children with neurodevelopmental disorders or preterm births were not included in the sample, and each family contributed only one child to the study. The resulting sample included 24% of ethnic minority children, 11% of mothers who had not completed high school, and 14% of mothers categorized as single. Mothers on average completed 14.4 years of education (*SD* = 2.49 years), and 51.7% of children were boys. At each assessment, written informed consent was obtained from parents; age‐appropriate consents were obtained from the children.

Attrition is a factor in any longitudinal study, and some families did not participate in every wave of data. At age 15, 958 (70%) of families remained and many children had some missing data. Missing data for study variables ranged from 6% to 35% with the highest amount of missing data at age 15. In a previous study by Vandell et al. (2010), comparisons of the age 15 sample and the other 406 youth in the birth cohort sample showed that nonparticipant children were more likely to be male, had lower math skills at 54 months‐of‐age, and their mothers had almost 1 year less of education and provided somewhat lower quality parenting than other mothers. To account for missing data, full information maximum likelihood structural equation analyses were conducted to include all observations with at least partial data on endogenous variables; cases missing data on exogenous variables were omitted from the analyses (Enders and Bandalos 2001). We also conducted an additional analysis that implemented full information maximum likelihood that includes modeling exogenous time‐invariant covariates as endogenous and found that modeling results for the paths central to our inferences were substantively identical.

### 2.2 Procedures and Measures

Home, laboratory, and childcare or school assessments occurred when children were 1, 6, 15, 24, 36, and 54 months old, in kindergarten, and when children were in grades 1, 2, 3, 4, 5, and 6, and age 15. This study used data at 1, 6, 15, 24, 36, and 54 months, grades 1, 3, and 5, and age 15. Maternal sensitivity, cognition, and academic measures were collected as described below; demographic and interview data were also collected. Children's self‐reliant behavior in grades 1, 3, and 5 was assessed using the classroom observation system (COS) developed by the NICHD‐SECCYD to examine the study child's experiences and behavior in the classroom. Detailed information on data collection at each assessment can be found at https://www.icpsr.umich.edu/web/ICPSR/search/studies?q=NICHD+Study+of+Early+Child+care+and+Youth+Development.

#### 2.2.1 Maternal Sensitivity

##### 2.2.1.1 Procedures

Trained observers videotaped mother–child interactions during 15–20 min semi‐structured sessions when the child was 6, 15, 24, 36, and 54 months of age, in grades 1, 3, and 5 (approximately 6, 8, and 10 years of age), and at age 15. Detailed descriptions of mother–child interaction tasks are provided in Supplement (S1).

##### 2.2.1.2 Measures

From videotapes of the sessions, trained coders rated mothers’ sensitivity on scales refined at each time period for age and task appropriateness (see M. T. Owen et al., 2000, Unpublished manuscript). Coders rated mothers’ behaviors based on a 7‐point scale (1 = *very low observed behavior* to 7 = *very high observed behavior*). Because ratings at 6, 15, and 24 months were originally based on 4‐point scales, we transformed these scales to 7‐point scales to provide scoring consistent with later ages (see Burchinal, Vandell, and Belsky 2014)

At 6, 15, and 24 months, raters assessed mothers’ responses to nondistress, positive regard, and intrusiveness (reversed). Examples of nondistress included acknowledgments of child affect, contingent vocalizations, and appropriate attention focusing. Examples of positive regard included speaking in a warm tone of voice, praising the child, and expressing physical affection. Examples of intrusiveness included not allowing the child to influence play or handle reached for toys and insisting that the child acts in a particular way. Inter‐coder reliabilities ranged from 0.62 to 0.87.

At 54 months and in grades 1, 3, and 5, raters assessed mothers’ supportiveness, respect for autonomy, and hostility (reversed). Examples of supportiveness included acknowledging child accomplishments, positive encouragement, and providing an affectively secure base. Examples of respect for child autonomy included acknowledging the child's perspectives and desires, explicitly negotiating rules, and acknowledging the child's intention. Examples of mothers’ hostility included clearly and overtly rejecting the child and blaming the child for mistakes. Intercoder reliabilities ranged from 0.62 to 0.87. At age 15, raters assessed mothers’ warmth and responsiveness, respect for autonomy, and hostility (reversed). Intercoder reliabilities ranged from 0.68 to 0.87.

#### 2.2.2 Child Language at 36 and 54 Months

At 36 months, trained testers administered The Reynell Developmental Language Scales (Reynell and Gruber 1990) to assess verbal comprehension and expressive language. At 54 months, trained testers administered the Preschool Language Scale‐3 (PLS‐3; Zimmerman, Steiner, and Pond 1992) to assess auditory comprehension and expressive communication. Standardized scores at 36 and 54 months were used in our analyses.

#### 2.2.3 EF in Early Childhood

##### 2.2.3.1 Continuous Performance Task

The Continuous Performance Task was selected to measure sustained attention and inhibitory control (Mirsky et al. 1991). The task was computer‐generated revealing dot matrix pictures of familiar objects (e.g., butterfly, fish, flower) on a 2‐in. square screen in front of the child over a 14‐min period. The child was instructed to press a button each time the target stimulus appeared. The computer produced statistics based on the child's performance across all 22 trial blocks. Sustained attention was measured as the number of targets to which the child did not respond (errors of omission‐higher numbers reflecting higher inattention). Inhibitory control was measured as the number of times the child responded to a non‐target (errors of commission‐higher numbers reflecting less inhibitory control). For ease of interpretability, both measures were reversed coded so that higher scores reflect better performance.

##### 2.2.3.2 Working Memory

The Woodcock–Johnson Psycho‐Educational Battery‐Revised (WJ‐R; Woodcock–Johnson 1989) tests individual cognitive abilities and achievement. At 54 months, we used standardized scores on The Memory for Sentences subtest to measure children's working memory. The subtest assessed the child's ability to remember and repeat simple words, phrases, and sentences presented auditorily. Children earned 2 points for repeating an item correctly, 1 point for responses with one error, and 0 points for responses with two or more errors (α = 0.82).

#### 2.2.4 Self‐Reliance

In grades 1, 3, and 5, children were observed for 30‐min over a 2‐h period at the official start of the school day using the COS. Trained coders rated self‐reliance on a 7‐point scale from low to high self‐reliance reflecting children's personal initiative, self‐regulation, autonomy, and persistence and engagement in the classroom. Children high in self‐reliance take responsibility for their materials, actions, and activities, persist in difficult situations, and seek adult assistance only after using their own resources. Children low in self‐reliance lack self‐directedness and initiative in ambiguous or challenging situations thus asking for help frequently, appearing passive, and seeking adult attention rather than demonstrating autonomy. The reliability coefficient across all pairs of coders was 0.76 at Grade 1, 0.86 at Grade 3, and 0.97 at Grade 5.

At age 15, adolescents completed the 30‐item Psychosocial Maturity Inventory (Greenberger 1976) to measure the adolescent's capacity for responsible self‐management. The self‐reliance scale consisted of 10 items with responses on a 4‐point scale ranging from 1 = “*strongly disagree*” to 4 = “*strongly agree*” with higher scores indicating greater feelings of internal control and ability to make decisions without extreme reliance on others (α = 0.71).

#### 2.2.5 Academic Achievement in Middle Childhood and Adolescence

Children's reading and math achievement was assessed at each grade and at age 15 by standardized scores on the Woodcock–Johnson Psycho‐Educational Battery‐Revised (WJ‐R; Woodcock and Johnson 1989).

##### 2.2.5.1 Reading Achievement

The Passage Comprehension subscale was selected to measure reading achievement at grades 3 and 5, and age 15. (α = 0.83; α = 0.81; α = 0.81, respectively). The first four Passage Comprehension items are presented in a multiple‐choice format requiring the subject to point to the picture represented by a phrase. The remaining items measure the subject's skill in reading a short passage and identifying a missing keyword. The task requires the subject to state a word that would be appropriate in the context of the passage. In this modified cloze procedure, the subject must exercise a variety of comprehension and vocabulary skills.

##### 2.2.5.2 Math Achievement

The Applied Problems subscale was selected to measure math achievement at grades 1, 3, 5, and age 15 (α = 0.83; α = 0.81; α = 0.82; α = 0.87, respectively). The test measures the subject's skill in analyzing and solving practical problems in mathematics. To solve the problems, the subject must recognize the procedure to be followed and then perform relatively simple calculations.

#### 2.2.6 Covariates

Specific covariates were added to the analyses because each of them has been shown or hypothesized to correlate with maternal sensitivity and child language. Child‐level covariates included gender, ethnicity/race, temperament, and early cognitive development. At 6 months, mothers reported on their child's temperament using an adaptation of the Infant Temperament Questionnaire (Carey and McDevitt 1978; α = 0.81). At 15 months, trained observers assessed infant cognitive development using the Bayley Mental Development Index (Bayley 1991). Family‐level covariates collected at 1‐month included income‐to‐needs ratio, maternal education, and maternal depression. Mothers’ depressive symptoms were assessed with the Center for Epidemiological Studies Depression Scale (CES‐D; Radloff 1977). The measure consisted of 20 items and each item stated a depressive symptomology. Mothers rated the frequency of each symptomology during the past week on a 4‐point Likert scale (0 = *less than 1 day*; 1 = *1–2 days*; 2 = *3–4 days*; and 3 = *5–7 days*). Scores range from 0 to 60 with a score of 16 or above considered at risk for clinical depression.

### 2.3 Data Analysis Strategy

All models were estimated using Mplus 8.10 (Muthen and Muthen 1997–2018). We took a model‐building approach to develop the full model and then trimmed non‐significant paths from the model to yield a final model for interpretation. The first model included the random intercepts for maternal sensitivity, reading ability, math ability, and self‐reliance. The random intercept for maternal sensitivity was specified as a second‐order factor with three observed indicators at 6, 15, 24, 36, and 54 months, grades 1, 3, and 5, and age 15.5. The first indicator at each time point was fixed to equal 1. Random intercepts for self‐reliance and math abilities were modeled using observations at grades 1, 3, and 5 and age 15.5. Reading ability was modeled using observations at grades 3, and 5 and age 15.5. All covariances between random intercepts were freely estimated. This model had adequate fit (*χ* ^2^[666] = 2192.99, *p *< 0.0001; CFI = 0.919; RMSEA = 0.041 [0.040–0.043]). In this model, there was also a small, non‐significant negative residual variance for one of the maternal sensitivity indicators at 6 months.

The second model built on the previous model specification by including all lagged associations for within‐ and between‐construct associations. These paths were specified on the residuals of the occasion‐specific indicators of the random intercept factors. This model had adequate fit (*χ* ^2^[615] = 1881.39, *p *< 0.0001; CFI = 0.932; RMSEA = 0.039 [0.037–0.041]). In this model, there was a small, non‐significant negative residual variance for one of the maternal sensitivity indicators at 6‐months.

The third model (complete output in Supplementary Output 1) relies on the base of the second model and included influences of a set of time‐invariant covariates (Child Gender, Child Ethnicity, Income/Needs Ratio, Maternal Education, Maternal Depression, Child Temperament, Child Intelligence) on random‐intercepts. Receptive Language and Expressive Language at 36 months were predicted by maternal sensitivity at 24 months. Receptive Language, Expressive Language, Sustained Attention, Inhibitory Control, and Working Memory at 54 months were predicted by Receptive Language and Expressive Language at 36 months and were predicted by maternal sensitivity at 24 months. The child 36 and 54‐month time‐invariant covariates were also regressed on Child Gender, Child Ethnicity, Income/Needs Ratio, Maternal Education, Maternal Depression, Child Temperament, and Child Intelligence. The time‐invariant covariates included at 54 months predicted maternal sensitivity, math ability, and self‐reliance at grade 1. This model also included contemporaneous covariances between time‐invariant covariates and occasion‐specific residuals (i.e., Receptive Language and Expressive Language at 36 months covaried with occasion‐specific residuals of maternal sensitivity at 36 months). This model had adequate fit (*χ* ^2^[1067] = 2353.05, *p *< 0.0001; CFI = 0.937; RMSEA = 0.033 [0.031–0.035]). In this model, the residual variance of the math and reading random intercepts had negative variance estimates.

The final model (complete output in Supplementary Output 2) was specified in general form to be the same as the third model. However, non‐significant paths were trimmed from the model, with the following exceptions. First, we included all time‐invariant influences on the random intercepts as these are theoretically motivated and should be included regardless of whether they are statistically significant. Second, we included all time‐invariant influences on Receptive Language and Expressive Language at 36 months and Receptive Language, Expressive Language, Sustained Attention, Inhibitory Control, and Working Memory at 54 months. Third, all covariances between random intercepts were included. Finally, this model also included contemporaneous covariances between time‐invariant covariates and occasion‐specific residuals. The resulting model had adequate fit (*χ* ^2^[1130] = 2643.05, *p *< 0.0001; CFI = 0.926; RMSEA = 0.035 [0.033–0.037]). There were no inadmissible parameter estimates in this model. Some model parameters that were statistically significant in the previous model were not statistically significant in this model.

**FIGURE 3:**
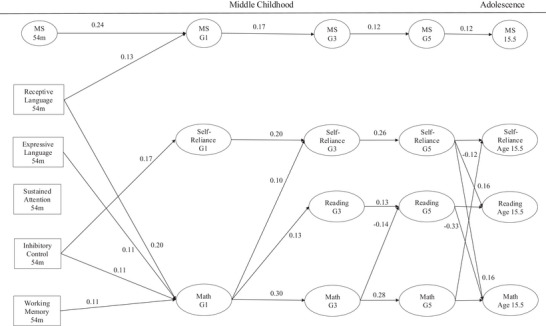
Later maternal sensitivity: Developmental pathways across middle childhood and adolescence. Standardized beta coefficients shown. *p* < 0.05.

As the model is large, it is presented in three figures in the manuscript. Figure 1 shows standardized residual covariances among the random intercepts. Figure 2 shows parameter associations across the 6‐, 15‐, 24‐, 36‐, and 54‐ assessments. Figure 3 includes associations across 54‐months, grades 1, 3, and 5, and age 15.5 years. The complete, full model is provided in Figure S1. Associations with Child Gender, Child Ethnicity, Income/Needs Ratio, Maternal Education, Maternal Depression, Child Temperament, and Child Intelligence are not displayed.

## 3 Results

### 3.1 Descriptive Analyses

Descriptive statistics and correlations for study variables and their covariates are provided in Tables S1 and S2.

### 3.2 Model Results

We first report the amount of variance explained by between‐person associations and within‐person associations. Next, at the between‐person level, we report the significance and the magnitude of correlations between the random intercepts of maternal sensitivity, reading, math, and self‐reliance. Then, we report the developmental pathways that unfolded over four developmental stages including autoregressive and cross‐lagged associations. We also report whether early maternal sensitivity has enduring indirect effects on reading, math, and self‐reliance. Finally, we report the effects of later maternal sensitivity—during early childhood and middle childhood—on these same outcomes after controlling for maternal sensitivity in the preceding period.

#### 3.2.1 Between‐Person and Within‐Person Variance

To explain the between‐ and within‐person variance for study variables, we calculated intraclass correlations. Results showed that the variance accounted for by differences between‐person associations were 78% for maternal sensitivity, 68% for reading, 72% for math, and 27% for self‐reliance. Within‐person associations were 22%, 32%, 28%, and 73%, respectively.

#### 3.2.2 Random Intercepts

The random intercept between two variables represents between‐person differences. Results show that all correlations between the random intercepts were significant. Associations between the random intercept for maternal sensitivity and reading, math, and self‐reliance were small in magnitude (*r* ∼ 0.22). Associations between self‐reliance and reading and math were moderate (*r *∼ 0.40). Associations between reading and math were strong (*r* ∼ 0.80). Figure 1 shows relations between random intercepts.

#### 3.2.3 Developmental Pathways

##### 3.2.3.1 Autoregressive Effects

Autoregressive effects measure the extent to which an individual's deviation from their average (within‐person) score at one‐time point predicts their deviation from that average score at the next time point after accounting for between‐person effects. For maternal sensitivity, autoregressive effects were significant except between 15 and 24 months. Results showed that sensitivity scores from 6 to 54 months ranged from 0.10 to 0.30, respectively, demonstrating time‐varying effects and increasing levels of stability over the period. From 54 months to grade 1, the autoregressive effect was 0.24 which was slightly lower than that of the preceding wave. From grade 1 to age 15.5, autoregressive effects ranged from 0.17 to 0.12, respectively, indicating lower levels of within‐person stability compared to prior periods. For reading, autoregressive effects were significant between grades 3 and 5 indicating increasing stability. For math and self‐reliance, autoregressive effects from grades 1 to 5 increased over their average scores showing moderate stability over the period. Figures 2 and 3 show the autoregressive coefficients across time.

##### 3.2.3.2 Cross‐Lagged Associations

Both receptive and expressive language at 36 months were predicted by occasion specific variance in maternal sensitivity at 24 months. Receptive language and expressive language at 54 months were both positively associated with receptive language and expressive language at 36 months. Sustained attention at 54 months was positively associated with receptive language at 36 months, but negatively associated with expressive language at 36 months. Inhibitory control at 54 months was positively associated with receptive language, but not significantly associated with expressive language at 36 months. Working memory at 54 months was positively associated with receptive language at 36 months, but not significantly associated with expressive language at 36 months. Figure 2 shows these associations.

Grade 1 occasion‐specific maternal sensitivity was predicted by receptive language at 54 months. Grade 1 occasion‐specific self‐reliance was positively predicted by inhibitory control at 54 months. Grade 1 occasion‐specific math ability was positively predicted by receptive language, working memory, and inhibitory control at 54 months. Grade 3 occasion‐specific self‐reliance was predicted by grade 1 occasion‐specific math ability. Grade 3 occasion‐specific reading was positively predicted by grade 1 occasion‐specific math ability. Grade 5 occasion‐specific reading was negatively predicted by grade 3 occasion‐specific math ability. Age 15 occasion‐specific self‐reliance was negatively predicted by grade 5 occasion‐specific math ability. Age 15 occasion‐specific reading was positively predicted by grade 5 occasion‐specific self‐reliance. Age 15 occasion‐specific math ability was positively predicted by grade 5 occasion‐specific self‐reliance and reading. Figure 3 shows these associations.

#### 3.2.4 Indirect Effects

We were also interested in understanding whether maternal sensitivity in the infancy/toddler years has enduring indirect effects on reading, math, and children's self‐reliance through age 15 via early language and EF. For indirect effects, we estimated all possible indirect effects from maternal sensitivity at 24 months to reading, math, and self‐reliance outcomes at grades 1, 3, 5, and age 15. All significant indirect effects are shown in Table 1. There were no significant indirect effects of maternal sensitivity at 24 months on reading outcomes in grades 1, 3, 5, and age 15. There were significant indirect effects of maternal sensitivity at 24 months to math in grades 1 and 3. One set of indirect effects were linked via 36‐ and 54‐month receptive language and expressive language. Another set of indirect effects was linked via 36‐month receptive language and 54 month sustained attention. Finally, there was a significant indirect effect from maternal sensitivity at 24 months to grade 1 math via 36‐month receptive language and 54 month working memory. For self‐reliance, there were significant indirect effects from maternal sensitivity at 24 months to self‐reliance in grades 1, 3, and 5. In each of these indirect effects, maternal sensitivity predicted receptive language at 36 months and that, in turn, predicted sustained attention at 54 months. These effects predicted self‐reliance at grade 1 and the later influences were maintained across time via autoregressive effects across grades 3 and 5. Overall, even though these indirect effects were significant, they were very small in size and not of practical significance.

**TABLE 1:** Indirect effects from maternal sensitivity at 24 months to later child outcomes.

Distal predictor	Intervening variables				Outcome	Indirect effect B (SE)
MS‐24m	RLanguage‐36m	IC‐54m	Self‐Rel‐G1	Self‐Rel‐G3	Self‐Rel‐G5	0.00 (0.00)^a^
MS‐24m	RLanguage‐36m	IC‐54m	Self‐Rel‐G1		Self‐Rel‐G3	0.001 (0.001)
MS‐24m	RLanguage‐36m	RLanguage‐54m	Math‐G1		Math‐G3	0.005 (0.002)
MS‐24m	RLanguage‐36m	IC‐54m	Math‐G1		Math‐G3	0.001 (0.001)
MS‐24m	RLanguage‐36m	WM‐54m	Math‐G1		Math‐G3	0.002 (0.001)
MS‐24m	RLanguage‐36m	RLanguage‐54m			MS‐G1	0.010 (0.004)
MS‐24m	MS‐36m	MS‐54m			MS‐G1	0.016 (0.008)
MS‐24m	RLanguage‐36m	IC‐54m			Self‐Rel‐G1	0.007 (0.002)
MS‐24m	RLanguage‐36m	RLanguage‐54m			Math‐G1	0.015 (0.005)
MS‐24m	RLanguage‐36m	ELanguage‐54m			Math‐G1	0.007 (0.004)
MS‐24m	RLanguage‐36m	IC‐54m			Math‐G1	0.004 (0.002)
MS‐24m	RLanguage‐36m	WM‐54m			Math‐G1	0.006 (0.003)
MS‐24m	MS‐36m				MS‐54m	0.069 (0.023)
MS‐24m	RLanguage‐36m				RLanguage‐54m	0.075 (0.017)
MS‐24m	RLanguage‐36m				ELanguage‐54m	0.065 (0.015)
MS‐24m	ELanguage‐36m				ELanguage‐54m	0.014 (0.005)
MS‐24m	RLanguage‐36m				SusAtt‐54m	−0.03 (0.008)
MS‐24m	RLanguage‐36m				IC‐54m	−0.039 (0.011)
MS‐24m	RLanguage‐36m				WM‐54m	0.059 (0.014)

*Note*: MS‐24m, Maternal Sensitivity‐24 months; MS‐36m, Maternal Sensitivity‐36 months; MS‐54m, Maternal Sensitivity‐54 months; MS‐G1, Maternal Sensitivity‐Grade 1; RLanguage‐36m, Receptive Language‐36 months; ELanguage‐36m, Expressive Language‐36 months; IC‐54m, Inhibitory Control‐54 months; SusAtt‐54m, Sustained Attention‐54 months; Wm‐54m, Working Memory‐54 months; Math‐G1, Math‐Grade 1; Math‐G3, Math‐Grade 3; Self‐Rel‐G1, Self‐Reliance‐Grade 1; Self‐Rel‐G3, Self‐Reliance‐Grade 3; Self‐Rel‐G5, Self‐Reliance‐Grade 5. Unstandardized beta coefficients shown.

^a^ Compound path coefficient is less than 0.001. *p < 0.05; **p < 0.01; ***p < 0.001.

#### 3.2.5 Effects of Later Maternal Sensitivity After Controlling for Early Sensitivity

To test the relative effects of early and later maternal sensitivity on reading, math, and self‐reliance, we modified the specification of the full model to include separate random intercepts for maternal sensitivity through 36 months and after 36 months but leaving all other model specifications unchanged. This model failed to yield an admissible solution owing to the correlation between the early and later maternal sensitivity random intercepts. Thus, we simplified the model to include only the early and later random intercepts for maternal sensitivity and the random intercepts for reading, math, self‐reliance, contemporaneous associations, and residual covariances for latent variable indicators. This model was a good fit to the data, *χ* ^2^(640) = 2020.94, *p* < 0.0001; CFI = 0.926; RMSEA = 0.04 (0.038, 0.042). Associations between random intercepts for early and later maternal sensitivity and reading, math, and self‐reliance are shown in Table 2. All associations were statistically significant. None of the associations with child outcomes significantly differed in magnitude between early and later maternal sensitivity.

**TABLE 2:** Correlations between early and later maternal sensitivity and reading, math, and self‐reliance.

	Early maternal sensitivity	Later maternal sensitivity
Later maternal sensitivity	0.93***	
Reading	0.53***	0.53***
Math	0.51***	0.51***
Self‐reliance	0.49***	0.54***

*Note*: All correlations significant at *p *< 0.001.

## 4 Discussion

This study used a transactional model of development to examine the relative contributions of both early and later maternal sensitivity in the development of multiple domains of children's cognition and achievement over 15 years. We explored the temporal sequence of development for mothers’ sensitivity and children's language, EF, reading and math achievement, and self‐reliance. We hoped not only to assess the contributions of early and later sensitivity but also to determine whether these effects demonstrated stable, trait‐like associations, state‐like, time‐varying associations, or a combination of both associations. Our findings show that relations between maternal sensitivity and these child outcomes are best characterized by stable, trait‐like associations that persist over time with limited state‐like time‐varying associations. Given the nature of these trait‐like associations over four developmental stages, we extend prior research by demonstrating that the effects of both early and later maternal sensitivity are important to children's cognition and achievement.

Based on prior research, we hypothesized that early language and EF would mediate the effects of infant/toddler maternal sensitivity on academic achievement and self‐reliance. The indirect effects among these variables were so small that we can offer no support for the indirect effects of early maternal sensitivity acting through the child's early language and EF. Our second hypothesis—that later maternal sensitivity, after controlling for earlier effects, is associated with children's academic achievement and self‐reliance behaviors was supported from a trait perspective. Our third hypothesis—that mothers’ sensitivity and children's cognition and achievement would be reciprocally related over time—received only minimal support. Mothers’ sensitivity at 24 months positively predicted children's receptive language at 36 months, and in turn, children's receptive language at 54 months positively predicted mothers’ sensitivity at grade 1.

This study's findings demonstrate that relations in the mother–child dyad reflect stable, between‐person differences, that these trait‐like differences begin very early in life, and they are sustained over four consecutive developmental stages. Importantly, the trait‐like associations for early and later sensitivity and child outcomes appear to be similar in magnitude. More specifically, these findings demonstrate that mothers who begin parenting with higher levels of sensitivity, compared to other mothers, have children with stronger language and EF skills in early childhood. In middle childhood and adolescence, these children score higher on standardized tests of reading and math, and they also demonstrate stronger self‐reliant behaviors. Mothers who begin parenting with lower levels of sensitivity have children whose outcomes may not be as promising, in accordance with the findings of Deans (2020).

Much of what we know about the effects of mothers’ sensitive parenting on child outcomes stems from analytical methods that combine between‐person and within‐person associations. When these effects are not disaggregated, studies often report more dynamic, cross‐lagged predictions and conflated coefficients suggesting that time‐specific interventions may address deficiencies. For example, results may show that mothers’ sensitivity at a particular point in development predicts positive change in a skill or behavior at the next wave. Because these methods combine between‐ and within‐person effects, such a finding may not apply to an individual mother–child dyad.

Prior research has reported stability in mothers’ sensitive parenting during infancy (e.g., Behrens, Hart, and Parker 2012; Bigelow et al. 2010), and in one instance, from infancy to adolescence (Bornstein and Putnick 2021). In this study, using advanced analytical methods to establish autoregressive effects at the within‐person level, mothers’ sensitive parenting exhibited very little stability from 6 to 24 months. From 36 to 54 months and from 54 months to first grade, stability increased to moderate levels. From first grade to age 15, however, stability declined. When between‐ and within‐person differences in mothers’ sensitivity are disaggregated, there is some evidence for fluctuations in individual mothers’ sensitivity across a 15‐year period.

We were also interested in uncovering the mechanisms through which maternal sensitivity influences child outcomes. Maternal sensitivity when the child is 24 months‐of‐age initiates a series of developmental transactions predicting children's language at age 36 months, which in turn predicts multiple facets of EF at 54 months which in turn predicts math ability and classroom self‐reliance at grade 1. This cascade informs our understanding of the temporal sequence of effects for mothers’ sensitivity and children's early cognition that takes place by the transition to formal schooling.

Contrary to our expectations, mothers’ early sensitivity did not predict age 54‐month EF. Instead, receptive language at age 36 months mediates relations between early sensitivity and preschool EF. Prior research has reported equivocal findings over the first 5 years as to whether sensitivity directly predicts EF (e.g., Frick et al.), whether language mediates relations (e.g., Matte‐Gagné and Bernier 2011), or whether no evidence exists for associations between sensitivity and EF (Jónsdóttir et al. 2024). Our finding that vocabulary comprehension at age 36 months mediates the effects of early sensitivity on preschool EF is in line with the premise that children's understanding of spoken language helps them to internalize word meanings to support attention control and working memory capacity (Diaz and Berk 2016; Vygotsky 1987). This finding also supports theoretical claims that early language complexity acts as a precursor to EF (Zelazo et al. 2003). At least at this stage, receptive language may be more important than expressive language for sustaining attention and working memory capacity.

Additionally, we observed important reciprocal effects between maternal sensitivity and children's receptive language between age 24 months and first grade. This finding demonstrates how mothers’ sensitivity shapes and is shaped by children's receptive language skills between toddlerhood and formal school entry. Using this same dataset, Barnett et al. (2012) found that maternal sensitivity predicted early receptive language, although they were limited in their ability to reveal reciprocal effects. Methodologies used in this study may have led to a more nuanced understanding of the longitudinal associations and reciprocal influences between the mother's sensitivity and children's receptive language in this early period of development.

Prior research reported that early sensitivity has enduring indirect effects on later academic performance (Fraley, Roisman, and Haltigan 2013). Although we found that early sensitivity has significant indirect effects on child outcomes during preschool and middle childhood, these effects are very small and not of practical significance. Instead, we found consistent trait associations for maternal sensitivity, reading and math achievement, and self‐reliance in middle childhood and adolescence, and no evidence for state‐like, time‐varying associations for maternal sensitivity and these same child outcomes during this period.

Beyond relations with maternal sensitivity, our analyses reveal both trait‐like and state‐like associations for reading, math, and self‐reliance. For trait associations, children who score higher in reading comprehension also score higher in math problem‐solving, and they also demonstrate stronger self‐reliant behaviors in the classroom. After accounting for these stable relations, several noteworthy within‐child associations extend prior research by demonstrating a complex set of temporal interdependencies. Higher than average first grade math problem‐solving skills predict higher than average third‐grade reading comprehension skills. This finding is informative because prior research has sought to determine whether math and reading co‐develop (e.g., Bailey et al. 2020; Hübner et al. 2022) or whether one is the leading indicator of the other (Erbeli et al. 2020; Rinne, Ye, and Jordan 2020; Cameron et al. 2019). At least early in middle childhood, math problem‐solving skills appear to be the leading indicator of later reading comprehension skills. Interestingly, the direction of effects reversed later in childhood. Higher than average third grade math problem‐solving skills predict lower than average fifth grade reading comprehension skills. Similarly, in fifth‐grade, higher than average reading comprehension skills predict lower than average math problem‐solving skills. This pattern of alternating interdependencies has been reported by Gnambs and Lockl (2023) using similar analytical methods that disaggregate between‐ and within‐child associations. In general, these associations have received limited attention at the individual child level when examining developmental growth patterns between academic domains.

There are several explanations for why students with higher‐than‐average competencies in one domain might have correspondingly lower than average competencies in another domain at the next assessment. These effects could be related to resource allocation of effort (Kahneman 1973), motivational factors (Ryan and Deci 2000), or environmental factors that favor one academic domain over another (Gnambs and Lockl 2023). In the case of cognitive resource allocation, for example, students with lower math achievement may exert greater cognitive resources to increase math scores at the expense of resources dedicated to reading comprehension. Additionally, a student who scores high in one domain may be intrinsically motivated to expend more effort in this subject due to self‐concept (Brunner, Lüdtke, and Trautwein 2008; Dennissen et al. 2007; Gogol et al. 2017) or because of praise or instructional emphasis (Gnambs and Lockl 2023). Of course, these associations could be spurious due to unmeasured confounders or other methodological issues.

Lastly, our findings reveal reciprocal relations between math problem‐solving and classroom self‐reliance over middle childhood and adolescence. A child who scores higher than average in first‐grade math also displays higher than average self‐reliance 2 years later. Higher math performance over average may build the child's confidence and self‐esteem which may lead to the demonstration of more self‐reliant behaviors in the classroom (Ryan and Deci 2000). By the end of middle childhood, we found that a child's higher than average self‐reliance forecast higher than average math problem‐solving in adolescence. Children's ability to problem‐solve is fundamental to learning advanced mathematical procedures. It is also essential for demonstrating self‐reliant behaviors because problem‐solving has been shown to foster independence and responsibility—qualities that teachers value both academically and behaviorally (McClelland, Acock, and Morrison 2006; Rimm‐Kaufman, Pianta, and Cox 2000). These findings provide a case for early interventions targeting math problem‐solving, which later in childhood, may support confidence building and independence in the classroom. In turn, the experience of problem‐solving with peers and teachers in the classroom may also support the cognitive processes that support math problem‐solving in adolescence.

### 4.1 Theoretical Significance

The nature of persistent stable, trait‐like associations for mothers’ sensitivity and child outcomes across development suggests that the effects of sensitive parenting are most likely *enduring* because of its *consistency* over development. Maternal sensitivity later in childhood appears to be as important as sensitivity early in development. These findings not only inform us about the long‐term associations for mothers’ sensitivity and child outcomes, but they also enlighten us about the potential long‐term possibilities for interventions targeted to mothers who begin parenthood with less than adequate levels of sensitivity.

This study was originally guided by Sameroff's model of transactional development (2009) which emphasizes the dynamic and reciprocal nature of the child and environment over time. Using this theoretical foundation, we expected to find evidence that throughout the first 15 years of a child's life, mothers’ sensitivity shapes and is shaped by children's cognitive growth. Methodologically, such transactions would be expected to demonstrate within‐subject, state‐like time‐varying associations between mother and child at adjacent assessment points. In fact, our findings show few state‐like effects over time. Instead, these findings are more suggestive of trait associations that reflect individual differences and trait theories of development.

### 4.2 Strengths and Limitations

A major strength of this study was the examination of nine waves of data over four consecutive developmental periods—infancy/toddlerhood, early childhood, middle childhood, and adolescence—using repeated measures of maternal sensitivity and multiple measures of children's cognition and academic performance. The advanced analytical methods in this study extend previous research on mothers’ sensitivity and child outcomes leading to a more nuanced interpretation of these relations. In addition, the findings further demonstrate the utility of the NICHD‐SECCYD dataset for understanding the developmental pathways by which early and later experiences together influence child outcomes as suggested by Vandell and Gülseven (2023). Future studies might want to use the modeling approach adopted here to examine how father–child and mother–child interactions measured over consecutive developmental stages can have mutual but possibly unique influences on child outcomes and to examine whether paths differ for boys and girls.

There are several limitations. First, because we did not examine time‐variant risk factors, it is possible that mothers with high or average sensitivity could be negatively impacted at a time when risk presents at the family‐or child‐level potentially undermining the stable, trait associations found here. Second, random intercept cross‐lagged panel models (RI‐CLPM) require that all variables have three or more repeated measures at consecutive time points over time. Although we measured maternal sensitivity over nine waves—a key measure in this study—the NICHD‐SECCYD dataset did not include three or more repeated measures of language and EF during the first 5 years of life. Although some of our early results did replicate prior research, it is possible that the cross‐domain coefficients we report could be inflated. At the same time, our study did meet this repeated measurement requirement during middle childhood and adolescence, allowing us to examine between‐ and within‐person associations during middle childhood—a period that has received less attention for considering relations between a mother's sensitivity and her child's academic performance. Third, we examined only the contribution of maternal sensitivity to child outcomes. Maternal sensitivity is only one of many important variables of children's experiences that affect developmental outcomes. Fourth, the role of culture in the mother–child dyad was beyond the scope of this study. Cultural belief systems may play a role in parental cognitions about parenting practices, which in turn, may influence children's actions and ideas (Bornstein 2009). The findings in this study may or may not support relations between the mother–child dyad when cultural differences are considered.

### 4.3 Implications

These findings once again highlight the importance of investing in resources that facilitate and strengthen positive parenting before birth and in infancy to help set the course for children's subsequent cognitive development. The consistent stable nature of associations between maternal sensitivity child outcomes over 15 years beginning in infancy suggests that we may be able to forecast the effects of a mother's sensitivity on her child's cognitive and academic performance at 6 months‐of‐age. Mothers with high levels of stable sensitivity are likely to have children with positive developmental outcomes in adolescence. For mothers who begin parenting with less than adequate skills, early and continuing interventions might be necessary. Language and literacy interventions may be most effective when programs strengthen sensitive parenting skills even into middle childhood and adolescence (e.g., Guevara et al. 2020). Also, because our data demonstrate the importance of self‐reliance in children's academic success, later parenting interventions might focus on helping parents learn how to encourage and support their children in taking personal responsibility for their own achievement.

### 4.4 Conclusions

The results of this exploratory study demonstrate that maternal sensitivity at all points during children's development—early as well as later—is associated with cognitive development and academic achievement. The predominance of trait associations in mother–child outcomes demonstrates that the *level* of mothers’ early ability to sensitively parent—whether positive or negative—is maintained over four consecutive developmental stages. The stable nature of maternal sensitivity also may suggest that mothers who are consistently aware of their child's changing needs likely also acknowledge the need to demonstrate age‐appropriate sensitive behaviors as children seek more autonomy in middle childhood and adolescence. By explicating the pathways through which maternal sensitivity influences child outcomes, this study's findings support the need for very early and sustained intervention, but also acknowledge that multiple opportunities exist to change the trajectory of a child's success (Supplementary).

## Ethics Statement

This study was carried out in accordance with the recommendations of the Institutional Review Board (IRB) of Temple University with written informed consent from all subjects. The protocol was approved by the IRB of Temple University.

## Conflicts of Interest

The authors declare no conflicts of interest.

## Supporting information



Supporting information

Supporting information

Supporting information

Supporting information

Supporting information

Supporting information

Supporting information

## Data Availability

The data drawn from the NICHD SECCYD archive that support the findings of this study are openly available at http://www.icpsr.umich.edu/web/ICPSR/series/00233. The data generated for this analysis that support the findings of this study are available from the corresponding author upon request.

## References

[desc13594-bib-0001] Andersen, S. H. , L. Steinberg , and J. Belsky . 2021. “Beyond Early Years Versus Adolescence: The Interactive Effect of Adversity in Both Periods on Life‐Course Development.” Developmental Psychology 57, no. 11: 1958–1967. 10.1037/dev0001247. 34914456

[desc13594-bib-0002] Andersen, S. L. 2003. “Trajectories of Brain Development: Point of Vulnerability or Window of Opportunity?” Neuroscience & Biobehavioral Reviews 27, no. 1–2: 3–18. 10.1016/s0149-7634(03)00005-8. 12732219

[desc13594-bib-0003] Bailey, D. H. , Y. Oh , G. Farkas , P. Morgan , and M. Hillemeier . 2020. “Reciprocal Effects of Reading and Mathematics? Beyond the Cross‐Lagged Panel Model.” Developmental Psychology 56, no. 5: 912–921. 10.1037/dev0000902. 32105116

[desc13594-bib-0004] Barnett, M. A. , H. Gustafsson , M. Deng , W. R. Mills‐Koonce , and M. Cox . 2012. “Bidirectional Associations Among Sensitive Parenting, Language Development, and Social Competence.” Infant and Child Development 21, no. 4: 374–393. 10.1002/icd.1750. 25126021 PMC4128493

[desc13594-bib-0005] Bayley, N. 1991. Bayley Scales of Infant Development: Standardization Version. 2nd ed. New York, NY: Psychological Corporation.

[desc13594-bib-0006] Behrens, K. Y. , S. L. Hart , and A. C. Parker . 2012. “Maternal Sensitivity: Evidence of Stability Across Time, Contexts, and Measurement Instruments.” Infant and Child Development 21, no. 4: 348–355. 10.1002/icd.1747.

[desc13594-bib-0007] Belsky, J. , and M. de Haan . 2011. “Annual Research Review: Parenting and Children's Brain Development: The End of the Beginning.” Journal of Child Psychology and Psychiatry 52, no. 4: 409–428. 10.1111/j.1469-7610.2010.02281.x. 20626527

[desc13594-bib-0008] Bernier, A. , M. H. Beauchamp , and C. Cimon‐Paquet . 2020. “From Early Relationships to Preacademic Knowledge: A Sociocognitive Developmental Cascade to School Readiness.” Child Development 91, no. 1: e134–e145. 10.1111/cdev.13160. 30295317

[desc13594-bib-0009] Bernier, A. , S. M. Carlson , and N. Whipple . 2010. “From External Regulation to Self‐Regulation: Early Parenting Precursors of Young Children's Executive Functioning.” Child Development 81, no. 1: 326–339. 10.1111/j.1467-8624.2009.01397.x. 20331670

[desc13594-bib-0010] Bernier, A. , F. Dégeilh , É. Leblanc , V. Daneault , H. N. Bailey , and M. H. Beauchamp . 2019. “Mother–Infant Interaction and Child Brain Morphology: A Multidimensional Approach to Maternal Sensitivity.” Infancy 24, no. 2: 120–138. 10.1111/infa.12270. 32677197

[desc13594-bib-0011] Bernier, A. , C. A. McMahon , and R. Perrier . 2017. “Maternal Mind‐Mindedness and Children's School Readiness: A Longitudinal Study of Developmental Processes.” Developmental Psychology 53, no. 2: 210–221. 10.1037/dev0000225. 27709997

[desc13594-bib-0012] Berry, D. , and M. T. Willoughby . 2016. “On the Practical Interpretability of Cross‐Lagged Panel Models: Rethinking a Developmental Workhorse.” Child Development 88, no. 4: 1186–1206. 10.1111/cdev.12660. 27878996

[desc13594-bib-0013] Bick, J. , and C. A. Nelson . 2016. “Early Experience and Brain Development.” Wiley Interdisciplinary Reviews: Cognitive Science 8, no. 1‐2: 177–196. 10.1002/wcs.1387. PMC8103659 27906514

[desc13594-bib-0014] Bigelow, A. E. , K. MacLean , J. Proctor , T. Myatt , R. Gillis , and M. Power . 2010. “Maternal Sensitivity throughout Infancy: Continuity and Relation to Attachment Security.” Infant Behavior and Development 33, no. 1: 50–60. 10.1016/j.infbeh.2009.10.009. 20004022

[desc13594-bib-0015] Bindman, S. W. , E. M. Pomerantz , and G. I. Roisman . 2015. “Do Children's Executive Functions Account for Associations Between Early Autonomy‐Supportive Parenting and Achievement Through High School?” Journal of Educational Psychology 107, no. 3: 756–770. 10.1037/edu0000017. 26366009 PMC4562792

[desc13594-bib-0016] Blankenship, T. L. , M. A. Slough , S. D. Calkins , K. Deater‐Deckard , J. Kim‐Spoon , and M. A. Bell . 2019. “Attention and Executive Functioning in Infancy: Links to Childhood Executive Function and Reading Achievement.” Developmental Science 22, no. 6: 1–14. 10.1111/desc.12824. PMC6722030 30828908

[desc13594-bib-0017] Bornstein, M. H. 2009. “Toward a Model of Culture‐Parent‐Child Transactions.” In The Transactional Model of Development: How Children and Contexts Shape each Other, edited by A. Sameroff , 139–161. Washington, DC: American Psychological Association.

[desc13594-bib-0018] Bornstein, M. H. , C.‐S. Hahn , D. L. Putnick , and R. M. Pearson . 2018. “Stability of Core Language Skill From Infancy to Adolescence in Typical and Atypical Development.” Science Advances 4, no. 11: 1–12. 10.1126/sciadv.aat7422. PMC6248911 30474055

[desc13594-bib-0019] Bornstein, M. H. , and D. L. Putnick . 2021. “Dyadic Development in the Family: Stability in Mother–Child Relationship Quality From Infancy to Adolescence.” Journal of Family Psychology 35, no. 4: 445–456. 10.1037/fam0000794. 32757574 PMC7865016

[desc13594-bib-0020] Bornstein, M. H. , D. L. Putnick , Y. Bohr , M. Abdelmaseh , C. Y. Lee , and G. Esposito . 2020. “Maternal Sensitivity and Language in Infancy Each Promotes Child Core Language Skill in Preschool.” Early Childhood Research Quarterly 51: 483–489. 10.1016/j.ecresq.2020.01.002. 32280159 PMC7147483

[desc13594-bib-0021] Bowlby, J. 1982. Attachment and Loss, 2nd ed., Vol. 1. New York, NY: Basic Books.

[desc13594-bib-0022] Bradley, R. H. , and A. L. Pennar . 2011. “Maternal Sensitivity in Middle Childhood.” In Maternal Sensitivity: A Scientific Foundation for Practice, edited by D. W. Davis and M. C. Logsdon . Happauge, NY: Nova Science Publishers.

[desc13594-bib-0023] Bridgewater, J. M. , and T. M. Yates . 2020. “Academic Implications of Insensitive Parenting: A Mediating Path Through Children's Relational Representations.” Journal of Applied Developmental Psychology 71: 101201. 10.1016/j.appdev.2020.101201.

[desc13594-bib-0024] Brunner, M. , O. Lüdtke , and U. Trautwein . 2008. “The Internal/External Frame of Reference Model Revisited: Incorporating General Cognitive Ability and General Academic Self‐Concept.” Multivariate Behavioral Research 43, no. 1: 137–172. 10.1080/00273170701836737. 26788975

[desc13594-bib-0025] Burchinal, M. R. , D. L. Vandell , and J. Belsky . 2014. “Is the Prediction of Adolescent Outcomes From Early Child Care Moderated by Later Maternal Sensitivity? Results From the NICHD Study of Early Child Care and Youth Development.” Developmental Psychology 50, no. 2: 542–553. 10.1037/a0033709. 23937381 PMC4718076

[desc13594-bib-0026] Buttelmann, F. , and J. Karbach . 2017. “Development and Plasticity of Cognitive Flexibility in Early and Middle Childhood.” Frontiers in Psychology 8: 1–6. 10.3389/fpsyg.2017.01040. 28676784 PMC5476931

[desc13594-bib-0027] Cameron, C. E. , H. Kim , R. J. Duncan , D. R. Becker , and M. M. McClelland . 2019. “Bidirectional and Co‐Developing Associations of Cognitive, Mathematics, and Literacy Skills During Kindergarten.” Journal of Applied Developmental Psychology 62: 135–144. 10.1016/j.appdev.2019.02.004.

[desc13594-bib-0028] Carey, W. B. , and S. C. McDevitt . 1978. “Infant Temperament Questionnaire–Revised.” APA PsycTests. 10.1037/t05932-000.

[desc13594-bib-0029] Cimon‐Paquet, C. , A. Bernier , C. Matte‐Gagné , and G. A. Mageau . 2020. “Early Maternal Autonomy Support and Mathematical Achievement Trajectories During Elementary School.” Learning and Individual Differences 79: 101855. 10.1016/j.lindif.2020.101855.

[desc13594-bib-0030] Conway, A. 2020. “Longitudinal Associations Between Parenting and Inattention, Impulsivity, and Delay of Gratification in Preschool‐Aged Children: The Role of Temperamental Difficultness and Toddler Attention Focusing.” Developmental Neuropsychology 45, no. 5: 309–329. 10.1080/87565641.2020.1797042. 32791853

[desc13594-bib-0031] Deans, C. L. 2020. “Maternal Sensitivity, Its Relationship With Child Outcomes, and Interventions That Address It: A Systematic Literature Review.” Early Child Development and Care 190, no. 2: 252–275. 10.1080/03004430.2018.1465415.

[desc13594-bib-0032] Denissen, J. J. , N. R. Zarrett , and J. S. Eccles . 2007. “I Like to Do It, I'm Able, and I Know I am: Longitudinal Couplings Between Domain‐Specific Achievement, Self‐Concept, and Interest.” Child Development 78, no. 2: 430–447. 10.1111/j.1467-8624.2007.01007.x. 17381782

[desc13594-bib-0033] Diaz, R. M. , and L. E. Berk . 2016. Private Speech: From Social Interaction to Self‐Regulation, edited by R. M. Diaz and L. E. Berk , 1st ed., Mahwah, NJ: Lawrence Erlbaum Associates, Inc.

[desc13594-bib-0034] Enders, C. , and D. Bandalos . 2001. “The Relative Performance of Full Information Maximum Likelihood Estimation for Missing Data in Structural Equation Models.” Structural Equation Modeling: A Multidisciplinary Journal 8, no. 3: 430–457. 10.1207/s15328007sem0803_5.

[desc13594-bib-0035] Erbeli, F. , Q. Shi , A. R. Campbell , S. A. Hart , and S. Woltering . 2020. “Developmental Dynamics Between Reading and Math in Elementary School.” Developmental Science 24, no. 1: 1–14. 10.1111/desc.13004. PMC7725923 32524716

[desc13594-bib-0036] Fay‐Stammbach, T. , D. J. Hawes , and P. Meredith . 2014. “Parenting Influences on Executive Function in Early Childhood: A Review.” Child Development Perspectives 8, no. 4: 258–264. 10.1111/cdep.12095.

[desc13594-bib-0037] Fenesy, M. C. , and S. S. Lee . 2018. “Executive Functioning Mediates Predictions of Youth Academic and Social Development From Parenting Behavior.” Developmental Neuropsychology 43, no. 8: 729–750. 10.1080/87565641.2018.1525384. 30299975 PMC6391311

[desc13594-bib-0038] Fraley, R. C. , G. I. Roisman , and J. D. Haltigan . 2013. “The Legacy of Early Experiences in Development: Formalizing Alternative Models of How Early Experiences Are Carried Forward Over Time.” Developmental Psychology 49, no. 1: 109–126. 10.1037/a0027852. 22448982 PMC13152673

[desc13594-bib-0039] Frick, M. A. , T. Forslund , and K. C. Brocki . 2019. “Does Child Verbal Ability Mediate the Relationship Between Maternal Sensitivity and Later Self‐Regulation? A Longitudinal Study From Infancy to 4 Years.” Scandinavian Journal of Psychology 60, no. 2: 97–105. 10.1111/sjop.12512. 30625240

[desc13594-bib-0040] Gilmore, J. H. , R. C. Knickmeyer , and W. Gao . 2018. “Imaging Structural and Functional Brain Development in Early Childhood.” Nature Reviews Neuroscience 19, no. 3: 123–137. 10.1038/nrn.2018.1. 29449712 PMC5987539

[desc13594-bib-0041] Gnambs, T. , and K. Lockl . 2023. “Bidirectional Effects Between Reading and Mathematics Development Across Secondary School.” Zeitschrift Für Erziehungswissenschaft 26, no. 2: 345–371. 10.1007/s11618-022-01108-w.

[desc13594-bib-0042] Gogol, K. , M. Brunner , R. Martin , F. Preckel , and T. Goetz . 2017. “Affect and Motivation Within and Between School Subjects: Development and Validation of an Integrative Structural Model of Academic Self‐Concept, Interest, and Anxiety.” Contemporary Educational Psychology 49: 46–65. 10.1016/j.cedpsych.2016.11.003.

[desc13594-bib-0043] Greenberger, E. R. Josselson , C. Knerr , and B. Knelt . 1975. “The Measurement and Structure of Psychosocial Maturity.” Journal of Youth and Adolescence 4, no. 2: 127–143. 24414543 10.1007/BF01537437

[desc13594-bib-0044] Guevara, J. P. , D. Erkoboni , M. Gerdes , et al. 2020. “Effects of Early Literacy Promotion on Child Language Development and Home Reading Environment: A Randomized Controlled Trial.” Journal of Pediatrics 221: 1–7. 10.1016/j.ympdx.2020.100020. PMC10236559 37332625

[desc13594-bib-0045] Hamaker, E. L. , R. M. Kuiper , and R. P. Grasman . 2015. “A Critique of the Cross‐Lagged Panel Model.” Psychological Methods 20, no. 1: 102–116. 10.1037/a0038889. 25822208

[desc13594-bib-0090] Hamaker, E. L. 2023. “The Within‐Between Dispute in Cross‐Lagged Panel Research and how to Move Forward.” Psychological Methods. Advance online publication. 10.1037/met0000600. 37902677

[desc13594-bib-0046] Hirsh‐Pasek, K. , and M. Burchinal . 2006. “Mother and Caregiver Sensitivity Over Time: Predicting Language and Academic Outcomes With Variable‐ and Person‐Centered Approaches.” Merrill–Palmer Quarterly 52, no. 3: 449–485. 10.1353/mpq.2006.0027.

[desc13594-bib-0047] Hübner, N. , C. Merrell , H. Cramman , J. Little , D. Bolden , and B. Nagengast . 2022. “Reading to Learn? The Co‐Development of Mathematics and Reading During Primary School.” Child Development 93, no. 6: 1760–1776. 10.1111/cdev.13817. 35730926

[desc13594-bib-0048] Ilyka, D. , M. H. Johnson , and S. Lloyd‐Fox . 2021. “Infant Social Interactions and Brain Development: A Systematic Review.” Neuroscience and Biobehavioral Reviews 130: 448–469. 10.1016/j.neubiorev.2021.09.001. 34506843 PMC8522805

[desc13594-bib-0049] Jónsdóttir, L. K. , T. Forslund , M. A. Frick , A. Frick , E. J. Heeman , and K. C. Brocki . 2024. “A Challenge to the Expected: Lack of Longitudinal Associations Between the Early Caregiving Environment, Executive Functions in Toddlerhood, and Self‐Regulation at 6 Years.” Developmental Science 27, no. 5: 1–15. 10.1111/desc.13526. 38712829

[desc13594-bib-0050] Kahneman, D. 1973. Attention and Effort. Englewood Cliffs, NJ: Prentice‐Hall.

[desc13594-bib-0051] Kuhl, P. K. 2011. “Social Mechanisms in Early Language Acquisition: Understanding Integrated Brain Systems Supporting Language.” In The Oxford handbook of social neuroscience, edited by J. Decety and J. T. Cacioppo , 649–667. New York, NY: Oxford University Press. 10.1093/oxfordhb/9780195342161.013.0043.

[desc13594-bib-0052] Lewis, M. 1998. Altering Fate: Why the Past Does Not Predict the Future. New York: Guilford Press.

[desc13594-bib-0053] Lucas, R. E. 2023. “Why the Cross‐lagged Panel Model Is Almost Never the Right Choice.” Advances in Methods and Practices in Psychological Science 6, no. 1: 1–22. 10.1177/25152459231158378.

[desc13594-bib-0054] Lyons‐Ruth, K. , P. Pechtel , S. A. Yoon , C. M. Anderson , and M. H. Teicher . 2016. “Disorganized Attachment in Infancy Predicts Greater Amygdala Volume in Adulthood.” Behavioural Brain Research 308: 83–93. 10.1016/j.bbr.2016.03.050. 27060720 PMC5017306

[desc13594-bib-0055] Madigan, S. , H. Prime , S. A. Graham , et al. 2019. “Parenting Behavior and Child Language: A Meta‐Analysis.” Pediatrics 144, no. 4: 1–12. 10.1542/peds.2018-3556. 31551396

[desc13594-bib-0056] Magro, S. W. , M. D. Nivison , M. M. Englund , and G. I. Roisman . 2022. “The Quality of Early Caregiving and Teacher‐Student Relationships in Grade School Independently Predict Adolescent Academic Achievement.” International Journal of Behavioral Development 47, no. 2: 158–168. 10.1177/01650254221137511. 36874534 PMC9983819

[desc13594-bib-0057] Marshall, P. J. , and J. W. Kenney . 2009. “Biological Perspectives on the Effects of Early Psychosocial Experience.” Developmental Review 29, no. 2: 96–119. 10.1016/j.dr.2009.05.001.

[desc13594-bib-0058] Masek, L. R. , B. T. M. McMillan , S. J. Paterson , C. S. Tamis‐LeMonda , R. M. Golinkoff , and K. Hirsh‐Pasek . 2021. “Where Language Meets Attention: How Contingent Interactions Promote Learning.” Developmental Review 60: 100961. 10.1016/j.dr.2021.100961.

[desc13594-bib-0059] Masten, A. S. , and D. Cicchetti . 2010. “Developmental Cascades.” Development and Psychopathology 22, no. 3: 491–495. 10.1017/s0954579410000222. 20576173

[desc13594-bib-0060] Matte‐Gagné, C. , and A. Bernier . 2011. “Prospective Relations Between Maternal Autonomy Support and Child Executive Functioning: Investigating the Mediating Role of Child Language Ability.” Journal of Experimental Child Psychology 110, no. 4: 611–625. 10.1016/j.jecp.2011.06.006. 21798554

[desc13594-bib-0061] McClelland, M. M. , A. C. Acock , and F. J. Morrison . 2006. “The Impact of Kindergarten Learning‐Related Skills on Academic Trajectories at the End of Elementary School.” Early Childhood Research Quarterly 21, no. 4: 471–490. 10.1016/j.ecresq.2006.09.003.

[desc13594-bib-0062] Mirsky, A. F. , B. J. Anthony , C. C. Duncan , M. B. Ahearn , and S. G. Kellam . 1991. “Analysis of the Elements of Attention: A Neuropsychological Approach.” Neuropsychology Review 2, no. 2: 109–145. 10.1007/bf01109051. 1844706

[desc13594-bib-0063] Muthén, L. K. , and B. O. Muthén . 1998‐2018. Mplus User's Guide. 8th ed. Los Angeles, CA: Muthén & Muthén.

[desc13594-bib-0064] NICHD Early Child Care Research Network . 2008. “Mothers' and Fathers' Support for Child Autonomy and Early School Achievement.” Developmental Psychology 44, no. 4: 895–907. 10.1037/0012-1649.44.4.895. 18605822

[desc13594-bib-0065] Phelps, E. A. , and J. E. LeDoux . 2005. “Contributions of the Amygdala to Emotion Processing: From Animal Models to Human Behavior.” Neuron 48, no. 2: 175–187. 10.1016/j.neuron.2005.09.025. 16242399

[desc13594-bib-0066] Raby, K. L. , G. I. Roisman , R. C. Fraley , and J. A. Simpson . 2015. “The Enduring Predictive Significance of Early Maternal Sensitivity: Social and Academic Competence Through Age 32 Years.” Child Development 86, no. 3: 695–708. 10.1111/cdev.12325. 25521785 PMC4428971

[desc13594-bib-0067] Radloff, L. S. 1977. “The CES‐D Scale: A Self‐Report Depression Scale for Research in the General Population.” Applied Psychological Measurement 1: 385–401.

[desc13594-bib-0068] Reynell, J. , and C. P. Gruber . 1990. Reynell Developmental Language Scales: Manual . Los Angeles, CA: Western Psychological Services.

[desc13594-bib-0069] Rimm‐Kaufman, S. E. , R. C. Pianta , and M. J. Cox . 2000. “Teachers' Judgments of Problems in the Transition to Kindergarten.” Early Childhood Research Quarterly 15, no. 2: 147–166. 10.1016/s0885-2006(00)00049-1.

[desc13594-bib-0070] Rinne, L. F. , A. Ye , and N. C. Jordan . 2020. “Development of Arithmetic Fluency: A Direct Effect of Reading Fluency?” Journal of Educational Psychology 112, no. 1: 110–130. 10.1037/edu0000362.

[desc13594-bib-0071] Rowe, M. L. , G. B. Ramani , and E. M. Pomerantz . 2016. “Parental Involvement and Children's Motivation and Achievement: A Domain‐Specific Perspective.” In Handbook of Motivation at School 2nd ed., 459–476. Philadelphia, PA: Taylor and Francis Inc.

[desc13594-bib-0072] Rubin, R. D. , P. D. Watson , M. C. Duff , and N. J. Cohen . 2014. “The Role of the Hippocampus in Flexible Cognition and Social Behavior.” Frontiers in Human Neuroscience 8: 1–15. 10.3389/fnhum.2014.00742. 25324753 PMC4179699

[desc13594-bib-0073] Ryan, R. M. , and E. L. Deci . 2000. “Self‐Determination Theory and the Facilitation of Intrinsic Motivation, Social Development, and Well‐Being.” American Psychologist 55, no. 1: 68–78. 10.1037/0003-066x.55.1.68. 11392867

[desc13594-bib-0074] Sameroff, A. J. 2009. The Transactional Model of Development: How Children and Contexts Shape Each Other. Washington, D.C.: American Psychological Association.

[desc13594-bib-0075] Silinskas, G. , and E. Kikas . 2019. “Math Homework: Parental Help and Children's Academic Outcomes.” Contemporary Educational Psychology 59: 101784. 10.1016/j.cedpsych.2019.101784.

[desc13594-bib-0076] Sroufe, L. A. , B. Coffino , and E. A. Carlson . 2010. “Conceptualizing the Role of Early Experience: Lessons From the Minnesota Longitudinal Study.” Developmental Review 30, no. 1: 36–51. 10.1016/j.dr.2009.12.002. 20419077 PMC2857405

[desc13594-bib-0077] Steinberg, L. 1990. “Autonomy, Conflict, and Harmony in the family relationship.” In At the Threshold: The Developing Adolescent, edited by S. S. Feldman and G. R. Elliot , 255–276. Cambridge, MA: Harvard University Press.

[desc13594-bib-0078] Tamis‐LeMonda, C. S. , Y. Kuchirko , and L. Song . 2014. “Why Is Infant Language Learning Facilitated by Parental Responsiveness?” Current Directions in Psychological Science 23, no. 2: 121–126. 10.1177/0963721414522813.

[desc13594-bib-0079] Thompson, A. , and N. Steinbeis . 2020. “Sensitive Periods in Executive Function Development.” Current Opinion in Behavioral Sciences 36: 98–105. 10.1016/j.cobeha.2020.08.001. 33457470 PMC7789036

[desc13594-bib-0080] Tottenham, N. 2013. “Importance of Early Experiences for Neuro‐Affective Development.” In The Neurobiology of Childhood. Current Topics in Behavioral Neurosciences, edited by S. Andersen , D. Pine (eds), vol 16. Springer, Berlin, Heidelberg. https://doi-org.libproxy.temple.edu/10.1007/7854_2013_254. 10.1007/7854_2013_254 PMC4021037 24264369

[desc13594-bib-0081] Vallotton, C. D. , A. Mastergeorge , T. Foster , K. B. Decker , and C. Ayoub . 2017. “Parenting Supports for Early Vocabulary Development: Specific Effects of Sensitivity and Stimulation Through Infancy.” Infancy 22, no. 1: 78–107. 10.1111/infa.12147. 28111526 PMC5240633

[desc13594-bib-0082] Vandell, D. L. , J. Belsky , M. Burchinal , L. Steinberg , and N. Vandergrift , and NICHD Early Child Care Research Network . 2010. “Do Effects of Early Child Care Extend to Age 15 Years? Results From the NICHD Study of Early Child Care and Youth Development.” Child Development 81, no. 3: 737–756. 10.1111/j.1467-8624.2010.01431.x. 20573102 PMC2938040

[desc13594-bib-0083] Vandell, D. L. , and Z. Gülseven . 2023. “The Study of Early Child Care and Youth Development (SECCYD): Studying Development From Infancy to Adulthood.” Annual Review of Developmental Psychology 5, no. 1: 331–354. 10.1146/annurev-devpsych-120621-035345.

[desc13594-bib-0089] Vygotsky, L. V. 1978. Mind in Society: The Development of Higher Mental Processes. Cambridge, MA: Harvard University Press.

[desc13594-bib-0084] Vygotsky, L. S. 1987. “Thinking and Speech.” In The Collected Works of L.S. Vygotsky, edited by R. W. Rieber and A. S. Carton , Vol. 1, Ser. Problems of General Psychology, 39–285. New York, NY: Plenum Press.

[desc13594-bib-0085] Wade, M. , J. M. Jenkins , V. P. Venkadasalam , N. Binnoon‐Erez , and P. A. Ganea . 2018. “The Role of Maternal Responsiveness and Linguistic Input in Pre‐Academic Skill Development: A Longitudinal Analysis of Pathways.” Cognitive Development 45: 125–140. 10.1016/j.cogdev.2018.01.005.

[desc13594-bib-0086] Woodcock, R. W. , and M. B. Johnson . 1989. Woodcock‐Johnson Psycho‐Educational Battery‐Revised. Allen, TX: DLM Teaching Resources.

[desc13594-bib-0087] Zelazo, P. D. , U. Muller , D. Frye , et al. 2003. “The Development of Executive Function in Childhood: I. The Development of Executive Function.” Monographs of the Society for Research in Child Development 68, no. 3: 11–27. 10.1111/j.0037-976X.2003.00261.x. 14723273

[desc13594-bib-0088] Zimmerman, I. L. , V. G. Steiner , and R. E. Pond . 1992. PLS‐3: Preschool Language Scale‐3. San Antonio, TX: The Psychological Association.

