# Supplementary material for: On the Broader Significance of Maternal Sensitivity: Mothers’ Early and Later Sensitive Parenting Matter to Children's Language, Executive Function, Academics, and Self‐Reliance
Source: Dev Sci. 2024 Dec 16;28(1):e13594. doi: 10.1111/desc.13594 (PMC11647561; doi:10.1111/desc.13594)
Supplement: Supplementary file 1 — Supporting information [file DESC-28-e13594-s005.docx]

**Supplemental Materials (S1)**

**Maternal Sensitivity: Mother-Child Interaction Tasks**

***Procedures***

Trained observers videotaped mother-child interactions during 15-20 min semi-structured sessions when the child was 6, 15, 24, 36, and 54 months of age, in Grades 1, 3, and 5 (approximately 6, 8, and 10 years of age), and at age 15.

At the 6 months assessment, mothers played with their infant for 7-8 min with toys of their choosing and then for 7-8 min with toys provided by the observer. At 15 and 24 months, mothers showed their infant age-appropriate toys and games in three containers in a set order. At 36 months, mothers and children played with toys and games contained in three separate boxes. Activities were chosen because they would be interesting to children based on age, fostered different types of activities, and had the potential to be used differently by the children and their mothers.

At 54 months, mothers and their children participated in three tasks. The first two tasks were designed to be too difficult for 54-month-old children to complete independently, requiring the mothers’ instruction and assistance. Activities included completing a maze using an Etch-A-Sketch, building towers of blocks of various shapes and sizes, and playing with six hand puppets together.

In grade 1, mothers and children participated in three activities requiring coordination and planning. The first activity involved the mother and child operating an Etch-A-Sketch together to draw a picture of a house and a tree on the screen. The second activity required the child to use colored shapes to fill in three geometric cutout frames, a task expected to be too difficult for most first graders to carry out independently. The third activity was an interactive card game in which mothers and children competed to win cards.

In grade 3, mothers and children participated in a rules discussion task and a problem-solving task. In the discussion task, the mother and child selected a card to discuss from three sets of cards containing kid rules, parent rules, and difficult decisions. The problem-solving task required the mother and child to plan how they would accomplish 11 errands in a trip around a town.

In grade 5, the mother and child participated in a family issues discussion task and a shared problem-solving activity. In the discussion task, mother and child selected topics from a set of 22 cards related to typical family issues and then discussed their perspectives. In the problem-solving task, mother and child attempted to construct an egg bungee jump using a set of construction materials.

At the 15-year assessment, the adolescent selected two topics from a list (e.g., chores, homework, money) of topics parents and adolescents often disagree about. Mothers and adolescents discussed these areas for up to 8 minutes (minimum of 5 min).
