# Supplementary material for: On the Broader Significance of Maternal Sensitivity: Mothers’ Early and Later Sensitive Parenting Matter to Children's Language, Executive Function, Academics, and Self‐Reliance
Source: Dev Sci. 2024 Dec 16;28(1):e13594. doi: 10.1111/desc.13594 (PMC11647561; doi:10.1111/desc.13594)
Supplement: Supplementary file 3 — Supporting information [file DESC-28-e13594-s003.pdf]

Table S1

Descriptive Statistics for Study Variables

Variable	*n*	*M*	*SD*	Min	Max
MS-Nondistress6m	1272	5.17	1.27	1.75	7.00
MS-Pos Regard6m	1272	4.99	1.18	1.75	7.00
MS-Intrusiveness6m	1272	5.97	1.36	1.75	7.00
MS-Nondistress15m	1240	5.31	1.20	1.75	7.00
MS-Pos Regard15m	1240	4.91	1.16	1.75	7.00
MS-Intrusiveness15m	1240	6.23	1.28	1.75	7.00
MS-Nondistress24m	1172	5.27	1.30	1.75	7.00
MS-Pos Regard24m	1172	4.94	1.22	1.75	7.00
MS-Intrusiveness24m	1172	6.16	1.27	1.75	7.00
MS-SupportiveP36m	1161	5.28	1.32	1.00	7.00
MS-RespectAut36m	1161	5.29	1.10	1.00	7.00
MS-Hostility36m	1161	6.62	.82	1.00	7.00
MS-SupportiveP54m	1040	5.16	1.30	1.00	7.00
MS-RespectAut54m	1040	5.22	1.11	1.00	7.00
MS-Hostility54m	1040	6.57	.89	2.00	7.00
MS-SupportivePG1	1004	5.16	1.39	1.00	7.00
MS-RespectAutG1	1004	5.26	1.16	1.00	7.00
MS-HostilityG1	1004	6.47	.93	2.00	7.00
MS-SupportivePG3	982	4.99	1.07	1.00	7.00
MS-RespectAutG3	982	4.89	1.02	1.00	7.00
MS-HostilityG3	982	6.46	.83	1.00	7.00
MS-SupportivePG5	929	5.11	.94	2.00	7.00
MS-RespectAutG5	929	4.98	.94	2.00	7.00
MS-HostilityG5	929	6.41	.87	2.00	7.00
MS-WRespons15	897	11.39	2.90	3	21
MS-RespectAut15	898	4.05	1.54	1.00	7.00
MS-Hostility15	898	1.50	1.11	1.00	7.00
ReceptiveLang36m	1158	97.85	15.85	62	136

Variable	*n*	*M*	*SD*	Min	Max
ExpressiveLang36m	1130	96.88	14.53	62	138
ReceptiveLang54m	1064	98.34	19.92	50	139
ExpressiveLang54m	1055	100.62	19.95	50	128
Sustained Attention54m	1001	9.13	7.59	0	41.07
Inhibitory Control54m	1002	14.19	21.30	0	154
Working Memory54m	1054	91.74	18.49	17	142
Self-RelianceG1	966	4.66	1.12	1.00	7.00
Self-RelianceG3	971	4.36	.97	1.13	7.00
Self-RelianceG5	955	4.71	.92	1.00	7.00
Self-Reliance15	957	3.44	.92	1.00	7.00
ReadingG3	1011	112.76	14.04	47	147
ReadingG5	991	105.39	12.33	29	151
Reading15	887	107.71	15.72	44	160
MathG1	1023	110.80	17.14	46	163
MathG3	1013	115.05	15.00	30	153
MathG5	993	109.31	13.54	37	156
Math15	887	102.92	14.22	48	168
Child Gender	1364	1.48	.50	1	2
Child Ethnicity	1364	3.87	.51	1	5
Inc-Needs-Ratio1m	1273	2.86	2.61	0.08	25.08
Maternal Education	1363	14.23	2.51	7	21
Maternal Depression	1363	11.36	9.02	0	53
Child Temperment6m	1279	3.18	.40	1.54	4.72
Child Intelligence15m	1180	108.58	14.01	63	150

Note: MS = Maternal Sensitivity; Pos Regard = Positive Regard; SupportiveP = Supportive Presence; RespectAut = Respect for Autonomy; WResponsiveness = Warm Responsiveness; ReceptiveLang = Receptive Language; ExpressiveLang = Expressive Language; Inc-Needs-Ratio = Income-to-Needs Ratio.
