# Supplementary material for: On the Broader Significance of Maternal Sensitivity: Mothers’ Early and Later Sensitive Parenting Matter to Children's Language, Executive Function, Academics, and Self‐Reliance
Source: Dev Sci. 2024 Dec 16;28(1):e13594. doi: 10.1111/desc.13594 (PMC11647561; doi:10.1111/desc.13594)
Supplement: Supplementary file 4 — Supporting information [file DESC-28-e13594-s004.pdf]

Table S2

Correlations for Study Variables

	Variable	1	2	3	4	5	6	7	8	9	10	11	12	13	14	15	16	17
1.	MS Nondistress6m	—																
2.	MS Pos Regard6m	**.56**	—															
3.	MS Intrusiveness6m	**.67**	**.28**	—														
4.	MS Nondistress15m	**.33**	**.28**	**.24**	—													
5.	MS Pos Regard15m	**.26**	**.29**	**.13**	**.48**	—												
6.	MS Intrusiveness15m	**.29**	**.14**	**.33**	**.57**	**.28**	—											
7.	MS Nondistress24m	**.26**	**.17**	**.19**	**.30**	**.25**	**.21**	—										
8.	MS Pos Regard24m	**.22**	**.23**	**.13**	**.28**	**.32**	**.14**	**.61**	—									
9.	MS Intrusiveness24m	**.23**	**.12**	**.22**	**.30**	**.17**	**.34**	**.54**	**.30**	—								
10.	MS SupportiveP36m	**.36**	**.34**	**.26**	**.37**	**.32**	**.25**	**.41**	**.37**	**.29**	—							
11.	MS RespectAut36m	**.34**	**.27**	**.28**	**.31**	**.23**	**.30**	**.38**	**.28**	**.37**	**.68**	—						
12.	MS Hostility36m	**.28**	**.25**	**.22**	**.23**	**.21**	**.21**	**.32**	**.25**	**.31**	**.55**	**.54**	—					
13.	MS SupportiveP54m	**.32**	**.30**	**.23**	**.30**	**.26**	**.19**	**.39**	**.34**	**.25**	**.48**	**.41**	**.35**	—				
14.	MS RespectAut54m	**.30**	**.22**	**.24**	**.29**	**.27**	**.26**	**.36**	**.28**	**.33**	**.41**	**.44**	**.31**	**.72**	—			
15.	MS Hostility54m	**.23**	**.16**	**.19**	**.21**	**.17**	**.19**	**.30**	**.22**	**.33**	**.34**	**.36**	**.36**	**.61**	**.64**	—		
16.	MS SupportivePG1	**.32**	**.29**	**.21**	**.36**	**.32**	**.27**	**.37**	**.32**	**.26**	**.45**	**.42**	**.36**	**.49**	**.39**	**.35**	—	
17.	MS RespectAutG1	**.31**	**.22**	**.26**	**.32**	**.23**	**.31**	**.36**	**.25**	**.34**	**.39**	**.41**	**.34**	**.40**	**.37**	**.34**	**.75**	—
18.	MS HostilityG1	**.20**	**.16**	**.15**	**.18**	**.13**	**.17**	**.25**	**.18**	**.26**	**.30**	**.28**	**.36**	**.32**	**.26**	**.38**	**.57**	**.60**
19.	MS SupportivePG3	**.32**	**.25**	**.23**	**.35**	**.30**	**.26**	**.30**	**.22**	**.25**	**.38**	**.38**	**.26**	**.39**	**.34**	**.29**	**.44**	**.45**
20.	MS RespectAutG3	**.30**	**.21**	**.24**	**.32**	**.23**	**.28**	**.28**	**.17**	**.25**	**.30**	**.33**	**.23**	**.31**	**.29**	**.28**	**.34**	**.39**
21.	MS HostilityG3	**.14**	.06	**.12**	**.14**	**.15**	**.13**	**.18**	**.10**	**.18**	**.14**	**.17**	**.15**	**.20**	**.20**	**.26**	**.22**	**.27**
22.	MS SupportivePG5	**.28**	**.22**	**.21**	**.31**	**.24**	**.25**	**.31**	**.20**	**.28**	**.37**	**.37**	**.24**	**.35**	**.35**	**.29**	**.41**	**.38**
23.	MS RespectAutG5	**.27**	**.18**	**.21**	**.29**	**.18**	**.27**	**.24**	**.17**	**.26**	**.34**	**.37**	**.21**	**.30**	**.34**	**.26**	**.32**	**.35**
24.	MS HostilityG5	**.19**	**.13**	**.12**	**.17**	**.12**	**.18**	**.25**	**.13**	**.26**	**.23**	**.26**	**.24**	**.20**	**.21**	**.27**	**.30**	**.31**
25.	MS WRespons15	**.19**	**.19**	**.13**	**.17**	**.17**	**.09**	**.15**	**.15**	**.13**	**.28**	**.28**	**.21**	**28**	**.28**	**.18**	**.30**	**.25**
26.	MS RespectAut15	**.19**	**.11**	**.17**	**.16**	**.15**	**.16**	**.18**	**.17**	**.20**	**.26**	**.29**	**.20**	**.23**	**.26**	**.20**	**.27**	**.29**

	Variable	1	2	3	4	5	6	7	8	9	10	11	12	13	14	15	16	17
27.	MS Hostility15	**.18**	**.10**	**.14**	**.16**	**.14**	**.16**	**.16**	**.15**	**.18**	**.26**	**.28**	**.22**	**.23**	**.27**	**.21**	**.28**	**.29**
28.	ReceptiveLang36m	**.27**	**.22**	**.23**	**.35**	**.22**	**.26**	**.38**	**.25**	**.33**	**.41**	**.41**	**.26**	**.34**	**.33**	**.23**	**.35**	**.33**
29.	ExpressiveLang36m	**.20**	**.13**	**.17**	**.24**	**.12**	**.13**	**.27**	**.19**	**.22**	**.28**	**.26**	**.16**	**.22**	**.21**	**.13**	**.21**	**.23**
30.	ReceptiveLang54m	**.26**	**.21**	**.22**	**.34**	**.22**	**.26**	**.33**	**.25**	**.29**	**.40**	**.39**	**.27**	**.34**	**.35**	**.25**	**.38**	**.33**
31.	ExpressiveLang54m	**.25**	**.21**	**.19**	**.31**	**.21**	**.24**	**.34**	**.24**	**.28**	**.39**	**.37**	**.25**	**.35**	**.31**	**.24**	**.34**	**.33**
32.	Sustained Attention54m	**.16**	**.12**	**.11**	**.11**	.06	**.11**	**.18**	**.13**	**.14**	**.17**	**.18**	**.11**	**.26**	**.25**	**.18**	**.15**	**.15**
33.	Inhibitory Control54m	**.12**	.05	**.07**	**.13**	**.08**	**.11**	**.18**	**.12**	**.18**	**.18**	**.21**	**.19**	**.18**	**.23**	**.21**	**.18**	**.18**
34.	Working Memory54m	**.19**	**.15**	**.15**	**.25**	**.14**	**.15**	**.23**	**.14**	**.15**	**.24**	**.28**	**.16**	**.20**	**.21**	**.14**	**.23**	**.22**
35.	Self-RelianceG1	**.13**	**.12**	**.12**	**.12**	.05	**.12**	**.11**	**.08**	**.07**	**.10**	**.14**	**.16**	**.15**	**.12**	**.11**	**.14**	**.15**
36.	Self-RelianceG3	**.12**	**.09**	**.10**	**.16**	**.13**	**.15**	**.11**	**.11**	**.10**	**.15**	**.17**	**.15**	**.20**	**.15**	**.15**	**.13**	**.20**
37.	Self-RelianceG5	**.15**	**.15**	**.11**	**.22**	**.17**	**.17**	**.16**	**.15**	**.12**	**.24**	**.17**	**.10**	**.21**	**.16**	**.13**	**.18**	**.19**
38.	Self-Reliance15	**.07**	.06	.05	**.07**	.06	**.10**	.06	**.09**	.04	**.08**	**.09**	.04	**.07**	.03	.04	**.13**	**.11**
39.	ReadingG3	**.22**	**.20**	**.17**	**.25**	**.16**	**.20**	**.28**	**.21**	**.22**	**.27**	**.28**	**.22**	**.26**	**.27**	**.20**	**.27**	**.29**
40.	ReadingG5	**.23**	**.17**	**.22**	**.22**	**.15**	**.19**	**.26**	**.17**	**.21**	**.26**	**.27**	**.18**	**.24**	**.26**	**.19**	**.25**	**.28**
41.	Reading15	**.27**	**.23**	**.23**	**.28**	**.16**	**.25**	**.26**	**.21**	**.23**	**.33**	**.32**	**.24**	**.31**	**.30**	**.22**	**.29**	**.32**
42.	MathG1	**.23**	**.17**	**.16**	**.24**	**.15**	**.19**	**.26**	**.20**	**.16**	**.32**	**.31**	**.25**	**.30**	**.28**	**.22**	**.29**	**.27**
43.	MathG3	**.18**	**.17**	**.16**	**.21**	**.13**	**.22**	**.23**	**.21**	**.18**	**.28**	**.29**	**.23**	**.27**	**.25**	**.19**	**.28**	**.31**
44.	MathG5	**.18**	**.11**	**.20**	**.24**	**.13**	**.27**	**.26**	**.18**	**.21**	**.26**	**.29**	**.25**	**.29**	**.30**	**.22**	**.29**	**.29**
45.	Math15	**.24**	**.21**	**.23**	**.24**	**.12**	**.25**	**.22**	**.17**	**.16**	**.30**	**.30**	**.20**	**.30**	**.28**	**.20**	**.27**	**.29**
46.	Child Gender	.03	.01	.05	.05	.03	.05	.05	.05	**.14**	**.07**	**.10**	.05	-.02	.04	.01	**-.08**	**.01**
47.	Child Ethnicity	**.14**	**.09**	**.13**	**.19**	**.11**	**.22**	**.15**	**.10**	**.13**	**.15**	**.15**	**.11**	**.14**	**.19**	**.12**	**.23**	**.23**
48.	Inc/Needs Ratio1m	**.26**	**.21**	**.22**	**.23**	**.18**	**.15**	**.21**	**.19**	**.15**	**.30**	**.25**	**.18**	**.21**	**.21**	**.12**	**.24**	**.18**
49.	Maternal Education	**.38**	**.31**	**.29**	**.34**	**.25**	**.26**	**.31**	**.27**	**.26**	**.39**	**.36**	**.27**	**.35**	**.32**	**.25**	**.39**	**.35**
50.	Maternal Depression	**-.17**	**-.08**	**-.12**	**-.18**	**-.12**	**-.13**	**-.17**	**-.14**	**-.17**	**-.17**	**-.20**	**-.14**	**-.14**	**-.13**	**-.13**	**-.15**	**-.15**
51.	Child Temperment6m	**-.14**	**-.07**	**-.11**	**-.14**	**-.09**	**-.09**	**-.14**	**-.10**	**-.12**	**-.14**	**-.12**	**-.10**	**-.14**	**-.11**	**-.05**	**-.16**	**-.13**
52.	Child Intelligence15m	**.15**	**.10**	**.10**	**.23**	**.12**	**.14**	**.23**	**.16**	**.16**	**.17**	**.17**	**.09**	**.15**	**.16**	**.09**	**.14**	**.16**

	Variable	18	19	20	21	22	23	24	25	26	27	28	29	30	31	32	33	34
18.	MS HostilityG1	—																
19.	MS SupportivePG3	**.32**	—															
20.	MS RespectAutG3	**.27**	**.77**	—														
21.	MS HostilityG3	**.25**	**.48**	**.47**	—													
22.	MS SupportivePG5	**.27**	**.42**	**.38**	**.27**	—												
23.	MS RespectAutG5	**.24**	**.43**	**.39**	**.24**	**.78**	—											
24.	MS HostilityG5	**.31**	**.32**	**.31**	**.32**	**.65**	**.55**	—										
25.	MS WRespons15	**.17**	**.30**	**.26**	**.13**	**.39**	**.31**	**.22**	—									
26.	MS RespectAut15	**.22**	**.31**	**.28**	**.19**	**.35**	**.30**	**.28**	**.59**	—								
27.	MS Hostility15	**.22**	**.28**	**.25**	**.23**	**.31**	**.28**	**.25**	**.54**	**.82**	—							
28.	ReceptiveLang36m	**.16**	**.33**	**.35**	**.15**	**.36**	**.34**	**.22**	**.21**	**.19**	**.15**	—						
29.	ExpressiveLang36m	**.10**	**.24**	**.21**	**.08**	**.22**	**.20**	**.14**	**.16**	**.13**	**.09**	**.57**	—					
30.	ReceptiveLang54m	**.20**	**.36**	**.33**	**.13**	**.36**	**.32**	**.19**	**.20**	**.19**	**.13**	**.73**	**.46**	—				
31.	ExpressiveLang54m	**.19**	**.34**	**.32**	**.11**	**.37**	**.35**	**.19**	**.22**	**.15**	**.10**	**.69**	**.49**	**.70**	—			
32.	Sustained Attention54m	**.10**	**.18**	**.16**	**.12**	**.15**	**.14**	**.10**	**.12**	**.11**	**.10**	**.35**	**.19**	**.30**	**.31**	—		
33.	Inhibitory Control54m	**.14**	**.19**	**.20**	**.17**	**.20**	**.18**	**.16**	**.12**	**.16**	**.13**	**.32**	**.13**	**.33**	**.32**	**.24**	—	
34.	Working Memory54m	**.13**	**.19**	**.21**	**.12**	**.23**	**.23**	**.13**	**.16**	**.11**	**.09**	**.55**	**.35**	**.51**	**.53**	**.24**	**.20**	—
35.	Self-RelianceG1	**.06**	**.20**	**.20**	**.15**	**.13**	**.11**	**.08**	.07	.04	.05	**.21**	**.12**	**.20**	**.19**	**.20**	**.14**	**.14**
36.	Self-RelianceG3	**.11**	**.22**	**.24**	**.21**	**.19**	**.21**	**.10**	**.16**	**.16**	**.11**	**.21**	**.19**	**.22**	**.25**	**.13**	**.15**	**.15**
37.	Self-RelianceG5	**.13**	**.18**	**.20**	**.08**	**.19**	**.19**	**.08**	**.13**	**.08**	.05	**.24**	**.19**	**.27**	**.29**	**.17**	**.17**	**.16**
38.	Self-Reliance15	.07	**.18**	**.16**	**.15**	**.11**	**.16**	**.07**	.06	**.08**	**.09**	**.19**	**.13**	**.18**	**.24**	**.12**	**.08**	**.17**
39.	ReadingG3	**.17**	**.33**	**.33**	**.10**	**.30**	**.27**	**.17**	**.16**	**.19**	**.12**	**.50**	**.35**	**.52**	**.51**	**.23**	**.24**	**.45**
40.	ReadingG5	**.17**	**.30**	**.28**	**.09**	**.29**	**.28**	**.17**	**.18**	**.19**	**.13**	**.38**	**.38**	**.50**	**.48**	**.20**	**.18**	**.42**
41.	Reading15	**.21**	**.32**	**.30**	**.08**	**.32**	**.30**	**.20**	**.23**	**.17**	**.14**	**.49**	**.35**	**.50**	**.50**	**.21**	**.22**	**.43**
42.	MathG1	**.15**	**.25**	**.25**	.06	**.27**	**.25**	**.14**	**.17**	**.15**	**.11**	**.56**	**.35**	**.56**	**.53**	**.30**	**.26**	**.45**

	Variable	18	19	20	21	22	23	24	25	26	27	28	29	30	31	32	33	34
43.	MathG3	**.17**	**.28**	**.25**	**.08**	**.25**	**.24**	**.15**	**.15**	**.16**	**.09**	**.50**	**.34**	**.49**	**.50**	**.24**	**.21**	**.40**
44.	MathG5	**.19**	**.28**	**.27**	**.10**	**.29**	**.28**	**.18**	**.17**	**.18**	**.14**	**.47**	**.28**	**.51**	**.49**	**.21**	**.19**	**.39**
45.	Math15	**.17**	**.28**	**.28**	**.09**	**.29**	**.30**	**.21**	**.20**	**.18**	**.13**	**.42**	**.29**	**.47**	**.41**	**.20**	**.17**	**.34**
46.	Child Gender	**.07**	**.10**	**.13**	**.09**	**.10**	**.13**	.04	.02	.05	.05	**.20**	**.16**	**.16**	**.13**	**.07**	**.24**	.06
47.	Child Ethnicity	**.14**	**.11**	**.09**	.02	**.19**	**.17**	**.11**	**.08**	**.07**	**.04**	**.21**	**.12**	**.21**	**.22**	**.11**	**.14**	**.13**
48.	Inc/Needs Ratio1m	**.11**	**.26**	**.25**	**.10**	**.22**	**.21**	**.13**	**.17**	**.16**	**.15**	**.31**	**.17**	**.31**	**.26**	**.10**	**.11**	**.20**
49.	Maternal Education	**.23**	**.38**	**.36**	**.17**	**.32**	**.33**	**.29**	**.25**	**.27**	**.22**	**.44**	**.31**	**.43**	**.41**	**.18**	**.21**	**.28**
50.	Maternal Depression	**-.13**	**-.18**	**-.16**	**-.12**	**-.20**	**-.19**	**-.13**	**-.10**	**-.12**	**-.10**	**-.18**	**-.12**	**-.19**	**-.15**	**-.14**	**-.09**	**-.17**
51.	Child Temperment6m	-.03	**-.11**	**-.11**	**-.07**	**-.07**	-.03	-.02	**-.12**	-.05	**-.08**	**-.14**	**-.09**	**-.13**	**-.15**	-.06	**-.06**	**-.07**
52.	Child Intelligence15m	.04	**.16**	**.16**	**.10**	**.21**	**.17**	**.10**	**.13**	**.07**	.04	**.44**	**.29**	**.35**	**.37**	**.20**	**.20**	**.33**

	Variable	35	36	37	38	39	40	41	42	43	44	45	46	47	48	49	50
35.	Self-RelianceG1	—															
36.	Self-RelianceG3	**.29**	—														
37.	Self-RelianceG5	**.22**	**.35**	—													
38.	Self-Reliance15	**.14**	**.16**	**.09**	—												
39.	ReadingG3	**.15**	**.28**	**.25**	**.17**	—											
40.	ReadingG5	**.10**	**.25**	**.29**	**.23**	**.75**	—										
41.	Reading15	**.23**	**.28**	**.26**	**.18**	**.66**	**.67**	—									
42.	MathG1	**.23**	**.28**	**.26**	**.18**	**.56**	**.53**	**.57**	—								
43.	MathG3	**.20**	**.29**	**.31**	**.17**	**.65**	**.58**	**.55**	**.70**	—							
44.	MathG5	**.12**	**.31**	**.31**	**.20**	**.61**	**.61**	**.60**	**.70**	**.76**	—						
45.	Math15	**.18**	**.34**	**.32**	**.15**	**.54**	**.54**	**.67**	**.64**	**.65**	**.72**	—					
46.	Child Gender	**.12**	**.14**	**.12**	**.15**	**.10**	**.07**	-.05	-.02	.02	-.02	**-.09**	—				
47.	Child Ethnicity	.00	**.09**	**.09**	.02	**.15**	**.15**	**.16**	**.17**	**.17**	**.18**	**.13**	.01	—			
48.	Inc/Needs Ratio1m	**.11**	**.14**	**.16**	**.10**	**.23**	**.22**	**.23**	**.20**	**.23**	**.24**	**.23**	.01	**.15**	—		
49.	Maternal Education	**.18**	**.23**	**.23**	**.13**	**.36**	**.36**	**.35**	**.33**	**.37**	**.36**	**.39**	.01	**.07**	**.42**	—	
50.	Maternal Depression	**-.09**	**-.07**	**-.10**	**-.08**	**-.13**	**-.17**	**-.16**	**-.14**	**-.18**	**-.17**	**-.14**	-.04	**-.06**	**-.17**	**-.23**	—
51.	Child Temperment6m	-.02	-.02	**-.10**	**-.07**	**-.13**	**-.13**	**-.11**	**-.15**	**-.16**	**-.18**	**-.15**	.05	**-.14**	**-.14**	**-.14**	**-.23**
52.	Child Intelligence15m	**.13**	**.13**	**.12**	**.11**	**.23**	**.30**	**.35**	**.27**	**.27**	**.27**	**.22**	**.13**	**.16**	**.11**	**.13**	**-.08**

	Variable	51	52
51.	Child Tempermemt6m	—	
52.	Child Intelligence15m	-.06	—

Note: MS = Maternal Sensitivity; Pos Regard = Positive Regard; SupportiveP = Supportive Presence; RespectAut = Respect for Autonomy; WResponsiveness = Warm Responsiveness; ReceptiveLang = Receptive Language; ExpressiveLang = Expressive Language; Inc/Needs Ratio = Income to Needs Ratio.

All correlation coefficients shown in bold text are significant at $p < .05$. Correlation coefficients not shown in bold text are not significant.
