# Supplementary material for: On the Broader Significance of Maternal Sensitivity: Mothers’ Early and Later Sensitive Parenting Matter to Children's Language, Executive Function, Academics, and Self‐Reliance
Source: Dev Sci. 2024 Dec 16;28(1):e13594. doi: 10.1111/desc.13594 (PMC11647561; doi:10.1111/desc.13594)
Supplement: Supplementary file 5 — Supporting information [file DESC-28-e13594-s007.pdf]

Mplus VERSION 8.10
MUTHEN & MUTHEN
10/30/2024   5:49 PM

INPUT INSTRUCTIONS

```
  TITLE: Foley & Weinraub
  DATA: FILE IS 'matsens_DS_file_mplus.csv';
  VARIABLE: NAMES ARE ID CSEX_M01 CRACEM01 MEDUCM01 TEMP_M06 MDI15O15 RAWVPM24
   STDSCM36 SPTSIO36 RSCSIO36 HOSSIO36 RVCSTM36 RELSTM36 AVBTCO36 TOTBCO36
   PLSASC54 PLSESC54 WJMSSC54 CPINCC54 CPOMSC54 SLFR_O1S WJLWSC1S WJAPSC1S
   WJBRSCG3 WJBMSCG3 SLFR_OG3 SLFR_OG5 WJBRSCG5 WJBMSCG5 WJPCSCX5 WJAPSCX5
   SLFRLCX5 SPTSIO54 RSCSIO54 HOSSIO54 SPTSIO1S RSCSIO1S HOSSIO1S SPTSIOG3
   RSCSIOG3 HOSSIOG3 SPTSIOG5 RSCSIOG5 HOSSIOG5 MRASIOX5 MWRSIOX5 MHISIOX5
   VCRAWM15 VPRAWM15 Nondistress24m_7trans Posregard24m_7trans nondistress6m_7trans
   Posregard6m_7trans intrusiveness6m_reversed_7trans Nondistress15m_7trans
   Posregard15m_7trans intrusiveness15m_reversed_7trans Hostility36m_reversed
   Hostility54m_reversed HostilityG1_reversed HostilityG3_reversed
   HostilityG5_reversed Intrusiveness_REVER_Trans24m WJPCSCG3 WJAPSCG3
   WJPCSCG5 WJAPSCG5 INCNTM01 MADEPM01;
        USEVARIABLES ARE nondistress6m_7trans Posregard6m_7trans
   intrusiveness6m_reversed_7trans Nondistress15m_7trans Posregard15m_7trans
   intrusiveness15m_reversed_7trans Nondistress24m_7trans Posregard24m_7trans
   Intrusiveness_REVER_Trans24m SPTSIO36 RSCSIO36 Hostility36m_reversed
   SPTSIO54 RSCSIO54 Hostility54m_reversed SPTSIO1S RSCSIO1S HostilityG1_reversed
   SPTSIOG3 RSCSIOG3 HostilityG3_reversed SPTSIOG5 RSCSIOG5 HostilityG5_reversed
   MRASIOX5 MWRSIOX5 MHISIOX5 SLFR_O1S SLFR_OG3 SLFR_OG5 SLFRLCX5 WJAPSC1S
   WJAPSCG3 WJAPSCG5 WJAPSCX5 WJPCSCG3 WJPCSCG5 WJPCSCX5 RVCSTM36 RELSTM36
   PLSASC54 PLSESC54 CPINCC54 CPOMSC54 WJMSSC54 CSEX_M01 CRACEM01 MEDUCM01
   TEMP_M06 MDI15O15 INCNTM01 MADEPM01;
        MISSING IS .;
        USEOBSERVATIONS ARE (CSEX_M01 == 1 OR CSEX_M01 == 2);
  ANALYSIS: ESTIMATOR IS MLR;
        MODEL IS NOCOVARIANCES;
  MODEL:
  MS_6m BY nondistress6m_7trans
        Posregard6m_7trans !(a)
        intrusiveness6m_reversed_7trans; ! (b);
              MS_15m BY Nondistress15m_7trans
                    Posregard15m_7trans !(a)
                 intrusiveness15m_reversed_7trans; ! (b);
              MS_24m BY Nondistress24m_7trans
                    Posregard24m_7trans! (a)
                 Intrusiveness_REVER_Trans24m; ! (b);
              MS_36m BY SPTSIO36
                     RSCSIO36! (a)
                     Hostility36m_reversed; ! (b);
              MS_54m BY SPTSIO54
                     RSCSIO54! (a)
                     Hostility54m_reversed; ! (b);
              MS_G1 BY SPTSIO1S
                    RSCSIO1S! (a)
                    HostilityG1_reversed; ! (b);
```

```
 MS_G3 BY SPTSIOG3
        RSCSIOG3! (a)
        HostilityG3_reversed; ! (b);
 MS_G5 BY SPTSIOG5
        RSCSIOG5! (a)
        HostilityG5_reversed; ! (b);
 MS_15 BY MRASIOX5
        MWRSIOX5! (a)
        MHISIOX5; ! (b);

 MS_RI BY MS_6m@1 MS_15m@1 MS_24m@1 MS_36m@1 MS_54m@1 MS_G1@1
     MS_G3@1 MS_G5@1 MS_15@1;

 MS_6m@0; MS_15m@0; MS_24m@0; MS_36m@0; MS_54m@0; MS_G1@0;
 MS_G3@0; MS_G5@0; MS_15@0;;

 MS_6mr BY MS_6m@1;
MS_15mr BY MS_15m@1;
 MS_24mr BY MS_24m@1;
 MS_36mr BY MS_36m@1;
 MS_54mr BY MS_54m@1;
 MS_G1r BY MS_G1@1;
 MS_G3r BY MS_G3@1;
 MS_G5r BY MS_G5@1;
 MS_15r BY MS_15@1;

sr BY SLFR_O1S@1 SLFR_OG3@1 SLFR_OG5@1 SLFRLCX5@1;

SLFR_O1S@0; SLFR_OG3@0; SLFR_OG5@0; SLFRLCX5@0

slfr_g1r BY SLFR_O1S@1;
slfr_g3r BY SLFR_OG3@1;
slfr_g5r BY SLFR_OG5@1;
slfr_15r BY SLFRLCX5@1;

  read BY WJPCSCG3@1 WJPCSCG5@1 WJPCSCX5@1;

  WJPCSCG3@0; WJPCSCG5@0; WJPCSCX5@0;

  WJPCSCG3_r BY WJPCSCG3@1;
  WJPCSCG5_r BY WJPCSCG5@1;
  WJPCSCX5_r BY WJPCSCX5@1;

  math BY WJAPSC1S@1 WJAPSCG3@1 WJAPSCG5@1 WJAPSCX5@1;


  WJAPSC1S@0; WJAPSCG3@0; WJAPSCG5@0; WJAPSCX5@0;

  WJAPSC1S_r BY WJAPSC1S@1;
  WJAPSCG3_r BY WJAPSCG3@1;
  WJAPSCG5_r BY WJAPSCG5@1;
  WJAPSCX5_r BY WJAPSCX5@1;

   MS_RI WITH sr math read;
```

```
            sr WITH math read;
            read WITH math;

            MS_RI sr math read ON
              CSEX_M01 CRACEM01 MEDUCM01 TEMP_M06 MDI15O15 INCNTM01 MADEPM01;

            PLSASC54 PLSESC54 CPINCC54 CPOMSC54 WJMSSC54 RVCSTM36 RELSTM36 ON
                CSEX_M01 CRACEM01 MEDUCM01 TEMP_M06 MDI15O15 INCNTM01 MADEPM01;

  ! Lags

  MS_15mr ON MS_6mr;
  MS_24mr ON MS_15mr;
  MS_36mr ON MS_24mr;
  MS_54mr PLSASC54 PLSESC54 CPINCC54 CPOMSC54 WJMSSC54 ON
    MS_36mr RVCSTM36 RELSTM36;
  MS_G1r slfr_g1r WJAPSC1S_r ON
    MS_54mr PLSASC54 PLSESC54 CPINCC54 CPOMSC54 WJMSSC54;
  MS_G3r slfr_g3r WJPCSCG3_r WJAPSCG3_r ON MS_G1r slfr_g1r WJAPSC1S_r;
  MS_G5r slfr_g5r WJPCSCG5_r WJAPSCG5_r ON MS_G3r slfr_g3r WJPCSCG3_r WJAPSCG3_r;
  MS_15r slfr_15r WJPCSCX5_r WJAPSCX5_r ON MS_G5r slfr_g5r WJPCSCG5_r WJAPSCG5_r;

  ! 36 Month Covariates

  RVCSTM36 RELSTM36 ON MS_24mr;

  ! Covariances

  MS_54mr PLSASC54 PLSESC54 CPINCC54 CPOMSC54 WJMSSC54 WITH
    MS_54mr PLSASC54 PLSESC54 CPINCC54 CPOMSC54 WJMSSC54;

  MS_36mr RVCSTM36 RELSTM36 WITH MS_36mr RVCSTM36 RELSTM36;


  CSEX_M01 CRACEM01 MEDUCM01 TEMP_M06 MDI15O15 INCNTM01 MADEPM01 WITH
     CSEX_M01 CRACEM01 MEDUCM01 TEMP_M06 MDI15O15 INCNTM01 MADEPM01;

  OUTPUT: sampstat stdyx modindices(25);



*** WARNING in VARIABLE command
  Note that only the first 8 characters of variable names are used in the output.
  Shorten variable names to avoid any confusion.
   1 WARNING(S) FOUND IN THE INPUT INSTRUCTIONS



Foley & Weinraub

SUMMARY OF ANALYSIS

Number of groups                                 1
Number of observations                           1364
```

Number of dependent variables 45
Number of independent variables 7
Number of continuous latent variables 33

Observed dependent variables

Continuous
NONDISTRES POSREGARD6 INTRUSIVEN NONDISTRES POSREGARD1 INTRUSIVEN
NONDISTRES POSREGARD2 INTRUSIVEN SPTSIO36 RSCSIO36 HOSTILITY3
SPTSIO54 RSCSIO54 HOSTILITY5 SPTSIO1S RSCSIO1S HOSTILITYG
SPTSIOG3 RSCSIOG3 HOSTILITYG SPTSIOG5 RSCSIOG5 HOSTILITYG
MRASIOX5 MWRSIOX5 MHISIOX5 SLFR_O1S SLFR_OG3 SLFR_OG5
SLFRLCX5 WJAPSC1S WJAPSCG3 WJAPSCG5 WJAPSCX5 WJPCSCG3
WJPCSCG5 WJPCSCX5 RVCSTM36 RELSTM36 PLSASC54 PLSESC54
CPINCC54 CPOMSC54 WJMSSC54

Observed independent variables
CSEX_M01 CRACEM01 MEDUCM01 TEMP_M06 MDI15O15 INCNTM01
MADEPM01

Continuous latent variables
MS_6M MS_15M MS_24M MS_36M MS_54M MS_G1
MS_G3 MS_G5 MS_15 MS_RI MS_6MR MS_15MR
MS_24MR MS_36MR MS_54MR MS_G1R MS_G3R MS_G5R
MS_15R SR SLFR_G1R SLFR_G3R SLFR_G5R SLFR_15R
READ WJPCSCG3 WJPCSCG5 WJPCSCX5 MATH WJAPSC1S
WJAPSCG3 WJAPSCG5 WJAPSCX5

Estimator MLR
Information matrix OBSERVED
Maximum number of iterations 1000
Convergence criterion 0.500D-04
Maximum number of steepest descent iterations 20
Maximum number of iterations for H1 2000
Convergence criterion for H1 0.100D-03

Input data file(s)
matsens_DS_file_mplus.csv

Input data format FREE

SUMMARY OF DATA

Number of missing data patterns 422

COVARIANCE COVERAGE OF DATA

Minimum covariance coverage value 0.100

PROPORTION OF DATA PRESENT

Covariance Coverage

	NONDISTR	POSREGAR	INTRUSIV	NONDISTR	POSREGAR
NONDISTR	0.933				
POSREGAR	0.933	0.933			
INTRUSIV	0.933	0.933	0.933		
NONDISTR	0.890	0.890	0.890	0.909	
POSREGAR	0.890	0.890	0.890	0.909	0.909
INTRUSIV	0.890	0.890	0.890	0.909	0.909
NONDISTR	0.840	0.840	0.840	0.848	0.848
POSREGAR	0.840	0.840	0.840	0.848	0.848
INTRUSIV	0.840	0.840	0.840	0.848	0.848
SPTSIO36	0.831	0.831	0.831	0.838	0.838
RSCSIO36	0.831	0.831	0.831	0.838	0.838
HOSTILIT	0.831	0.831	0.831	0.838	0.838
SPTSIO54	0.746	0.746	0.746	0.753	0.753
RSCSIO54	0.746	0.746	0.746	0.753	0.753
HOSTILIT	0.746	0.746	0.746	0.753	0.753
SPTSIO1S	0.724	0.724	0.724	0.726	0.726
RSCSIO1S	0.724	0.724	0.724	0.726	0.726
HOSTILIT	0.724	0.724	0.724	0.726	0.726
SPTSIOG3	0.701	0.701	0.701	0.701	0.701
RSCSIOG3	0.701	0.701	0.701	0.701	0.701
HOSTILIT	0.701	0.701	0.701	0.701	0.701
SPTSIOG5	0.664	0.664	0.664	0.665	0.665
RSCSIOG5	0.664	0.664	0.664	0.665	0.665
HOSTILIT	0.664	0.664	0.664	0.665	0.665
MRASIOX5	0.641	0.641	0.641	0.639	0.639
MWRSIOX5	0.640	0.640	0.640	0.638	0.638
MHISIOX5	0.641	0.641	0.641	0.639	0.639
SLFR_O1S	0.696	0.696	0.696	0.697	0.697
SLFR_OG3	0.695	0.695	0.695	0.695	0.695
SLFR_OG5	0.683	0.683	0.683	0.684	0.684
SLFRLCX5	0.684	0.684	0.684	0.683	0.683
WJAPSC1S	0.736	0.736	0.736	0.738	0.738
WJAPSCG3	0.717	0.717	0.717	0.724	0.724
WJAPSCG5	0.700	0.700	0.700	0.709	0.709
WJAPSCX5	0.635	0.635	0.635	0.635	0.635
WJPCSCG3	0.716	0.716	0.716	0.722	0.722
WJPCSCG5	0.699	0.699	0.699	0.708	0.708
WJPCSCX5	0.636	0.636	0.636	0.636	0.636
RVCSTM36	0.830	0.830	0.830	0.837	0.837
RELSTM36	0.811	0.811	0.811	0.817	0.817
PLSASC54	0.763	0.763	0.763	0.768	0.768
PLSESC54	0.757	0.757	0.757	0.761	0.761
CPINCC54	0.717	0.717	0.717	0.723	0.723
CPOMSC54	0.717	0.717	0.717	0.723	0.723
WJMSSC54	0.754	0.754	0.754	0.761	0.761
CSEX_M01	0.933	0.933	0.933	0.909	0.909
CRACEM01	0.933	0.933	0.933	0.909	0.909
MEDUCM01	0.933	0.933	0.933	0.909	0.909

TEMP_M06	0.932	0.932	0.932	0.895	0.895
MDI15O15	0.850	0.850	0.850	0.860	0.860
INCNTM01	0.837	0.837	0.837	0.849	0.849
MADEPM01	0.896	0.896	0.896	0.908	0.908

Covariance Coverage

	INTRUSIV	NONDISTR	POSREGAR	INTRUSIV	SPTSIO36
INTRUSIV	0.909				
NONDISTR	0.848	0.859			
POSREGAR	0.848	0.859	0.859		
INTRUSIV	0.848	0.859	0.859	0.859	
SPTSIO36	0.838	0.820	0.820	0.820	0.851
RSCSIO36	0.838	0.820	0.820	0.820	0.851
HOSTILIT	0.838	0.820	0.820	0.820	0.851
SPTSIO54	0.753	0.738	0.738	0.738	0.743
RSCSIO54	0.753	0.738	0.738	0.738	0.743
HOSTILIT	0.753	0.738	0.738	0.738	0.743
SPTSIO1S	0.726	0.713	0.713	0.713	0.715
RSCSIO1S	0.726	0.713	0.713	0.713	0.715
HOSTILIT	0.726	0.713	0.713	0.713	0.715
SPTSIOG3	0.701	0.678	0.678	0.678	0.680
RSCSIOG3	0.701	0.678	0.678	0.678	0.680
HOSTILIT	0.701	0.678	0.678	0.678	0.680
SPTSIOG5	0.665	0.642	0.642	0.642	0.641
RSCSIOG5	0.665	0.642	0.642	0.642	0.641
HOSTILIT	0.665	0.642	0.642	0.642	0.641
MRASIOX5	0.639	0.621	0.621	0.621	0.622
MWRSIOX5	0.638	0.620	0.620	0.620	0.621
MHISIOX5	0.639	0.621	0.621	0.621	0.622
SLFR_O1S	0.697	0.680	0.680	0.680	0.685
SLFR_OG3	0.695	0.672	0.672	0.672	0.673
SLFR_OG5	0.684	0.661	0.661	0.661	0.658
SLFRLCX5	0.683	0.662	0.662	0.662	0.661
WJAPSC1S	0.738	0.725	0.725	0.725	0.727
WJAPSCG3	0.724	0.699	0.699	0.699	0.696
WJAPSCG5	0.709	0.682	0.682	0.682	0.680
WJAPSCX5	0.635	0.620	0.620	0.620	0.617
WJPCSCG3	0.722	0.697	0.697	0.697	0.694
WJPCSCG5	0.708	0.681	0.681	0.681	0.680
WJPCSCX5	0.636	0.621	0.621	0.621	0.617
RVCSTM36	0.837	0.820	0.820	0.820	0.842
RELSTM36	0.817	0.801	0.801	0.801	0.822
PLSASC54	0.768	0.751	0.751	0.751	0.755
PLSESC54	0.761	0.745	0.745	0.745	0.749
CPINCC54	0.723	0.710	0.710	0.710	0.716
CPOMSC54	0.723	0.710	0.710	0.710	0.716
WJMSSC54	0.761	0.746	0.746	0.746	0.750
CSEX_M01	0.909	0.859	0.859	0.859	0.851
CRACEM01	0.909	0.859	0.859	0.859	0.851
MEDUCM01	0.909	0.859	0.859	0.859	0.851
TEMP_M06	0.895	0.845	0.845	0.845	0.837
MDI15O15	0.860	0.821	0.821	0.821	0.809

INCNTM01	0.849	0.800	0.800	0.800	0.793
MADEPM01	0.908	0.852	0.852	0.852	0.842

Covariance Coverage

	RSCSIO36	HOSTILIT	SPTSIO54	RSCSIO54	HOSTILIT
RSCSIO36	0.851				
HOSTILIT	0.851	0.851			
SPTSIO54	0.743	0.743	0.762		
RSCSIO54	0.743	0.743	0.762	0.762	
HOSTILIT	0.743	0.743	0.762	0.762	0.762
SPTSIO1S	0.715	0.715	0.705	0.705	0.705
RSCSIO1S	0.715	0.715	0.705	0.705	0.705
HOSTILIT	0.715	0.715	0.705	0.705	0.705
SPTSIOG3	0.680	0.680	0.658	0.658	0.658
RSCSIOG3	0.680	0.680	0.658	0.658	0.658
HOSTILIT	0.680	0.680	0.658	0.658	0.658
SPTSIOG5	0.641	0.641	0.617	0.617	0.617
RSCSIOG5	0.641	0.641	0.617	0.617	0.617
HOSTILIT	0.641	0.641	0.617	0.617	0.617
MRASIOX5	0.622	0.622	0.599	0.599	0.599
MWRSIOX5	0.621	0.621	0.598	0.598	0.598
MHISIOX5	0.622	0.622	0.599	0.599	0.599
SLFR_O1S	0.685	0.685	0.673	0.673	0.673
SLFR_OG3	0.673	0.673	0.651	0.651	0.651
SLFR_OG5	0.658	0.658	0.638	0.638	0.638
SLFRLCX5	0.661	0.661	0.639	0.639	0.639
WJAPSC1S	0.727	0.727	0.713	0.713	0.713
WJAPSCG3	0.696	0.696	0.677	0.677	0.677
WJAPSCG5	0.680	0.680	0.655	0.655	0.655
WJAPSCX5	0.617	0.617	0.603	0.603	0.603
WJPCSCG3	0.694	0.694	0.675	0.675	0.675
WJPCSCG5	0.680	0.680	0.654	0.654	0.654
WJPCSCX5	0.617	0.617	0.603	0.603	0.603
RVCSTM36	0.842	0.842	0.740	0.740	0.740
RELSTM36	0.822	0.822	0.724	0.724	0.724
PLSASC54	0.755	0.755	0.751	0.751	0.751
PLSESC54	0.749	0.749	0.745	0.745	0.745
CPINCC54	0.716	0.716	0.723	0.723	0.723
CPOMSC54	0.716	0.716	0.723	0.723	0.723
WJMSSC54	0.750	0.750	0.757	0.757	0.757
CSEX_M01	0.851	0.851	0.762	0.762	0.762
CRACEM01	0.851	0.851	0.762	0.762	0.762
MEDUCM01	0.851	0.851	0.762	0.762	0.762
TEMP_M06	0.837	0.837	0.750	0.750	0.750
MDI15O15	0.809	0.809	0.728	0.728	0.728
INCNTM01	0.793	0.793	0.715	0.715	0.715
MADEPM01	0.842	0.842	0.757	0.757	0.757

Covariance Coverage

	SPTSIO1S	RSCSIO1S	HOSTILIT	SPTSIOG3	RSCSIOG3

SPTSIO1S	0.736				
RSCSIO1S	0.736	0.736			
HOSTILIT	0.736	0.736	0.736		
SPTSIOG3	0.655	0.655	0.655	0.720	
RSCSIOG3	0.655	0.655	0.655	0.720	0.720
HOSTILIT	0.655	0.655	0.655	0.720	0.720
SPTSIOG5	0.614	0.614	0.614	0.629	0.629
RSCSIOG5	0.614	0.614	0.614	0.629	0.629
HOSTILIT	0.614	0.614	0.614	0.629	0.629
MRASIOX5	0.595	0.595	0.595	0.604	0.604
MWRSIOX5	0.595	0.595	0.595	0.603	0.603
MHISIOX5	0.595	0.595	0.595	0.604	0.604
SLFR_O1S	0.677	0.677	0.677	0.625	0.625
SLFR_OG3	0.645	0.645	0.645	0.652	0.652
SLFR_OG5	0.625	0.625	0.625	0.633	0.633
SLFRLCX5	0.634	0.634	0.634	0.642	0.642
WJAPSC1S	0.732	0.732	0.732	0.666	0.666
WJAPSCG3	0.669	0.669	0.669	0.680	0.680
WJAPSCG5	0.648	0.648	0.648	0.652	0.652
WJAPSCX5	0.595	0.595	0.595	0.600	0.600
WJPCSCG3	0.667	0.667	0.667	0.679	0.679
WJPCSCG5	0.648	0.648	0.648	0.650	0.650
WJPCSCX5	0.596	0.596	0.596	0.600	0.600
RVCSTM36	0.713	0.713	0.713	0.675	0.675
RELSTM36	0.696	0.696	0.696	0.657	0.657
PLSASC54	0.711	0.711	0.711	0.667	0.667
PLSESC54	0.705	0.705	0.705	0.662	0.662
CPINCC54	0.677	0.677	0.677	0.635	0.635
CPOMSC54	0.677	0.677	0.677	0.635	0.635
WJMSSC54	0.708	0.708	0.708	0.663	0.663
CSEX_M01	0.736	0.736	0.736	0.720	0.720
CRACEM01	0.736	0.736	0.736	0.720	0.720
MEDUCM01	0.736	0.736	0.736	0.720	0.720
TEMP_M06	0.727	0.727	0.727	0.705	0.705
MDI15O15	0.703	0.703	0.703	0.674	0.674
INCNTM01	0.688	0.688	0.688	0.666	0.666
MADEPM01	0.730	0.730	0.730	0.705	0.705

Covariance Coverage

	HOSTILIT	SPTSIOG5	RSCSIOG5	HOSTILIT	MRASIOX5
HOSTILIT	0.720				
SPTSIOG5	0.629	0.681			
RSCSIOG5	0.629	0.681	0.681		
HOSTILIT	0.629	0.681	0.681	0.681	
MRASIOX5	0.604	0.587	0.587	0.587	0.658
MWRSIOX5	0.603	0.587	0.587	0.587	0.658
MHISIOX5	0.604	0.587	0.587	0.587	0.658
SLFR_O1S	0.625	0.586	0.586	0.586	0.567
SLFR_OG3	0.652	0.608	0.608	0.608	0.589
SLFR_OG5	0.633	0.617	0.617	0.617	0.595
SLFRLCX5	0.642	0.625	0.625	0.625	0.641
WJAPSC1S	0.666	0.625	0.625	0.625	0.605

WJAPSCG3	0.680	0.632	0.632	0.632	0.603
WJAPSCG5	0.652	0.654	0.654	0.654	0.614
WJAPSCX5	0.600	0.583	0.583	0.583	0.608
WJPCSCG3	0.679	0.630	0.630	0.630	0.602
WJPCSCG5	0.650	0.652	0.652	0.652	0.612
WJPCSCX5	0.600	0.584	0.584	0.584	0.609
RVCSTM36	0.675	0.639	0.639	0.639	0.622
RELSTM36	0.657	0.625	0.625	0.625	0.606
PLSASC54	0.667	0.625	0.625	0.625	0.607
PLSESC54	0.662	0.621	0.621	0.621	0.604
CPINCC54	0.635	0.592	0.592	0.592	0.578
CPOMSC54	0.635	0.592	0.592	0.592	0.578
WJMSSC54	0.663	0.623	0.623	0.623	0.604
CSEX_M01	0.720	0.681	0.681	0.681	0.658
CRACEM01	0.720	0.681	0.681	0.681	0.658
MEDUCM01	0.720	0.681	0.681	0.681	0.658
TEMP_M06	0.705	0.668	0.668	0.668	0.644
MDI15O15	0.674	0.641	0.641	0.641	0.614
INCNTM01	0.666	0.622	0.622	0.622	0.604
MADEPM01	0.705	0.667	0.667	0.667	0.642

Covariance Coverage

	MWRSIOX5	MHISIOX5	SLFR_O1S	SLFR_OG3	SLFR_OG5
MWRSIOX5	0.658				
MHISIOX5	0.658	0.658			
SLFR_O1S	0.566	0.567	0.708		
SLFR_OG3	0.588	0.589	0.641	0.712	
SLFR_OG5	0.594	0.595	0.615	0.643	0.700
SLFRLCX5	0.641	0.641	0.603	0.625	0.634
WJAPSC1S	0.604	0.605	0.689	0.658	0.639
WJAPSCG3	0.602	0.603	0.644	0.665	0.647
WJAPSCG5	0.613	0.614	0.617	0.636	0.646
WJAPSCX5	0.607	0.608	0.565	0.583	0.587
WJPCSCG3	0.601	0.602	0.642	0.663	0.646
WJPCSCG5	0.611	0.612	0.617	0.636	0.644
WJPCSCX5	0.608	0.609	0.565	0.583	0.587
RVCSTM36	0.621	0.622	0.683	0.671	0.655
RELSTM36	0.606	0.606	0.667	0.654	0.640
PLSASC54	0.606	0.607	0.687	0.663	0.648
PLSESC54	0.603	0.604	0.682	0.657	0.643
CPINCC54	0.578	0.578	0.652	0.627	0.612
CPOMSC54	0.578	0.578	0.652	0.627	0.612
WJMSSC54	0.603	0.604	0.681	0.656	0.642
CSEX_M01	0.658	0.658	0.708	0.712	0.700
CRACEM01	0.658	0.658	0.708	0.712	0.700
MEDUCM01	0.658	0.658	0.708	0.712	0.700
TEMP_M06	0.644	0.644	0.699	0.697	0.685
MDI15O15	0.614	0.614	0.672	0.668	0.655
INCNTM01	0.603	0.604	0.662	0.659	0.647
MADEPM01	0.641	0.642	0.702	0.699	0.687

Covariance Coverage

	SLFRLCX5	WJAPSC1S	WJAPSCG3	WJAPSCG5	WJAPSCX5
SLFRLCX5	0.702				
WJAPSC1S	0.645	0.750			
WJAPSCG3	0.646	0.680	0.729		
WJAPSCG5	0.655	0.659	0.671	0.713	
WJAPSCX5	0.650	0.606	0.606	0.613	0.650
WJPCSCG3	0.645	0.679	0.727	0.669	0.605
WJPCSCG5	0.653	0.658	0.669	0.712	0.611
WJPCSCX5	0.650	0.607	0.606	0.614	0.647
RVCSTM36	0.660	0.725	0.693	0.677	0.616
RELSTM36	0.644	0.710	0.677	0.661	0.600
PLSASC54	0.646	0.727	0.683	0.663	0.609
PLSESC54	0.643	0.721	0.677	0.658	0.606
CPINCC54	0.617	0.689	0.652	0.630	0.583
CPOMSC54	0.617	0.689	0.652	0.630	0.583
WJMSSC54	0.644	0.721	0.681	0.661	0.607
CSEX_M01	0.702	0.750	0.729	0.713	0.650
CRACEM01	0.702	0.750	0.729	0.713	0.650
MEDUCM01	0.702	0.750	0.729	0.713	0.650
TEMP_M06	0.688	0.740	0.721	0.704	0.639
MDI15O15	0.657	0.715	0.697	0.681	0.614
INCNTM01	0.644	0.699	0.685	0.666	0.600
MADEPM01	0.686	0.743	0.729	0.713	0.639

Covariance Coverage

	WJPCSCG3	WJPCSCG5	WJPCSCX5	RVCSTM36	RELSTM36
WJPCSCG3	0.727				
WJPCSCG5	0.668	0.712			
WJPCSCX5	0.605	0.612	0.650		
RVCSTM36	0.691	0.676	0.617	0.849	
RELSTM36	0.675	0.660	0.601	0.828	0.828
PLSASC54	0.681	0.662	0.609	0.753	0.738
PLSESC54	0.676	0.657	0.606	0.746	0.732
CPINCC54	0.650	0.629	0.582	0.715	0.699
CPOMSC54	0.650	0.629	0.582	0.715	0.699
WJMSSC54	0.680	0.660	0.607	0.747	0.732
CSEX_M01	0.727	0.712	0.650	0.849	0.828
CRACEM01	0.727	0.712	0.650	0.849	0.828
MEDUCM01	0.727	0.712	0.650	0.849	0.828
TEMP_M06	0.719	0.702	0.639	0.836	0.817
MDI15O15	0.696	0.680	0.614	0.810	0.792
INCNTM01	0.683	0.665	0.601	0.792	0.773
MADEPM01	0.727	0.712	0.639	0.840	0.820

Covariance Coverage

	PLSASC54	PLSESC54	CPINCC54	CPOMSC54	WJMSSC54
PLSASC54	0.780				
PLSESC54	0.772	0.773			

CPINCC54	0.727	0.720	0.735		
CPOMSC54	0.727	0.720	0.735	0.735	
WJMSSC54	0.762	0.757	0.730	0.730	0.773
CSEX_M01	0.780	0.773	0.735	0.735	0.773
CRACEM01	0.780	0.773	0.735	0.735	0.773
MEDUCM01	0.780	0.773	0.735	0.735	0.773
TEMP_M06	0.768	0.761	0.721	0.721	0.759
MDI15O15	0.739	0.732	0.699	0.699	0.735
INCNTM01	0.728	0.721	0.687	0.687	0.721
MADEPM01	0.773	0.766	0.728	0.728	0.766

Covariance Coverage

	CSEX_M01	CRACEM01	MEDUCM01	TEMP_M06	MDI15O15
CSEX_M01	1.000				
CRACEM01	1.000	1.000			
MEDUCM01	0.999	0.999	0.999		
TEMP_M06	0.938	0.938	0.938	0.938	
MDI15O15	0.865	0.865	0.865	0.855	0.865
INCNTM01	0.858	0.858	0.858	0.843	0.812
MADEPM01	0.918	0.918	0.918	0.902	0.863

Covariance Coverage

	INCNTM01	MADEPM01
INCNTM01	0.858	
MADEPM01	0.858	0.918

SAMPLE STATISTICS

ESTIMATED SAMPLE STATISTICS

Means

NONDISTR	POSREGAR	INTRUSIV	NONDISTR	POSREGAR
5.150	4.967	5.956	5.282	4.888

Means

INTRUSIV	NONDISTR	POSREGAR	INTRUSIV	SPTSIO36
6.209	5.245	4.921	6.132	5.241

Means

RSCSIO36	HOSTILIT	SPTSIO54	RSCSIO54	HOSTILIT
5.258	6.597	5.118	5.183	6.545

Means

SPTSIO1S	RSCSIO1S	HOSTILIT	SPTSIOG3	RSCSIOG3
5.085	5.189	6.429	4.935	4.845

Means

HOSTILIT	SPTSIOG5	RSCSIOG5	HOSTILIT	MRASIOX5
6.441	5.046	4.916	6.369	4.994

Means

MWRSIOX5	MHISIOX5	SLFR_O1S	SLFR_OG3	SLFR_OG5
13.036	6.156	4.634	4.332	4.674

Means

SLFRLCX5	WJAPSC1S	WJAPSCG3	WJAPSCG5	WJAPSCX5
3.431	109.715	114.328	108.822	102.200

Means

WJPCSCG3	WJPCSCG5	WJPCSCX5	RVCSTM36	RELSTM36
112.006	104.738	106.699	97.184	96.264

Means

PLSASC54	PLSESC54	CPINCC54	CPOMSC54	WJMSSC54
97.287	99.418	15.027	9.452	90.970

Means

CSEX_M01	CRACEM01	MEDUCM01	TEMP_M06	MDI15O15
1.483	3.875	14.234	3.181	108.312

Means

INCNTM01	MADEPM01
2.848	11.381

Covariances

	NONDISTR	POSREGAR	INTRUSIV	NONDISTR	POSREGAR
NONDISTR	1.631				
POSREGAR	0.843	1.390			

INTRUSIV	1.160	0.447	1.859		
NONDISTR	0.522	0.407	0.388	1.448	
POSREGAR	0.387	0.409	0.207	0.680	1.348
INTRUSIV	0.483	0.211	0.577	0.877	0.416
NONDISTR	0.460	0.289	0.347	0.482	0.400
POSREGAR	0.365	0.350	0.224	0.444	0.475
INTRUSIV	0.382	0.195	0.390	0.462	0.260
SPTSIO36	0.635	0.557	0.465	0.606	0.518
RSCSIO36	0.488	0.372	0.415	0.431	0.319
HOSTILIT	0.302	0.256	0.243	0.243	0.218
SPTSIO54	0.547	0.463	0.422	0.527	0.415
RSCSIO54	0.426	0.290	0.360	0.414	0.362
HOSTILIT	0.264	0.166	0.235	0.252	0.196
SPTSIO1S	0.590	0.490	0.429	0.651	0.548
RSCSIO1S	0.472	0.319	0.435	0.499	0.326
HOSTILIT	0.257	0.189	0.218	0.235	0.164
SPTSIOG3	0.451	0.325	0.365	0.461	0.373
RSCSIOG3	0.394	0.259	0.354	0.393	0.267
HOSTILIT	0.137	0.053	0.136	0.135	0.130
SPTSIOG5	0.374	0.275	0.288	0.377	0.287
RSCSIOG5	0.339	0.220	0.284	0.344	0.212
HOSTILIT	0.243	0.160	0.174	0.209	0.151
MRASIOX5	0.302	0.147	0.272	0.234	0.206
MWRSIOX5	0.773	0.641	0.544	0.582	0.560
MHISIOX5	-0.655	-0.290	-0.533	-0.559	-0.483
SLFR_O1S	0.189	0.170	0.176	0.174	0.077
SLFR_OG3	0.151	0.116	0.152	0.177	0.139
SLFR_OG5	0.184	0.171	0.150	0.243	0.187
SLFRLCX5	0.031	0.027	0.030	0.032	0.031
WJAPSC1S	5.629	3.902	4.747	5.409	3.088
WJAPSCG3	3.763	3.139	3.935	3.926	2.371
WJAPSCG5	3.293	2.133	3.756	3.871	2.372
WJAPSCX5	4.548	3.639	4.735	4.160	2.313
WJPCSCG3	4.047	3.265	3.752	4.345	2.762
WJPCSCG5	3.742	2.615	3.657	3.201	2.445
WJPCSCX5	5.484	4.340	5.290	5.182	3.193
RVCSTM36	5.774	4.460	4.997	6.962	4.396
RELSTM36	3.894	2.462	3.446	4.373	2.186
PLSASC54	7.183	5.483	6.511	8.596	5.361
PLSESC54	6.825	5.289	5.779	7.680	4.995
CPINCC54	-3.634	-1.739	-2.210	-3.859	-2.370
CPOMSC54	-1.638	-1.155	-1.199	-1.235	-0.648
WJMSSC54	4.785	3.609	4.047	5.801	3.271
CSEX_M01	0.021	0.003	0.039	0.031	0.018
CRACEM01	0.088	0.052	0.090	0.114	0.066
MEDUCM01	1.219	0.923	1.012	1.048	0.754
TEMP_M06	-0.074	-0.035	-0.063	-0.073	-0.046
MDI15O15	2.641	1.731	1.845	3.968	1.943
INCNTM01	0.899	0.677	0.794	0.806	0.617
MADEPM01	-2.045	-0.962	-1.614	-2.097	-1.375

Covariances

	INTRUSIV	NONDISTR	POSREGAR	INTRUSIV	SPTSIO36

INTRUSIV	1.635				
NONDISTR	0.361	1.708			
POSREGAR	0.225	0.982	1.511		
INTRUSIV	0.554	0.893	0.471	1.610	
SPTSIO36	0.426	0.724	0.614	0.504	1.761
RSCSIO36	0.432	0.553	0.394	0.532	1.009
HOSTILIT	0.232	0.348	0.258	0.331	0.602
SPTSIO54	0.336	0.674	0.560	0.430	0.855
RSCSIO54	0.393	0.530	0.388	0.470	0.622
HOSTILIT	0.240	0.360	0.249	0.370	0.431
SPTSIO1S	0.510	0.718	0.599	0.513	0.893
RSCSIO1S	0.502	0.594	0.386	0.543	0.649
HOSTILIT	0.229	0.334	0.235	0.339	0.425
SPTSIOG3	0.361	0.444	0.319	0.344	0.553
RSCSIOG3	0.362	0.380	0.231	0.323	0.427
HOSTILIT	0.130	0.197	0.106	0.192	0.173
SPTSIOG5	0.324	0.421	0.273	0.356	0.505
RSCSIOG5	0.333	0.326	0.235	0.318	0.447
HOSTILIT	0.225	0.321	0.173	0.307	0.326
MRASIOX5	0.244	0.321	0.271	0.320	0.416
MWRSIOX5	0.356	0.703	0.602	0.537	1.130
MHISIOX5	-0.607	-0.710	-0.580	-0.712	-1.034
SLFR_O1S	0.154	0.186	0.130	0.126	0.167
SLFR_OG3	0.178	0.162	0.152	0.123	0.209
SLFR_OG5	0.199	0.196	0.187	0.128	0.312
SLFRLCX5	0.051	0.041	0.053	0.021	0.043
WJAPSC1S	4.719	6.412	4.323	4.391	7.281
WJAPSCG3	4.391	4.889	4.056	3.702	6.237
WJAPSCG5	4.644	5.054	3.459	3.567	5.080
WJAPSCX5	4.602	4.599	2.965	3.208	5.877
WJPCSCG3	3.783	5.533	3.793	4.219	5.483
WJPCSCG5	2.959	4.492	2.996	3.362	4.630
WJPCSCX5	4.975	5.828	4.125	4.926	7.241
RVCSTM36	5.364	8.176	5.099	6.748	8.801
RELSTM36	2.413	5.387	3.495	4.252	5.493
PLSASC54	6.728	9.105	6.276	7.628	10.782
PLSESC54	6.265	9.303	6.047	7.428	10.592
CPINCC54	-3.508	-5.591	-3.622	-4.764	-5.550
CPOMSC54	-1.155	-1.943	-1.269	-1.420	-1.930
WJMSSC54	3.642	6.033	3.380	3.661	6.076
CSEX_M01	0.034	0.038	0.036	0.088	0.045
CRACEM01	0.147	0.104	0.065	0.084	0.105
MEDUCM01	0.852	1.045	0.866	0.854	1.319
TEMP_M06	-0.048	-0.078	-0.055	-0.068	-0.082
MDI15O15	2.599	4.494	2.992	2.953	3.328
INCNTM01	0.554	0.762	0.648	0.539	1.123
MADEPM01	-1.596	-2.053	-1.572	-1.953	-2.271

Covariances

	RSCSIO36	HOSTILIT	SPTSIO54	RSCSIO54	HOSTILIT
RSCSIO36	1.222				

HOSTILIT	0.497	0.676			
SPTSIO54	0.601	0.378	1.706		
RSCSIO54	0.550	0.283	1.050	1.238	
HOSTILIT	0.366	0.282	0.715	0.638	0.807
SPTSIO1S	0.678	0.446	0.927	0.653	0.486
RSCSIO1S	0.555	0.347	0.633	0.515	0.392
HOSTILIT	0.329	0.317	0.419	0.309	0.356
SPTSIOG3	0.462	0.238	0.546	0.417	0.278
RSCSIOG3	0.389	0.207	0.427	0.347	0.260
HOSTILIT	0.170	0.118	0.229	0.195	0.197
SPTSIOG5	0.410	0.219	0.463	0.391	0.278
RSCSIOG5	0.398	0.184	0.392	0.372	0.242
HOSTILIT	0.281	0.222	0.258	0.230	0.250
MRASIOX5	0.391	0.206	0.359	0.358	0.227
MWRSIOX5	0.927	0.548	1.136	0.952	0.523
MHISIOX5	-0.926	-0.561	-0.916	-0.915	-0.584
SLFR_O1S	0.179	0.158	0.231	0.170	0.122
SLFR_OG3	0.189	0.128	0.252	0.171	0.131
SLFR_OG5	0.188	0.086	0.270	0.165	0.109
SLFRLCX5	0.041	0.013	0.041	0.018	0.016
WJAPSC1S	6.125	3.432	7.154	5.938	3.755
WJAPSCG3	5.396	3.069	5.527	4.475	2.781
WJAPSCG5	4.466	2.873	5.333	4.650	2.823
WJAPSCX5	4.873	2.383	5.658	4.586	2.770
WJPCSCG3	4.618	2.723	5.207	4.494	2.705
WJPCSCG5	3.688	1.986	4.040	3.749	2.267
WJPCSCX5	5.673	3.162	6.277	5.316	3.206
RVCSTM36	7.525	3.534	7.439	6.189	3.395
RELSTM36	4.452	1.974	4.571	3.695	1.824
PLSASC54	8.823	4.418	8.982	7.988	4.559
PLSESC54	8.540	4.029	9.406	7.283	4.477
CPINCC54	-5.397	-3.455	-5.411	-5.851	-4.272
CPOMSC54	-1.694	-0.776	-2.727	-2.187	-1.293
WJMSSC54	6.072	2.396	5.218	4.538	2.387
CSEX_M01	0.053	0.019	-0.011	0.023	0.003
CRACEM01	0.083	0.048	0.088	0.099	0.050
MEDUCM01	1.016	0.577	1.172	0.919	0.565
TEMP_M06	-0.061	-0.035	-0.079	-0.053	-0.019
MDI15O15	2.756	1.125	3.006	2.715	1.259
INCNTM01	0.771	0.431	0.772	0.678	0.300
MADEPM01	-2.237	-1.316	-1.611	-1.289	-1.020

Covariances

	SPTSIO1S	RSCSIO1S	HOSTILIT	SPTSIOG3	RSCSIOG3
SPTSIO1S	1.985				
RSCSIO1S	1.188	1.398			
HOSTILIT	0.776	0.688	0.895		
SPTSIOG3	0.666	0.577	0.332	1.147	
RSCSIOG3	0.506	0.479	0.276	0.846	1.052
HOSTILIT	0.270	0.277	0.206	0.428	0.398
SPTSIOG5	0.579	0.442	0.256	0.439	0.386
RSCSIOG5	0.437	0.400	0.223	0.438	0.388

HOSTILIT	0.386	0.341	0.274	0.314	0.291
MRASIOX5	0.445	0.394	0.248	0.392	0.339
MWRSIOX5	1.243	0.860	0.491	0.958	0.798
MHISIOX5	-1.110	-0.966	-0.605	-0.893	-0.754
SLFR_O1S	0.236	0.208	0.096	0.255	0.240
SLFR_OG3	0.179	0.228	0.098	0.239	0.248
SLFR_OG5	0.251	0.199	0.110	0.191	0.205
SLFRLCX5	0.068	0.048	0.021	0.076	0.065
WJAPSC1S	7.444	6.064	2.804	5.177	5.036
WJAPSCG3	6.254	5.730	2.597	4.750	4.162
WJAPSCG5	5.741	5.018	2.463	4.260	4.007
WJAPSCX5	5.998	4.977	2.306	4.601	4.354
WJPCSCG3	5.694	5.147	2.457	5.060	5.023
WJPCSCG5	4.762	4.432	2.031	3.941	3.774
WJPCSCX5	6.874	6.004	3.273	5.419	5.021
RVCSTM36	8.069	6.457	2.637	5.880	6.006
RELSTM36	4.454	4.192	1.510	4.202	3.657
PLSASC54	11.033	9.294	4.035	7.966	7.355
PLSESC54	10.103	8.452	3.840	7.565	7.075
CPINCC54	-5.747	-4.669	-3.051	-4.456	-4.454
CPOMSC54	-1.858	-1.548	-0.840	-1.670	-1.444
WJMSSC54	6.412	5.184	2.569	4.230	4.378
CSEX_M01	-0.052	0.003	-0.028	0.052	0.065
CRACEM01	0.173	0.146	0.074	0.061	0.051
MEDUCM01	1.414	1.080	0.550	1.046	0.958
TEMP_M06	-0.097	-0.069	-0.020	-0.048	-0.042
MDI15O15	2.884	2.875	0.720	2.654	2.542
INCNTM01	0.951	0.615	0.287	0.794	0.719
MADEPM01	-2.000	-1.661	-1.202	-1.672	-1.379

Covariances

	HOSTILIT	SPTSIOG5	RSCSIOG5	HOSTILIT	MRASIOX5
HOSTILIT	0.695				
SPTSIOG5	0.232	0.916			
RSCSIOG5	0.207	0.706	0.894		
HOSTILIT	0.250	0.561	0.466	0.787	
MRASIOX5	0.198	0.383	0.331	0.283	1.327
MWRSIOX5	0.334	1.085	0.844	0.576	1.992
MHISIOX5	-0.591	-0.840	-0.740	-0.631	-2.704
SLFR_O1S	0.154	0.165	0.147	0.100	0.043
SLFR_OG3	0.171	0.196	0.211	0.099	0.166
SLFR_OG5	0.070	0.172	0.173	0.066	0.077
SLFRLCX5	0.047	0.042	0.063	0.026	0.038
WJAPSC1S	1.272	4.844	4.578	2.379	3.441
WJAPSCG3	1.086	4.038	3.875	2.382	2.830
WJAPSCG5	1.209	4.043	3.749	2.411	2.746
WJAPSCX5	1.191	4.239	4.146	2.694	3.196
WJPCSCG3	1.246	4.474	3.962	2.437	2.857
WJPCSCG5	0.934	3.638	3.469	2.103	2.602
WJPCSCX5	1.071	5.138	4.646	2.974	3.278
RVCSTM36	2.043	5.826	5.500	3.383	3.839
RELSTM36	1.067	3.445	3.116	1.890	2.240

PLSASC54	2.284	7.519	6.729	3.959	4.445
PLSESC54	1.873	7.651	7.167	3.745	3.802
CPINCC54	-3.135	-4.547	-4.008	-3.333	-4.351
CPOMSC54	-0.897	-1.363	-1.328	-0.843	-1.068
WJMSSC54	2.038	4.682	4.561	2.579	2.441
CSEX_M01	0.034	0.049	0.062	0.020	0.028
CRACEM01	0.009	0.099	0.086	0.054	0.050
MEDUCM01	0.369	0.818	0.810	0.456	0.822
TEMP_M06	-0.024	-0.032	-0.015	-0.011	-0.024
MDI15O15	1.196	2.975	2.405	1.488	1.256
INCNTM01	0.216	0.633	0.566	0.323	0.522
MADEPM01	-0.838	-1.769	-1.640	-1.279	-1.270

Covariances

	MWRSIOX5	MHISIOX5	SLFR_O1S	SLFR_OG3	SLFR_OG5
MWRSIOX5	8.618				
MHISIOX5	-4.556	8.071			
SLFR_O1S	0.228	-0.164	1.248		
SLFR_OG3	0.436	-0.275	0.315	0.949	
SLFR_OG5	0.314	-0.092	0.227	0.323	0.850
SLFRLCX5	0.076	-0.099	0.061	0.057	0.037
WJAPSC1S	9.673	-6.129	4.785	5.066	4.460
WJAPSCG3	7.866	-4.517	3.500	4.500	3.510
WJAPSCG5	7.606	-5.598	3.220	3.966	3.967
WJAPSCX5	9.212	-6.062	3.143	4.558	4.247
WJPCSCG3	8.198	-5.058	2.711	4.068	3.644
WJPCSCG5	7.017	-4.606	1.532	3.215	3.445
WJPCSCX5	11.392	-6.887	2.398	4.707	4.587
RVCSTM36	10.117	-7.255	3.996	3.707	3.850
RELSTM36	6.916	-3.789	2.193	3.056	2.801
PLSASC54	12.023	-7.337	4.633	4.600	4.983
PLSESC54	13.276	-6.075	4.521	5.253	5.599
CPINCC54	-8.173	9.161	-3.710	-3.541	-3.539
CPOMSC54	-2.935	2.464	-1.805	-1.072	-1.319
WJMSSC54	8.573	-4.698	2.965	2.989	2.929
CSEX_M01	0.027	-0.069	0.071	0.072	0.058
CRACEM01	0.137	-0.072	-0.006	0.036	0.045
MEDUCM01	1.918	-1.716	0.504	0.565	0.533
TEMP_M06	-0.143	0.089	-0.006	-0.006	-0.035
MDI15O15	5.529	-2.129	2.175	2.028	1.694
INCNTM01	1.454	-1.231	0.362	0.388	0.423
MADEPM01	-2.801	2.727	-0.922	-0.645	-0.782

Covariances

	SLFRLCX5	WJAPSC1S	WJAPSCG3	WJAPSCG5	WJAPSCX5
SLFRLCX5	0.151				
WJAPSC1S	1.344	307.067			
WJAPSCG3	1.226	189.769	227.487		
WJAPSCG5	0.923	170.370	155.063	183.041	
WJAPSCX5	0.877	168.461	146.131	144.465	210.598

WJPCSCG3	0.953	145.447	140.431	119.394	114.926
WJPCSCG5	1.124	121.289	110.022	103.690	101.256
WJPCSCX5	1.259	163.115	141.361	134.719	158.672
RVCSTM36	1.355	161.402	126.656	107.228	105.410
RELSTM36	0.936	97.017	83.584	63.208	67.699
PLSASC54	1.541	207.051	156.533	145.765	145.147
PLSESC54	1.972	195.270	160.894	138.416	125.164
CPINCC54	-0.810	-101.247	-70.622	-59.157	-52.445
CPOMSC54	-0.450	-43.144	-30.485	-25.557	-25.188
WJMSSC54	1.327	150.664	117.198	101.029	97.353
CSEX_M01	0.030	-0.320	-0.110	-0.073	-0.559
CRACEM01	-0.002	1.419	1.251	1.259	0.989
MEDUCM01	0.136	16.105	13.240	12.853	15.026
TEMP_M06	-0.010	-0.837	-0.851	-0.933	-0.843
MDI15O15	0.690	88.003	67.005	52.971	48.664
INCNTM01	0.128	12.020	9.068	10.172	10.082
MADEPM01	-0.264	-27.549	-19.444	-20.233	-16.753

Covariances

	WJPCSCG3	WJPCSCG5	WJPCSCX5	RVCSTM36	RELSTM36
WJPCSCG3	199.944				
WJPCSCG5	132.401	154.853			
WJPCSCX5	153.863	135.311	256.017		
RVCSTM36	118.378	95.220	130.152	256.446	
RELSTM36	82.314	73.287	87.507	136.189	214.833
PLSASC54	153.576	128.253	167.817	237.679	140.576
PLSESC54	150.354	122.357	165.166	225.592	149.258
CPINCC54	-75.084	-48.534	-76.611	-114.777	-50.079
CPOMSC54	-27.535	-21.429	-27.864	-44.847	-24.687
WJMSSC54	123.383	98.437	129.569	167.801	102.896
CSEX_M01	0.738	0.504	0.421	1.528	1.130
CRACEM01	1.100	0.970	1.626	1.751	0.961
MEDUCM01	13.913	11.818	16.745	17.924	11.461
TEMP_M06	-0.611	-0.619	-0.672	-1.008	-0.574
MDI15O15	65.533	43.888	62.545	99.987	61.061
INCNTM01	9.174	7.936	11.120	13.748	6.842
MADEPM01	-16.602	-17.975	-22.108	-28.716	-17.736

Covariances

	PLSASC54	PLSESC54	CPINCC54	CPOMSC54	WJMSSC54
PLSASC54	405.651				
PLSESC54	287.274	406.825			
CPINCC54	-146.479	-144.420	457.806		
CPOMSC54	-48.475	-51.971	40.467	58.460	
WJMSSC54	195.050	204.841	-85.754	-36.259	348.320
CSEX_M01	1.703	1.435	-2.480	-0.268	0.626
CRACEM01	2.107	2.168	-1.487	-0.400	1.118
MEDUCM01	21.719	20.492	-11.097	-3.503	13.631
TEMP_M06	-1.092	-1.271	0.591	0.213	-0.602
MDI15O15	101.662	107.185	-61.293	-22.239	87.898

INCNTM01	17.698	14.888	-7.409	-2.395	10.746
MADEPM01	-33.691	-29.315	18.098	10.137	-28.545

Covariances

	CSEX_M01	CRACEM01	MEDUCM01	TEMP_M06	MDI15O15
CSEX_M01	0.250				
CRACEM01	0.000	0.258			
MEDUCM01	0.046	0.083	6.308		
TEMP_M06	0.009	-0.028	-0.145	0.164	
MDI15O15	0.887	1.133	4.924	-0.398	198.074
INCNTM01	0.017	0.219	2.750	-0.163	4.335
MADEPM01	-0.202	-0.315	-5.276	0.876	-11.289

Covariances

	INCNTM01	MADEPM01
INCNTM01	7.177	
MADEPM01	-4.028	81.877

Correlations

	NONDISTR	POSREGAR	INTRUSIV	NONDISTR	POSREGAR
NONDISTR	1.000				
POSREGAR	0.560	1.000			
INTRUSIV	0.666	0.278	1.000		
NONDISTR	0.339	0.287	0.236	1.000	
POSREGAR	0.261	0.299	0.131	0.487	1.000
INTRUSIV	0.295	0.140	0.331	0.570	0.280
NONDISTR	0.275	0.188	0.194	0.307	0.263
POSREGAR	0.233	0.241	0.134	0.300	0.333
INTRUSIV	0.236	0.130	0.225	0.303	0.176
SPTSIO36	0.375	0.356	0.257	0.380	0.336
RSCSIO36	0.345	0.286	0.275	0.324	0.249
HOSTILIT	0.287	0.264	0.216	0.246	0.229
SPTSIO54	0.328	0.301	0.237	0.335	0.274
RSCSIO54	0.299	0.221	0.237	0.309	0.280
HOSTILIT	0.231	0.157	0.192	0.233	0.188
SPTSIO1S	0.328	0.295	0.223	0.384	0.335
RSCSIO1S	0.313	0.229	0.270	0.350	0.238
HOSTILIT	0.212	0.169	0.169	0.207	0.149
SPTSIOG3	0.330	0.257	0.250	0.358	0.300
RSCSIOG3	0.301	0.214	0.253	0.319	0.225
HOSTILIT	0.129	0.054	0.119	0.134	0.134
SPTSIOG5	0.306	0.244	0.221	0.327	0.258
RSCSIOG5	0.281	0.198	0.220	0.303	0.193
HOSTILIT	0.214	0.153	0.144	0.196	0.146
MRASIOX5	0.205	0.108	0.173	0.169	0.154
MWRSIOX5	0.206	0.185	0.136	0.165	0.164
MHISIOX5	-0.180	-0.087	-0.137	-0.163	-0.146
SLFR_O1S	0.132	0.129	0.116	0.130	0.060

SLFR_OG3	0.121	0.101	0.115	0.151	0.123
SLFR_OG5	0.156	0.157	0.120	0.219	0.174
SLFRLCX5	0.062	0.058	0.056	0.069	0.069
WJAPSC1S	0.252	0.189	0.199	0.256	0.152
WJAPSCG3	0.195	0.176	0.191	0.216	0.135
WJAPSCG5	0.191	0.134	0.204	0.238	0.151
WJAPSCX5	0.245	0.213	0.239	0.238	0.137
WJPCSCG3	0.224	0.196	0.195	0.255	0.168
WJPCSCG5	0.235	0.178	0.216	0.214	0.169
WJPCSCX5	0.268	0.230	0.242	0.269	0.172
RVCSTM36	0.282	0.236	0.229	0.361	0.236
RELSTM36	0.208	0.142	0.172	0.248	0.128
PLSASC54	0.279	0.231	0.237	0.355	0.229
PLSESC54	0.265	0.222	0.210	0.316	0.213
CPINCC54	-0.133	-0.069	-0.076	-0.150	-0.095
CPOMSC54	-0.168	-0.128	-0.115	-0.134	-0.073
WJMSSC54	0.201	0.164	0.159	0.258	0.151
CSEX_M01	0.033	0.006	0.057	0.052	0.032
CRACEM01	0.136	0.087	0.130	0.187	0.112
MEDUCM01	0.380	0.312	0.296	0.347	0.259
TEMP_M06	-0.143	-0.074	-0.114	-0.149	-0.098
MDI15O15	0.147	0.104	0.096	0.234	0.119
INCNTM01	0.263	0.214	0.217	0.250	0.198
MADEPM01	-0.177	-0.090	-0.131	-0.193	-0.131

Correlations

	INTRUSIV	NONDISTR	POSREGAR	INTRUSIV	SPTSIO36
INTRUSIV	1.000				
NONDISTR	0.216	1.000			
POSREGAR	0.143	0.611	1.000		
INTRUSIV	0.341	0.539	0.302	1.000	
SPTSIO36	0.251	0.418	0.376	0.300	1.000
RSCSIO36	0.306	0.383	0.290	0.379	0.688
HOSTILIT	0.221	0.324	0.255	0.318	0.552
SPTSIO54	0.201	0.395	0.349	0.259	0.493
RSCSIO54	0.276	0.365	0.284	0.333	0.421
HOSTILIT	0.209	0.307	0.226	0.325	0.362
SPTSIO1S	0.283	0.390	0.346	0.287	0.478
RSCSIO1S	0.332	0.384	0.266	0.362	0.413
HOSTILIT	0.190	0.270	0.202	0.282	0.338
SPTSIOG3	0.263	0.317	0.242	0.253	0.389
RSCSIOG3	0.276	0.283	0.184	0.249	0.314
HOSTILIT	0.122	0.181	0.104	0.181	0.156
SPTSIOG5	0.265	0.336	0.232	0.293	0.398
RSCSIOG5	0.275	0.263	0.203	0.265	0.357
HOSTILIT	0.198	0.277	0.158	0.273	0.277
MRASIOX5	0.166	0.214	0.191	0.219	0.272
MWRSIOX5	0.095	0.183	0.167	0.144	0.290
MHISIOX5	-0.167	-0.191	-0.166	-0.198	-0.274
SLFR_O1S	0.107	0.127	0.095	0.089	0.113
SLFR_OG3	0.143	0.127	0.127	0.100	0.162
SLFR_OG5	0.169	0.163	0.165	0.110	0.255

SLFRLCX5	0.102	0.082	0.111	0.042	0.083
WJAPSC1S	0.211	0.280	0.201	0.198	0.313
WJAPSCG3	0.228	0.248	0.219	0.193	0.312
WJAPSCG5	0.268	0.286	0.208	0.208	0.283
WJAPSCX5	0.248	0.242	0.166	0.174	0.305
WJPCSCG3	0.209	0.299	0.218	0.235	0.292
WJPCSCG5	0.186	0.276	0.196	0.213	0.280
WJPCSCX5	0.243	0.279	0.210	0.243	0.341
RVCSTM36	0.262	0.391	0.259	0.332	0.414
RELSTM36	0.129	0.281	0.194	0.229	0.282
PLSASC54	0.261	0.346	0.254	0.299	0.403
PLSESC54	0.243	0.353	0.244	0.290	0.396
CPINCC54	-0.128	-0.200	-0.138	-0.175	-0.195
CPOMSC54	-0.118	-0.194	-0.135	-0.146	-0.190
WJMSSC54	0.153	0.247	0.147	0.155	0.245
CSEX_M01	0.053	0.059	0.058	0.139	0.068
CRACEM01	0.227	0.157	0.104	0.130	0.156
MEDUCM01	0.265	0.318	0.281	0.268	0.396
TEMP_M06	-0.092	-0.148	-0.110	-0.133	-0.153
MDI15O15	0.144	0.244	0.173	0.165	0.178
INCNTM01	0.162	0.218	0.197	0.158	0.316
MADEPM01	-0.138	-0.174	-0.141	-0.170	-0.189

Correlations

	RSCSIO36	HOSTILIT	SPTSIO54	RSCSIO54	HOSTILIT
RSCSIO36	1.000				
HOSTILIT	0.547	1.000			
SPTSIO54	0.416	0.352	1.000		
RSCSIO54	0.447	0.309	0.723	1.000	
HOSTILIT	0.368	0.381	0.610	0.638	1.000
SPTSIO1S	0.436	0.385	0.504	0.416	0.384
RSCSIO1S	0.425	0.357	0.410	0.391	0.369
HOSTILIT	0.314	0.408	0.339	0.293	0.419
SPTSIOG3	0.391	0.270	0.390	0.350	0.289
RSCSIOG3	0.343	0.245	0.319	0.304	0.283
HOSTILIT	0.185	0.173	0.210	0.210	0.263
SPTSIOG5	0.388	0.279	0.370	0.367	0.323
RSCSIOG5	0.381	0.236	0.318	0.353	0.286
HOSTILIT	0.286	0.304	0.223	0.233	0.314
MRASIOX5	0.307	0.217	0.239	0.279	0.220
MWRSIOX5	0.286	0.227	0.296	0.291	0.198
MHISIOX5	-0.295	-0.240	-0.247	-0.289	-0.229
SLFR_O1S	0.145	0.172	0.159	0.137	0.121
SLFR_OG3	0.176	0.160	0.198	0.157	0.150
SLFR_OG5	0.185	0.113	0.224	0.161	0.132
SLFRLCX5	0.096	0.042	0.081	0.042	0.045
WJAPSC1S	0.316	0.238	0.313	0.305	0.239
WJAPSCG3	0.324	0.248	0.281	0.267	0.205
WJAPSCG5	0.299	0.258	0.302	0.309	0.232
WJAPSCX5	0.304	0.200	0.299	0.284	0.212
WJPCSCG3	0.296	0.234	0.282	0.286	0.213
WJPCSCG5	0.268	0.194	0.249	0.271	0.203

WJPCSCX5	0.321	0.240	0.300	0.299	0.223
RVCSTM36	0.425	0.268	0.356	0.347	0.236
RELSTM36	0.275	0.164	0.239	0.227	0.139
PLSASC54	0.396	0.267	0.341	0.356	0.252
PLSESC54	0.383	0.243	0.357	0.324	0.247
CPINCC54	-0.228	-0.196	-0.194	-0.246	-0.222
CPOMSC54	-0.200	-0.124	-0.273	-0.257	-0.188
WJMSSC54	0.294	0.156	0.214	0.218	0.142
CSEX_M01	0.097	0.047	-0.016	0.041	0.006
CRACEM01	0.147	0.114	0.132	0.176	0.111
MEDUCM01	0.366	0.280	0.357	0.329	0.251
TEMP_M06	-0.137	-0.105	-0.149	-0.117	-0.053
MDI15O15	0.177	0.097	0.164	0.173	0.100
INCNTM01	0.260	0.196	0.221	0.227	0.125
MADEPM01	-0.224	-0.177	-0.136	-0.128	-0.125

Correlations

	SPTSIO1S	RSCSIO1S	HOSTILIT	SPTSIOG3	RSCSIOG3
SPTSIO1S	1.000				
RSCSIO1S	0.713	1.000			
HOSTILIT	0.582	0.615	1.000		
SPTSIOG3	0.441	0.456	0.327	1.000	
RSCSIOG3	0.350	0.395	0.284	0.770	1.000
HOSTILIT	0.230	0.281	0.261	0.480	0.465
SPTSIOG5	0.429	0.391	0.283	0.429	0.393
RSCSIOG5	0.328	0.358	0.249	0.433	0.400
HOSTILIT	0.309	0.325	0.326	0.330	0.319
MRASIOX5	0.274	0.289	0.228	0.318	0.287
MWRSIOX5	0.301	0.248	0.177	0.305	0.265
MHISIOX5	-0.277	-0.288	-0.225	-0.294	-0.259
SLFR_O1S	0.150	0.157	0.091	0.213	0.210
SLFR_OG3	0.130	0.198	0.107	0.229	0.249
SLFR_OG5	0.193	0.183	0.126	0.193	0.217
SLFRLCX5	0.123	0.103	0.057	0.183	0.164
WJAPSC1S	0.302	0.293	0.169	0.276	0.280
WJAPSCG3	0.294	0.321	0.182	0.294	0.269
WJAPSCG5	0.301	0.314	0.192	0.294	0.289
WJAPSCX5	0.293	0.290	0.168	0.296	0.293
WJPCSCG3	0.286	0.308	0.184	0.334	0.346
WJPCSCG5	0.272	0.301	0.172	0.296	0.296
WJPCSCX5	0.305	0.317	0.216	0.316	0.306
RVCSTM36	0.358	0.341	0.174	0.343	0.366
RELSTM36	0.216	0.242	0.109	0.268	0.243
PLSASC54	0.389	0.390	0.212	0.369	0.356
PLSESC54	0.356	0.354	0.201	0.350	0.342
CPINCC54	-0.191	-0.185	-0.151	-0.194	-0.203
CPOMSC54	-0.173	-0.171	-0.116	-0.204	-0.184
WJMSSC54	0.244	0.235	0.146	0.212	0.229
CSEX_M01	-0.074	0.006	-0.060	0.098	0.127
CRACEM01	0.242	0.243	0.154	0.111	0.097
MEDUCM01	0.400	0.364	0.231	0.389	0.372
TEMP_M06	-0.171	-0.144	-0.051	-0.110	-0.101

MDI15O15	0.145	0.173	0.054	0.176	0.176
INCNTM01	0.252	0.194	0.113	0.277	0.262
MADEPM01	-0.157	-0.155	-0.140	-0.173	-0.149

Correlations

	HOSTILIT	SPTSIOG5	RSCSIOG5	HOSTILIT	MRASIOX5
HOSTILIT	1.000				
SPTSIOG5	0.291	1.000			
RSCSIOG5	0.262	0.780	1.000		
HOSTILIT	0.338	0.661	0.555	1.000	
MRASIOX5	0.206	0.347	0.304	0.277	1.000
MWRSIOX5	0.137	0.386	0.304	0.221	0.589
MHISIOX5	-0.250	-0.309	-0.276	-0.250	-0.826
SLFR_O1S	0.165	0.155	0.139	0.101	0.033
SLFR_OG3	0.210	0.211	0.229	0.114	0.148
SLFR_OG5	0.091	0.195	0.198	0.081	0.072
SLFRLCX5	0.145	0.114	0.170	0.074	0.084
WJAPSC1S	0.087	0.289	0.276	0.153	0.171
WJAPSCG3	0.086	0.280	0.272	0.178	0.163
WJAPSCG5	0.107	0.312	0.293	0.201	0.176
WJAPSCX5	0.098	0.305	0.302	0.209	0.191
WJPCSCG3	0.106	0.331	0.296	0.194	0.175
WJPCSCG5	0.090	0.305	0.295	0.190	0.182
WJPCSCX5	0.080	0.335	0.307	0.209	0.178
RVCSTM36	0.153	0.380	0.363	0.238	0.208
RELSTM36	0.087	0.246	0.225	0.145	0.133
PLSASC54	0.136	0.390	0.353	0.222	0.192
PLSESC54	0.111	0.396	0.376	0.209	0.164
CPINCC54	-0.176	-0.222	-0.198	-0.176	-0.177
CPOMSC54	-0.141	-0.186	-0.184	-0.124	-0.121
WJMSSC54	0.131	0.262	0.259	0.156	0.114
CSEX_M01	0.083	0.102	0.132	0.045	0.048
CRACEM01	0.020	0.204	0.179	0.121	0.085
MEDUCM01	0.176	0.340	0.341	0.205	0.284
TEMP_M06	-0.070	-0.083	-0.038	-0.031	-0.051
MDI15O15	0.102	0.221	0.181	0.119	0.077
INCNTM01	0.097	0.247	0.224	0.136	0.169
MADEPM01	-0.111	-0.204	-0.192	-0.159	-0.122

Correlations

	MWRSIOX5	MHISIOX5	SLFR_O1S	SLFR_OG3	SLFR_OG5
MWRSIOX5	1.000				
MHISIOX5	-0.546	1.000			
SLFR_O1S	0.070	-0.052	1.000		
SLFR_OG3	0.152	-0.099	0.289	1.000	
SLFR_OG5	0.116	-0.035	0.221	0.359	1.000
SLFRLCX5	0.067	-0.090	0.139	0.151	0.102
WJAPSC1S	0.188	-0.123	0.244	0.297	0.276
WJAPSCG3	0.178	-0.105	0.208	0.306	0.252
WJAPSCG5	0.192	-0.146	0.213	0.301	0.318

WJAPSCX5	0.216	-0.147	0.194	0.322	0.317
WJPCSCG3	0.197	-0.126	0.172	0.295	0.280
WJPCSCG5	0.192	-0.130	0.110	0.265	0.300
WJPCSCX5	0.243	-0.152	0.134	0.302	0.311
RVCSTM36	0.215	-0.159	0.223	0.238	0.261
RELSTM36	0.161	-0.091	0.134	0.214	0.207
PLSASC54	0.203	-0.128	0.206	0.235	0.268
PLSESC54	0.224	-0.106	0.201	0.267	0.301
CPINCC54	-0.130	0.151	-0.155	-0.170	-0.179
CPOMSC54	-0.131	0.113	-0.211	-0.144	-0.187
WJMSSC54	0.156	-0.089	0.142	0.164	0.170
CSEX_M01	0.018	-0.049	0.126	0.148	0.126
CRACEM01	0.092	-0.050	-0.010	0.073	0.097
MEDUCM01	0.260	-0.240	0.179	0.231	0.230
TEMP_M06	-0.121	0.078	-0.013	-0.014	-0.095
MDI15O15	0.134	-0.053	0.138	0.148	0.131
INCNTM01	0.185	-0.162	0.121	0.149	0.171
MADEPM01	-0.105	0.106	-0.091	-0.073	-0.094

Correlations

	SLFRLCX5	WJAPSC1S	WJAPSCG3	WJAPSCG5	WJAPSCX5
SLFRLCX5	1.000				
WJAPSC1S	0.197	1.000			
WJAPSCG3	0.209	0.718	1.000		
WJAPSCG5	0.175	0.719	0.760	1.000	
WJAPSCX5	0.155	0.662	0.668	0.736	1.000
WJPCSCG3	0.173	0.587	0.658	0.624	0.560
WJPCSCG5	0.232	0.556	0.586	0.616	0.561
WJPCSCX5	0.202	0.582	0.586	0.622	0.683
RVCSTM36	0.217	0.575	0.524	0.495	0.454
RELSTM36	0.164	0.378	0.378	0.319	0.318
PLSASC54	0.197	0.587	0.515	0.535	0.497
PLSESC54	0.251	0.552	0.529	0.507	0.428
CPINCC54	-0.097	-0.270	-0.219	-0.204	-0.169
CPOMSC54	-0.151	-0.322	-0.264	-0.247	-0.227
WJMSSC54	0.183	0.461	0.416	0.400	0.359
CSEX_M01	0.157	-0.037	-0.015	-0.011	-0.077
CRACEM01	-0.011	0.159	0.163	0.183	0.134
MEDUCM01	0.139	0.366	0.350	0.378	0.412
TEMP_M06	-0.062	-0.118	-0.139	-0.171	-0.144
MDI15O15	0.126	0.357	0.316	0.278	0.238
INCNTM01	0.122	0.256	0.224	0.281	0.259
MADEPM01	-0.075	-0.174	-0.142	-0.165	-0.128

Correlations

	WJPCSCG3	WJPCSCG5	WJPCSCX5	RVCSTM36	RELSTM36
WJPCSCG3	1.000				
WJPCSCG5	0.752	1.000			
WJPCSCX5	0.680	0.680	1.000		
RVCSTM36	0.523	0.478	0.508	1.000	

RELSTM36	0.397	0.402	0.373	0.580	1.000
PLSASC54	0.539	0.512	0.521	0.737	0.476
PLSESC54	0.527	0.487	0.512	0.698	0.505
CPINCC54	-0.248	-0.182	-0.224	-0.335	-0.160
CPOMSC54	-0.255	-0.225	-0.228	-0.366	-0.220
WJMSSC54	0.468	0.424	0.434	0.561	0.376
CSEX_M01	0.104	0.081	0.053	0.191	0.154
CRACEM01	0.153	0.153	0.200	0.215	0.129
MEDUCM01	0.392	0.378	0.417	0.446	0.311
TEMP_M06	-0.107	-0.123	-0.104	-0.156	-0.097
MDI15O15	0.329	0.251	0.278	0.444	0.296
INCNTM01	0.242	0.238	0.259	0.320	0.174
MADEPM01	-0.130	-0.160	-0.153	-0.198	-0.134

Correlations

	PLSASC54	PLSESC54	CPINCC54	CPOMSC54	WJMSSC54
PLSASC54	1.000				
PLSESC54	0.707	1.000			
CPINCC54	-0.340	-0.335	1.000		
CPOMSC54	-0.315	-0.337	0.247	1.000	
WJMSSC54	0.519	0.544	-0.215	-0.254	1.000
CSEX_M01	0.169	0.142	-0.232	-0.070	0.067
CRACEM01	0.206	0.212	-0.137	-0.103	0.118
MEDUCM01	0.429	0.404	-0.206	-0.182	0.291
TEMP_M06	-0.134	-0.156	0.068	0.069	-0.080
MDI15O15	0.359	0.378	-0.204	-0.207	0.335
INCNTM01	0.328	0.276	-0.129	-0.117	0.215
MADEPM01	-0.185	-0.161	0.093	0.147	-0.169

Correlations

	CSEX_M01	CRACEM01	MEDUCM01	TEMP_M06	MDI15O15
CSEX_M01	1.000				
CRACEM01	0.002	1.000			
MEDUCM01	0.036	0.065	1.000		
TEMP_M06	0.044	-0.137	-0.142	1.000	
MDI15O15	0.126	0.159	0.139	-0.070	1.000
INCNTM01	0.013	0.161	0.409	-0.151	0.115
MADEPM01	-0.045	-0.069	-0.232	0.239	-0.089

Correlations

	INCNTM01	MADEPM01
INCNTM01	1.000	
MADEPM01	-0.166	1.000

MAXIMUM LOG-LIKELIHOOD VALUE FOR THE UNRESTRICTED (H1) MODEL IS -114269.818

UNIVARIATE SAMPLE STATISTICS

UNIVARIATE HIGHER-ORDER MOMENT DESCRIPTIVE STATISTICS

Variable/ Sample Size	Mean/ Variance	Skewness/ Kurtosis	Minimum/ Maximum	% with Min/Max	Percentiles 20%/60%	40%/80%	Median
NONDISTRESS6M_7	5.170	-0.187	1.750	1.65%	3.500	5.250	5.250
1272.000	1.621	-0.467	7.000	22.64%	5.250	7.000	
POSREGARD6M_7TR	4.984	0.033	1.750	0.79%	3.500	5.250	5.250
1272.000	1.381	-0.485	7.000	15.33%	5.250	5.250	
INTRUSIVENESS6M	5.970	-1.080	1.750	1.73%	5.250	5.250	7.000
1272.000	1.854	0.210	7.000	57.55%	7.000	7.000	
NONDISTRESS15M_	5.306	-0.282	1.750	1.29%	5.250	5.250	5.250
1240.000	1.434	-0.114	7.000	23.79%	5.250	7.000	
POSREGARD15M_7T	4.906	-0.080	1.750	1.53%	3.500	5.250	5.250
1240.000	1.339	-0.181	7.000	12.42%	5.250	5.250	
INTRUSIVENESS15	6.229	-1.586	1.750	1.77%	5.250	7.000	7.000
1240.000	1.629	1.727	7.000	68.47%	7.000	7.000	
NONDISTRESS24M_	5.272	-0.406	1.750	2.65%	3.500	5.250	5.250
1172.000	1.695	-0.131	7.000	25.68%	5.250	7.000	
POSREGARD24M_7T	4.942	-0.105	1.750	2.05%	3.500	5.250	5.250
1172.000	1.494	-0.278	7.000	15.10%	5.250	5.250	
INTRUSIVENESS_R	6.156	-1.471	1.750	2.05%	5.250	7.000	7.000
1172.000	1.606	1.654	7.000	63.48%	7.000	7.000	
SPTSIO36	5.277	-0.905	1.000	1.38%	4.000	5.000	6.000
1161.000	1.732	0.717	7.000	15.85%	6.000	6.000	
RSCSIO36	5.291	-0.910	1.000	0.52%	5.000	5.000	5.000
1161.000	1.206	1.208	7.000	9.13%	6.000	6.000	
HOSTILITY36M_RE	6.618	-2.983	1.000	0.17%	6.000	7.000	7.000
1161.000	0.663	11.265	7.000	74.76%	7.000	7.000	
SPTSIO54	5.160	-0.753	1.000	0.96%	4.000	5.000	5.000
1040.000	1.682	0.354	7.000	12.88%	6.000	6.000	
RSCSIO54	5.221	-0.890	1.000	0.38%	4.000	5.000	5.000
1040.000	1.226	0.982	7.000	7.40%	6.000	6.000	
HOSTILITY54M_RE	6.568	-2.749	2.000	0.96%	6.000	7.000	7.000
1040.000	0.799	8.453	7.000	72.98%	7.000	7.000	
SPTSIO1S	5.157	-0.754	1.000	1.20%	4.000	5.000	5.000
1004.000	1.917	0.143	7.000	15.34%	6.000	6.000	
RSCSIO1S	5.256	-0.695	1.000	0.20%	4.000	5.000	5.000
1004.000	1.350	0.346	7.000	11.35%	6.000	6.000	
HOSTILITYG1_REV	6.466	-2.133	2.000	0.50%	6.000	7.000	7.000
1004.000	0.858	4.802	7.000	66.24%	7.000	7.000	
SPTSIOG3	4.990	-0.473	1.000	0.10%	4.000	5.000	5.000
982.000	1.138	0.048	7.000	4.79%	5.000	6.000	
RSCSIOG3	4.894	-0.205	1.000	0.10%	4.000	5.000	5.000
982.000	1.044	0.028	7.000	4.89%	5.000	6.000	
HOSTILITYG3_REV	6.460	-2.350	1.000	0.10%	6.000	6.000	7.000
982.000	0.694	7.899	7.000	59.67%	7.000	7.000	
SPTSIOG5	5.112	-0.611	2.000	0.54%	4.000	5.000	5.000
929.000	0.887	0.087	7.000	2.48%	5.000	6.000	
RSCSIOG5	4.977	-0.144	2.000	0.11%	4.000	5.000	5.000
929.000	0.877	-0.162	7.000	4.09%	5.000	6.000	

HOSTILITYG5_REV	6.414	-1.699	2.000	0.22%	6.000	7.000	7.000
929.000	0.759	3.104	7.000	60.39%	7.000	7.000	
MRASIOX5	5.048	-0.578	1.000	0.78%	4.000	5.000	5.000
898.000	1.304	0.668	7.000	8.57%	5.000	6.000	
MWRSIOX5	13.164	0.011	4.000	0.11%	11.000	12.000	13.000
897.000	8.567	-0.045	21.000	0.78%	14.000	16.000	
MHISIOX5	6.033	1.869	3.000	7.80%	4.000	5.000	5.000
898.000	7.912	3.957	21.000	0.11%	5.000	8.000	
SLFR_O1S	4.660	-0.329	1.000	0.10%	3.667	4.333	4.667
966.000	1.241	-0.207	7.000	1.45%	5.000	5.667	
SLFR_OG3	4.363	-0.275	1.125	0.10%	3.625	4.125	4.375
971.000	0.940	0.016	7.000	0.10%	4.625	5.125	
SLFR_OG5	4.708	-0.464	1.000	0.10%	4.000	4.500	4.750
955.000	0.845	0.506	7.000	0.10%	5.000	5.500	
SLFRLCX5	3.440	-0.885	1.300	0.10%	3.100	3.400	3.500
957.000	0.150	1.278	4.000	6.69%	3.600	3.800	
WJAPSC1S	110.804	-0.022	46.000	0.10%	96.000	106.000	111.000
1023.000	293.407	-0.049	163.000	0.10%	117.000	125.000	
WJAPSCG3	115.162	-0.806	30.000	0.10%	103.000	114.000	117.000
994.000	222.798	2.041	153.000	0.10%	120.000	127.000	
WJAPSCG5	109.324	-0.400	37.000	0.10%	98.000	106.000	110.000
973.000	180.899	1.741	156.000	0.10%	113.000	120.000	
WJAPSCX5	102.921	0.731	48.000	0.11%	92.000	97.000	99.000
887.000	202.100	1.580	168.000	0.11%	104.000	115.000	
WJPCSCG3	112.827	-0.548	47.000	0.10%	101.000	111.000	114.000
992.000	195.117	0.887	147.000	0.10%	118.000	124.000	
WJPCSCG5	105.365	-0.356	29.000	0.10%	96.000	102.000	105.000
971.000	150.681	2.486	151.000	0.10%	108.000	115.000	
WJPCSCX5	107.710	0.402	44.000	0.11%	95.000	100.000	105.000
887.000	246.894	0.529	160.000	0.11%	110.000	120.000	
RVCSTM36	97.845	-0.172	62.000	1.99%	83.000	94.000	98.000
1158.000	251.096	-0.593	136.000	0.09%	104.000	112.000	
RELSTM36	96.876	-0.095	62.000	1.06%	84.000	94.000	99.000
1130.000	210.855	-0.164	138.000	0.27%	101.000	108.000	
PLSASC54	98.345	-0.027	50.000	0.38%	79.000	88.000	98.000
1064.000	396.450	-1.095	139.000	0.19%	109.000	120.000	
PLSESC54	100.616	-0.545	50.000	0.38%	81.000	97.000	104.000
1055.000	397.668	-0.836	128.000	0.09%	111.000	119.000	
CPINCC54	14.188	2.838	0.000	8.18%	1.000	4.000	6.000
1002.000	453.297	9.632	154.000	0.10%	8.000	21.000	
CPOMSC54	9.134	1.156	0.000	4.29%	3.000	5.000	7.000
1002.000	57.607	1.000	41.067	0.10%	9.000	15.000	
WJMSSC54	91.745	-0.150	17.000	0.09%	72.000	88.000	93.000
1054.000	341.380	-0.442	142.000	0.09%	98.000	109.000	
CSEX_M01	1.483	0.067	1.000	51.69%	1.000	1.000	1.000
1364.000	0.250	-1.995	2.000	48.31%	2.000	2.000	
CRACEM01	3.875	-1.614	1.000	0.37%	4.000	4.000	4.000
1364.000	0.258	6.039	5.000	4.69%	4.000	4.000	
MEDUCM01	14.234	0.148	7.000	0.22%	12.000	14.000	14.000
1363.000	6.308	-0.036	21.000	1.69%	14.000	16.000	
TEMP_M06	3.178	-0.131	1.540	0.08%	2.852	3.094	3.200
1279.000	0.163	0.473	4.722	0.08%	3.292	3.491	
MDI15O15	108.580	-0.074	63.000	0.08%	97.000	106.000	109.000
1180.000	197.684	0.074	150.000	0.59%	112.000	121.000	

```
INCNTM01           2.960       2.529       0.082    0.09%       1.086      1.991      2.307
      1170.000      7.055      10.017      25.084    0.09%       2.736      4.153
MADEPM01          11.272       1.289       0.000    3.83%       4.000      7.000      9.000
      1252.000     81.449       1.730      53.000    0.08%      11.000     18.000
```

THIS ANALYSIS MAY HAVE MULTIPLE SOLUTIONS. EXPLORE THIS USING RANDOM STARTS,
FOR EXAMPLE, STARTS = 20. USE A LARGE ENOUGH NUMBER OF STARTS SO THAT THE
BEST FIT FUNCTION VALUE IS REPLICATED SEVERAL TIMES.

THE MODEL ESTIMATION TERMINATED NORMALLY

MODEL FIT INFORMATION

Number of Free Parameters   342

Loglikelihood

```
          H0 Value                     -115770.227
          H0 Scaling Correction Factor      1.1682
            for MLR
          H1 Value                     -114269.818
          H1 Scaling Correction Factor      1.0606
            for MLR
```

Information Criteria

```
          Akaike (AIC)                  232224.453
          Bayesian (BIC)                234009.070
          Sample-Size Adjusted BIC      232922.676
            (n* = (n + 2) / 24)
```

Chi-Square Test of Model Fit

```
          Value                           2922.733*
          Degrees of Freedom                  1088
          P-Value                           0.0000
          Scaling Correction Factor         1.0267
            for MLR
```

*   The chi-square value for MLM, MLMV, MLR, ULSMV, WLSM and WLSMV cannot be used
    for chi-square difference testing in the regular way. MLM, MLR and WLSM
    chi-square difference testing is described on the Mplus website. MLMV, WLSMV,
    and ULSMV difference testing is done using the DIFFTEST option.

RMSEA (Root Mean Square Error Of Approximation)

```
          Estimate                           0.035
          90 Percent C.I.                    0.034  0.037
          Probability RMSEA <= .05           1.000
```

CFI/TLI

CFI	0.924
TLI	0.908

Chi-Square Test of Model Fit for the Baseline Model

Value	25290.694
Degrees of Freedom	1305
P-Value	0.0000

SRMR (Standardized Root Mean Square Residual)

Value	0.073

MODEL RESULTS

	Estimate	S.E.	Est./S.E.	Two-Tailed P-Value
MS_6M BY				
NONDISTRES	1.000	0.000	999.000	999.000
POSREGARD6	0.517	0.028	18.643	0.000
INTRUSIVEN	0.715	0.033	21.530	0.000
MS_15M BY				
NONDISTRES	1.000	0.000	999.000	999.000
POSREGARD1	0.618	0.036	16.966	0.000
INTRUSIVEN	0.799	0.045	17.705	0.000
MS_24M BY				
NONDISTRES	1.000	0.000	999.000	999.000
POSREGARD2	0.698	0.032	21.812	0.000
INTRUSIVEN	0.653	0.044	14.946	0.000
MS_36M BY				
SPTSIO36	1.000	0.000	999.000	999.000
RSCSIO36	0.856	0.033	25.912	0.000
HOSTILITY3	0.502	0.036	13.836	0.000
MS_54M BY				
SPTSIO54	1.000	0.000	999.000	999.000
RSCSIO54	0.848	0.030	27.836	0.000
HOSTILITY5	0.591	0.036	16.398	0.000
MS_G1 BY				
SPTSIO1S	1.000	0.000	999.000	999.000
RSCSIO1S	0.879	0.035	24.974	0.000
HOSTILITYG	0.556	0.034	16.262	0.000
MS_G3 BY				
SPTSIOG3	1.000	0.000	999.000	999.000

```
    RSCSIOG3           0.873      0.032     27.440      0.000
    HOSTILITYG         0.452      0.036     12.405      0.000

 MS_G5    BY
    SPTSIOG5           1.000      0.000    999.000    999.000
    RSCSIOG5           0.859      0.028     30.428      0.000
    HOSTILITYG         0.664      0.034     19.369      0.000

 MS_15    BY
    MRASIOX5           1.000      0.000    999.000    999.000
    MWRSIOX5           1.707      0.080     21.348      0.000
    MHISIOX5          -2.275      0.091    -25.006      0.000

 SR       BY
    SLFR_O1S           1.000      0.000    999.000    999.000
    SLFR_OG3           1.000      0.000    999.000    999.000
    SLFR_OG5           1.000      0.000    999.000    999.000
    SLFRLCX5           1.000      0.000    999.000    999.000

 SLFR_G1R BY
    SLFR_O1S           1.000      0.000    999.000    999.000

 SLFR_G3R BY
    SLFR_OG3           1.000      0.000    999.000    999.000

 SLFR_G5R BY
    SLFR_OG5           1.000      0.000    999.000    999.000

 SLFR_15R BY
    SLFRLCX5           1.000      0.000    999.000    999.000

 READ     BY
    WJPCSCG3           1.000      0.000    999.000    999.000
    WJPCSCG5           1.000      0.000    999.000    999.000
    WJPCSCX5           1.000      0.000    999.000    999.000

 WJPCSCG3 BY
    WJPCSCG3           1.000      0.000    999.000    999.000

 WJPCSCG5 BY
    WJPCSCG5           1.000      0.000    999.000    999.000

 WJPCSCX5 BY
    WJPCSCX5           1.000      0.000    999.000    999.000

 MATH     BY
    WJAPSC1S           1.000      0.000    999.000    999.000
    WJAPSCG3           1.000      0.000    999.000    999.000
    WJAPSCG5           1.000      0.000    999.000    999.000
    WJAPSCX5           1.000      0.000    999.000    999.000

 WJAPSC1S BY
    WJAPSC1S           1.000      0.000    999.000    999.000
```

```
WJAPSCG3 BY
   WJAPSCG3           1.000      0.000    999.000    999.000

WJAPSCG5 BY
   WJAPSCG5           1.000      0.000    999.000    999.000

WJAPSCX5 BY
   WJAPSCX5           1.000      0.000    999.000    999.000

MS_RI    BY
   MS_6M              1.000      0.000    999.000    999.000
   MS_15M             1.000      0.000    999.000    999.000
   MS_24M             1.000      0.000    999.000    999.000
   MS_36M             1.000      0.000    999.000    999.000
   MS_54M             1.000      0.000    999.000    999.000
   MS_G1              1.000      0.000    999.000    999.000
   MS_G3              1.000      0.000    999.000    999.000
   MS_G5              1.000      0.000    999.000    999.000
   MS_15              1.000      0.000    999.000    999.000

MS_6MR   BY
   MS_6M              1.000      0.000    999.000    999.000

MS_15MR  BY
   MS_15M             1.000      0.000    999.000    999.000

MS_24MR  BY
   MS_24M             1.000      0.000    999.000    999.000

MS_36MR  BY
   MS_36M             1.000      0.000    999.000    999.000

MS_54MR  BY
   MS_54M             1.000      0.000    999.000    999.000

MS_G1R   BY
   MS_G1              1.000      0.000    999.000    999.000

MS_G3R   BY
   MS_G3              1.000      0.000    999.000    999.000

MS_G5R   BY
   MS_G5              1.000      0.000    999.000    999.000

MS_15R   BY
   MS_15              1.000      0.000    999.000    999.000

MS_15MR  ON
   MS_6MR             0.069      0.029      2.347      0.019

MS_24MR  ON
   MS_15MR            0.100      0.054      1.828      0.068

MS_36MR  ON
```

```
  MS_24MR            0.205      0.046      4.460      0.000

 MS_54MR  ON
  MS_36MR            0.353      0.064      5.548      0.000

 MS_G1R   ON
  MS_54MR            0.243      0.064      3.801      0.000

 SLFR_G1R ON
  MS_54MR            0.028      0.051      0.544      0.586

 WJAPSC1S ON
  MS_54MR            0.594      0.522      1.139      0.255

 MS_G3R   ON
  MS_G1R             0.156      0.048      3.275      0.001
  SLFR_G1R           0.085      0.032      2.617      0.009
  WJAPSC1S_R        -0.002      0.003     -0.663      0.507

 SLFR_G3R ON
  MS_G1R             0.060      0.046      1.292      0.196
  SLFR_G1R           0.168      0.031      5.346      0.000
  WJAPSC1S_R         0.006      0.003      1.842      0.065

 WJPCSCG3 ON
  MS_G1R             0.253      0.477      0.529      0.597
  SLFR_G1R           0.216      0.312      0.694      0.488
  WJAPSC1S_R         0.076      0.039      1.936      0.053

 WJAPSCG3 ON
  MS_G1R             0.970      0.559      1.735      0.083
  SLFR_G1R           0.220      0.327      0.674      0.500
  WJAPSC1S_R         0.229      0.045      5.135      0.000

 MS_G5R   ON
  MS_G3R             0.111      0.051      2.186      0.029
  SLFR_G3R           0.032      0.033      0.954      0.340
  WJPCSCG3_R         0.002      0.004      0.479      0.632
  WJAPSCG3_R         0.000      0.003      0.073      0.942

 SLFR_G5R ON
  MS_G3R            -0.011      0.052     -0.206      0.837
  SLFR_G3R           0.253      0.036      7.086      0.000
  WJPCSCG3_R         0.002      0.005      0.501      0.616
  WJAPSCG3_R        -0.002      0.004     -0.540      0.589

 WJPCSCG5 ON
  MS_G3R            -0.158      0.383     -0.413      0.680
  SLFR_G3R          -0.501      0.271     -1.850      0.064
  WJPCSCG3_R         0.091      0.080      1.148      0.251
  WJAPSCG3_R        -0.081      0.034     -2.374      0.018

 WJAPSCG5 ON
  MS_G3R            -0.091      0.466     -0.196      0.845
```

```
   SLFR_G3R           0.161      0.361      0.445      0.656
   WJPCSCG3_R         0.024      0.063      0.380      0.704
   WJAPSCG3_R         0.215      0.059      3.640      0.000

 MS_15R   ON
   MS_G5R             0.171      0.066      2.580      0.010
   SLFR_G5R          -0.113      0.049     -2.298      0.022
   WJPCSCG5_R         0.005      0.008      0.591      0.554
   WJAPSCG5_R         0.000      0.006     -0.071      0.943

 SLFR_15R ON
   MS_G5R             0.009      0.024      0.381      0.703
   SLFR_G5R          -0.048      0.023     -2.112      0.035
   WJPCSCG5_R         0.007      0.004      1.858      0.063
   WJAPSCG5_R        -0.005      0.002     -2.072      0.038

 WJPCSCX5 ON
   MS_G5R             0.405      0.667      0.607      0.544
   SLFR_G5R           1.445      0.501      2.885      0.004
   WJPCSCG5_R        -0.456      0.321     -1.422      0.155
   WJAPSCG5_R        -0.085      0.116     -0.728      0.467

 WJAPSCX5 ON
   MS_G5R             0.843      0.580      1.454      0.146
   SLFR_G5R           1.166      0.419      2.787      0.005
   WJPCSCG5_R        -0.440      0.195     -2.252      0.024
   WJAPSCG5_R         0.058      0.119      0.491      0.623

 MS_RI    ON
   CSEX_M01           0.066      0.035      1.915      0.056
   CRACEM01           0.243      0.041      5.870      0.000
   MEDUCM01           0.128      0.008     16.288      0.000
   TEMP_M06          -0.100      0.043     -2.329      0.020
   MDI15O15           0.008      0.001      6.792      0.000
   INCNTM01           0.036      0.009      4.174      0.000
   MADEPM01          -0.009      0.002     -4.432      0.000

 SR       ON
   CSEX_M01           0.133      0.022      6.074      0.000
   CRACEM01          -0.019      0.022     -0.864      0.388
   MEDUCM01           0.025      0.005      4.944      0.000
   TEMP_M06          -0.038      0.031     -1.223      0.221
   MDI15O15           0.004      0.001      3.780      0.000
   INCNTM01           0.013      0.005      2.593      0.010
   MADEPM01          -0.001      0.001     -0.789      0.430

 MATH     ON
   CSEX_M01          -2.704      0.654     -4.131      0.000
   CRACEM01           1.850      0.803      2.305      0.021
   MEDUCM01           1.513      0.158      9.591      0.000
   TEMP_M06          -2.061      0.855     -2.411      0.016
   MDI15O15           0.215      0.027      7.911      0.000
   INCNTM01           0.401      0.149      2.697      0.007
   MADEPM01          -0.057      0.039     -1.459      0.145
```

READ ON				
CSEX_M01	0.862	0.662	1.301	0.193
CRACEM01	2.403	0.714	3.364	0.001
MEDUCM01	1.745	0.160	10.888	0.000
TEMP_M06	-0.950	0.832	-1.142	0.253
MDI15O15	0.204	0.025	7.987	0.000
INCNTM01	0.335	0.140	2.388	0.017
MADEPM01	-0.052	0.039	-1.341	0.180
MS_54MR ON				
RVCSTM36	0.003	0.002	1.122	0.262
RELSTM36	-0.001	0.003	-0.202	0.840
MS_G1R ON				
PLSASC54	0.006	0.002	2.996	0.003
PLSESC54	0.002	0.002	0.906	0.365
CPINCC54	0.000	0.002	-0.087	0.930
CPOMSC54	0.003	0.005	0.733	0.463
WJMSSC54	-0.001	0.002	-0.254	0.799
SLFR_G1R ON				
PLSASC54	0.002	0.002	0.892	0.373
PLSESC54	0.001	0.003	0.403	0.687
CPINCC54	-0.003	0.002	-1.343	0.179
CPOMSC54	-0.019	0.005	-3.708	0.000
WJMSSC54	0.000	0.002	-0.046	0.963
WJAPSC1S ON				
PLSASC54	0.109	0.028	3.878	0.000
PLSESC54	0.051	0.029	1.799	0.072
CPINCC54	-0.035	0.017	-1.980	0.048
CPOMSC54	-0.157	0.051	-3.060	0.002
WJMSSC54	0.060	0.025	2.427	0.015
PLSASC54 ON				
MS_36MR	1.674	0.708	2.364	0.018
PLSESC54 ON				
MS_36MR	2.007	0.795	2.525	0.012
CPINCC54 ON				
MS_36MR	-2.067	1.502	-1.377	0.169
CPOMSC54 ON				
MS_36MR	-0.214	0.401	-0.534	0.594
WJMSSC54 ON				
MS_36MR	0.136	0.776	0.175	0.861
RVCSTM36 ON				
MS_24MR	2.334	0.491	4.758	0.000
RELSTM36 ON				

```
    MS_24MR            1.739      0.503      3.455      0.001

 PLSASC54 ON
    CSEX_M01           1.445      0.837      1.726      0.084
    CRACEM01           1.910      0.932      2.050      0.040
    MEDUCM01           0.813      0.205      3.966      0.000
    TEMP_M06           0.057      1.079      0.052      0.958
    MDI15O15           0.072      0.034      2.111      0.035
    INCNTM01           0.540      0.189      2.853      0.004
    MADEPM01          -0.049      0.045     -1.102      0.270
    RVCSTM36           0.721      0.038     19.230      0.000
    RELSTM36           0.078      0.036      2.150      0.032

 PLSESC54 ON
    CSEX_M01           0.489      0.886      0.551      0.581
    CRACEM01           2.379      1.033      2.302      0.021
    MEDUCM01           0.887      0.220      4.030      0.000
    TEMP_M06          -1.689      1.196     -1.412      0.158
    MDI15O15           0.137      0.036      3.847      0.000
    INCNTM01           0.182      0.176      1.034      0.301
    MADEPM01           0.010      0.054      0.191      0.848
    RVCSTM36           0.608      0.040     15.118      0.000
    RELSTM36           0.178      0.039      4.556      0.000

 CPINCC54 ON
    CSEX_M01          -8.295      1.230     -6.743      0.000
    CRACEM01          -3.214      1.370     -2.345      0.019
    MEDUCM01          -0.842      0.295     -2.855      0.004
    TEMP_M06           0.822      1.666      0.493      0.622
    MDI15O15          -0.116      0.056     -2.064      0.039
    INCNTM01          -0.028      0.266     -0.104      0.917
    MADEPM01           0.033      0.080      0.419      0.675
    RVCSTM36          -0.301      0.060     -4.983      0.000
    RELSTM36           0.115      0.058      1.989      0.047

 CPOMSC54 ON
    CSEX_M01          -0.167      0.457     -0.366      0.714
    CRACEM01          -0.306      0.504     -0.607      0.544
    MEDUCM01          -0.055      0.108     -0.514      0.607
    TEMP_M06          -0.162      0.594     -0.273      0.785
    MDI15O15          -0.033      0.019     -1.759      0.079
    INCNTM01           0.021      0.091      0.231      0.818
    MADEPM01           0.073      0.028      2.574      0.010
    RVCSTM36          -0.146      0.022     -6.587      0.000
    RELSTM36           0.001      0.020      0.045      0.964

 WJMSSC54 ON
    CSEX_M01          -1.899      0.971     -1.955      0.051
    CRACEM01          -0.577      0.904     -0.638      0.523
    MEDUCM01           0.244      0.232      1.052      0.293
    TEMP_M06           1.062      1.222      0.869      0.385
    MDI15O15           0.152      0.040      3.818      0.000
    INCNTM01           0.203      0.228      0.889      0.374
    MADEPM01          -0.113      0.058     -1.952      0.051
```

```
    RVCSTM36         0.528      0.044     12.118      0.000
    RELSTM36         0.077      0.044      1.770      0.077

 RVCSTM36 ON
    CSEX_M01         3.942      0.728      5.414      0.000
    CRACEM01         3.898      0.842      4.632      0.000
    MEDUCM01         1.987      0.160     12.393      0.000
    TEMP_M06        -1.501      0.973     -1.542      0.123
    MDI15O15         0.387      0.026     14.625      0.000
    INCNTM01         0.657      0.168      3.903      0.000
    MADEPM01        -0.068      0.044     -1.538      0.124

 RELSTM36 ON
    CSEX_M01         3.251      0.797      4.077      0.000
    CRACEM01         2.051      0.826      2.482      0.013
    MEDUCM01         1.425      0.188      7.581      0.000
    TEMP_M06        -0.747      1.017     -0.735      0.463
    MDI15O15         0.234      0.029      7.963      0.000
    INCNTM01         0.111      0.177      0.627      0.531
    MADEPM01        -0.051      0.045     -1.129      0.259

 MS_RI    WITH
    SR               0.021      0.008      2.663      0.008
    MATH             0.878      0.216      4.071      0.000
    READ             1.093      0.222      4.927      0.000

 SR       WITH
    MATH             1.007      0.187      5.385      0.000
    READ             0.774      0.155      4.979      0.000

 READ     WITH
    MATH            74.921      6.635     11.292      0.000

 MS_54MR  WITH
    PLSASC54         0.682      0.413      1.652      0.099
    PLSESC54         0.698      0.453      1.539      0.124
    CPINCC54        -1.876      0.805     -2.331      0.020
    CPOMSC54        -1.033      0.239     -4.315      0.000
    WJMSSC54        -0.276      0.483     -0.570      0.569

 MS_36MR  WITH
    RVCSTM36         1.939      0.345      5.616      0.000
    RELSTM36         0.986      0.400      2.464      0.014

 PLSASC54 WITH
    PLSESC54        65.274      6.182     10.559      0.000
    CPINCC54       -30.419      7.317     -4.157      0.000
    CPOMSC54        -5.004      2.967     -1.687      0.092
    WJMSSC54        34.087      6.358      5.361      0.000

 PLSESC54 WITH
    CPINCC54       -35.871      8.911     -4.026      0.000
    CPOMSC54       -10.481      3.421     -3.064      0.002
    WJMSSC54        49.530      7.241      6.840      0.000
```

CPINCC54 WITH

CPOMSC54	18.785	3.879	4.843	0.000
WJMSSC54	-11.145	8.178	-1.363	0.173

CPOMSC54 WITH

WJMSSC54	-5.289	3.794	-1.394	0.163

RVCSTM36 WITH

RELSTM36	68.484	5.414	12.649	0.000

CSEX_M01 WITH

CRACEM01	0.000	0.007	0.066	0.948
MEDUCM01	0.046	0.034	1.339	0.180
TEMP_M06	0.009	0.006	1.600	0.110
MDI15O15	0.892	0.202	4.409	0.000
INCNTM01	0.015	0.039	0.393	0.694
MADEPM01	-0.197	0.127	-1.557	0.120

CRACEM01 WITH

MEDUCM01	0.083	0.034	2.448	0.014
TEMP_M06	-0.028	0.006	-4.857	0.000
MDI15O15	1.114	0.192	5.799	0.000
INCNTM01	0.218	0.040	5.421	0.000
MADEPM01	-0.314	0.151	-2.074	0.038

MEDUCM01 WITH

TEMP_M06	-0.143	0.029	-4.869	0.000
MDI15O15	5.147	1.041	4.942	0.000
INCNTM01	2.760	0.204	13.518	0.000
MADEPM01	-5.233	0.669	-7.817	0.000

TEMP_M06 WITH

MDI15O15	-0.408	0.163	-2.499	0.012
INCNTM01	-0.162	0.037	-4.372	0.000
MADEPM01	0.871	0.101	8.626	0.000

MDI15O15 WITH

INCNTM01	4.484	1.164	3.850	0.000
MADEPM01	-11.463	3.774	-3.037	0.002

INCNTM01 WITH

MADEPM01	-4.122	0.629	-6.549	0.000

Means

CSEX_M01	1.483	0.014	109.614	0.000
CRACEM01	3.875	0.014	281.867	0.000
MEDUCM01	14.234	0.068	209.226	0.000
TEMP_M06	3.181	0.011	281.740	0.000
MDI15O15	108.215	0.408	264.979	0.000
INCNTM01	2.846	0.074	38.391	0.000
MADEPM01	11.364	0.257	44.233	0.000

Intercepts

NONDISTRES	1.686	0.277	6.085	0.000
POSREGARD6	3.185	0.177	18.041	0.000
INTRUSIVEN	3.480	0.237	14.679	0.000
NONDISTRES	1.819	0.278	6.535	0.000
POSREGARD1	2.752	0.222	12.400	0.000
INTRUSIVEN	3.444	0.289	11.933	0.000
NONDISTRES	1.781	0.279	6.374	0.000
POSREGARD2	2.504	0.228	10.980	0.000
INTRUSIVEN	3.877	0.252	15.371	0.000
SPTSIO36	1.780	0.279	6.369	0.000
RSCSIO36	2.298	0.265	8.681	0.000
HOSTILITY3	4.862	0.206	23.645	0.000
SPTSIO54	1.430	0.353	4.052	0.000
RSCSIO54	2.057	0.311	6.611	0.000
HOSTILITY5	4.365	0.253	17.264	0.000
SPTSIO1S	0.738	0.377	1.956	0.050
RSCSIO1S	1.371	0.344	3.987	0.000
HOSTILITYG	4.011	0.254	15.818	0.000
SPTSIOG3	1.361	0.277	4.905	0.000
RSCSIOG3	1.726	0.265	6.523	0.000
HOSTILITYG	4.822	0.191	25.298	0.000
SPTSIOG5	1.556	0.278	5.601	0.000
RSCSIOG5	1.923	0.259	7.422	0.000
HOSTILITYG	4.055	0.234	17.354	0.000
MRASIOX5	1.517	0.280	5.420	0.000
MWRSIOX5	7.136	0.553	12.908	0.000
MHISIOX5	14.065	0.734	19.167	0.000
SLFR_O1S	3.785	0.308	12.290	0.000
SLFR_OG3	3.392	0.198	17.123	0.000
SLFR_OG5	3.886	0.184	21.111	0.000
SLFRLCX5	2.672	0.185	14.428	0.000
WJAPSC1S	48.643	5.446	8.932	0.000
WJAPSCG3	67.392	5.210	12.935	0.000
WJAPSCG5	65.950	5.276	12.501	0.000
WJAPSCX5	60.230	5.343	11.272	0.000
WJPCSCG3	55.680	5.092	10.935	0.000
WJPCSCG5	50.424	5.241	9.621	0.000
WJPCSCX5	52.101	5.130	10.155	0.000
RVCSTM36	9.758	5.665	1.723	0.085
RELSTM36	40.537	6.156	6.585	0.000
PLSASC54	-10.395	6.472	-1.606	0.108
PLSESC54	-9.512	7.233	-1.315	0.188
CPINCC54	79.612	10.483	7.595	0.000
CPOMSC54	29.008	3.655	7.936	0.000
WJMSSC54	14.633	7.398	1.978	0.048

Variances

CSEX_M01	0.250	0.000	547.251	0.000
CRACEM01	0.258	0.020	13.026	0.000
MEDUCM01	6.308	0.239	26.346	0.000
TEMP_M06	0.163	0.007	22.737	0.000
MDI15O15	200.076	8.472	23.615	0.000
INCNTM01	7.182	0.726	9.888	0.000
MADEPM01	81.713	4.464	18.303	0.000

MS_6MR	1.147	0.082	14.041	0.000

Residual Variances

NONDISTRES	0.008	0.054	0.156	0.876
POSREGARD6	0.951	0.037	25.568	0.000
INTRUSIVEN	1.030	0.047	21.969	0.000
NONDISTRES	0.367	0.053	6.896	0.000
POSREGARD1	0.934	0.040	23.109	0.000
INTRUSIVEN	0.952	0.051	18.530	0.000
NONDISTRES	0.345	0.053	6.463	0.000
POSREGARD2	0.839	0.039	21.559	0.000
INTRUSIVEN	1.033	0.055	18.635	0.000
SPTSIO36	0.540	0.045	11.951	0.000
RSCSIO36	0.386	0.029	13.244	0.000
HOSTILITY3	0.381	0.030	12.714	0.000
SPTSIO54	0.469	0.038	12.274	0.000
RSCSIO54	0.345	0.029	11.746	0.000
HOSTILITY5	0.372	0.028	13.424	0.000
SPTSIO1S	0.587	0.053	11.062	0.000
RSCSIO1S	0.358	0.036	9.910	0.000
HOSTILITYG	0.462	0.032	14.460	0.000
SPTSIOG3	0.182	0.026	6.866	0.000
RSCSIOG3	0.309	0.023	13.448	0.000
HOSTILITYG	0.498	0.044	11.293	0.000
SPTSIOG5	0.097	0.017	5.641	0.000
RSCSIOG5	0.283	0.019	15.213	0.000
HOSTILITYG	0.405	0.027	14.908	0.000
MRASIOX5	0.154	0.028	5.533	0.000
MWRSIOX5	5.158	0.261	19.726	0.000
MHISIOX5	1.867	0.170	10.956	0.000
SLFR_O1S	0.000	0.000	999.000	999.000
SLFR_OG3	0.000	0.000	999.000	999.000
SLFR_OG5	0.000	0.000	999.000	999.000
SLFRLCX5	0.000	0.000	999.000	999.000
WJAPSC1S	0.000	0.000	999.000	999.000
WJAPSCG3	0.000	0.000	999.000	999.000
WJAPSCG5	0.000	0.000	999.000	999.000
WJAPSCX5	0.000	0.000	999.000	999.000
WJPCSCG3	0.000	0.000	999.000	999.000
WJPCSCG5	0.000	0.000	999.000	999.000
WJPCSCX5	0.000	0.000	999.000	999.000
RVCSTM36	148.196	6.286	23.574	0.000
RELSTM36	171.641	7.184	23.892	0.000
PLSASC54	173.012	7.151	24.194	0.000
PLSESC54	191.064	7.974	23.960	0.000
CPINCC54	381.391	40.830	9.341	0.000
CPOMSC54	49.916	2.633	18.955	0.000
WJMSSC54	230.073	11.059	20.805	0.000
MS_6M	0.000	0.000	999.000	999.000
MS_15M	0.000	0.000	999.000	999.000
MS_24M	0.000	0.000	999.000	999.000
MS_36M	0.000	0.000	999.000	999.000
MS_54M	0.000	0.000	999.000	999.000
MS_G1	0.000	0.000	999.000	999.000

MS_G3	0.000	0.000	999.000	999.000
MS_G5	0.000	0.000	999.000	999.000
MS_15	0.000	0.000	999.000	999.000
MS_RI	0.208	0.016	12.822	0.000
MS_15MR	0.606	0.061	9.933	0.000
MS_24MR	0.845	0.075	11.261	0.000
MS_36MR	0.544	0.045	12.070	0.000
MS_54MR	0.647	0.051	12.698	0.000
MS_G1R	0.645	0.050	12.967	0.000
MS_G3R	0.510	0.038	13.322	0.000
MS_G5R	0.451	0.034	13.448	0.000
MS_15R	0.878	0.063	13.909	0.000
SR	0.035	0.013	2.739	0.006
SLFR_G1R	1.097	0.051	21.577	0.000
SLFR_G3R	0.789	0.035	22.318	0.000
SLFR_G5R	0.684	0.036	19.259	0.000
SLFR_15R	0.110	0.014	7.626	0.000
READ	97.464	7.832	12.445	0.000
WJPCSCG3_R	62.820	3.982	15.775	0.000
WJPCSCG5_R	32.837	9.791	3.354	0.001
WJPCSCX5_R	68.259	10.904	6.260	0.000
MATH	87.660	10.035	8.735	0.000
WJAPSC1S_R	108.015	6.269	17.231	0.000
WJAPSCG3_R	77.919	4.846	16.080	0.000
WJAPSCG5_R	47.372	5.333	8.883	0.000
WJAPSCX5_R	55.115	6.764	8.148	0.000

QUALITY OF NUMERICAL RESULTS

Condition Number for the Information Matrix 0.219E-08
(ratio of smallest to largest eigenvalue)

STANDARDIZED MODEL RESULTS

STDYX Standardization

	Estimate	S.E.	Est./S.E.	Two-Tailed P-Value
MS_6M BY				
NONDISTRES	0.997	0.017	59.137	0.000
POSREGARD6	0.555	0.021	25.995	0.000
INTRUSIVEN	0.664	0.017	38.011	0.000
MS_15M BY				
NONDISTRES	0.861	0.021	40.212	0.000
POSREGARD1	0.549	0.025	22.162	0.000
INTRUSIVEN	0.643	0.022	28.903	0.000
MS_24M BY				
NONDISTRES	0.889	0.019	47.724	0.000

```
    POSREGARD2         0.655      0.020     32.501      0.000
    INTRUSIVEN         0.590      0.025     23.989      0.000

 MS_36M   BY
    SPTSIO36           0.809      0.015     52.768      0.000
    RSCSIO36           0.813      0.016     52.160      0.000
    HOSTILITY3         0.635      0.024     26.088      0.000

 MS_54M   BY
    SPTSIO54           0.847      0.013     64.780      0.000
    RSCSIO54           0.844      0.016     52.419      0.000
    HOSTILITY5         0.726      0.021     35.038      0.000

 MS_G1    BY
    SPTSIO1S           0.825      0.016     50.639      0.000
    RSCSIO1S           0.854      0.017     51.106      0.000
    HOSTILITYG         0.674      0.023     28.692      0.000

 MS_G3    BY
    SPTSIOG3           0.919      0.012     74.387      0.000
    RSCSIOG3           0.843      0.014     59.959      0.000
    HOSTILITYG         0.538      0.026     20.501      0.000

 MS_G5    BY
    SPTSIOG5           0.950      0.009    103.883      0.000
    RSCSIOG5           0.838      0.013     64.874      0.000
    HOSTILITYG         0.704      0.020     34.880      0.000

 MS_15    BY
    MRASIOX5           0.947      0.010     95.664      0.000
    MWRSIOX5           0.657      0.021     31.276      0.000
    MHISIOX5          -0.888      0.011    -82.428      0.000

 SR       BY
    SLFR_O1S           0.208      0.028      7.355      0.000
    SLFR_OG3           0.242      0.032      7.639      0.000
    SLFR_OG5           0.257      0.035      7.375      0.000
    SLFRLCX5           0.561      0.069      8.100      0.000

 SLFR_G1R BY
    SLFR_O1S           0.969      0.006    149.588      0.000

 SLFR_G3R BY
    SLFR_OG3           0.965      0.008    119.479      0.000

 SLFR_G5R BY
    SLFR_OG5           0.965      0.009    104.857      0.000

 SLFR_15R BY
    SLFRLCX5           0.829      0.047     17.779      0.000

 READ     BY
    WJPCSCG3           0.823      0.012     69.670      0.000
    WJPCSCG5           0.899      0.030     29.994      0.000
```

```
    WJPCSCX5           0.802      0.016     49.147      0.000

 WJPCSCG3 BY
    WJPCSCG3           0.555      0.017     32.652      0.000

 WJPCSCG5 BY
    WJPCSCG5           0.443      0.059      7.497      0.000

 WJPCSCX5 BY
    WJPCSCX5           0.596      0.022     26.719      0.000

 MATH     BY
    WJAPSC1S           0.663      0.028     23.748      0.000
    WJAPSCG3           0.757      0.027     27.818      0.000
    WJAPSCG5           0.839      0.030     28.047      0.000
    WJAPSCX5           0.816      0.028     29.118      0.000

 WJAPSC1S BY
    WJAPSC1S           0.671      0.025     27.070      0.000

 WJAPSCG3 BY
    WJAPSCG3           0.623      0.028     22.439      0.000

 WJAPSCG5 BY
    WJAPSCG5           0.534      0.043     12.523      0.000

 WJAPSCX5 BY
    WJAPSCX5           0.576      0.038     15.277      0.000

 MS_RI    BY
    MS_6M              0.528      0.019     28.149      0.000
    MS_15M             0.648      0.023     28.723      0.000
    MS_24M             0.585      0.021     27.688      0.000
    MS_36M             0.658      0.020     33.628      0.000
    MS_54M             0.610      0.020     29.880      0.000
    MS_G1              0.596      0.021     28.615      0.000
    MS_G3              0.667      0.019     35.878      0.000
    MS_G5              0.699      0.018     39.868      0.000
    MS_15              0.574      0.018     32.669      0.000

 MS_6MR   BY
    MS_6M              0.849      0.012     72.971      0.000

 MS_15MR  BY
    MS_15M             0.762      0.019     39.658      0.000

 MS_24MR  BY
    MS_24M             0.811      0.015     53.240      0.000

 MS_36MR  BY
    MS_36M             0.753      0.017     44.066      0.000

 MS_54MR  BY
    MS_54M             0.781      0.016     50.176      0.000
```

```
 MS_G1R   BY
    MS_G1              0.759      0.016     46.352      0.000

 MS_G3R   BY
    MS_G3              0.734      0.016     45.665      0.000

 MS_G5R   BY
    MS_G5              0.712      0.017     42.025      0.000

 MS_15R   BY
    MS_15              0.819      0.012     66.332      0.000

 MS_15MR  ON
    MS_6MR             0.094      0.040      2.378      0.017

 MS_24MR  ON
    MS_15MR            0.084      0.047      1.813      0.070

 MS_36MR  ON
    MS_24MR            0.248      0.050      4.929      0.000

 MS_54MR  ON
    MS_36MR            0.316      0.056      5.620      0.000

 MS_G1R   ON
    MS_54MR            0.244      0.062      3.960      0.000

 SLFR_G1R ON
    MS_54MR            0.022      0.041      0.544      0.586

 WJAPSC1S ON
    MS_54MR            0.044      0.039      1.139      0.255

 MS_G3R   ON
    MS_G1R             0.180      0.053      3.372      0.001
    SLFR_G1R           0.124      0.047      2.615      0.009
    WJAPSC1S_R        -0.031      0.046     -0.667      0.505

 SLFR_G3R ON
    MS_G1R             0.056      0.043      1.290      0.197
    SLFR_G1R           0.196      0.036      5.403      0.000
    WJAPSC1S_R         0.077      0.042      1.818      0.069

 WJPCSCG3 ON
    MS_G1R             0.027      0.051      0.530      0.596
    SLFR_G1R           0.029      0.041      0.696      0.486
    WJAPSC1S_R         0.109      0.057      1.919      0.055

 WJAPSCG3 ON
    MS_G1R             0.089      0.050      1.763      0.078
    SLFR_G1R           0.025      0.037      0.677      0.499
    WJAPSC1S_R         0.282      0.052      5.392      0.000
```

```
MS_G5R   ON
   MS_G3R             0.120      0.055      2.187      0.029
   SLFR_G3R           0.043      0.045      0.954      0.340
   WJPCSCG3_R         0.022      0.046      0.480      0.631
   WJAPSCG3_R         0.003      0.045      0.073      0.942

SLFR_G5R ON
   MS_G3R            -0.009      0.044     -0.206      0.837
   SLFR_G3R           0.269      0.036      7.478      0.000
   WJPCSCG3_R         0.021      0.043      0.501      0.616
   WJAPSCG3_R        -0.023      0.042     -0.543      0.587

WJPCSCG5 ON
   MS_G3R            -0.020      0.047     -0.418      0.676
   SLFR_G3R          -0.078      0.047     -1.659      0.097
   WJPCSCG3_R         0.125      0.091      1.367      0.172
   WJAPSCG3_R        -0.129      0.054     -2.377      0.017

WJAPSCG5 ON
   MS_G3R            -0.009      0.048     -0.196      0.845
   SLFR_G3R           0.020      0.045      0.450      0.653
   WJPCSCG3_R         0.027      0.070      0.382      0.703
   WJAPSCG3_R         0.277      0.067      4.166      0.000

MS_15R   ON
   MS_G5R             0.122      0.047      2.565      0.010
   SLFR_G5R          -0.102      0.044     -2.334      0.020
   WJPCSCG5_R         0.030      0.050      0.593      0.553
   WJAPSCG5_R        -0.003      0.044     -0.071      0.943

SLFR_15R ON
   MS_G5R             0.018      0.048      0.380      0.704
   SLFR_G5R          -0.121      0.061     -1.986      0.047
   WJPCSCG5_R         0.113      0.069      1.640      0.101
   WJAPSCG5_R        -0.104      0.050     -2.065      0.039

WJPCSCX5 ON
   MS_G5R             0.031      0.051      0.607      0.544
   SLFR_G5R           0.141      0.049      2.904      0.004
   WJPCSCG5_R        -0.303      0.173     -1.756      0.079
   WJAPSCG5_R        -0.069      0.092     -0.753      0.452

WJAPSCX5 ON
   MS_G5R             0.072      0.050      1.447      0.148
   SLFR_G5R           0.126      0.044      2.836      0.005
   WJPCSCG5_R        -0.323      0.095     -3.412      0.001
   WJAPSCG5_R         0.052      0.108      0.487      0.626

MS_RI    ON
   CSEX_M01           0.050      0.026      1.919      0.055
   CRACEM01           0.186      0.030      6.291      0.000
   MEDUCM01           0.485      0.027     17.686      0.000
   TEMP_M06          -0.061      0.026     -2.350      0.019
   MDI15O15           0.181      0.026      6.853      0.000
```

```
    INCNTM01           0.144      0.031      4.624      0.000
    MADEPM01          -0.126      0.028     -4.497      0.000

 SR       ON
    CSEX_M01           0.289      0.060      4.784      0.000
    CRACEM01          -0.043      0.050     -0.856      0.392
    MEDUCM01           0.275      0.061      4.527      0.000
    TEMP_M06          -0.068      0.054     -1.249      0.212
    MDI15O15           0.221      0.056      3.967      0.000
    INCNTM01           0.147      0.057      2.563      0.010
    MADEPM01          -0.044      0.057     -0.782      0.434

 MATH     ON
    CSEX_M01          -0.120      0.030     -3.974      0.000
    CRACEM01           0.083      0.035      2.374      0.018
    MEDUCM01           0.337      0.030     11.098      0.000
    TEMP_M06          -0.074      0.030     -2.463      0.014
    MDI15O15           0.269      0.031      8.718      0.000
    INCNTM01           0.095      0.034      2.767      0.006
    MADEPM01          -0.046      0.032     -1.440      0.150

 READ     ON
    CSEX_M01           0.036      0.028      1.305      0.192
    CRACEM01           0.103      0.030      3.485      0.000
    MEDUCM01           0.370      0.032     11.539      0.000
    TEMP_M06          -0.032      0.028     -1.153      0.249
    MDI15O15           0.243      0.030      8.028      0.000
    INCNTM01           0.076      0.031      2.417      0.016
    MADEPM01          -0.040      0.030     -1.334      0.182

 MS_54MR  ON
    RVCSTM36           0.052      0.046      1.128      0.259
    RELSTM36          -0.009      0.047     -0.202      0.840

 MS_G1R   ON
    PLSASC54           0.151      0.049      3.063      0.002
    PLSESC54           0.052      0.057      0.905      0.365
    CPINCC54          -0.005      0.052     -0.087      0.930
    CPOMSC54           0.031      0.043      0.733      0.464
    WJMSSC54          -0.011      0.044     -0.254      0.799

 SLFR_G1R ON
    PLSASC54           0.040      0.045      0.892      0.372
    PLSESC54           0.019      0.047      0.404      0.687
    CPINCC54          -0.054      0.040     -1.345      0.179
    CPOMSC54          -0.136      0.036     -3.728      0.000
    WJMSSC54          -0.002      0.041     -0.046      0.963

 WJAPSC1S ON
    PLSASC54           0.189      0.047      4.057      0.000
    PLSESC54           0.090      0.049      1.820      0.069
    CPINCC54          -0.065      0.033     -1.975      0.048
    CPOMSC54          -0.105      0.034     -3.112      0.002
    WJMSSC54           0.097      0.040      2.443      0.015
```

```
PLSASC54 ON
   MS_36MR            0.064      0.027      2.356      0.018

PLSESC54 ON
   MS_36MR            0.077      0.030      2.521      0.012

CPINCC54 ON
   MS_36MR           -0.074      0.054     -1.369      0.171

CPOMSC54 ON
   MS_36MR           -0.021      0.040     -0.534      0.593

WJMSSC54 ON
   MS_36MR            0.006      0.032      0.175      0.861

RVCSTM36 ON
   MS_24MR            0.136      0.028      4.847      0.000

RELSTM36 ON
   MS_24MR            0.110      0.032      3.489      0.000

PLSASC54 ON
   CSEX_M01           0.036      0.021      1.727      0.084
   CRACEM01           0.049      0.024      2.061      0.039
   MEDUCM01           0.103      0.026      3.973      0.000
   TEMP_M06           0.001      0.022      0.052      0.958
   MDI15O15           0.051      0.024      2.111      0.035
   INCNTM01           0.073      0.025      2.882      0.004
   MADEPM01          -0.022      0.020     -1.098      0.272
   RVCSTM36           0.574      0.029     19.582      0.000
   RELSTM36           0.057      0.026      2.160      0.031

PLSESC54 ON
   CSEX_M01           0.012      0.022      0.551      0.581
   CRACEM01           0.061      0.026      2.300      0.021
   MEDUCM01           0.112      0.028      4.037      0.000
   TEMP_M06          -0.034      0.024     -1.412      0.158
   MDI15O15           0.097      0.025      3.838      0.000
   INCNTM01           0.024      0.024      1.040      0.298
   MADEPM01           0.005      0.024      0.191      0.848
   RVCSTM36           0.483      0.032     15.097      0.000
   RELSTM36           0.130      0.028      4.602      0.000

CPINCC54 ON
   CSEX_M01          -0.194      0.025     -7.627      0.000
   CRACEM01          -0.076      0.032     -2.419      0.016
   MEDUCM01          -0.099      0.034     -2.953      0.003
   TEMP_M06           0.016      0.032      0.493      0.622
   MDI15O15          -0.077      0.037     -2.058      0.040
   INCNTM01          -0.003      0.033     -0.104      0.917
   MADEPM01           0.014      0.034      0.420      0.674
   RVCSTM36          -0.223      0.043     -5.248      0.000
   RELSTM36           0.078      0.039      2.008      0.045
```

```
CPOMSC54 ON
   CSEX_M01          -0.011      0.030     -0.366      0.714
   CRACEM01          -0.020      0.033     -0.609      0.542
   MEDUCM01          -0.018      0.036     -0.513      0.608
   TEMP_M06          -0.009      0.032     -0.273      0.785
   MDI15O15          -0.061      0.035     -1.764      0.078
   INCNTM01           0.007      0.032      0.230      0.818
   MADEPM01           0.086      0.033      2.578      0.010
   RVCSTM36          -0.304      0.045     -6.782      0.000
   RELSTM36           0.002      0.039      0.045      0.964

WJMSSC54 ON
   CSEX_M01          -0.051      0.026     -1.951      0.051
   CRACEM01          -0.016      0.025     -0.639      0.523
   MEDUCM01           0.033      0.031      1.057      0.291
   TEMP_M06           0.023      0.027      0.870      0.384
   MDI15O15           0.116      0.030      3.819      0.000
   INCNTM01           0.029      0.033      0.899      0.369
   MADEPM01          -0.055      0.028     -1.957      0.050
   RVCSTM36           0.451      0.037     12.299      0.000
   RELSTM36           0.061      0.034      1.770      0.077

RVCSTM36 ON
   CSEX_M01           0.124      0.023      5.449      0.000
   CRACEM01           0.125      0.026      4.790      0.000
   MEDUCM01           0.315      0.025     12.515      0.000
   TEMP_M06          -0.038      0.025     -1.548      0.122
   MDI15O15           0.346      0.023     15.208      0.000
   INCNTM01           0.111      0.028      3.935      0.000
   MADEPM01          -0.039      0.025     -1.541      0.123

RELSTM36 ON
   CSEX_M01           0.112      0.027      4.115      0.000
   CRACEM01           0.072      0.029      2.489      0.013
   MEDUCM01           0.246      0.032      7.694      0.000
   TEMP_M06          -0.021      0.028     -0.735      0.462
   MDI15O15           0.227      0.028      8.089      0.000
   INCNTM01           0.020      0.032      0.634      0.526
   MADEPM01          -0.032      0.028     -1.129      0.259

MS_RI    WITH
   SR                 0.243      0.095      2.565      0.010
   MATH               0.205      0.045      4.518      0.000
   READ               0.243      0.046      5.238      0.000

SR       WITH
   MATH               0.575      0.123      4.672      0.000
   READ               0.419      0.092      4.530      0.000

READ     WITH
   MATH               0.811      0.043     18.686      0.000

MS_54MR  WITH
```

```
    PLSASC54           0.064      0.039      1.659      0.097
    PLSESC54           0.063      0.040      1.553      0.120
    CPINCC54          -0.119      0.050     -2.373      0.018
    CPOMSC54          -0.182      0.041     -4.480      0.000
    WJMSSC54          -0.023      0.040     -0.570      0.569

 MS_36MR  WITH
    RVCSTM36           0.216      0.036      6.000      0.000
    RELSTM36           0.102      0.041      2.512      0.012

 PLSASC54 WITH
    PLSESC54           0.359      0.028     12.803      0.000
    CPINCC54          -0.118      0.027     -4.366      0.000
    CPOMSC54          -0.054      0.032     -1.698      0.090
    WJMSSC54           0.171      0.031      5.574      0.000

 PLSESC54 WITH
    CPINCC54          -0.133      0.030     -4.490      0.000
    CPOMSC54          -0.107      0.034     -3.122      0.002
    WJMSSC54           0.236      0.031      7.513      0.000

 CPINCC54 WITH
    CPOMSC54           0.136      0.031      4.417      0.000
    WJMSSC54          -0.038      0.027     -1.371      0.170

 CPOMSC54 WITH
    WJMSSC54          -0.049      0.035     -1.403      0.161

 RVCSTM36 WITH
    RELSTM36           0.429      0.025     17.037      0.000

 CSEX_M01 WITH
    CRACEM01           0.002      0.027      0.066      0.948
    MEDUCM01           0.036      0.027      1.340      0.180
    TEMP_M06           0.045      0.028      1.604      0.109
    MDI15O15           0.126      0.028      4.467      0.000
    INCNTM01           0.011      0.029      0.395      0.693
    MADEPM01          -0.044      0.028     -1.563      0.118

 CRACEM01 WITH
    MEDUCM01           0.065      0.027      2.408      0.016
    TEMP_M06          -0.136      0.027     -5.016      0.000
    MDI15O15           0.155      0.026      5.889      0.000
    INCNTM01           0.161      0.027      5.856      0.000
    MADEPM01          -0.068      0.033     -2.070      0.038

 MEDUCM01 WITH
    TEMP_M06          -0.141      0.028     -4.956      0.000
    MDI15O15           0.145      0.028      5.090      0.000
    INCNTM01           0.410      0.023     18.140      0.000
    MADEPM01          -0.231      0.027     -8.499      0.000

 TEMP_M06 WITH
    MDI15O15          -0.071      0.028     -2.527      0.011
```

```
    INCNTM01          -0.150      0.031     -4.815      0.000
    MADEPM01           0.238      0.025      9.457      0.000

 MDI15O15 WITH
    INCNTM01           0.118      0.032      3.711      0.000
    MADEPM01          -0.090      0.029     -3.079      0.002

 INCNTM01 WITH
    MADEPM01          -0.170      0.024     -7.051      0.000

 Means
    CSEX_M01           2.968      0.024    121.814      0.000
    CRACEM01           7.632      0.309     24.685      0.000
    MEDUCM01           5.667      0.108     52.424      0.000
    TEMP_M06           7.867      0.177     44.347      0.000
    MDI15O15           7.651      0.167     45.814      0.000
    INCNTM01           1.062      0.040     26.628      0.000
    MADEPM01           1.257      0.026     48.377      0.000

 Intercepts
    NONDISTRES         1.334      0.225      5.921      0.000
    POSREGARD6         2.716      0.170     15.957      0.000
    INTRUSIVEN         2.564      0.200     12.790      0.000
    NONDISTRES         1.526      0.240      6.355      0.000
    POSREGARD1         2.381      0.212     11.220      0.000
    INTRUSIVEN         2.702      0.268     10.084      0.000
    NONDISTRES         1.391      0.223      6.227      0.000
    POSREGARD2         2.066      0.204     10.131      0.000
    INTRUSIVEN         3.080      0.254     12.140      0.000
    SPTSIO36           1.424      0.232      6.144      0.000
    RSCSIO36           2.157      0.274      7.878      0.000
    HOSTILITY3         6.082      0.523     11.634      0.000
    SPTSIO54           1.111      0.282      3.932      0.000
    RSCSIO54           1.877      0.307      6.124      0.000
    HOSTILITY5         4.921      0.470     10.478      0.000
    SPTSIO1S           0.545      0.283      1.927      0.054
    RSCSIO1S           1.193      0.312      3.818      0.000
    HOSTILITYG         4.358      0.406     10.737      0.000
    SPTSIOG3           1.255      0.263      4.771      0.000
    RSCSIOG3           1.671      0.272      6.141      0.000
    HOSTILITYG         5.760      0.475     12.134      0.000
    SPTSIOG5           1.555      0.287      5.413      0.000
    RSCSIOG5           1.972      0.284      6.946      0.000
    HOSTILITYG         4.523      0.379     11.940      0.000
    MRASIOX5           1.240      0.237      5.227      0.000
    MWRSIOX5           2.369      0.213     11.146      0.000
    MHISIOX5           4.736      0.217     21.807      0.000
    SLFR_O1S           3.435      0.297     11.582      0.000
    SLFR_OG3           3.588      0.233     15.427      0.000
    SLFR_OG5           4.365      0.239     18.288      0.000
    SLFRLCX5           6.540      0.569     11.503      0.000
    WJAPSC1S           2.859      0.347      8.234      0.000
    WJAPSCG3           4.526      0.415     10.899      0.000
    WJAPSCG5           4.907      0.460     10.657      0.000
```

WJAPSCX5	4.356	0.450	9.682	0.000
WJPCSCG3	3.867	0.398	9.713	0.000
WJPCSCG5	3.827	0.483	7.924	0.000
WJPCSCX5	3.529	0.380	9.285	0.000
RVCSTM36	0.616	0.362	1.702	0.089
RELSTM36	2.785	0.443	6.289	0.000
PLSASC54	-0.522	0.324	-1.615	0.106
PLSESC54	-0.477	0.360	-1.323	0.186
CPINCC54	3.727	0.433	8.603	0.000
CPOMSC54	3.804	0.457	8.327	0.000
WJMSSC54	0.789	0.403	1.957	0.050

Variances

CSEX_M01	1.000	0.000	999.000	999.000
CRACEM01	1.000	0.000	999.000	999.000
MEDUCM01	1.000	0.000	999.000	999.000
TEMP_M06	1.000	0.000	999.000	999.000
MDI15O15	1.000	0.000	999.000	999.000
INCNTM01	1.000	0.000	999.000	999.000
MADEPM01	1.000	0.000	999.000	999.000
MS_6MR	1.000	0.000	999.000	999.000

Residual Variances

NONDISTRES	0.005	0.034	0.156	0.876
POSREGARD6	0.691	0.024	29.127	0.000
INTRUSIVEN	0.559	0.023	24.115	0.000
NONDISTRES	0.258	0.037	7.001	0.000
POSREGARD1	0.699	0.027	25.749	0.000
INTRUSIVEN	0.586	0.029	20.472	0.000
NONDISTRES	0.210	0.033	6.360	0.000
POSREGARD2	0.571	0.026	21.606	0.000
INTRUSIVEN	0.652	0.029	22.492	0.000
SPTSIO36	0.345	0.025	13.924	0.000
RSCSIO36	0.340	0.025	13.425	0.000
HOSTILITY3	0.597	0.031	19.307	0.000
SPTSIO54	0.283	0.022	12.762	0.000
RSCSIO54	0.287	0.027	10.556	0.000
HOSTILITY5	0.473	0.030	15.693	0.000
SPTSIO1S	0.320	0.027	11.923	0.000
RSCSIO1S	0.271	0.029	9.503	0.000
HOSTILITYG	0.546	0.032	17.240	0.000
SPTSIOG3	0.155	0.023	6.803	0.000
RSCSIOG3	0.290	0.024	12.230	0.000
HOSTILITYG	0.711	0.028	25.184	0.000
SPTSIOG5	0.097	0.017	5.594	0.000
RSCSIOG5	0.298	0.022	13.749	0.000
HOSTILITYG	0.504	0.028	17.732	0.000
MRASIOX5	0.103	0.019	5.499	0.000
MWRSIOX5	0.568	0.028	20.592	0.000
MHISIOX5	0.212	0.019	11.067	0.000
SLFR_O1S	0.000	999.000	999.000	999.000
SLFR_OG3	0.000	999.000	999.000	999.000
SLFR_OG5	0.000	999.000	999.000	999.000
SLFRLCX5	0.000	999.000	999.000	999.000

WJAPSC1S	0.000	999.000	999.000	999.000
WJAPSCG3	0.000	999.000	999.000	999.000
WJAPSCG5	0.000	999.000	999.000	999.000
WJAPSCX5	0.000	999.000	999.000	999.000
WJPCSCG3	0.000	999.000	999.000	999.000
WJPCSCG5	0.000	999.000	999.000	999.000
WJPCSCX5	0.000	999.000	999.000	999.000
RVCSTM36	0.591	0.023	25.378	0.000
RELSTM36	0.810	0.022	36.447	0.000
PLSASC54	0.437	0.019	22.740	0.000
PLSESC54	0.480	0.021	23.097	0.000
CPINCC54	0.836	0.020	42.535	0.000
CPOMSC54	0.858	0.020	42.588	0.000
WJMSSC54	0.669	0.025	26.741	0.000
MS_6M	0.000	999.000	999.000	999.000
MS_15M	0.000	999.000	999.000	999.000
MS_24M	0.000	999.000	999.000	999.000
MS_36M	0.000	999.000	999.000	999.000
MS_54M	0.000	999.000	999.000	999.000
MS_G1	0.000	999.000	999.000	999.000
MS_G3	0.000	999.000	999.000	999.000
MS_G5	0.000	999.000	999.000	999.000
MS_15	0.000	999.000	999.000	999.000
MS_RI	0.470	0.027	17.469	0.000
MS_15MR	0.991	0.007	132.456	0.000
MS_24MR	0.993	0.008	126.421	0.000
MS_36MR	0.938	0.025	37.471	0.000
MS_54MR	0.893	0.035	25.659	0.000
MS_G1R	0.898	0.036	25.213	0.000
MS_G3R	0.951	0.022	42.372	0.000
MS_G5R	0.983	0.014	71.590	0.000
MS_15R	0.974	0.015	65.014	0.000
SR	0.668	0.083	8.055	0.000
SLFR_G1R	0.963	0.014	70.437	0.000
SLFR_G3R	0.949	0.017	56.199	0.000
SLFR_G5R	0.927	0.019	47.803	0.000
SLFR_15R	0.960	0.026	37.082	0.000
READ	0.695	0.031	22.244	0.000
WJPCSCG3_R	0.986	0.014	70.816	0.000
WJPCSCG5_R	0.962	0.024	40.418	0.000
WJPCSCX5_R	0.883	0.099	8.890	0.000
MATH	0.689	0.027	25.834	0.000
WJAPSC1S_R	0.830	0.029	28.860	0.000
WJAPSCG3_R	0.907	0.034	26.845	0.000
WJAPSCG5_R	0.921	0.039	23.337	0.000
WJAPSCX5_R	0.869	0.071	12.230	0.000

R-SQUARE

Observed Variable	Estimate	S.E.	Two-Tailed Est./S.E.	P-Value
NONDISTR	0.995	0.034	29.568	0.000

POSREGAR	0.309	0.024	12.997	0.000
INTRUSIV	0.441	0.023	19.006	0.000
NONDISTR	0.742	0.037	20.106	0.000
POSREGAR	0.301	0.027	11.081	0.000
INTRUSIV	0.414	0.029	14.452	0.000
NONDISTR	0.790	0.033	23.862	0.000
POSREGAR	0.429	0.026	16.251	0.000
INTRUSIV	0.348	0.029	11.994	0.000
SPTSIO36	0.655	0.025	26.384	0.000
RSCSIO36	0.660	0.025	26.080	0.000
HOSTILIT	0.403	0.031	13.044	0.000
SPTSIO54	0.717	0.022	32.390	0.000
RSCSIO54	0.713	0.027	26.210	0.000
HOSTILIT	0.527	0.030	17.519	0.000
SPTSIO1S	0.680	0.027	25.320	0.000
RSCSIO1S	0.729	0.029	25.553	0.000
HOSTILIT	0.454	0.032	14.346	0.000
SPTSIOG3	0.845	0.023	37.194	0.000
RSCSIOG3	0.710	0.024	29.980	0.000
HOSTILIT	0.289	0.028	10.250	0.000
SPTSIOG5	0.903	0.017	51.942	0.000
RSCSIOG5	0.702	0.022	32.437	0.000
HOSTILIT	0.496	0.028	17.440	0.000
MRASIOX5	0.897	0.019	47.832	0.000
MWRSIOX5	0.432	0.028	15.638	0.000
MHISIOX5	0.788	0.019	41.214	0.000
SLFR_O1S	1.000	999.000	999.000	999.000
SLFR_OG3	1.000	999.000	999.000	999.000
SLFR_OG5	1.000	999.000	999.000	999.000
SLFRLCX5	1.000	999.000	999.000	999.000
WJAPSC1S	1.000	999.000	999.000	999.000
WJAPSCG3	1.000	999.000	999.000	999.000
WJAPSCG5	1.000	999.000	999.000	999.000
WJAPSCX5	1.000	999.000	999.000	999.000
WJPCSCG3	1.000	999.000	999.000	999.000
WJPCSCG5	1.000	999.000	999.000	999.000
WJPCSCX5	1.000	999.000	999.000	999.000
RVCSTM36	0.409	0.023	17.568	0.000
RELSTM36	0.190	0.022	8.547	0.000
PLSASC54	0.563	0.019	29.308	0.000
PLSESC54	0.520	0.021	25.009	0.000
CPINCC54	0.164	0.020	8.362	0.000
CPOMSC54	0.142	0.020	7.032	0.000
WJMSSC54	0.331	0.025	13.211	0.000

Latent Variable	Estimate	S.E.	Est./S.E.	Two-Tailed P-Value
MS_6M	1.000	999.000	999.000	999.000
MS_15M	1.000	999.000	999.000	999.000
MS_24M	1.000	999.000	999.000	999.000
MS_36M	1.000	999.000	999.000	999.000
MS_54M	1.000	999.000	999.000	999.000
MS_G1	1.000	999.000	999.000	999.000

MS_G3	1.000	999.000	999.000	999.000
MS_G5	1.000	999.000	999.000	999.000
MS_15	1.000	999.000	999.000	999.000
MS_RI	0.530	0.027	19.667	0.000
MS_15MR	0.009	0.007	1.189	0.234
MS_24MR	0.007	0.008	0.907	0.365
MS_36MR	0.062	0.025	2.464	0.014
MS_54MR	0.107	0.035	3.089	0.002
MS_G1R	0.102	0.036	2.853	0.004
MS_G3R	0.049	0.022	2.169	0.030
MS_G5R	0.017	0.014	1.260	0.208
MS_15R	0.026	0.015	1.733	0.083
SR	0.332	0.083	4.008	0.000
SLFR_G1R	0.037	0.014	2.723	0.006
SLFR_G3R	0.051	0.017	3.021	0.003
SLFR_G5R	0.073	0.019	3.761	0.000
SLFR_15R	0.040	0.026	1.537	0.124
READ	0.305	0.031	9.765	0.000
WJPCSCG3	0.014	0.014	1.032	0.302
WJPCSCG5	0.038	0.024	1.605	0.108
WJPCSCX5	0.117	0.099	1.184	0.237
MATH	0.311	0.027	11.667	0.000
WJAPSC1S	0.170	0.029	5.919	0.000
WJAPSCG3	0.093	0.034	2.765	0.006
WJAPSCG5	0.079	0.039	2.004	0.045
WJAPSCX5	0.131	0.071	1.841	0.066

MODEL MODIFICATION INDICES

NOTE: Modification indices for direct effects of observed dependent variables regressed on covariates may not be included. To include these, request MODINDICES (ALL).

Minimum M.I. value for printing the modification index 25.000

	M.I.	E.P.C.	Std E.P.C.	StdYX E.P.C.
**BY Statements**				
MS_15M BY INTRUSIV	29.854	0.227	0.233	0.185
MS_36M BY POSREGAR	37.809	0.217	0.219	0.187
MS_G5 BY MWRSIOX5	33.265	0.604	0.575	0.191
MS_G5 BY SLFRLCX5	35.600	-0.250	-0.238	-0.583
MS_RI BY POSREGAR	29.942	0.348	0.232	0.197
MS_RI BY INTRUSIV	28.207	0.415	0.276	0.219
MS_RI BY MWRSIOX5	29.550	0.938	0.624	0.207
MS_RI BY SLFRLCX5	35.600	-0.250	-0.167	-0.408
MS_6MR BY NONDISTR	54.880	0.705	0.756	0.598
MS_6MR BY POSREGAR	29.943	-0.348	-0.373	-0.318
MS_15MR BY NONDISTR	27.262	0.640	0.501	0.420
MS_24MR BY NONDISTR	34.627	0.600	0.553	0.432
MS_24MR BY INTRUSIV	28.207	-0.415	-0.383	-0.304
MS_15R BY MWRSIOX5	29.550	-0.938	-0.891	-0.296

SR BY SLFRLCX5	47.089	-1.075	-0.246	-0.603
READ BY SLFRLCX5	28.420	-0.012	-0.142	-0.346
READ BY WJPCSCX5	36.819	0.253	2.993	0.203
WJPCSCG3 BY WJAPSC1S	37.524	-0.624	-4.985	-0.293
WJPCSCG3 BY WJAPSCG3	67.253	0.399	3.185	0.214
WJPCSCG3 BY WJAPSCX5	28.237	-0.281	-2.240	-0.162
WJPCSCG3 BY WJPCSCG5	28.094	-0.640	-5.110	-0.388
WJPCSCG5 BY WJAPSCG3	46.647	1.297	7.576	0.509
WJPCSCX5 BY WJAPSCG5	26.988	0.559	4.918	0.366
WJPCSCX5 BY WJAPSCX5	49.380	0.530	4.659	0.337
MATH BY SLFRLCX5	31.927	-0.014	-0.154	-0.376
MATH BY WJPCSCX5	37.105	0.245	2.766	0.187
WJAPSC1S BY WJAPSCG3	26.425	-0.628	-7.170	-0.481
WJAPSCG3 BY WJPCSCG3	67.253	0.322	2.982	0.207
WJAPSCG5 BY WJPCSCG3	37.152	0.977	7.010	0.487
WJAPSCX5 BY WJPCSCG3	27.135	-0.317	-2.522	-0.175
WJAPSCX5 BY WJPCSCG5	57.405	0.912	7.263	0.551
WJAPSCX5 BY WJPCSCX5	49.380	0.656	5.225	0.354

ON/BY Statements

RVCSTM36 ON MS_24M /				
MS_24M BY RVCSTM36	28.447	5.393	6.134	0.387
RVCSTM36 ON MS_36M /				
MS_36M BY RVCSTM36	28.447	5.393	5.453	0.344
RVCSTM36 ON MS_RI /				
MS_RI BY RVCSTM36	28.447	5.393	3.588	0.227
RVCSTM36 ON SR /				
SR BY RVCSTM36	63.933	22.613	5.180	0.327
RVCSTM36 ON READ /				
READ BY RVCSTM36	68.226	0.310	3.672	0.232
RVCSTM36 ON MATH /				
MATH BY RVCSTM36	81.760	0.366	4.133	0.261
PLSASC54 ON READ /				
READ BY PLSASC54	27.525	0.223	2.642	0.133
PLSASC54 ON MATH /				
MATH BY PLSASC54	29.924	0.253	2.859	0.144
MS_RI ON MS_G1 /				
MS_G1 BY MS_RI	29.449	0.173	0.290	0.290
MS_RI ON MS_G1R /				
MS_G1R BY MS_RI	29.448	0.173	0.220	0.220
SLFR_G3R ON SR /				
SR BY SLFR_G3R	28.010	0.988	0.248	0.248
SLFR_G3R ON READ /				
READ BY SLFR_G3R	40.308	0.018	0.232	0.232
SLFR_G3R ON MATH /				
MATH BY SLFR_G3R	32.153	0.017	0.215	0.215
SLFR_G5R ON MS_15M /				
MS_15M BY SLFR_G5R	26.422	0.155	0.185	0.185
SLFR_G5R ON MS_G3 /				
MS_G3 BY SLFR_G5R	26.819	0.249	0.288	0.288
SLFR_G5R ON MS_RI /				
MS_RI BY SLFR_G5R	26.819	0.249	0.192	0.192
SLFR_15R ON MS_G5 /				

MS_G5   BY SLFR_15R   35.600   -0.250   -0.703   -0.703
SLFR_15R ON MS_RI   /
MS_RI   BY SLFR_15R   35.600   -0.250   -0.491   -0.491
SLFR_15R ON SR   /
SR   BY SLFR_15R   47.089   -1.075   -0.727   -0.727
SLFR_15R ON READ   /
READ   BY SLFR_15R   28.420   -0.012   -0.418   -0.418
SLFR_15R ON MATH   /
MATH   BY SLFR_15R   31.927   -0.014   -0.453   -0.453
WJPCSCG3 ON WJAPSCG3 /
WJAPSCG3 BY WJPCSCG3   67.253   0.322   0.373   0.373
WJPCSCG3 ON WJAPSCG5 /
WJAPSCG5 BY WJPCSCG3   35.646   1.040   0.935   0.935
WJPCSCG3 ON WJAPSCX5 /
WJAPSCX5 BY WJPCSCG3   26.654   -0.320   -0.319   -0.319
WJPCSCG5 ON MS_G3   /
MS_G3   BY WJPCSCG5   26.309   -2.118   -0.361   -0.361
WJPCSCG5 ON MS_RI   /
MS_RI   BY WJPCSCG5   26.309   -2.118   -0.241   -0.241
WJPCSCG5 ON READ   /
READ   BY WJPCSCG5   39.378   -0.190   -0.385   -0.385
WJPCSCG5 ON MATH   /
MATH   BY WJPCSCG5   36.520   -0.172   -0.332   -0.332
WJPCSCX5 ON READ   /
READ   BY WJPCSCX5   36.819   0.253   0.340   0.340
WJPCSCX5 ON MATH   /
MATH   BY WJPCSCX5   37.105   0.245   0.315   0.315
WJPCSCX5 ON WJAPSCX5 /
WJAPSCX5 BY WJPCSCX5   49.380   0.656   0.594   0.594
MATH   ON WJAPSC1S /
WJAPSC1S BY MATH   35.545   0.267   0.270   0.270
WJAPSCG3 ON WJPCSCG3 /
WJPCSCG3 BY WJAPSCG3   67.253   0.399   0.344   0.344
WJAPSCG3 ON WJPCSCG5 /
WJPCSCG5 BY WJAPSCG3   28.982   1.099   0.693   0.693
WJAPSCG5 ON WJPCSCX5 /
WJPCSCX5 BY WJAPSCG5   28.467   0.579   0.710   0.710
WJAPSCG5 ON WJAPSCX5 /
WJAPSCX5 BY WJAPSCG5   25.764   0.658   0.731   0.731
WJAPSCX5 ON WJPCSCG3 /
WJPCSCG3 BY WJAPSCX5   28.237   -0.281   -0.281   -0.281
WJAPSCX5 ON WJPCSCX5 /
WJPCSCX5 BY WJAPSCX5   49.380   0.530   0.585   0.585

ON Statements

MS_G1   ON CSEX_M01   26.934   -0.329   -0.294   -0.147
SLFR_G5R ON PLSESC54   25.271   0.007   0.008   0.168
READ   ON RELSTM36   28.703   0.107   0.009   0.132
READ   ON PLSASC54   30.161   0.090   0.008   0.152
READ   ON PLSESC54   32.256   0.092   0.008   0.155
READ   ON WJMSSC54   45.298   0.108   0.009   0.170
WJPCSCX5 ON PLSESC54   26.952   0.094   0.011   0.212
MATH   ON PLSASC54   31.558   0.094   0.008   0.166

WITH Statements

INTRUSIV WITH NONDISTR	32.145	0.551	0.551	5.932
INTRUSIV WITH POSREGAR	58.169	-0.299	-0.299	-0.302
POSREGAR WITH POSREGAR	27.665	0.150	0.150	0.159
INTRUSIV WITH INTRUSIV	52.172	0.223	0.223	0.225
INTRUSIV WITH POSREGAR	30.938	-0.199	-0.199	-0.211
POSREGAR WITH POSREGAR	34.194	0.171	0.171	0.193
POSREGAR WITH NONDISTR	45.123	0.414	0.414	0.771
INTRUSIV WITH INTRUSIV	49.963	0.233	0.233	0.235
INTRUSIV WITH POSREGAR	53.560	-0.274	-0.274	-0.294
SPTSIO54 WITH INTRUSIV	31.552	-0.158	-0.158	-0.227
SPTSIO1S WITH SPTSIO54	27.846	0.131	0.131	0.250
HOSTILIT WITH HOSTILIT	31.393	0.086	0.086	0.204
HOSTILIT WITH HOSTILIT	46.488	0.106	0.106	0.255
HOSTILIT WITH HOSTILIT	26.083	0.084	0.084	0.187
MHISIOX5 WITH MRASIOX5	28.424	-1.029	-1.029	-1.916
WJAPSCG3 WITH WJAPSC1S	33.058	-38.419	-38.419	999.000
WJPCSCG3 WITH WJAPSC1S	32.341	-34.627	-34.627	999.000
WJPCSCG3 WITH WJAPSCG3	68.356	25.263	25.263	999.000
WJPCSCG3 WITH WJAPSCX5	29.171	-17.582	-17.582	999.000
WJPCSCG5 WITH WJAPSCX5	54.662	52.115	52.115	999.000
WJPCSCX5 WITH WJAPSCX5	49.380	36.161	36.161	999.000
WJAPSCG3 WITH WJPCSCG3	67.253	25.061	0.358	0.358
WJAPSCG3 WITH MATH	26.532	-19.222	-0.233	-0.233
WJAPSCX5 WITH WJPCSCG3	29.260	-17.923	-0.305	-0.305
WJAPSCX5 WITH WJPCSCX5	49.380	36.161	0.590	0.590

Variances/Residual Variances

WJAPSC1S	31.755	182.111	182.111	0.629
WJAPSCG3	31.391	203.671	203.671	0.918
WJPCSCG5	34.643	96.175	96.175	0.554

DIAGRAM INFORMATION

Use View Diagram under the Diagram menu in the Mplus Editor to view the diagram.
If running Mplus from the Mplus Diagrammer, the diagram opens automatically.

Diagram output
c:\users\tuf22063\onedrive - temple university\foley & weinraub revision\r2\models\r2.5.1 ds - replication test - ra

Beginning Time: 17:49:14
Ending Time: 18:24:19
Elapsed Time: 00:35:05

MUTHEN & MUTHEN
3463 Stoner Ave.
Los Angeles, CA 90066

Tel: (310) 391-9971
Fax: (310) 391-8971
Web: www.StatModel.com
Support: Support@StatModel.com

Copyright (c) 1998-2023 Muthen & Muthen
