# Supplementary material for: On the Broader Significance of Maternal Sensitivity: Mothers’ Early and Later Sensitive Parenting Matter to Children's Language, Executive Function, Academics, and Self‐Reliance
Source: Dev Sci. 2024 Dec 16;28(1):e13594. doi: 10.1111/desc.13594 (PMC11647561; doi:10.1111/desc.13594)
Supplement: Supplementary file 6 — Supporting information [file DESC-28-e13594-s006.pdf]

Mplus VERSION 8.10
MUTHEN & MUTHEN
10/30/2024   6:44 PM

INPUT INSTRUCTIONS

```
  TITLE: Foley & Weinraub
  DATA: FILE IS 'matsens_DS_file_mplus.csv';
  VARIABLE: NAMES ARE ID CSEX_M01 CRACEM01 MEDUCM01 TEMP_M06 MDI15O15 RAWVPM24
    STDSCM36 SPTSIO36 RSCSIO36 HOSSIO36 RVCSTM36 RELSTM36 AVBTCO36 TOTBCO36
    PLSASC54 PLSESC54 WJMSSC54 CPINCC54 CPOMSC54 SLFR_O1S WJLWSC1S WJAPSC1S
    WJBRSCG3 WJBMSCG3 SLFR_OG3 SLFR_OG5 WJBRSCG5 WJBMSCG5 WJPCSCX5 WJAPSCX5
    SLFRLCX5 SPTSIO54 RSCSIO54 HOSSIO54 SPTSIO1S RSCSIO1S HOSSIO1S SPTSIOG3
    RSCSIOG3 HOSSIOG3 SPTSIOG5 RSCSIOG5 HOSSIOG5 MRASIOX5 MWRSIOX5 MHISIOX5
    VCRAWM15 VPRAWM15 Nondistress24m_7trans Posregard24m_7trans nondistress6m_7trans
    Posregard6m_7trans intrusiveness6m_reversed_7trans Nondistress15m_7trans
    Posregard15m_7trans intrusiveness15m_reversed_7trans Hostility36m_reversed
    Hostility54m_reversed HostilityG1_reversed HostilityG3_reversed
    HostilityG5_reversed Intrusiveness_REVER_Trans24m WJPCSCG3 WJAPSCG3
    WJPCSCG5 WJAPSCG5 INCNTM01 MADEPM01;
         USEVARIABLES ARE nondistress6m_7trans Posregard6m_7trans
    intrusiveness6m_reversed_7trans Nondistress15m_7trans Posregard15m_7trans
    intrusiveness15m_reversed_7trans Nondistress24m_7trans Posregard24m_7trans
    Intrusiveness_REVER_Trans24m SPTSIO36 RSCSIO36 Hostility36m_reversed
    SPTSIO54 RSCSIO54 Hostility54m_reversed SPTSIO1S RSCSIO1S HostilityG1_reversed
    SPTSIOG3 RSCSIOG3 HostilityG3_reversed SPTSIOG5 RSCSIOG5 HostilityG5_reversed
    MRASIOX5 MWRSIOX5 MHISIOX5 SLFR_O1S SLFR_OG3 SLFR_OG5 SLFRLCX5 WJAPSC1S
    WJAPSCG3 WJAPSCG5 WJAPSCX5 WJPCSCG3 WJPCSCG5 WJPCSCX5 RVCSTM36 RELSTM36
    PLSASC54 PLSESC54 CPINCC54 CPOMSC54 WJMSSC54 CSEX_M01 CRACEM01 MEDUCM01
    TEMP_M06 MDI15O15 INCNTM01 MADEPM01;
         MISSING IS .;
         USEOBSERVATIONS ARE (CSEX_M01 == 1 OR CSEX_M01 == 2);

  ANALYSIS: ESTIMATOR IS MLR;
         MODEL IS NOCOVARIANCES;
  MODEL:
  MS_6m BY nondistress6m_7trans
         Posregard6m_7trans !(a)
         intrusiveness6m_reversed_7trans; ! (b);
                MS_15m BY Nondistress15m_7trans
                       Posregard15m_7trans !(a)
                    intrusiveness15m_reversed_7trans; ! (b);
                MS_24m BY Nondistress24m_7trans
                       Posregard24m_7trans! (a)
                    Intrusiveness_REVER_Trans24m; ! (b);
                MS_36m BY SPTSIO36
                        RSCSIO36! (a)
                        Hostility36m_reversed; ! (b);
                MS_54m BY SPTSIO54
                        RSCSIO54! (a)
                        Hostility54m_reversed; ! (b);
                MS_G1 BY SPTSIO1S
                       RSCSIO1S! (a)
```

```
         HostilityG1_reversed; ! (b);
  MS_G3 BY SPTSIOG3
        RSCSIOG3! (a)
        HostilityG3_reversed; ! (b);
  MS_G5 BY SPTSIOG5
        RSCSIOG5! (a)
        HostilityG5_reversed; ! (b);
  MS_15 BY MRASIOX5
        MWRSIOX5! (a)
        MHISIOX5; ! (b);

  MS_RI BY MS_6m@1 MS_15m@1 MS_24m@1 MS_36m@1 MS_54m@1 MS_G1@1
     MS_G3@1 MS_G5@1 MS_15@1;

  MS_6m@0; MS_15m@0; MS_24m@0; MS_36m@0; MS_54m@0; MS_G1@0;
  MS_G3@0; MS_G5@0; MS_15@0;;

  MS_6mr BY MS_6m@1;
 MS_15mr BY MS_15m@1;
  MS_24mr BY MS_24m@1;
  MS_36mr BY MS_36m@1;
  MS_54mr BY MS_54m@1;
  MS_G1r BY MS_G1@1;
  MS_G3r BY MS_G3@1;
  MS_G5r BY MS_G5@1;
  MS_15r BY MS_15@1;

 sr BY SLFR_O1S@1 SLFR_OG3@1 SLFR_OG5@1 SLFRLCX5@1;

SLFR_O1S@0; SLFR_OG3@0; SLFR_OG5@0; SLFRLCX5@0

 slfr_g1r BY SLFR_O1S@1;
 slfr_g3r BY SLFR_OG3@1;
 slfr_g5r BY SLFR_OG5@1;
 slfr_15r BY SLFRLCX5@1;

  read BY WJPCSCG3@1 WJPCSCG5@1 WJPCSCX5@1;

  WJPCSCG3@0; WJPCSCG5@0; WJPCSCX5@0;

  WJPCSCG3_r BY WJPCSCG3@1;
  WJPCSCG5_r BY WJPCSCG5@1;
  WJPCSCX5_r BY WJPCSCX5@1;

  math BY WJAPSC1S@1 WJAPSCG3@1 WJAPSCG5@1 WJAPSCX5@1;


  WJAPSC1S@0; WJAPSCG3@0; WJAPSCG5@0; WJAPSCX5@0;

  WJAPSC1S_r BY WJAPSC1S@1;
  WJAPSCG3_r BY WJAPSCG3@1;
  WJAPSCG5_r BY WJAPSCG5@1;
  WJAPSCX5_r BY WJAPSCX5@1;
```

```
        MS_RI WITH sr math read;
        sr WITH math read;
        read WITH math;

        MS_RI sr math read ON
          CSEX_M01 CRACEM01 MEDUCM01 TEMP_M06 MDI15O15 INCNTM01 MADEPM01;

        PLSASC54 PLSESC54 CPINCC54 CPOMSC54 WJMSSC54 RVCSTM36 RELSTM36 ON
            CSEX_M01 CRACEM01 MEDUCM01 TEMP_M06 MDI15O15 INCNTM01 MADEPM01;

! Lags

MS_15mr ON MS_6mr;
! MS_24mr ON MS_15mr;
MS_36mr ON MS_24mr;

MS_54mr ON MS_36mr; !RVCSTM36 RELSTM36;
PLSASC54 ON RVCSTM36 RELSTM36; !MS_36mr
PLSESC54 ON RVCSTM36 RELSTM36; !MS_36mr
CPINCC54 ON RVCSTM36 RELSTM36; !MS_36mr
CPOMSC54 ON RVCSTM36 RELSTM36; !MS_36mr
WJMSSC54 ON RVCSTM36 RELSTM36; !MS_36mr

MS_G1r ON MS_54mr PLSASC54;! PLSESC54 CPINCC54 CPOMSC54 WJMSSC54;
slfr_g1r ON CPOMSC54; !PLSASC54 PLSESC54 CPINCC54 WJMSSC54; !MS_54mr
WJAPSC1S_r ON PLSASC54 PLSESC54 CPOMSC54 WJMSSC54; !MS_54mr CPINCC54

MS_G3r ON MS_G1r;! slfr_g1r; !WJAPSC1S_r
slfr_g3r ON slfr_g1r WJAPSC1S_r; !MS_G1r
WJPCSCG3_r ON WJAPSC1S_r; !MS_G1r slfr_g1r
WJAPSCG3_r ON WJAPSC1S_r; !MS_G1r slfr_g1r

MS_G5r ON MS_G3r; !slfr_g3r WJPCSCG3_r WJAPSCG3_r
slfr_g5r ON slfr_g3r WJPCSCG3_r; !MS_G3r  WJAPSCG3_r
WJPCSCG5_r ON WJAPSCG3_r WJPCSCG3_r; !MS_G3r slfr_g3r
WJAPSCG5_r ON WJAPSCG3_r WJPCSCG3_r; !MS_G3r slfr_g3r

MS_15r ON MS_G5r; !slfr_g5r WJPCSCG5_r WJAPSCG5_r
slfr_15r ON slfr_g5r WJAPSCG5_r; !MS_G5r  WJPCSCG5_r
WJPCSCX5_r ON slfr_g5r WJPCSCG5_r WJAPSCG5_r; !MS_G5r ;
WJAPSCX5_r ON slfr_g5r WJPCSCG5_r WJAPSCG5_r; !MS_G5r ;

! 36 Month Covariates

RVCSTM36 RELSTM36 ON MS_24mr;

! Covariances

MS_54mr PLSASC54 PLSESC54 CPINCC54 CPOMSC54 WJMSSC54 WITH
  MS_54mr PLSASC54 PLSESC54 CPINCC54 CPOMSC54 WJMSSC54;

MS_36mr RVCSTM36 RELSTM36 WITH MS_36mr RVCSTM36 RELSTM36;

CSEX_M01 CRACEM01 MEDUCM01 TEMP_M06 MDI15O15 INCNTM01 MADEPM01 WITH
```

```
    CSEX_M01 CRACEM01 MEDUCM01 TEMP_M06 MDI15O15 INCNTM01 MADEPM01;

MODEL INDIRECT:

MS_15r IND MS_24mr;
slfr_15r IND MS_24mr;
WJPCSCX5_r IND MS_24mr;
WJAPSCX5_r IND MS_24mr;

MS_G5r IND MS_24mr;
slfr_g5r IND MS_24mr;
WJPCSCG5_r IND MS_24mr;
WJAPSCG5_r IND MS_24mr;

MS_G3r IND MS_24mr;
slfr_g3r IND MS_24mr;
WJPCSCG3_r IND MS_24mr;
WJAPSCG3_r IND MS_24mr;

MS_G1r IND MS_24mr;
slfr_g1r IND MS_24mr;
WJAPSC1S_r IND MS_24mr;

MS_54mr IND MS_24mr;
PLSASC54 IND MS_24mr;
PLSESC54 IND MS_24mr;
CPINCC54 IND MS_24mr;
CPOMSC54 IND MS_24mr;
WJMSSC54 IND MS_24mr;

MS_15r IND MS_54mr;
slfr_15r IND MS_54mr;
WJPCSCX5_r IND MS_54mr;
WJAPSCX5_r IND MS_54mr;

MS_G5r IND MS_54mr;
slfr_g5r IND MS_54mr;
WJPCSCG5_r IND MS_54mr;
WJAPSCG5_r IND MS_54mr;

MS_G3r IND MS_54mr;
slfr_g3r IND MS_54mr;
WJPCSCG3_r IND MS_54mr;
WJAPSCG3_r IND MS_54mr;

MS_G1r IND MS_54mr;
slfr_g1r IND MS_54mr;
WJAPSC1S_r IND MS_54mr;

MS_15r IND PLSASC54;
slfr_15r IND PLSASC54;
WJPCSCX5_r IND PLSASC54;
WJAPSCX5_r IND PLSASC54;
```

```
MS_G5r IND PLSASC54;
slfr_g5r IND PLSASC54;
WJPCSCG5_r IND PLSASC54;
WJAPSCG5_r IND PLSASC54;

MS_G3r IND PLSASC54;
slfr_g3r IND PLSASC54;
WJPCSCG3_r IND PLSASC54;
WJAPSCG3_r IND PLSASC54;

MS_G1r IND PLSASC54;
slfr_g1r IND PLSASC54;
WJAPSC1S_r IND PLSASC54;

MS_15r IND PLSESC54;
slfr_15r IND PLSESC54;
WJPCSCX5_r IND PLSESC54;
WJAPSCX5_r IND PLSESC54;

MS_G5r IND PLSESC54;
slfr_g5r IND PLSESC54;
WJPCSCG5_r IND PLSESC54;
WJAPSCG5_r IND PLSESC54;

MS_G3r IND PLSESC54;
slfr_g3r IND PLSESC54;
WJPCSCG3_r IND PLSESC54;
WJAPSCG3_r IND PLSESC54;

MS_G1r IND PLSESC54;
slfr_g1r IND PLSESC54;
WJAPSC1S_r IND PLSESC54;

MS_15r IND CPINCC54;
slfr_15r IND CPINCC54;
WJPCSCX5_r IND CPINCC54;
WJAPSCX5_r IND CPINCC54;

MS_G5r IND CPINCC54;
slfr_g5r IND CPINCC54;
WJPCSCG5_r IND CPINCC54;
WJAPSCG5_r IND CPINCC54;

MS_G3r IND CPINCC54;
slfr_g3r IND CPINCC54;
WJPCSCG3_r IND CPINCC54;
WJAPSCG3_r IND CPINCC54;

MS_G1r IND CPINCC54;
slfr_g1r IND CPINCC54;
WJAPSC1S_r IND CPINCC54;

MS_15r IND CPOMSC54;
slfr_15r IND CPOMSC54;
```

```
WJPCSCX5_r IND CPOMSC54;
WJAPSCX5_r IND CPOMSC54;

MS_G5r IND CPOMSC54;
slfr_g5r IND CPOMSC54;
WJPCSCG5_r IND CPOMSC54;
WJAPSCG5_r IND CPOMSC54;

MS_G3r IND CPOMSC54;
slfr_g3r IND CPOMSC54;
WJPCSCG3_r IND CPOMSC54;
WJAPSCG3_r IND CPOMSC54;

MS_G1r IND CPOMSC54;
slfr_g1r IND CPOMSC54;
WJAPSC1S_r IND CPOMSC54;

MS_15r IND WJMSSC54;
slfr_15r IND WJMSSC54;
WJPCSCX5_r IND WJMSSC54;
WJAPSCX5_r IND WJMSSC54;

MS_G5r IND WJMSSC54;
slfr_g5r IND WJMSSC54;
WJPCSCG5_r IND WJMSSC54;
WJAPSCG5_r IND WJMSSC54;

MS_G3r IND WJMSSC54;
slfr_g3r IND WJMSSC54;
WJPCSCG3_r IND WJMSSC54;
WJAPSCG3_r IND WJMSSC54;

MS_G1r IND WJMSSC54;
slfr_g1r IND WJMSSC54;
WJAPSC1S_r IND WJMSSC54;

MS_15r IND RVCSTM36;
slfr_15r IND RVCSTM36;
WJPCSCX5_r IND RVCSTM36;
WJAPSCX5_r IND RVCSTM36;

MS_G5r IND RVCSTM36;
slfr_g5r IND RVCSTM36;
WJPCSCG5_r IND RVCSTM36;
WJAPSCG5_r IND RVCSTM36;

MS_G3r IND RVCSTM36;
slfr_g3r IND RVCSTM36;
WJPCSCG3_r IND RVCSTM36;
WJAPSCG3_r IND RVCSTM36;

MS_G1r IND RVCSTM36;
slfr_g1r IND RVCSTM36;
WJAPSC1S_r IND RVCSTM36;
```

```
  MS_15r IND RELSTM36;
  slfr_15r IND RELSTM36;
  WJPCSCX5_r IND RELSTM36;
  WJAPSCX5_r IND RELSTM36;

  MS_G5r IND RELSTM36;
  slfr_g5r IND RELSTM36;
  WJPCSCG5_r IND RELSTM36;
  WJAPSCG5_r IND RELSTM36;

  MS_G3r IND RELSTM36;
  slfr_g3r IND RELSTM36;
  WJPCSCG3_r IND RELSTM36;
  WJAPSCG3_r IND RELSTM36;

  MS_G1r IND RELSTM36;
  slfr_g1r IND RELSTM36;
  WJAPSC1S_r IND RELSTM36;

  OUTPUT: sampstat stdyx modindices(25);
  plot: type is plot1 plot2 plot3;



*** WARNING in VARIABLE command
  Note that only the first 8 characters of variable names are used in the output.
  Shorten variable names to avoid any confusion.
*** WARNING in PLOT command
  Note that only the first 8 characters of variable names are used in plots.
  If variable names are not unique within the first 8 characters, problems
  may occur.
   2 WARNING(S) FOUND IN THE INPUT INSTRUCTIONS



Foley & Weinraub

SUMMARY OF ANALYSIS

Number of groups                                                 1
Number of observations                                        1364

Number of dependent variables                                   45
Number of independent variables                                  7
Number of continuous latent variables                           33

Observed dependent variables

  Continuous
   NONDISTRES  POSREGARD6  INTRUSIVEN  NONDISTRES  POSREGARD1  INTRUSIVEN
   NONDISTRES  POSREGARD2  INTRUSIVEN  SPTSIO36    RSCSIO36    HOSTILITY3
   SPTSIO54    RSCSIO54    HOSTILITY5  SPTSIO1S    RSCSIO1S    HOSTILITYG
   SPTSIOG3    RSCSIOG3    HOSTILITYG  SPTSIOG5    RSCSIOG5    HOSTILITYG
```

MRASIOX5   MWRSIOX5   MHISIOX5   SLFR_O1S   SLFR_OG3   SLFR_OG5
SLFRLCX5   WJAPSC1S   WJAPSCG3   WJAPSCG5   WJAPSCX5   WJPCSCG3
WJPCSCG5   WJPCSCX5   RVCSTM36   RELSTM36   PLSASC54   PLSESC54
CPINCC54   CPOMSC54   WJMSSC54

Observed independent variables
CSEX_M01   CRACEM01   MEDUCM01   TEMP_M06   MDI15O15   INCNTM01
MADEPM01

Continuous latent variables
MS_6M   MS_15M   MS_24M   MS_36M   MS_54M   MS_G1
MS_G3   MS_G5   MS_15   MS_RI   MS_6MR   MS_15MR
MS_24MR   MS_36MR   MS_54MR   MS_G1R   MS_G3R   MS_G5R
MS_15R   SR   SLFR_G1R   SLFR_G3R   SLFR_G5R   SLFR_15R
READ   WJPCSCG3   WJPCSCG5   WJPCSCX5   MATH   WJAPSC1S
WJAPSCG3   WJAPSCG5   WJAPSCX5

Estimator   MLR
Information matrix   OBSERVED
Maximum number of iterations   1000
Convergence criterion   0.500D-04
Maximum number of steepest descent iterations   20
Maximum number of iterations for H1   2000
Convergence criterion for H1   0.100D-03

Input data file(s)
matsens_DS_file_mplus.csv

Input data format FREE

SUMMARY OF DATA

Number of missing data patterns   422

COVARIANCE COVERAGE OF DATA

Minimum covariance coverage value   0.100

PROPORTION OF DATA PRESENT

Covariance Coverage

	NONDISTR	POSREGAR	INTRUSIV	NONDISTR	POSREGAR
NONDISTR	0.933				
POSREGAR	0.933	0.933			
INTRUSIV	0.933	0.933	0.933		
NONDISTR	0.890	0.890	0.890	0.909	
POSREGAR	0.890	0.890	0.890	0.909	0.909
INTRUSIV	0.890	0.890	0.890	0.909	0.909

NONDISTR	0.840	0.840	0.840	0.848	0.848
POSREGAR	0.840	0.840	0.840	0.848	0.848
INTRUSIV	0.840	0.840	0.840	0.848	0.848
SPTSIO36	0.831	0.831	0.831	0.838	0.838
RSCSIO36	0.831	0.831	0.831	0.838	0.838
HOSTILIT	0.831	0.831	0.831	0.838	0.838
SPTSIO54	0.746	0.746	0.746	0.753	0.753
RSCSIO54	0.746	0.746	0.746	0.753	0.753
HOSTILIT	0.746	0.746	0.746	0.753	0.753
SPTSIO1S	0.724	0.724	0.724	0.726	0.726
RSCSIO1S	0.724	0.724	0.724	0.726	0.726
HOSTILIT	0.724	0.724	0.724	0.726	0.726
SPTSIOG3	0.701	0.701	0.701	0.701	0.701
RSCSIOG3	0.701	0.701	0.701	0.701	0.701
HOSTILIT	0.701	0.701	0.701	0.701	0.701
SPTSIOG5	0.664	0.664	0.664	0.665	0.665
RSCSIOG5	0.664	0.664	0.664	0.665	0.665
HOSTILIT	0.664	0.664	0.664	0.665	0.665
MRASIOX5	0.641	0.641	0.641	0.639	0.639
MWRSIOX5	0.640	0.640	0.640	0.638	0.638
MHISIOX5	0.641	0.641	0.641	0.639	0.639
SLFR_O1S	0.696	0.696	0.696	0.697	0.697
SLFR_OG3	0.695	0.695	0.695	0.695	0.695
SLFR_OG5	0.683	0.683	0.683	0.684	0.684
SLFRLCX5	0.684	0.684	0.684	0.683	0.683
WJAPSC1S	0.736	0.736	0.736	0.738	0.738
WJAPSCG3	0.717	0.717	0.717	0.724	0.724
WJAPSCG5	0.700	0.700	0.700	0.709	0.709
WJAPSCX5	0.635	0.635	0.635	0.635	0.635
WJPCSCG3	0.716	0.716	0.716	0.722	0.722
WJPCSCG5	0.699	0.699	0.699	0.708	0.708
WJPCSCX5	0.636	0.636	0.636	0.636	0.636
RVCSTM36	0.830	0.830	0.830	0.837	0.837
RELSTM36	0.811	0.811	0.811	0.817	0.817
PLSASC54	0.763	0.763	0.763	0.768	0.768
PLSESC54	0.757	0.757	0.757	0.761	0.761
CPINCC54	0.717	0.717	0.717	0.723	0.723
CPOMSC54	0.717	0.717	0.717	0.723	0.723
WJMSSC54	0.754	0.754	0.754	0.761	0.761
CSEX_M01	0.933	0.933	0.933	0.909	0.909
CRACEM01	0.933	0.933	0.933	0.909	0.909
MEDUCM01	0.933	0.933	0.933	0.909	0.909
TEMP_M06	0.932	0.932	0.932	0.895	0.895
MDI15O15	0.850	0.850	0.850	0.860	0.860
INCNTM01	0.837	0.837	0.837	0.849	0.849
MADEPM01	0.896	0.896	0.896	0.908	0.908

Covariance Coverage

	INTRUSIV	NONDISTR	POSREGAR	INTRUSIV	SPTSIO36
INTRUSIV	0.909				
NONDISTR	0.848	0.859			
POSREGAR	0.848	0.859	0.859		

INTRUSIV	0.848	0.859	0.859	0.859	
SPTSIO36	0.838	0.820	0.820	0.820	0.851
RSCSIO36	0.838	0.820	0.820	0.820	0.851
HOSTILIT	0.838	0.820	0.820	0.820	0.851
SPTSIO54	0.753	0.738	0.738	0.738	0.743
RSCSIO54	0.753	0.738	0.738	0.738	0.743
HOSTILIT	0.753	0.738	0.738	0.738	0.743
SPTSIO1S	0.726	0.713	0.713	0.713	0.715
RSCSIO1S	0.726	0.713	0.713	0.713	0.715
HOSTILIT	0.726	0.713	0.713	0.713	0.715
SPTSIOG3	0.701	0.678	0.678	0.678	0.680
RSCSIOG3	0.701	0.678	0.678	0.678	0.680
HOSTILIT	0.701	0.678	0.678	0.678	0.680
SPTSIOG5	0.665	0.642	0.642	0.642	0.641
RSCSIOG5	0.665	0.642	0.642	0.642	0.641
HOSTILIT	0.665	0.642	0.642	0.642	0.641
MRASIOX5	0.639	0.621	0.621	0.621	0.622
MWRSIOX5	0.638	0.620	0.620	0.620	0.621
MHISIOX5	0.639	0.621	0.621	0.621	0.622
SLFR_O1S	0.697	0.680	0.680	0.680	0.685
SLFR_OG3	0.695	0.672	0.672	0.672	0.673
SLFR_OG5	0.684	0.661	0.661	0.661	0.658
SLFRLCX5	0.683	0.662	0.662	0.662	0.661
WJAPSC1S	0.738	0.725	0.725	0.725	0.727
WJAPSCG3	0.724	0.699	0.699	0.699	0.696
WJAPSCG5	0.709	0.682	0.682	0.682	0.680
WJAPSCX5	0.635	0.620	0.620	0.620	0.617
WJPCSCG3	0.722	0.697	0.697	0.697	0.694
WJPCSCG5	0.708	0.681	0.681	0.681	0.680
WJPCSCX5	0.636	0.621	0.621	0.621	0.617
RVCSTM36	0.837	0.820	0.820	0.820	0.842
RELSTM36	0.817	0.801	0.801	0.801	0.822
PLSASC54	0.768	0.751	0.751	0.751	0.755
PLSESC54	0.761	0.745	0.745	0.745	0.749
CPINCC54	0.723	0.710	0.710	0.710	0.716
CPOMSC54	0.723	0.710	0.710	0.710	0.716
WJMSSC54	0.761	0.746	0.746	0.746	0.750
CSEX_M01	0.909	0.859	0.859	0.859	0.851
CRACEM01	0.909	0.859	0.859	0.859	0.851
MEDUCM01	0.909	0.859	0.859	0.859	0.851
TEMP_M06	0.895	0.845	0.845	0.845	0.837
MDI15O15	0.860	0.821	0.821	0.821	0.809
INCNTM01	0.849	0.800	0.800	0.800	0.793
MADEPM01	0.908	0.852	0.852	0.852	0.842

Covariance Coverage

	RSCSIO36	HOSTILIT	SPTSIO54	RSCSIO54	HOSTILIT
RSCSIO36	0.851				
HOSTILIT	0.851	0.851			
SPTSIO54	0.743	0.743	0.762		
RSCSIO54	0.743	0.743	0.762	0.762	
HOSTILIT	0.743	0.743	0.762	0.762	0.762

SPTSIO1S	0.715	0.715	0.705	0.705	0.705
RSCSIO1S	0.715	0.715	0.705	0.705	0.705
HOSTILIT	0.715	0.715	0.705	0.705	0.705
SPTSIOG3	0.680	0.680	0.658	0.658	0.658
RSCSIOG3	0.680	0.680	0.658	0.658	0.658
HOSTILIT	0.680	0.680	0.658	0.658	0.658
SPTSIOG5	0.641	0.641	0.617	0.617	0.617
RSCSIOG5	0.641	0.641	0.617	0.617	0.617
HOSTILIT	0.641	0.641	0.617	0.617	0.617
MRASIOX5	0.622	0.622	0.599	0.599	0.599
MWRSIOX5	0.621	0.621	0.598	0.598	0.598
MHISIOX5	0.622	0.622	0.599	0.599	0.599
SLFR_O1S	0.685	0.685	0.673	0.673	0.673
SLFR_OG3	0.673	0.673	0.651	0.651	0.651
SLFR_OG5	0.658	0.658	0.638	0.638	0.638
SLFRLCX5	0.661	0.661	0.639	0.639	0.639
WJAPSC1S	0.727	0.727	0.713	0.713	0.713
WJAPSCG3	0.696	0.696	0.677	0.677	0.677
WJAPSCG5	0.680	0.680	0.655	0.655	0.655
WJAPSCX5	0.617	0.617	0.603	0.603	0.603
WJPCSCG3	0.694	0.694	0.675	0.675	0.675
WJPCSCG5	0.680	0.680	0.654	0.654	0.654
WJPCSCX5	0.617	0.617	0.603	0.603	0.603
RVCSTM36	0.842	0.842	0.740	0.740	0.740
RELSTM36	0.822	0.822	0.724	0.724	0.724
PLSASC54	0.755	0.755	0.751	0.751	0.751
PLSESC54	0.749	0.749	0.745	0.745	0.745
CPINCC54	0.716	0.716	0.723	0.723	0.723
CPOMSC54	0.716	0.716	0.723	0.723	0.723
WJMSSC54	0.750	0.750	0.757	0.757	0.757
CSEX_M01	0.851	0.851	0.762	0.762	0.762
CRACEM01	0.851	0.851	0.762	0.762	0.762
MEDUCM01	0.851	0.851	0.762	0.762	0.762
TEMP_M06	0.837	0.837	0.750	0.750	0.750
MDI15O15	0.809	0.809	0.728	0.728	0.728
INCNTM01	0.793	0.793	0.715	0.715	0.715
MADEPM01	0.842	0.842	0.757	0.757	0.757

Covariance Coverage

	SPTSIO1S	RSCSIO1S	HOSTILIT	SPTSIOG3	RSCSIOG3
SPTSIO1S	0.736				
RSCSIO1S	0.736	0.736			
HOSTILIT	0.736	0.736	0.736		
SPTSIOG3	0.655	0.655	0.655	0.720	
RSCSIOG3	0.655	0.655	0.655	0.720	0.720
HOSTILIT	0.655	0.655	0.655	0.720	0.720
SPTSIOG5	0.614	0.614	0.614	0.629	0.629
RSCSIOG5	0.614	0.614	0.614	0.629	0.629
HOSTILIT	0.614	0.614	0.614	0.629	0.629
MRASIOX5	0.595	0.595	0.595	0.604	0.604
MWRSIOX5	0.595	0.595	0.595	0.603	0.603
MHISIOX5	0.595	0.595	0.595	0.604	0.604

SLFR_O1S	0.677	0.677	0.677	0.625	0.625
SLFR_OG3	0.645	0.645	0.645	0.652	0.652
SLFR_OG5	0.625	0.625	0.625	0.633	0.633
SLFRLCX5	0.634	0.634	0.634	0.642	0.642
WJAPSC1S	0.732	0.732	0.732	0.666	0.666
WJAPSCG3	0.669	0.669	0.669	0.680	0.680
WJAPSCG5	0.648	0.648	0.648	0.652	0.652
WJAPSCX5	0.595	0.595	0.595	0.600	0.600
WJPCSCG3	0.667	0.667	0.667	0.679	0.679
WJPCSCG5	0.648	0.648	0.648	0.650	0.650
WJPCSCX5	0.596	0.596	0.596	0.600	0.600
RVCSTM36	0.713	0.713	0.713	0.675	0.675
RELSTM36	0.696	0.696	0.696	0.657	0.657
PLSASC54	0.711	0.711	0.711	0.667	0.667
PLSESC54	0.705	0.705	0.705	0.662	0.662
CPINCC54	0.677	0.677	0.677	0.635	0.635
CPOMSC54	0.677	0.677	0.677	0.635	0.635
WJMSSC54	0.708	0.708	0.708	0.663	0.663
CSEX_M01	0.736	0.736	0.736	0.720	0.720
CRACEM01	0.736	0.736	0.736	0.720	0.720
MEDUCM01	0.736	0.736	0.736	0.720	0.720
TEMP_M06	0.727	0.727	0.727	0.705	0.705
MDI15O15	0.703	0.703	0.703	0.674	0.674
INCNTM01	0.688	0.688	0.688	0.666	0.666
MADEPM01	0.730	0.730	0.730	0.705	0.705

Covariance Coverage

	HOSTILIT	SPTSIOG5	RSCSIOG5	HOSTILIT	MRASIOX5
HOSTILIT	0.720				
SPTSIOG5	0.629	0.681			
RSCSIOG5	0.629	0.681	0.681		
HOSTILIT	0.629	0.681	0.681	0.681	
MRASIOX5	0.604	0.587	0.587	0.587	0.658
MWRSIOX5	0.603	0.587	0.587	0.587	0.658
MHISIOX5	0.604	0.587	0.587	0.587	0.658
SLFR_O1S	0.625	0.586	0.586	0.586	0.567
SLFR_OG3	0.652	0.608	0.608	0.608	0.589
SLFR_OG5	0.633	0.617	0.617	0.617	0.595
SLFRLCX5	0.642	0.625	0.625	0.625	0.641
WJAPSC1S	0.666	0.625	0.625	0.625	0.605
WJAPSCG3	0.680	0.632	0.632	0.632	0.603
WJAPSCG5	0.652	0.654	0.654	0.654	0.614
WJAPSCX5	0.600	0.583	0.583	0.583	0.608
WJPCSCG3	0.679	0.630	0.630	0.630	0.602
WJPCSCG5	0.650	0.652	0.652	0.652	0.612
WJPCSCX5	0.600	0.584	0.584	0.584	0.609
RVCSTM36	0.675	0.639	0.639	0.639	0.622
RELSTM36	0.657	0.625	0.625	0.625	0.606
PLSASC54	0.667	0.625	0.625	0.625	0.607
PLSESC54	0.662	0.621	0.621	0.621	0.604
CPINCC54	0.635	0.592	0.592	0.592	0.578
CPOMSC54	0.635	0.592	0.592	0.592	0.578

WJMSSC54	0.663	0.623	0.623	0.623	0.604
CSEX_M01	0.720	0.681	0.681	0.681	0.658
CRACEM01	0.720	0.681	0.681	0.681	0.658
MEDUCM01	0.720	0.681	0.681	0.681	0.658
TEMP_M06	0.705	0.668	0.668	0.668	0.644
MDI15O15	0.674	0.641	0.641	0.641	0.614
INCNTM01	0.666	0.622	0.622	0.622	0.604
MADEPM01	0.705	0.667	0.667	0.667	0.642

Covariance Coverage

	MWRSIOX5	MHISIOX5	SLFR_O1S	SLFR_OG3	SLFR_OG5
MWRSIOX5	0.658				
MHISIOX5	0.658	0.658			
SLFR_O1S	0.566	0.567	0.708		
SLFR_OG3	0.588	0.589	0.641	0.712	
SLFR_OG5	0.594	0.595	0.615	0.643	0.700
SLFRLCX5	0.641	0.641	0.603	0.625	0.634
WJAPSC1S	0.604	0.605	0.689	0.658	0.639
WJAPSCG3	0.602	0.603	0.644	0.665	0.647
WJAPSCG5	0.613	0.614	0.617	0.636	0.646
WJAPSCX5	0.607	0.608	0.565	0.583	0.587
WJPCSCG3	0.601	0.602	0.642	0.663	0.646
WJPCSCG5	0.611	0.612	0.617	0.636	0.644
WJPCSCX5	0.608	0.609	0.565	0.583	0.587
RVCSTM36	0.621	0.622	0.683	0.671	0.655
RELSTM36	0.606	0.606	0.667	0.654	0.640
PLSASC54	0.606	0.607	0.687	0.663	0.648
PLSESC54	0.603	0.604	0.682	0.657	0.643
CPINCC54	0.578	0.578	0.652	0.627	0.612
CPOMSC54	0.578	0.578	0.652	0.627	0.612
WJMSSC54	0.603	0.604	0.681	0.656	0.642
CSEX_M01	0.658	0.658	0.708	0.712	0.700
CRACEM01	0.658	0.658	0.708	0.712	0.700
MEDUCM01	0.658	0.658	0.708	0.712	0.700
TEMP_M06	0.644	0.644	0.699	0.697	0.685
MDI15O15	0.614	0.614	0.672	0.668	0.655
INCNTM01	0.603	0.604	0.662	0.659	0.647
MADEPM01	0.641	0.642	0.702	0.699	0.687

Covariance Coverage

	SLFRLCX5	WJAPSC1S	WJAPSCG3	WJAPSCG5	WJAPSCX5
SLFRLCX5	0.702				
WJAPSC1S	0.645	0.750			
WJAPSCG3	0.646	0.680	0.729		
WJAPSCG5	0.655	0.659	0.671	0.713	
WJAPSCX5	0.650	0.606	0.606	0.613	0.650
WJPCSCG3	0.645	0.679	0.727	0.669	0.605
WJPCSCG5	0.653	0.658	0.669	0.712	0.611
WJPCSCX5	0.650	0.607	0.606	0.614	0.647
RVCSTM36	0.660	0.725	0.693	0.677	0.616

RELSTM36	0.644	0.710	0.677	0.661	0.600
PLSASC54	0.646	0.727	0.683	0.663	0.609
PLSESC54	0.643	0.721	0.677	0.658	0.606
CPINCC54	0.617	0.689	0.652	0.630	0.583
CPOMSC54	0.617	0.689	0.652	0.630	0.583
WJMSSC54	0.644	0.721	0.681	0.661	0.607
CSEX_M01	0.702	0.750	0.729	0.713	0.650
CRACEM01	0.702	0.750	0.729	0.713	0.650
MEDUCM01	0.702	0.750	0.729	0.713	0.650
TEMP_M06	0.688	0.740	0.721	0.704	0.639
MDI15O15	0.657	0.715	0.697	0.681	0.614
INCNTM01	0.644	0.699	0.685	0.666	0.600
MADEPM01	0.686	0.743	0.729	0.713	0.639

Covariance Coverage

	WJPCSCG3	WJPCSCG5	WJPCSCX5	RVCSTM36	RELSTM36
WJPCSCG3	0.727				
WJPCSCG5	0.668	0.712			
WJPCSCX5	0.605	0.612	0.650		
RVCSTM36	0.691	0.676	0.617	0.849	
RELSTM36	0.675	0.660	0.601	0.828	0.828
PLSASC54	0.681	0.662	0.609	0.753	0.738
PLSESC54	0.676	0.657	0.606	0.746	0.732
CPINCC54	0.650	0.629	0.582	0.715	0.699
CPOMSC54	0.650	0.629	0.582	0.715	0.699
WJMSSC54	0.680	0.660	0.607	0.747	0.732
CSEX_M01	0.727	0.712	0.650	0.849	0.828
CRACEM01	0.727	0.712	0.650	0.849	0.828
MEDUCM01	0.727	0.712	0.650	0.849	0.828
TEMP_M06	0.719	0.702	0.639	0.836	0.817
MDI15O15	0.696	0.680	0.614	0.810	0.792
INCNTM01	0.683	0.665	0.601	0.792	0.773
MADEPM01	0.727	0.712	0.639	0.840	0.820

Covariance Coverage

	PLSASC54	PLSESC54	CPINCC54	CPOMSC54	WJMSSC54
PLSASC54	0.780				
PLSESC54	0.772	0.773			
CPINCC54	0.727	0.720	0.735		
CPOMSC54	0.727	0.720	0.735	0.735	
WJMSSC54	0.762	0.757	0.730	0.730	0.773
CSEX_M01	0.780	0.773	0.735	0.735	0.773
CRACEM01	0.780	0.773	0.735	0.735	0.773
MEDUCM01	0.780	0.773	0.735	0.735	0.773
TEMP_M06	0.768	0.761	0.721	0.721	0.759
MDI15O15	0.739	0.732	0.699	0.699	0.735
INCNTM01	0.728	0.721	0.687	0.687	0.721
MADEPM01	0.773	0.766	0.728	0.728	0.766

Covariance Coverage

	CSEX_M01	CRACEM01	MEDUCM01	TEMP_M06	MDI15O15
CSEX_M01	1.000				
CRACEM01	1.000	1.000			
MEDUCM01	0.999	0.999	0.999		
TEMP_M06	0.938	0.938	0.938	0.938	
MDI15O15	0.865	0.865	0.865	0.855	0.865
INCNTM01	0.858	0.858	0.858	0.843	0.812
MADEPM01	0.918	0.918	0.918	0.902	0.863

Covariance Coverage

	INCNTM01	MADEPM01
INCNTM01	0.858	
MADEPM01	0.858	0.918

SAMPLE STATISTICS

ESTIMATED SAMPLE STATISTICS

Means

NONDISTR	POSREGAR	INTRUSIV	NONDISTR	POSREGAR
5.150	4.967	5.956	5.282	4.888

Means

INTRUSIV	NONDISTR	POSREGAR	INTRUSIV	SPTSIO36
6.209	5.245	4.921	6.132	5.241

Means

RSCSIO36	HOSTILIT	SPTSIO54	RSCSIO54	HOSTILIT
5.258	6.597	5.118	5.183	6.545

Means

SPTSIO1S	RSCSIO1S	HOSTILIT	SPTSIOG3	RSCSIOG3
5.085	5.189	6.429	4.935	4.845

Means

HOSTILIT	SPTSIOG5	RSCSIOG5	HOSTILIT	MRASIOX5
6.441	5.046	4.916	6.369	4.994

Means

MWRSIOX5	MHISIOX5	SLFR_O1S	SLFR_OG3	SLFR_OG5
13.036	6.156	4.634	4.332	4.674

Means

SLFRLCX5	WJAPSC1S	WJAPSCG3	WJAPSCG5	WJAPSCX5
3.431	109.715	114.328	108.822	102.200

Means

WJPCSCG3	WJPCSCG5	WJPCSCX5	RVCSTM36	RELSTM36
112.006	104.738	106.699	97.184	96.264

Means

PLSASC54	PLSESC54	CPINCC54	CPOMSC54	WJMSSC54
97.287	99.418	15.027	9.452	90.970

Means

CSEX_M01	CRACEM01	MEDUCM01	TEMP_M06	MDI15O15
1.483	3.875	14.234	3.181	108.312

Means

INCNTM01	MADEPM01
2.848	11.381

Covariances

	NONDISTR	POSREGAR	INTRUSIV	NONDISTR	POSREGAR
NONDISTR	1.631				
POSREGAR	0.843	1.390			
INTRUSIV	1.160	0.447	1.859		
NONDISTR	0.522	0.407	0.388	1.448	
POSREGAR	0.387	0.409	0.207	0.680	1.348
INTRUSIV	0.483	0.211	0.577	0.877	0.416
NONDISTR	0.460	0.289	0.347	0.482	0.400
POSREGAR	0.365	0.350	0.224	0.444	0.475
INTRUSIV	0.382	0.195	0.390	0.462	0.260
SPTSIO36	0.635	0.557	0.465	0.606	0.518
RSCSIO36	0.488	0.372	0.415	0.431	0.319
HOSTILIT	0.302	0.256	0.243	0.243	0.218
SPTSIO54	0.547	0.463	0.422	0.527	0.415
RSCSIO54	0.426	0.290	0.360	0.414	0.362

HOSTILIT	0.264	0.166	0.235	0.252	0.196
SPTSIO1S	0.590	0.490	0.429	0.651	0.548
RSCSIO1S	0.472	0.319	0.435	0.499	0.326
HOSTILIT	0.257	0.189	0.218	0.235	0.164
SPTSIOG3	0.451	0.325	0.365	0.461	0.373
RSCSIOG3	0.394	0.259	0.354	0.393	0.267
HOSTILIT	0.137	0.053	0.136	0.135	0.130
SPTSIOG5	0.374	0.275	0.288	0.377	0.287
RSCSIOG5	0.339	0.220	0.284	0.344	0.212
HOSTILIT	0.243	0.160	0.174	0.209	0.151
MRASIOX5	0.302	0.147	0.272	0.234	0.206
MWRSIOX5	0.773	0.641	0.544	0.582	0.560
MHISIOX5	-0.655	-0.290	-0.533	-0.559	-0.483
SLFR_O1S	0.189	0.170	0.176	0.174	0.077
SLFR_OG3	0.151	0.116	0.152	0.177	0.139
SLFR_OG5	0.184	0.171	0.150	0.243	0.187
SLFRLCX5	0.031	0.027	0.030	0.032	0.031
WJAPSC1S	5.629	3.902	4.747	5.409	3.088
WJAPSCG3	3.763	3.139	3.935	3.926	2.371
WJAPSCG5	3.293	2.133	3.756	3.871	2.372
WJAPSCX5	4.548	3.639	4.735	4.160	2.313
WJPCSCG3	4.047	3.265	3.752	4.345	2.762
WJPCSCG5	3.742	2.615	3.657	3.201	2.445
WJPCSCX5	5.484	4.340	5.290	5.182	3.193
RVCSTM36	5.774	4.460	4.997	6.962	4.396
RELSTM36	3.894	2.462	3.446	4.373	2.186
PLSASC54	7.183	5.483	6.511	8.596	5.361
PLSESC54	6.825	5.289	5.779	7.680	4.995
CPINCC54	-3.634	-1.739	-2.210	-3.859	-2.370
CPOMSC54	-1.638	-1.155	-1.199	-1.235	-0.648
WJMSSC54	4.785	3.609	4.047	5.801	3.271
CSEX_M01	0.021	0.003	0.039	0.031	0.018
CRACEM01	0.088	0.052	0.090	0.114	0.066
MEDUCM01	1.219	0.923	1.012	1.048	0.754
TEMP_M06	-0.074	-0.035	-0.063	-0.073	-0.046
MDI15O15	2.641	1.731	1.845	3.968	1.943
INCNTM01	0.899	0.677	0.794	0.806	0.617
MADEPM01	-2.045	-0.962	-1.614	-2.097	-1.375

Covariances

	INTRUSIV	NONDISTR	POSREGAR	INTRUSIV	SPTSIO36
INTRUSIV	1.635				
NONDISTR	0.361	1.708			
POSREGAR	0.225	0.982	1.511		
INTRUSIV	0.554	0.893	0.471	1.610	
SPTSIO36	0.426	0.724	0.614	0.504	1.761
RSCSIO36	0.432	0.553	0.394	0.532	1.009
HOSTILIT	0.232	0.348	0.258	0.331	0.602
SPTSIO54	0.336	0.674	0.560	0.430	0.855
RSCSIO54	0.393	0.530	0.388	0.470	0.622
HOSTILIT	0.240	0.360	0.249	0.370	0.431
SPTSIO1S	0.510	0.718	0.599	0.513	0.893

RSCSIO1S	0.502	0.594	0.386	0.543	0.649
HOSTILIT	0.229	0.334	0.235	0.339	0.425
SPTSIOG3	0.361	0.444	0.319	0.344	0.553
RSCSIOG3	0.362	0.380	0.231	0.323	0.427
HOSTILIT	0.130	0.197	0.106	0.192	0.173
SPTSIOG5	0.324	0.421	0.273	0.356	0.505
RSCSIOG5	0.333	0.326	0.235	0.318	0.447
HOSTILIT	0.225	0.321	0.173	0.307	0.326
MRASIOX5	0.244	0.321	0.271	0.320	0.416
MWRSIOX5	0.356	0.703	0.602	0.537	1.130
MHISIOX5	-0.607	-0.710	-0.580	-0.712	-1.034
SLFR_O1S	0.154	0.186	0.130	0.126	0.167
SLFR_OG3	0.178	0.162	0.152	0.123	0.209
SLFR_OG5	0.199	0.196	0.187	0.128	0.312
SLFRLCX5	0.051	0.041	0.053	0.021	0.043
WJAPSC1S	4.719	6.412	4.323	4.391	7.281
WJAPSCG3	4.391	4.889	4.056	3.702	6.237
WJAPSCG5	4.644	5.054	3.459	3.567	5.080
WJAPSCX5	4.602	4.599	2.965	3.208	5.877
WJPCSCG3	3.783	5.533	3.793	4.219	5.483
WJPCSCG5	2.959	4.492	2.996	3.362	4.630
WJPCSCX5	4.975	5.828	4.125	4.926	7.241
RVCSTM36	5.364	8.176	5.099	6.748	8.801
RELSTM36	2.413	5.387	3.495	4.252	5.493
PLSASC54	6.728	9.105	6.276	7.628	10.782
PLSESC54	6.265	9.303	6.047	7.428	10.592
CPINCC54	-3.508	-5.591	-3.622	-4.764	-5.550
CPOMSC54	-1.155	-1.943	-1.269	-1.420	-1.930
WJMSSC54	3.642	6.033	3.380	3.661	6.076
CSEX_M01	0.034	0.038	0.036	0.088	0.045
CRACEM01	0.147	0.104	0.065	0.084	0.105
MEDUCM01	0.852	1.045	0.866	0.854	1.319
TEMP_M06	-0.048	-0.078	-0.055	-0.068	-0.082
MDI15O15	2.599	4.494	2.992	2.953	3.328
INCNTM01	0.554	0.762	0.648	0.539	1.123
MADEPM01	-1.596	-2.053	-1.572	-1.953	-2.271

Covariances

	RSCSIO36	HOSTILIT	SPTSIO54	RSCSIO54	HOSTILIT
RSCSIO36	1.222				
HOSTILIT	0.497	0.676			
SPTSIO54	0.601	0.378	1.706		
RSCSIO54	0.550	0.283	1.050	1.238	
HOSTILIT	0.366	0.282	0.715	0.638	0.807
SPTSIO1S	0.678	0.446	0.927	0.653	0.486
RSCSIO1S	0.555	0.347	0.633	0.515	0.392
HOSTILIT	0.329	0.317	0.419	0.309	0.356
SPTSIOG3	0.462	0.238	0.546	0.417	0.278
RSCSIOG3	0.389	0.207	0.427	0.347	0.260
HOSTILIT	0.170	0.118	0.229	0.195	0.197
SPTSIOG5	0.410	0.219	0.463	0.391	0.278
RSCSIOG5	0.398	0.184	0.392	0.372	0.242

HOSTILIT	0.281	0.222	0.258	0.230	0.250
MRASIOX5	0.391	0.206	0.359	0.358	0.227
MWRSIOX5	0.927	0.548	1.136	0.952	0.523
MHISIOX5	-0.926	-0.561	-0.916	-0.915	-0.584
SLFR_O1S	0.179	0.158	0.231	0.170	0.122
SLFR_OG3	0.189	0.128	0.252	0.171	0.131
SLFR_OG5	0.188	0.086	0.270	0.165	0.109
SLFRLCX5	0.041	0.013	0.041	0.018	0.016
WJAPSC1S	6.125	3.432	7.154	5.938	3.755
WJAPSCG3	5.396	3.069	5.527	4.475	2.781
WJAPSCG5	4.466	2.873	5.333	4.650	2.823
WJAPSCX5	4.873	2.383	5.658	4.586	2.770
WJPCSCG3	4.618	2.723	5.207	4.494	2.705
WJPCSCG5	3.688	1.986	4.040	3.749	2.267
WJPCSCX5	5.673	3.162	6.277	5.316	3.206
RVCSTM36	7.525	3.534	7.439	6.189	3.395
RELSTM36	4.452	1.974	4.571	3.695	1.824
PLSASC54	8.823	4.418	8.982	7.988	4.559
PLSESC54	8.540	4.029	9.406	7.283	4.477
CPINCC54	-5.397	-3.455	-5.411	-5.851	-4.272
CPOMSC54	-1.694	-0.776	-2.727	-2.187	-1.293
WJMSSC54	6.072	2.396	5.218	4.538	2.387
CSEX_M01	0.053	0.019	-0.011	0.023	0.003
CRACEM01	0.083	0.048	0.088	0.099	0.050
MEDUCM01	1.016	0.577	1.172	0.919	0.565
TEMP_M06	-0.061	-0.035	-0.079	-0.053	-0.019
MDI15O15	2.756	1.125	3.006	2.715	1.259
INCNTM01	0.771	0.431	0.772	0.678	0.300
MADEPM01	-2.237	-1.316	-1.611	-1.289	-1.020

Covariances

	SPTSIO1S	RSCSIO1S	HOSTILIT	SPTSIOG3	RSCSIOG3
SPTSIO1S	1.985				
RSCSIO1S	1.188	1.398			
HOSTILIT	0.776	0.688	0.895		
SPTSIOG3	0.666	0.577	0.332	1.147	
RSCSIOG3	0.506	0.479	0.276	0.846	1.052
HOSTILIT	0.270	0.277	0.206	0.428	0.398
SPTSIOG5	0.579	0.442	0.256	0.439	0.386
RSCSIOG5	0.437	0.400	0.223	0.438	0.388
HOSTILIT	0.386	0.341	0.274	0.314	0.291
MRASIOX5	0.445	0.394	0.248	0.392	0.339
MWRSIOX5	1.243	0.860	0.491	0.958	0.798
MHISIOX5	-1.110	-0.966	-0.605	-0.893	-0.754
SLFR_O1S	0.236	0.208	0.096	0.255	0.240
SLFR_OG3	0.179	0.228	0.098	0.239	0.248
SLFR_OG5	0.251	0.199	0.110	0.191	0.205
SLFRLCX5	0.068	0.048	0.021	0.076	0.065
WJAPSC1S	7.444	6.064	2.804	5.177	5.036
WJAPSCG3	6.254	5.730	2.597	4.750	4.162
WJAPSCG5	5.741	5.018	2.463	4.260	4.007
WJAPSCX5	5.998	4.977	2.306	4.601	4.354

WJPCSCG3	5.694	5.147	2.457	5.060	5.023
WJPCSCG5	4.762	4.432	2.031	3.941	3.774
WJPCSCX5	6.874	6.004	3.273	5.419	5.021
RVCSTM36	8.069	6.457	2.637	5.880	6.006
RELSTM36	4.454	4.192	1.510	4.202	3.657
PLSASC54	11.033	9.294	4.035	7.966	7.355
PLSESC54	10.103	8.452	3.840	7.565	7.075
CPINCC54	-5.747	-4.669	-3.051	-4.456	-4.454
CPOMSC54	-1.858	-1.548	-0.840	-1.670	-1.444
WJMSSC54	6.412	5.184	2.569	4.230	4.378
CSEX_M01	-0.052	0.003	-0.028	0.052	0.065
CRACEM01	0.173	0.146	0.074	0.061	0.051
MEDUCM01	1.414	1.080	0.550	1.046	0.958
TEMP_M06	-0.097	-0.069	-0.020	-0.048	-0.042
MDI15O15	2.884	2.875	0.720	2.654	2.542
INCNTM01	0.951	0.615	0.287	0.794	0.719
MADEPM01	-2.000	-1.661	-1.202	-1.672	-1.379

Covariances

	HOSTILIT	SPTSIOG5	RSCSIOG5	HOSTILIT	MRASIOX5
HOSTILIT	0.695				
SPTSIOG5	0.232	0.916			
RSCSIOG5	0.207	0.706	0.894		
HOSTILIT	0.250	0.561	0.466	0.787	
MRASIOX5	0.198	0.383	0.331	0.283	1.327
MWRSIOX5	0.334	1.085	0.844	0.576	1.992
MHISIOX5	-0.591	-0.840	-0.740	-0.631	-2.704
SLFR_O1S	0.154	0.165	0.147	0.100	0.043
SLFR_OG3	0.171	0.196	0.211	0.099	0.166
SLFR_OG5	0.070	0.172	0.173	0.066	0.077
SLFRLCX5	0.047	0.042	0.063	0.026	0.038
WJAPSC1S	1.272	4.844	4.578	2.379	3.441
WJAPSCG3	1.086	4.038	3.875	2.382	2.830
WJAPSCG5	1.209	4.043	3.749	2.411	2.746
WJAPSCX5	1.191	4.239	4.146	2.694	3.196
WJPCSCG3	1.246	4.474	3.962	2.437	2.857
WJPCSCG5	0.934	3.638	3.469	2.103	2.602
WJPCSCX5	1.071	5.138	4.646	2.974	3.278
RVCSTM36	2.043	5.826	5.500	3.383	3.839
RELSTM36	1.067	3.445	3.116	1.890	2.240
PLSASC54	2.284	7.519	6.729	3.959	4.445
PLSESC54	1.873	7.651	7.167	3.745	3.802
CPINCC54	-3.135	-4.547	-4.008	-3.333	-4.351
CPOMSC54	-0.897	-1.363	-1.328	-0.843	-1.068
WJMSSC54	2.038	4.682	4.561	2.579	2.441
CSEX_M01	0.034	0.049	0.062	0.020	0.028
CRACEM01	0.009	0.099	0.086	0.054	0.050
MEDUCM01	0.369	0.818	0.810	0.456	0.822
TEMP_M06	-0.024	-0.032	-0.015	-0.011	-0.024
MDI15O15	1.196	2.975	2.405	1.488	1.256
INCNTM01	0.216	0.633	0.566	0.323	0.522
MADEPM01	-0.838	-1.769	-1.640	-1.279	-1.270

Covariances

	MWRSIOX5	MHISIOX5	SLFR_O1S	SLFR_OG3	SLFR_OG5
MWRSIOX5	8.618				
MHISIOX5	-4.556	8.071			
SLFR_O1S	0.228	-0.164	1.248		
SLFR_OG3	0.436	-0.275	0.315	0.949	
SLFR_OG5	0.314	-0.092	0.227	0.323	0.850
SLFRLCX5	0.076	-0.099	0.061	0.057	0.037
WJAPSC1S	9.673	-6.129	4.785	5.066	4.460
WJAPSCG3	7.866	-4.517	3.500	4.500	3.510
WJAPSCG5	7.606	-5.598	3.220	3.966	3.967
WJAPSCX5	9.212	-6.062	3.143	4.558	4.247
WJPCSCG3	8.198	-5.058	2.711	4.068	3.644
WJPCSCG5	7.017	-4.606	1.532	3.215	3.445
WJPCSCX5	11.392	-6.887	2.398	4.707	4.587
RVCSTM36	10.117	-7.255	3.996	3.707	3.850
RELSTM36	6.916	-3.789	2.193	3.056	2.801
PLSASC54	12.023	-7.337	4.633	4.600	4.983
PLSESC54	13.276	-6.075	4.521	5.253	5.599
CPINCC54	-8.173	9.161	-3.710	-3.541	-3.539
CPOMSC54	-2.935	2.464	-1.805	-1.072	-1.319
WJMSSC54	8.573	-4.698	2.965	2.989	2.929
CSEX_M01	0.027	-0.069	0.071	0.072	0.058
CRACEM01	0.137	-0.072	-0.006	0.036	0.045
MEDUCM01	1.918	-1.716	0.504	0.565	0.533
TEMP_M06	-0.143	0.089	-0.006	-0.006	-0.035
MDI15O15	5.529	-2.129	2.175	2.028	1.694
INCNTM01	1.454	-1.231	0.362	0.388	0.423
MADEPM01	-2.801	2.727	-0.922	-0.645	-0.782

Covariances

	SLFRLCX5	WJAPSC1S	WJAPSCG3	WJAPSCG5	WJAPSCX5
SLFRLCX5	0.151				
WJAPSC1S	1.344	307.067			
WJAPSCG3	1.226	189.769	227.487		
WJAPSCG5	0.923	170.370	155.063	183.041	
WJAPSCX5	0.877	168.461	146.131	144.465	210.598
WJPCSCG3	0.953	145.447	140.431	119.394	114.926
WJPCSCG5	1.124	121.289	110.022	103.690	101.256
WJPCSCX5	1.259	163.115	141.361	134.719	158.672
RVCSTM36	1.355	161.402	126.656	107.228	105.410
RELSTM36	0.936	97.017	83.584	63.208	67.699
PLSASC54	1.541	207.051	156.533	145.765	145.147
PLSESC54	1.972	195.270	160.894	138.416	125.164
CPINCC54	-0.810	-101.247	-70.622	-59.157	-52.445
CPOMSC54	-0.450	-43.144	-30.485	-25.557	-25.188
WJMSSC54	1.327	150.664	117.198	101.029	97.353
CSEX_M01	0.030	-0.320	-0.110	-0.073	-0.559
CRACEM01	-0.002	1.419	1.251	1.259	0.989

MEDUCM01	0.136	16.105	13.240	12.853	15.026
TEMP_M06	-0.010	-0.837	-0.851	-0.933	-0.843
MDI15O15	0.690	88.003	67.005	52.971	48.664
INCNTM01	0.128	12.020	9.068	10.172	10.082
MADEPM01	-0.264	-27.549	-19.444	-20.233	-16.753

Covariances

	WJPCSCG3	WJPCSCG5	WJPCSCX5	RVCSTM36	RELSTM36
WJPCSCG3	199.944				
WJPCSCG5	132.401	154.853			
WJPCSCX5	153.863	135.311	256.017		
RVCSTM36	118.378	95.220	130.152	256.446	
RELSTM36	82.314	73.287	87.507	136.189	214.833
PLSASC54	153.576	128.253	167.817	237.679	140.576
PLSESC54	150.354	122.357	165.166	225.592	149.258
CPINCC54	-75.084	-48.534	-76.611	-114.777	-50.079
CPOMSC54	-27.535	-21.429	-27.864	-44.847	-24.687
WJMSSC54	123.383	98.437	129.569	167.801	102.896
CSEX_M01	0.738	0.504	0.421	1.528	1.130
CRACEM01	1.100	0.970	1.626	1.751	0.961
MEDUCM01	13.913	11.818	16.745	17.924	11.461
TEMP_M06	-0.611	-0.619	-0.672	-1.008	-0.574
MDI15O15	65.533	43.888	62.545	99.987	61.061
INCNTM01	9.174	7.936	11.120	13.748	6.842
MADEPM01	-16.602	-17.975	-22.108	-28.716	-17.736

Covariances

	PLSASC54	PLSESC54	CPINCC54	CPOMSC54	WJMSSC54
PLSASC54	405.651				
PLSESC54	287.274	406.825			
CPINCC54	-146.479	-144.420	457.806		
CPOMSC54	-48.475	-51.971	40.467	58.460	
WJMSSC54	195.050	204.841	-85.754	-36.259	348.320
CSEX_M01	1.703	1.435	-2.480	-0.268	0.626
CRACEM01	2.107	2.168	-1.487	-0.400	1.118
MEDUCM01	21.719	20.492	-11.097	-3.503	13.631
TEMP_M06	-1.092	-1.271	0.591	0.213	-0.602
MDI15O15	101.662	107.185	-61.293	-22.239	87.898
INCNTM01	17.698	14.888	-7.409	-2.395	10.746
MADEPM01	-33.691	-29.315	18.098	10.137	-28.545

Covariances

	CSEX_M01	CRACEM01	MEDUCM01	TEMP_M06	MDI15O15
CSEX_M01	0.250				
CRACEM01	0.000	0.258			
MEDUCM01	0.046	0.083	6.308		
TEMP_M06	0.009	-0.028	-0.145	0.164	
MDI15O15	0.887	1.133	4.924	-0.398	198.074

INCNTM01	0.017	0.219	2.750	-0.163	4.335
MADEPM01	-0.202	-0.315	-5.276	0.876	-11.289

Covariances

	INCNTM01	MADEPM01
INCNTM01	7.177	
MADEPM01	-4.028	81.877

Correlations

	NONDISTR	POSREGAR	INTRUSIV	NONDISTR	POSREGAR
NONDISTR	1.000				
POSREGAR	0.560	1.000			
INTRUSIV	0.666	0.278	1.000		
NONDISTR	0.339	0.287	0.236	1.000	
POSREGAR	0.261	0.299	0.131	0.487	1.000
INTRUSIV	0.295	0.140	0.331	0.570	0.280
NONDISTR	0.275	0.188	0.194	0.307	0.263
POSREGAR	0.233	0.241	0.134	0.300	0.333
INTRUSIV	0.236	0.130	0.225	0.303	0.176
SPTSIO36	0.375	0.356	0.257	0.380	0.336
RSCSIO36	0.345	0.286	0.275	0.324	0.249
HOSTILIT	0.287	0.264	0.216	0.246	0.229
SPTSIO54	0.328	0.301	0.237	0.335	0.274
RSCSIO54	0.299	0.221	0.237	0.309	0.280
HOSTILIT	0.231	0.157	0.192	0.233	0.188
SPTSIO1S	0.328	0.295	0.223	0.384	0.335
RSCSIO1S	0.313	0.229	0.270	0.350	0.238
HOSTILIT	0.212	0.169	0.169	0.207	0.149
SPTSIOG3	0.330	0.257	0.250	0.358	0.300
RSCSIOG3	0.301	0.214	0.253	0.319	0.225
HOSTILIT	0.129	0.054	0.119	0.134	0.134
SPTSIOG5	0.306	0.244	0.221	0.327	0.258
RSCSIOG5	0.281	0.198	0.220	0.303	0.193
HOSTILIT	0.214	0.153	0.144	0.196	0.146
MRASIOX5	0.205	0.108	0.173	0.169	0.154
MWRSIOX5	0.206	0.185	0.136	0.165	0.164
MHISIOX5	-0.180	-0.087	-0.137	-0.163	-0.146
SLFR_O1S	0.132	0.129	0.116	0.130	0.060
SLFR_OG3	0.121	0.101	0.115	0.151	0.123
SLFR_OG5	0.156	0.157	0.120	0.219	0.174
SLFRLCX5	0.062	0.058	0.056	0.069	0.069
WJAPSC1S	0.252	0.189	0.199	0.256	0.152
WJAPSCG3	0.195	0.176	0.191	0.216	0.135
WJAPSCG5	0.191	0.134	0.204	0.238	0.151
WJAPSCX5	0.245	0.213	0.239	0.238	0.137
WJPCSCG3	0.224	0.196	0.195	0.255	0.168
WJPCSCG5	0.235	0.178	0.216	0.214	0.169
WJPCSCX5	0.268	0.230	0.242	0.269	0.172
RVCSTM36	0.282	0.236	0.229	0.361	0.236
RELSTM36	0.208	0.142	0.172	0.248	0.128

PLSASC54	0.279	0.231	0.237	0.355	0.229
PLSESC54	0.265	0.222	0.210	0.316	0.213
CPINCC54	-0.133	-0.069	-0.076	-0.150	-0.095
CPOMSC54	-0.168	-0.128	-0.115	-0.134	-0.073
WJMSSC54	0.201	0.164	0.159	0.258	0.151
CSEX_M01	0.033	0.006	0.057	0.052	0.032
CRACEM01	0.136	0.087	0.130	0.187	0.112
MEDUCM01	0.380	0.312	0.296	0.347	0.259
TEMP_M06	-0.143	-0.074	-0.114	-0.149	-0.098
MDI15O15	0.147	0.104	0.096	0.234	0.119
INCNTM01	0.263	0.214	0.217	0.250	0.198
MADEPM01	-0.177	-0.090	-0.131	-0.193	-0.131

Correlations

	INTRUSIV	NONDISTR	POSREGAR	INTRUSIV	SPTSIO36
INTRUSIV	1.000				
NONDISTR	0.216	1.000			
POSREGAR	0.143	0.611	1.000		
INTRUSIV	0.341	0.539	0.302	1.000	
SPTSIO36	0.251	0.418	0.376	0.300	1.000
RSCSIO36	0.306	0.383	0.290	0.379	0.688
HOSTILIT	0.221	0.324	0.255	0.318	0.552
SPTSIO54	0.201	0.395	0.349	0.259	0.493
RSCSIO54	0.276	0.365	0.284	0.333	0.421
HOSTILIT	0.209	0.307	0.226	0.325	0.362
SPTSIO1S	0.283	0.390	0.346	0.287	0.478
RSCSIO1S	0.332	0.384	0.266	0.362	0.413
HOSTILIT	0.190	0.270	0.202	0.282	0.338
SPTSIOG3	0.263	0.317	0.242	0.253	0.389
RSCSIOG3	0.276	0.283	0.184	0.249	0.314
HOSTILIT	0.122	0.181	0.104	0.181	0.156
SPTSIOG5	0.265	0.336	0.232	0.293	0.398
RSCSIOG5	0.275	0.263	0.203	0.265	0.357
HOSTILIT	0.198	0.277	0.158	0.273	0.277
MRASIOX5	0.166	0.214	0.191	0.219	0.272
MWRSIOX5	0.095	0.183	0.167	0.144	0.290
MHISIOX5	-0.167	-0.191	-0.166	-0.198	-0.274
SLFR_O1S	0.107	0.127	0.095	0.089	0.113
SLFR_OG3	0.143	0.127	0.127	0.100	0.162
SLFR_OG5	0.169	0.163	0.165	0.110	0.255
SLFRLCX5	0.102	0.082	0.111	0.042	0.083
WJAPSC1S	0.211	0.280	0.201	0.198	0.313
WJAPSCG3	0.228	0.248	0.219	0.193	0.312
WJAPSCG5	0.268	0.286	0.208	0.208	0.283
WJAPSCX5	0.248	0.242	0.166	0.174	0.305
WJPCSCG3	0.209	0.299	0.218	0.235	0.292
WJPCSCG5	0.186	0.276	0.196	0.213	0.280
WJPCSCX5	0.243	0.279	0.210	0.243	0.341
RVCSTM36	0.262	0.391	0.259	0.332	0.414
RELSTM36	0.129	0.281	0.194	0.229	0.282
PLSASC54	0.261	0.346	0.254	0.299	0.403
PLSESC54	0.243	0.353	0.244	0.290	0.396

CPINCC54	-0.128	-0.200	-0.138	-0.175	-0.195
CPOMSC54	-0.118	-0.194	-0.135	-0.146	-0.190
WJMSSC54	0.153	0.247	0.147	0.155	0.245
CSEX_M01	0.053	0.059	0.058	0.139	0.068
CRACEM01	0.227	0.157	0.104	0.130	0.156
MEDUCM01	0.265	0.318	0.281	0.268	0.396
TEMP_M06	-0.092	-0.148	-0.110	-0.133	-0.153
MDI15O15	0.144	0.244	0.173	0.165	0.178
INCNTM01	0.162	0.218	0.197	0.158	0.316
MADEPM01	-0.138	-0.174	-0.141	-0.170	-0.189

Correlations

	RSCSIO36	HOSTILIT	SPTSIO54	RSCSIO54	HOSTILIT
RSCSIO36	1.000				
HOSTILIT	0.547	1.000			
SPTSIO54	0.416	0.352	1.000		
RSCSIO54	0.447	0.309	0.723	1.000	
HOSTILIT	0.368	0.381	0.610	0.638	1.000
SPTSIO1S	0.436	0.385	0.504	0.416	0.384
RSCSIO1S	0.425	0.357	0.410	0.391	0.369
HOSTILIT	0.314	0.408	0.339	0.293	0.419
SPTSIOG3	0.391	0.270	0.390	0.350	0.289
RSCSIOG3	0.343	0.245	0.319	0.304	0.283
HOSTILIT	0.185	0.173	0.210	0.210	0.263
SPTSIOG5	0.388	0.279	0.370	0.367	0.323
RSCSIOG5	0.381	0.236	0.318	0.353	0.286
HOSTILIT	0.286	0.304	0.223	0.233	0.314
MRASIOX5	0.307	0.217	0.239	0.279	0.220
MWRSIOX5	0.286	0.227	0.296	0.291	0.198
MHISIOX5	-0.295	-0.240	-0.247	-0.289	-0.229
SLFR_O1S	0.145	0.172	0.159	0.137	0.121
SLFR_OG3	0.176	0.160	0.198	0.157	0.150
SLFR_OG5	0.185	0.113	0.224	0.161	0.132
SLFRLCX5	0.096	0.042	0.081	0.042	0.045
WJAPSC1S	0.316	0.238	0.313	0.305	0.239
WJAPSCG3	0.324	0.248	0.281	0.267	0.205
WJAPSCG5	0.299	0.258	0.302	0.309	0.232
WJAPSCX5	0.304	0.200	0.299	0.284	0.212
WJPCSCG3	0.296	0.234	0.282	0.286	0.213
WJPCSCG5	0.268	0.194	0.249	0.271	0.203
WJPCSCX5	0.321	0.240	0.300	0.299	0.223
RVCSTM36	0.425	0.268	0.356	0.347	0.236
RELSTM36	0.275	0.164	0.239	0.227	0.139
PLSASC54	0.396	0.267	0.341	0.356	0.252
PLSESC54	0.383	0.243	0.357	0.324	0.247
CPINCC54	-0.228	-0.196	-0.194	-0.246	-0.222
CPOMSC54	-0.200	-0.124	-0.273	-0.257	-0.188
WJMSSC54	0.294	0.156	0.214	0.218	0.142
CSEX_M01	0.097	0.047	-0.016	0.041	0.006
CRACEM01	0.147	0.114	0.132	0.176	0.111
MEDUCM01	0.366	0.280	0.357	0.329	0.251
TEMP_M06	-0.137	-0.105	-0.149	-0.117	-0.053

MDI15O15	0.177	0.097	0.164	0.173	0.100
INCNTM01	0.260	0.196	0.221	0.227	0.125
MADEPM01	-0.224	-0.177	-0.136	-0.128	-0.125

Correlations

	SPTSIO1S	RSCSIO1S	HOSTILIT	SPTSIOG3	RSCSIOG3
SPTSIO1S	1.000				
RSCSIO1S	0.713	1.000			
HOSTILIT	0.582	0.615	1.000		
SPTSIOG3	0.441	0.456	0.327	1.000	
RSCSIOG3	0.350	0.395	0.284	0.770	1.000
HOSTILIT	0.230	0.281	0.261	0.480	0.465
SPTSIOG5	0.429	0.391	0.283	0.429	0.393
RSCSIOG5	0.328	0.358	0.249	0.433	0.400
HOSTILIT	0.309	0.325	0.326	0.330	0.319
MRASIOX5	0.274	0.289	0.228	0.318	0.287
MWRSIOX5	0.301	0.248	0.177	0.305	0.265
MHISIOX5	-0.277	-0.288	-0.225	-0.294	-0.259
SLFR_O1S	0.150	0.157	0.091	0.213	0.210
SLFR_OG3	0.130	0.198	0.107	0.229	0.249
SLFR_OG5	0.193	0.183	0.126	0.193	0.217
SLFRLCX5	0.123	0.103	0.057	0.183	0.164
WJAPSC1S	0.302	0.293	0.169	0.276	0.280
WJAPSCG3	0.294	0.321	0.182	0.294	0.269
WJAPSCG5	0.301	0.314	0.192	0.294	0.289
WJAPSCX5	0.293	0.290	0.168	0.296	0.293
WJPCSCG3	0.286	0.308	0.184	0.334	0.346
WJPCSCG5	0.272	0.301	0.172	0.296	0.296
WJPCSCX5	0.305	0.317	0.216	0.316	0.306
RVCSTM36	0.358	0.341	0.174	0.343	0.366
RELSTM36	0.216	0.242	0.109	0.268	0.243
PLSASC54	0.389	0.390	0.212	0.369	0.356
PLSESC54	0.356	0.354	0.201	0.350	0.342
CPINCC54	-0.191	-0.185	-0.151	-0.194	-0.203
CPOMSC54	-0.173	-0.171	-0.116	-0.204	-0.184
WJMSSC54	0.244	0.235	0.146	0.212	0.229
CSEX_M01	-0.074	0.006	-0.060	0.098	0.127
CRACEM01	0.242	0.243	0.154	0.111	0.097
MEDUCM01	0.400	0.364	0.231	0.389	0.372
TEMP_M06	-0.171	-0.144	-0.051	-0.110	-0.101
MDI15O15	0.145	0.173	0.054	0.176	0.176
INCNTM01	0.252	0.194	0.113	0.277	0.262
MADEPM01	-0.157	-0.155	-0.140	-0.173	-0.149

Correlations

	HOSTILIT	SPTSIOG5	RSCSIOG5	HOSTILIT	MRASIOX5
HOSTILIT	1.000				
SPTSIOG5	0.291	1.000			
RSCSIOG5	0.262	0.780	1.000		
HOSTILIT	0.338	0.661	0.555	1.000	

MRASIOX5	0.206	0.347	0.304	0.277	1.000
MWRSIOX5	0.137	0.386	0.304	0.221	0.589
MHISIOX5	-0.250	-0.309	-0.276	-0.250	-0.826
SLFR_O1S	0.165	0.155	0.139	0.101	0.033
SLFR_OG3	0.210	0.211	0.229	0.114	0.148
SLFR_OG5	0.091	0.195	0.198	0.081	0.072
SLFRLCX5	0.145	0.114	0.170	0.074	0.084
WJAPSC1S	0.087	0.289	0.276	0.153	0.171
WJAPSCG3	0.086	0.280	0.272	0.178	0.163
WJAPSCG5	0.107	0.312	0.293	0.201	0.176
WJAPSCX5	0.098	0.305	0.302	0.209	0.191
WJPCSCG3	0.106	0.331	0.296	0.194	0.175
WJPCSCG5	0.090	0.305	0.295	0.190	0.182
WJPCSCX5	0.080	0.335	0.307	0.209	0.178
RVCSTM36	0.153	0.380	0.363	0.238	0.208
RELSTM36	0.087	0.246	0.225	0.145	0.133
PLSASC54	0.136	0.390	0.353	0.222	0.192
PLSESC54	0.111	0.396	0.376	0.209	0.164
CPINCC54	-0.176	-0.222	-0.198	-0.176	-0.177
CPOMSC54	-0.141	-0.186	-0.184	-0.124	-0.121
WJMSSC54	0.131	0.262	0.259	0.156	0.114
CSEX_M01	0.083	0.102	0.132	0.045	0.048
CRACEM01	0.020	0.204	0.179	0.121	0.085
MEDUCM01	0.176	0.340	0.341	0.205	0.284
TEMP_M06	-0.070	-0.083	-0.038	-0.031	-0.051
MDI15O15	0.102	0.221	0.181	0.119	0.077
INCNTM01	0.097	0.247	0.224	0.136	0.169
MADEPM01	-0.111	-0.204	-0.192	-0.159	-0.122

Correlations

	MWRSIOX5	MHISIOX5	SLFR_O1S	SLFR_OG3	SLFR_OG5
MWRSIOX5	1.000				
MHISIOX5	-0.546	1.000			
SLFR_O1S	0.070	-0.052	1.000		
SLFR_OG3	0.152	-0.099	0.289	1.000	
SLFR_OG5	0.116	-0.035	0.221	0.359	1.000
SLFRLCX5	0.067	-0.090	0.139	0.151	0.102
WJAPSC1S	0.188	-0.123	0.244	0.297	0.276
WJAPSCG3	0.178	-0.105	0.208	0.306	0.252
WJAPSCG5	0.192	-0.146	0.213	0.301	0.318
WJAPSCX5	0.216	-0.147	0.194	0.322	0.317
WJPCSCG3	0.197	-0.126	0.172	0.295	0.280
WJPCSCG5	0.192	-0.130	0.110	0.265	0.300
WJPCSCX5	0.243	-0.152	0.134	0.302	0.311
RVCSTM36	0.215	-0.159	0.223	0.238	0.261
RELSTM36	0.161	-0.091	0.134	0.214	0.207
PLSASC54	0.203	-0.128	0.206	0.235	0.268
PLSESC54	0.224	-0.106	0.201	0.267	0.301
CPINCC54	-0.130	0.151	-0.155	-0.170	-0.179
CPOMSC54	-0.131	0.113	-0.211	-0.144	-0.187
WJMSSC54	0.156	-0.089	0.142	0.164	0.170
CSEX_M01	0.018	-0.049	0.126	0.148	0.126

CRACEM01	0.092	-0.050	-0.010	0.073	0.097
MEDUCM01	0.260	-0.240	0.179	0.231	0.230
TEMP_M06	-0.121	0.078	-0.013	-0.014	-0.095
MDI15O15	0.134	-0.053	0.138	0.148	0.131
INCNTM01	0.185	-0.162	0.121	0.149	0.171
MADEPM01	-0.105	0.106	-0.091	-0.073	-0.094

Correlations

	SLFRLCX5	WJAPSC1S	WJAPSCG3	WJAPSCG5	WJAPSCX5
SLFRLCX5	1.000				
WJAPSC1S	0.197	1.000			
WJAPSCG3	0.209	0.718	1.000		
WJAPSCG5	0.175	0.719	0.760	1.000	
WJAPSCX5	0.155	0.662	0.668	0.736	1.000
WJPCSCG3	0.173	0.587	0.658	0.624	0.560
WJPCSCG5	0.232	0.556	0.586	0.616	0.561
WJPCSCX5	0.202	0.582	0.586	0.622	0.683
RVCSTM36	0.217	0.575	0.524	0.495	0.454
RELSTM36	0.164	0.378	0.378	0.319	0.318
PLSASC54	0.197	0.587	0.515	0.535	0.497
PLSESC54	0.251	0.552	0.529	0.507	0.428
CPINCC54	-0.097	-0.270	-0.219	-0.204	-0.169
CPOMSC54	-0.151	-0.322	-0.264	-0.247	-0.227
WJMSSC54	0.183	0.461	0.416	0.400	0.359
CSEX_M01	0.157	-0.037	-0.015	-0.011	-0.077
CRACEM01	-0.011	0.159	0.163	0.183	0.134
MEDUCM01	0.139	0.366	0.350	0.378	0.412
TEMP_M06	-0.062	-0.118	-0.139	-0.171	-0.144
MDI15O15	0.126	0.357	0.316	0.278	0.238
INCNTM01	0.122	0.256	0.224	0.281	0.259
MADEPM01	-0.075	-0.174	-0.142	-0.165	-0.128

Correlations

	WJPCSCG3	WJPCSCG5	WJPCSCX5	RVCSTM36	RELSTM36
WJPCSCG3	1.000				
WJPCSCG5	0.752	1.000			
WJPCSCX5	0.680	0.680	1.000		
RVCSTM36	0.523	0.478	0.508	1.000	
RELSTM36	0.397	0.402	0.373	0.580	1.000
PLSASC54	0.539	0.512	0.521	0.737	0.476
PLSESC54	0.527	0.487	0.512	0.698	0.505
CPINCC54	-0.248	-0.182	-0.224	-0.335	-0.160
CPOMSC54	-0.255	-0.225	-0.228	-0.366	-0.220
WJMSSC54	0.468	0.424	0.434	0.561	0.376
CSEX_M01	0.104	0.081	0.053	0.191	0.154
CRACEM01	0.153	0.153	0.200	0.215	0.129
MEDUCM01	0.392	0.378	0.417	0.446	0.311
TEMP_M06	-0.107	-0.123	-0.104	-0.156	-0.097
MDI15O15	0.329	0.251	0.278	0.444	0.296
INCNTM01	0.242	0.238	0.259	0.320	0.174

MADEPM01	-0.130	-0.160	-0.153	-0.198	-0.134

Correlations

	PLSASC54	PLSESC54	CPINCC54	CPOMSC54	WJMSSC54
PLSASC54	1.000				
PLSESC54	0.707	1.000			
CPINCC54	-0.340	-0.335	1.000		
CPOMSC54	-0.315	-0.337	0.247	1.000	
WJMSSC54	0.519	0.544	-0.215	-0.254	1.000
CSEX_M01	0.169	0.142	-0.232	-0.070	0.067
CRACEM01	0.206	0.212	-0.137	-0.103	0.118
MEDUCM01	0.429	0.404	-0.206	-0.182	0.291
TEMP_M06	-0.134	-0.156	0.068	0.069	-0.080
MDI15O15	0.359	0.378	-0.204	-0.207	0.335
INCNTM01	0.328	0.276	-0.129	-0.117	0.215
MADEPM01	-0.185	-0.161	0.093	0.147	-0.169

Correlations

	CSEX_M01	CRACEM01	MEDUCM01	TEMP_M06	MDI15O15
CSEX_M01	1.000				
CRACEM01	0.002	1.000			
MEDUCM01	0.036	0.065	1.000		
TEMP_M06	0.044	-0.137	-0.142	1.000	
MDI15O15	0.126	0.159	0.139	-0.070	1.000
INCNTM01	0.013	0.161	0.409	-0.151	0.115
MADEPM01	-0.045	-0.069	-0.232	0.239	-0.089

Correlations

	INCNTM01	MADEPM01
INCNTM01	1.000	
MADEPM01	-0.166	1.000

MAXIMUM LOG-LIKELIHOOD VALUE FOR THE UNRESTRICTED (H1) MODEL IS -114269.818

UNIVARIATE SAMPLE STATISTICS

UNIVARIATE HIGHER-ORDER MOMENT DESCRIPTIVE STATISTICS

Variable/ Sample Size	Mean/ Variance	Skewness/ Kurtosis	Minimum/ Maximum	% with Min/Max	Percentiles 20%/60%	40%/80%	Median
NONDISTRESS6M_7	5.170	-0.187	1.750	1.65%	3.500	5.250	5.250
1272.000	1.621	-0.467	7.000	22.64%	5.250	7.000	
POSREGARD6M_7TR	4.984	0.033	1.750	0.79%	3.500	5.250	5.250
1272.000	1.381	-0.485	7.000	15.33%	5.250	5.250	

INTRUSIVENESS6M 5.970 -1.080 1.750 1.73% 5.250 5.250 7.000
1272.000 1.854 0.210 7.000 57.55% 7.000 7.000
NONDISTRESS15M_ 5.306 -0.282 1.750 1.29% 5.250 5.250 5.250
1240.000 1.434 -0.114 7.000 23.79% 5.250 7.000
POSREGARD15M_7T 4.906 -0.080 1.750 1.53% 3.500 5.250 5.250
1240.000 1.339 -0.181 7.000 12.42% 5.250 5.250
INTRUSIVENESS15 6.229 -1.586 1.750 1.77% 5.250 7.000 7.000
1240.000 1.629 1.727 7.000 68.47% 7.000 7.000
NONDISTRESS24M_ 5.272 -0.406 1.750 2.65% 3.500 5.250 5.250
1172.000 1.695 -0.131 7.000 25.68% 5.250 7.000
POSREGARD24M_7T 4.942 -0.105 1.750 2.05% 3.500 5.250 5.250
1172.000 1.494 -0.278 7.000 15.10% 5.250 5.250
INTRUSIVENESS_R 6.156 -1.471 1.750 2.05% 5.250 7.000 7.000
1172.000 1.606 1.654 7.000 63.48% 7.000 7.000
SPTSIO36 5.277 -0.905 1.000 1.38% 4.000 5.000 6.000
1161.000 1.732 0.717 7.000 15.85% 6.000 6.000
RSCSIO36 5.291 -0.910 1.000 0.52% 5.000 5.000 5.000
1161.000 1.206 1.208 7.000 9.13% 6.000 6.000
HOSTILITY36M_RE 6.618 -2.983 1.000 0.17% 6.000 7.000 7.000
1161.000 0.663 11.265 7.000 74.76% 7.000 7.000
SPTSIO54 5.160 -0.753 1.000 0.96% 4.000 5.000 5.000
1040.000 1.682 0.354 7.000 12.88% 6.000 6.000
RSCSIO54 5.221 -0.890 1.000 0.38% 4.000 5.000 5.000
1040.000 1.226 0.982 7.000 7.40% 6.000 6.000
HOSTILITY54M_RE 6.568 -2.749 2.000 0.96% 6.000 7.000 7.000
1040.000 0.799 8.453 7.000 72.98% 7.000 7.000
SPTSIO1S 5.157 -0.754 1.000 1.20% 4.000 5.000 5.000
1004.000 1.917 0.143 7.000 15.34% 6.000 6.000
RSCSIO1S 5.256 -0.695 1.000 0.20% 4.000 5.000 5.000
1004.000 1.350 0.346 7.000 11.35% 6.000 6.000
HOSTILITYG1_REV 6.466 -2.133 2.000 0.50% 6.000 7.000 7.000
1004.000 0.858 4.802 7.000 66.24% 7.000 7.000
SPTSIOG3 4.990 -0.473 1.000 0.10% 4.000 5.000 5.000
982.000 1.138 0.048 7.000 4.79% 5.000 6.000
RSCSIOG3 4.894 -0.205 1.000 0.10% 4.000 5.000 5.000
982.000 1.044 0.028 7.000 4.89% 5.000 6.000
HOSTILITYG3_REV 6.460 -2.350 1.000 0.10% 6.000 6.000 7.000
982.000 0.694 7.899 7.000 59.67% 7.000 7.000
SPTSIOG5 5.112 -0.611 2.000 0.54% 4.000 5.000 5.000
929.000 0.887 0.087 7.000 2.48% 5.000 6.000
RSCSIOG5 4.977 -0.144 2.000 0.11% 4.000 5.000 5.000
929.000 0.877 -0.162 7.000 4.09% 5.000 6.000
HOSTILITYG5_REV 6.414 -1.699 2.000 0.22% 6.000 7.000 7.000
929.000 0.759 3.104 7.000 60.39% 7.000 7.000
MRASIOX5 5.048 -0.578 1.000 0.78% 4.000 5.000 5.000
898.000 1.304 0.668 7.000 8.57% 5.000 6.000
MWRSIOX5 13.164 0.011 4.000 0.11% 11.000 12.000 13.000
897.000 8.567 -0.045 21.000 0.78% 14.000 16.000
MHISIOX5 6.033 1.869 3.000 7.80% 4.000 5.000 5.000
898.000 7.912 3.957 21.000 0.11% 5.000 8.000
SLFR_O1S 4.660 -0.329 1.000 0.10% 3.667 4.333 4.667
966.000 1.241 -0.207 7.000 1.45% 5.000 5.667
SLFR_OG3 4.363 -0.275 1.125 0.10% 3.625 4.125 4.375
971.000 0.940 0.016 7.000 0.10% 4.625 5.125

SLFR_OG5	4.708	-0.464	1.000	0.10%	4.000	4.500	4.750
	955.000	0.845	0.506	7.000	0.10%	5.000	5.500
SLFRLCX5	3.440	-0.885	1.300	0.10%	3.100	3.400	3.500
	957.000	0.150	1.278	4.000	6.69%	3.600	3.800
WJAPSC1S	110.804	-0.022	46.000	0.10%	96.000	106.000	111.000
	1023.000	293.407	-0.049	163.000	0.10%	117.000	125.000
WJAPSCG3	115.162	-0.806	30.000	0.10%	103.000	114.000	117.000
	994.000	222.798	2.041	153.000	0.10%	120.000	127.000
WJAPSCG5	109.324	-0.400	37.000	0.10%	98.000	106.000	110.000
	973.000	180.899	1.741	156.000	0.10%	113.000	120.000
WJAPSCX5	102.921	0.731	48.000	0.11%	92.000	97.000	99.000
	887.000	202.100	1.580	168.000	0.11%	104.000	115.000
WJPCSCG3	112.827	-0.548	47.000	0.10%	101.000	111.000	114.000
	992.000	195.117	0.887	147.000	0.10%	118.000	124.000
WJPCSCG5	105.365	-0.356	29.000	0.10%	96.000	102.000	105.000
	971.000	150.681	2.486	151.000	0.10%	108.000	115.000
WJPCSCX5	107.710	0.402	44.000	0.11%	95.000	100.000	105.000
	887.000	246.894	0.529	160.000	0.11%	110.000	120.000
RVCSTM36	97.845	-0.172	62.000	1.99%	83.000	94.000	98.000
	1158.000	251.096	-0.593	136.000	0.09%	104.000	112.000
RELSTM36	96.876	-0.095	62.000	1.06%	84.000	94.000	99.000
	1130.000	210.855	-0.164	138.000	0.27%	101.000	108.000
PLSASC54	98.345	-0.027	50.000	0.38%	79.000	88.000	98.000
	1064.000	396.450	-1.095	139.000	0.19%	109.000	120.000
PLSESC54	100.616	-0.545	50.000	0.38%	81.000	97.000	104.000
	1055.000	397.668	-0.836	128.000	0.09%	111.000	119.000
CPINCC54	14.188	2.838	0.000	8.18%	1.000	4.000	6.000
	1002.000	453.297	9.632	154.000	0.10%	8.000	21.000
CPOMSC54	9.134	1.156	0.000	4.29%	3.000	5.000	7.000
	1002.000	57.607	1.000	41.067	0.10%	9.000	15.000
WJMSSC54	91.745	-0.150	17.000	0.09%	72.000	88.000	93.000
	1054.000	341.380	-0.442	142.000	0.09%	98.000	109.000
CSEX_M01	1.483	0.067	1.000	51.69%	1.000	1.000	1.000
	1364.000	0.250	-1.995	2.000	48.31%	2.000	2.000
CRACEM01	3.875	-1.614	1.000	0.37%	4.000	4.000	4.000
	1364.000	0.258	6.039	5.000	4.69%	4.000	4.000
MEDUCM01	14.234	0.148	7.000	0.22%	12.000	14.000	14.000
	1363.000	6.308	-0.036	21.000	1.69%	14.000	16.000
TEMP_M06	3.178	-0.131	1.540	0.08%	2.852	3.094	3.200
	1279.000	0.163	0.473	4.722	0.08%	3.292	3.491
MDI15O15	108.580	-0.074	63.000	0.08%	97.000	106.000	109.000
	1180.000	197.684	0.074	150.000	0.59%	112.000	121.000
INCNTM01	2.960	2.529	0.082	0.09%	1.086	1.991	2.307
	1170.000	7.055	10.017	25.084	0.09%	2.736	4.153
MADEPM01	11.272	1.289	0.000	3.83%	4.000	7.000	9.000
	1252.000	81.449	1.730	53.000	0.08%	11.000	18.000

THIS ANALYSIS MAY HAVE MULTIPLE SOLUTIONS. EXPLORE THIS USING RANDOM STARTS, FOR EXAMPLE, STARTS = 20. USE A LARGE ENOUGH NUMBER OF STARTS SO THAT THE BEST FIT FUNCTION VALUE IS REPLICATED SEVERAL TIMES.

THE MODEL ESTIMATION TERMINATED NORMALLY

MODEL FIT INFORMATION

Number of Free Parameters 300

Loglikelihood

```
     H0 Value                  -115804.174
     H0 Scaling Correction Factor      1.1801
       for MLR
     H1 Value                  -114269.818
     H1 Scaling Correction Factor      1.0606
       for MLR
```

Information Criteria

```
     Akaike (AIC)               232208.347
     Bayesian (BIC)             233773.800
     Sample-Size Adjusted BIC      232820.824
       (n* = (n + 2) / 24)
```

Chi-Square Test of Model Fit

```
     Value                     2982.771*
     Degrees of Freedom             1130
     P-Value                     0.0000
     Scaling Correction Factor        1.0288
       for MLR
```

* The chi-square value for MLM, MLMV, MLR, ULSMV, WLSM and WLSMV cannot be used
for chi-square difference testing in the regular way. MLM, MLR and WLSM
chi-square difference testing is described on the Mplus website. MLMV, WLSMV,
and ULSMV difference testing is done using the DIFFTEST option.

RMSEA (Root Mean Square Error Of Approximation)

```
     Estimate                   0.035
     90 Percent C.I.               0.033  0.036
     Probability RMSEA <= .05         1.000
```

CFI/TLI

```
     CFI                        0.923
     TLI                        0.911
```

Chi-Square Test of Model Fit for the Baseline Model

```
     Value                    25290.694
     Degrees of Freedom             1305
     P-Value                     0.0000
```

SRMR (Standardized Root Mean Square Residual)

Value 0.075

MODEL RESULTS

	Estimate	S.E.	Est./S.E.	Two-Tailed P-Value
MS_6M BY				
NONDISTRES	1.000	0.000	999.000	999.000
POSREGARD6	0.518	0.028	18.749	0.000
INTRUSIVEN	0.716	0.033	21.668	0.000
MS_15M BY				
NONDISTRES	1.000	0.000	999.000	999.000
POSREGARD1	0.617	0.036	17.037	0.000
INTRUSIVEN	0.802	0.046	17.552	0.000
MS_24M BY				
NONDISTRES	1.000	0.000	999.000	999.000
POSREGARD2	0.693	0.032	21.704	0.000
INTRUSIVEN	0.647	0.043	14.991	0.000
MS_36M BY				
SPTSIO36	1.000	0.000	999.000	999.000
RSCSIO36	0.858	0.033	26.231	0.000
HOSTILITY3	0.502	0.036	13.823	0.000
MS_54M BY				
SPTSIO54	1.000	0.000	999.000	999.000
RSCSIO54	0.853	0.031	27.590	0.000
HOSTILITY5	0.594	0.036	16.474	0.000
MS_G1 BY				
SPTSIO1S	1.000	0.000	999.000	999.000
RSCSIO1S	0.879	0.035	24.906	0.000
HOSTILITYG	0.557	0.034	16.264	0.000
MS_G3 BY				
SPTSIOG3	1.000	0.000	999.000	999.000
RSCSIOG3	0.873	0.032	27.053	0.000
HOSTILITYG	0.451	0.036	12.402	0.000
MS_G5 BY				
SPTSIOG5	1.000	0.000	999.000	999.000
RSCSIOG5	0.857	0.028	30.646	0.000
HOSTILITYG	0.663	0.034	19.330	0.000
MS_15 BY				
MRASIOX5	1.000	0.000	999.000	999.000
MWRSIOX5	1.700	0.080	21.363	0.000
MHISIOX5	-2.260	0.089	-25.369	0.000

```
SR       BY
   SLFR_O1S           1.000      0.000    999.000    999.000
   SLFR_OG3           1.000      0.000    999.000    999.000
   SLFR_OG5           1.000      0.000    999.000    999.000
   SLFRLCX5           1.000      0.000    999.000    999.000

SLFR_G1R BY
   SLFR_O1S           1.000      0.000    999.000    999.000

SLFR_G3R BY
   SLFR_OG3           1.000      0.000    999.000    999.000

SLFR_G5R BY
   SLFR_OG5           1.000      0.000    999.000    999.000

SLFR_15R BY
   SLFRLCX5           1.000      0.000    999.000    999.000

READ     BY
   WJPCSCG3           1.000      0.000    999.000    999.000
   WJPCSCG5           1.000      0.000    999.000    999.000
   WJPCSCX5           1.000      0.000    999.000    999.000

WJPCSCG3 BY
   WJPCSCG3           1.000      0.000    999.000    999.000

WJPCSCG5 BY
   WJPCSCG5           1.000      0.000    999.000    999.000

WJPCSCX5 BY
   WJPCSCX5           1.000      0.000    999.000    999.000

MATH     BY
   WJAPSC1S           1.000      0.000    999.000    999.000
   WJAPSCG3           1.000      0.000    999.000    999.000
   WJAPSCG5           1.000      0.000    999.000    999.000
   WJAPSCX5           1.000      0.000    999.000    999.000

WJAPSC1S BY
   WJAPSC1S           1.000      0.000    999.000    999.000

WJAPSCG3 BY
   WJAPSCG3           1.000      0.000    999.000    999.000

WJAPSCG5 BY
   WJAPSCG5           1.000      0.000    999.000    999.000

WJAPSCX5 BY
   WJAPSCX5           1.000      0.000    999.000    999.000

MS_RI    BY
   MS_6M              1.000      0.000    999.000    999.000
   MS_15M             1.000      0.000    999.000    999.000
```

```
    MS_24M           1.000      0.000    999.000    999.000
    MS_36M           1.000      0.000    999.000    999.000
    MS_54M           1.000      0.000    999.000    999.000
    MS_G1            1.000      0.000    999.000    999.000
    MS_G3            1.000      0.000    999.000    999.000
    MS_G5            1.000      0.000    999.000    999.000
    MS_15            1.000      0.000    999.000    999.000

 MS_6MR   BY
    MS_6M            1.000      0.000    999.000    999.000

 MS_15MR  BY
    MS_15M           1.000      0.000    999.000    999.000

 MS_24MR  BY
    MS_24M           1.000      0.000    999.000    999.000

 MS_36MR  BY
    MS_36M           1.000      0.000    999.000    999.000

 MS_54MR  BY
    MS_54M           1.000      0.000    999.000    999.000

 MS_G1R   BY
    MS_G1            1.000      0.000    999.000    999.000

 MS_G3R   BY
    MS_G3            1.000      0.000    999.000    999.000

 MS_G5R   BY
    MS_G5            1.000      0.000    999.000    999.000

 MS_15R   BY
    MS_15            1.000      0.000    999.000    999.000

 MS_15MR  ON
    MS_6MR           0.064      0.029      2.205      0.027

 MS_36MR  ON
    MS_24MR          0.186      0.044      4.285      0.000

 MS_54MR  ON
    MS_36MR          0.342      0.060      5.698      0.000

 MS_G1R   ON
    MS_54MR          0.236      0.062      3.780      0.000

 MS_G3R   ON
    MS_G1R           0.150      0.046      3.277      0.001

 SLFR_G3R ON
    SLFR_G1R         0.167      0.031      5.377      0.000
    WJAPSC1S_R       0.007      0.003      2.261      0.024
```

```
 WJPCSCG3 ON
    WJAPSC1S_R         0.085      0.037      2.296      0.022

 WJAPSCG3 ON
    WJAPSC1S_R         0.241      0.043      5.611      0.000

 MS_G5R   ON
    MS_G3R             0.111      0.049      2.264      0.024

 SLFR_G5R ON
    SLFR_G3R           0.250      0.035      7.215      0.000
    WJPCSCG3_R         0.002      0.004      0.375      0.708

 WJPCSCG5 ON
    WJAPSCG3_R        -0.088      0.034     -2.622      0.009
    WJPCSCG3_R         0.085      0.066      1.294      0.196

 WJAPSCG5 ON
    WJAPSCG3_R         0.217      0.057      3.824      0.000
    WJPCSCG3_R         0.028      0.057      0.492      0.623

 MS_15R   ON
    MS_G5R             0.179      0.066      2.716      0.007

 SLFR_15R ON
    SLFR_G5R          -0.048      0.022     -2.226      0.026
    WJAPSCG5_R        -0.006      0.002     -2.381      0.017

 WJPCSCX5 ON
    SLFR_G5R           1.557      0.492      3.165      0.002
    WJPCSCG5_R        -0.483      0.285     -1.695      0.090
    WJAPSCG5_R        -0.076      0.108     -0.702      0.482

 WJAPSCX5 ON
    SLFR_G5R           1.291      0.406      3.180      0.001
    WJPCSCG5_R        -0.450      0.178     -2.530      0.011
    WJAPSCG5_R         0.067      0.117      0.569      0.569

 MS_RI    ON
    CSEX_M01           0.070      0.034      2.030      0.042
    CRACEM01           0.246      0.042      5.926      0.000
    MEDUCM01           0.130      0.008     16.559      0.000
    TEMP_M06          -0.100      0.043     -2.318      0.020
    MDI15O15           0.009      0.001      7.083      0.000
    INCNTM01           0.036      0.009      4.167      0.000
    MADEPM01          -0.009      0.002     -4.413      0.000

 SR       ON
    CSEX_M01           0.137      0.022      6.258      0.000
    CRACEM01          -0.019      0.023     -0.855      0.392
    MEDUCM01           0.025      0.005      5.027      0.000
    TEMP_M06          -0.040      0.031     -1.288      0.198
    MDI15O15           0.004      0.001      3.959      0.000
    INCNTM01           0.013      0.005      2.643      0.008
```

```
   MADEPM01          -0.001      0.001     -0.826      0.409

 MATH     ON
   CSEX_M01         -2.685      0.653     -4.114      0.000
   CRACEM01          1.898      0.801      2.371      0.018
   MEDUCM01          1.515      0.154      9.853      0.000
   TEMP_M06         -2.046      0.854     -2.397      0.017
   MDI15O15          0.214      0.027      8.057      0.000
   INCNTM01          0.393      0.148      2.645      0.008
   MADEPM01         -0.058      0.039     -1.467      0.142

 READ     ON
   CSEX_M01          0.841      0.662      1.271      0.204
   CRACEM01          2.384      0.710      3.360      0.001
   MEDUCM01          1.725      0.158     10.900      0.000
   TEMP_M06         -0.945      0.833     -1.135      0.256
   MDI15O15          0.203      0.025      8.169      0.000
   INCNTM01          0.330      0.140      2.362      0.018
   MADEPM01         -0.052      0.039     -1.357      0.175

 MS_G1R   ON
   PLSASC54          0.007      0.002      3.882      0.000

 SLFR_G1R ON
   CPOMSC54         -0.024      0.005     -4.888      0.000

 WJAPSC1S ON
   PLSASC54          0.117      0.028      4.197      0.000
   PLSESC54          0.058      0.028      2.076      0.038
   CPOMSC54         -0.182      0.051     -3.599      0.000
   WJMSSC54          0.059      0.024      2.428      0.015

 RVCSTM36 ON
   MS_24MR           2.214      0.478      4.636      0.000

 RELSTM36 ON
   MS_24MR           1.673      0.496      3.375      0.001

 PLSASC54 ON
   CSEX_M01          1.409      0.838      1.681      0.093
   CRACEM01          1.787      0.932      1.917      0.055
   MEDUCM01          0.776      0.205      3.787      0.000
   TEMP_M06          0.020      1.077      0.019      0.985
   MDI15O15          0.062      0.034      1.854      0.064
   INCNTM01          0.550      0.189      2.917      0.004
   MADEPM01         -0.051      0.045     -1.147      0.251
   RVCSTM36          0.744      0.036     20.697      0.000
   RELSTM36          0.080      0.036      2.208      0.027

 PLSESC54 ON
   CSEX_M01          0.444      0.889      0.500      0.617
   CRACEM01          2.227      1.036      2.151      0.032
   MEDUCM01          0.841      0.220      3.824      0.000
   TEMP_M06         -1.729      1.202     -1.439      0.150
```

MDI15O15	0.126	0.036	3.495	0.000
INCNTM01	0.193	0.177	1.090	0.276
MADEPM01	0.008	0.054	0.144	0.886
RVCSTM36	0.636	0.039	16.284	0.000
RELSTM36	0.181	0.039	4.635	0.000

CPINCC54 ON

CSEX_M01	-8.241	1.228	-6.712	0.000
CRACEM01	-3.118	1.350	-2.310	0.021
MEDUCM01	-0.805	0.293	-2.744	0.006
TEMP_M06	0.872	1.669	0.522	0.601
MDI15O15	-0.106	0.057	-1.858	0.063
INCNTM01	-0.040	0.267	-0.150	0.881
MADEPM01	0.036	0.080	0.456	0.648
RVCSTM36	-0.322	0.056	-5.705	0.000
RELSTM36	0.109	0.058	1.894	0.058

CPOMSC54 ON

CSEX_M01	-0.161	0.455	-0.353	0.724
CRACEM01	-0.291	0.504	-0.578	0.563
MEDUCM01	-0.049	0.107	-0.459	0.647
TEMP_M06	-0.161	0.594	-0.271	0.786
MDI15O15	-0.032	0.019	-1.712	0.087
INCNTM01	0.020	0.091	0.224	0.823
MADEPM01	0.073	0.028	2.582	0.010
RVCSTM36	-0.147	0.022	-6.782	0.000
RELSTM36	0.000	0.020	0.001	0.999

WJMSSC54 ON

CSEX_M01	-1.901	0.972	-1.957	0.050
CRACEM01	-0.593	0.904	-0.656	0.512
MEDUCM01	0.240	0.233	1.028	0.304
TEMP_M06	1.051	1.222	0.860	0.390
MDI15O15	0.151	0.040	3.821	0.000
INCNTM01	0.206	0.228	0.902	0.367
MADEPM01	-0.113	0.058	-1.950	0.051
RVCSTM36	0.531	0.042	12.608	0.000
RELSTM36	0.077	0.043	1.777	0.076

RVCSTM36 ON

CSEX_M01	3.959	0.728	5.440	0.000
CRACEM01	3.915	0.842	4.650	0.000
MEDUCM01	1.993	0.160	12.420	0.000
TEMP_M06	-1.512	0.974	-1.552	0.121
MDI15O15	0.389	0.026	14.697	0.000
INCNTM01	0.657	0.168	3.903	0.000
MADEPM01	-0.068	0.045	-1.531	0.126

RELSTM36 ON

CSEX_M01	3.265	0.797	4.098	0.000
CRACEM01	2.065	0.827	2.497	0.013
MEDUCM01	1.429	0.188	7.601	0.000
TEMP_M06	-0.751	1.016	-0.739	0.460
MDI15O15	0.235	0.029	7.997	0.000

```
   INCNTM01           0.112      0.178      0.627      0.530
   MADEPM01          -0.051      0.045     -1.127      0.260

 MS_RI    WITH
   SR                 0.023      0.007      3.184      0.001
   MATH               0.998      0.203      4.923      0.000
   READ               1.158      0.212      5.463      0.000

 SR       WITH
   MATH               1.007      0.171      5.890      0.000
   READ               0.829      0.162      5.117      0.000

 READ     WITH
   MATH              74.679      6.392     11.683      0.000

 MS_54MR  WITH
   PLSASC54           0.697      0.412      1.690      0.091
   PLSESC54           0.705      0.450      1.565      0.118
   CPINCC54          -1.839      0.807     -2.278      0.023
   CPOMSC54          -1.031      0.238     -4.323      0.000
   WJMSSC54          -0.271      0.479     -0.567      0.571

 MS_36MR  WITH
   RVCSTM36           1.909      0.342      5.582      0.000
   RELSTM36           0.965      0.398      2.427      0.015

 PLSASC54 WITH
   PLSESC54          66.966      6.234     10.741      0.000
   CPINCC54         -32.040      7.559     -4.239      0.000
   CPOMSC54          -5.187      2.964     -1.750      0.080
   WJMSSC54          34.040      6.309      5.395      0.000

 PLSESC54 WITH
   CPINCC54         -37.700      8.870     -4.250      0.000
   CPOMSC54         -10.682      3.428     -3.116      0.002
   WJMSSC54          49.497      7.251      6.826      0.000

 CPINCC54 WITH
   CPOMSC54          18.986      3.885      4.887      0.000
   WJMSSC54         -11.477      8.228     -1.395      0.163

 CPOMSC54 WITH
   WJMSSC54          -5.263      3.791     -1.388      0.165

 RVCSTM36 WITH
   RELSTM36          68.818      5.413     12.715      0.000

 CSEX_M01 WITH
   CRACEM01           0.000      0.007      0.066      0.948
   MEDUCM01           0.046      0.034      1.339      0.180
   TEMP_M06           0.009      0.006      1.598      0.110
   MDI15O15           0.891      0.202      4.403      0.000
   INCNTM01           0.015      0.039      0.394      0.694
   MADEPM01          -0.197      0.127     -1.557      0.119
```

CRACEM01 WITH

MEDUCM01	0.083	0.034	2.448	0.014
TEMP_M06	-0.028	0.006	-4.858	0.000
MDI15O15	1.114	0.192	5.796	0.000
INCNTM01	0.218	0.040	5.421	0.000
MADEPM01	-0.314	0.151	-2.074	0.038

MEDUCM01 WITH

TEMP_M06	-0.143	0.029	-4.872	0.000
MDI15O15	5.153	1.042	4.946	0.000
INCNTM01	2.762	0.204	13.526	0.000
MADEPM01	-5.235	0.670	-7.818	0.000

TEMP_M06 WITH

MDI15O15	-0.409	0.163	-2.505	0.012
INCNTM01	-0.163	0.037	-4.373	0.000
MADEPM01	0.871	0.101	8.626	0.000

MDI15O15 WITH

INCNTM01	4.490	1.165	3.856	0.000
MADEPM01	-11.465	3.775	-3.037	0.002

INCNTM01 WITH

MADEPM01	-4.124	0.629	-6.552	0.000

Means

CSEX_M01	1.483	0.014	109.614	0.000
CRACEM01	3.875	0.014	281.867	0.000
MEDUCM01	14.234	0.068	209.226	0.000
TEMP_M06	3.181	0.011	281.720	0.000
MDI15O15	108.214	0.409	264.839	0.000
INCNTM01	2.845	0.074	38.384	0.000
MADEPM01	11.364	0.257	44.232	0.000

Intercepts

NONDISTRES	1.606	0.276	5.814	0.000
POSREGARD6	3.139	0.177	17.693	0.000
INTRUSIVEN	3.416	0.237	14.394	0.000
NONDISTRES	1.738	0.277	6.274	0.000
POSREGARD1	2.704	0.223	12.118	0.000
INTRUSIVEN	3.369	0.291	11.567	0.000
NONDISTRES	1.701	0.278	6.109	0.000
POSREGARD2	2.467	0.228	10.799	0.000
INTRUSIVEN	3.847	0.253	15.212	0.000
SPTSIO36	1.699	0.278	6.109	0.000
RSCSIO36	2.223	0.265	8.371	0.000
HOSTILITY3	4.820	0.207	23.254	0.000
SPTSIO54	1.570	0.279	5.630	0.000
RSCSIO54	2.160	0.257	8.419	0.000
HOSTILITY5	4.436	0.225	19.752	0.000
SPTSIO1S	0.894	0.331	2.702	0.007
RSCSIO1S	1.507	0.301	5.006	0.000
HOSTILITYG	4.090	0.234	17.461	0.000

SPTSIOG3	1.290	0.276	4.676	0.000
RSCSIOG3	1.664	0.264	6.299	0.000
HOSTILITYG	4.793	0.191	25.106	0.000
SPTSIOG5	1.487	0.278	5.346	0.000
RSCSIOG5	1.870	0.259	7.222	0.000
HOSTILITYG	4.011	0.235	17.042	0.000
MRASIOX5	1.431	0.279	5.137	0.000
MWRSIOX5	7.015	0.556	12.622	0.000
MHISIOX5	14.206	0.728	19.517	0.000
SLFR_O1S	4.102	0.189	21.660	0.000
SLFR_OG3	3.476	0.192	18.080	0.000
SLFR_OG5	3.895	0.181	21.539	0.000
SLFRLCX5	2.669	0.181	14.773	0.000
WJAPSC1S	46.865	5.389	8.696	0.000
WJAPSCG3	67.451	5.149	13.100	0.000
WJAPSCG5	65.788	5.209	12.629	0.000
WJAPSCX5	60.081	5.256	11.432	0.000
WJPCSCG3	56.100	5.039	11.133	0.000
WJPCSCG5	50.797	5.148	9.867	0.000
WJPCSCX5	52.626	5.074	10.371	0.000
RVCSTM36	9.430	5.658	1.667	0.096
RELSTM36	40.308	6.162	6.542	0.000
PLSASC54	-10.627	6.480	-1.640	0.101
PLSESC54	-9.835	7.234	-1.360	0.174
CPINCC54	79.913	10.346	7.724	0.000
CPOMSC54	28.922	3.646	7.933	0.000
WJMSSC54	14.595	7.383	1.977	0.048

Variances

CSEX_M01	0.250	0.000	547.251	0.000
CRACEM01	0.258	0.020	13.026	0.000
MEDUCM01	6.308	0.239	26.346	0.000
TEMP_M06	0.163	0.007	22.737	0.000
MDI15O15	200.160	8.481	23.600	0.000
INCNTM01	7.185	0.727	9.889	0.000
MADEPM01	81.717	4.465	18.303	0.000
MS_6MR	1.142	0.081	14.080	0.000
MS_24MR	0.847	0.076	11.193	0.000

Residual Variances

NONDISTRES	0.013	0.053	0.237	0.813
POSREGARD6	0.950	0.037	25.560	0.000
INTRUSIVEN	1.028	0.047	21.969	0.000
NONDISTRES	0.369	0.054	6.881	0.000
POSREGARD1	0.936	0.040	23.169	0.000
INTRUSIVEN	0.949	0.052	18.380	0.000
NONDISTRES	0.330	0.053	6.249	0.000
POSREGARD2	0.842	0.039	21.674	0.000
INTRUSIVEN	1.039	0.055	18.745	0.000
SPTSIO36	0.542	0.045	12.042	0.000
RSCSIO36	0.384	0.029	13.262	0.000
HOSTILITY3	0.382	0.030	12.691	0.000
SPTSIO54	0.471	0.038	12.334	0.000
RSCSIO54	0.343	0.029	11.647	0.000

HOSTILITY5	0.371	0.027	13.509	0.000
SPTSIO1S	0.582	0.053	11.004	0.000
RSCSIO1S	0.361	0.036	9.943	0.000
HOSTILITYG	0.461	0.032	14.493	0.000
SPTSIOG3	0.181	0.027	6.777	0.000
RSCSIOG3	0.309	0.023	13.245	0.000
HOSTILITYG	0.499	0.044	11.274	0.000
SPTSIOG5	0.095	0.017	5.603	0.000
RSCSIOG5	0.285	0.018	15.425	0.000
HOSTILITYG	0.405	0.027	14.909	0.000
MRASIOX5	0.150	0.028	5.454	0.000
MWRSIOX5	5.157	0.262	19.710	0.000
MHISIOX5	1.893	0.169	11.218	0.000
SLFR_O1S	0.000	0.000	999.000	999.000
SLFR_OG3	0.000	0.000	999.000	999.000
SLFR_OG5	0.000	0.000	999.000	999.000
SLFRLCX5	0.000	0.000	999.000	999.000
WJAPSC1S	0.000	0.000	999.000	999.000
WJAPSCG3	0.000	0.000	999.000	999.000
WJAPSCG5	0.000	0.000	999.000	999.000
WJAPSCX5	0.000	0.000	999.000	999.000
WJPCSCG3	0.000	0.000	999.000	999.000
WJPCSCG5	0.000	0.000	999.000	999.000
WJPCSCX5	0.000	0.000	999.000	999.000
RVCSTM36	148.668	6.292	23.629	0.000
RELSTM36	171.860	7.179	23.938	0.000
PLSASC54	174.475	7.140	24.435	0.000
PLSESC54	193.066	7.902	24.433	0.000
CPINCC54	383.440	40.658	9.431	0.000
CPOMSC54	49.948	2.640	18.923	0.000
WJMSSC54	230.021	11.051	20.814	0.000
MS_6M	0.000	0.000	999.000	999.000
MS_15M	0.000	0.000	999.000	999.000
MS_24M	0.000	0.000	999.000	999.000
MS_36M	0.000	0.000	999.000	999.000
MS_54M	0.000	0.000	999.000	999.000
MS_G1	0.000	0.000	999.000	999.000
MS_G3	0.000	0.000	999.000	999.000
MS_G5	0.000	0.000	999.000	999.000
MS_15	0.000	0.000	999.000	999.000
MS_RI	0.216	0.016	13.614	0.000
MS_15MR	0.593	0.060	9.812	0.000
MS_36MR	0.538	0.044	12.132	0.000
MS_54MR	0.644	0.051	12.686	0.000
MS_G1R	0.644	0.050	12.911	0.000
MS_G3R	0.517	0.038	13.533	0.000
MS_G5R	0.452	0.034	13.417	0.000
MS_15R	0.900	0.065	13.920	0.000
SR	0.036	0.012	2.916	0.004
SLFR_G1R	1.102	0.051	21.761	0.000
SLFR_G3R	0.790	0.035	22.373	0.000
SLFR_G5R	0.684	0.035	19.420	0.000
SLFR_15R	0.111	0.014	7.726	0.000
READ	97.529	7.420	13.144	0.000

```
    WJPCSCG3_R        62.951      3.983     15.806      0.000
    WJPCSCG5_R        32.110      8.138      3.946      0.000
    WJPCSCX5_R        67.567     10.220      6.611      0.000
    MATH              87.502      9.620      9.096      0.000
    WJAPSC1S_R       109.321      6.362     17.183      0.000
    WJAPSCG3_R        78.881      4.720     16.712      0.000
    WJAPSCG5_R        47.466      5.148      9.221      0.000
    WJAPSCX5_R        55.840      6.698      8.337      0.000


QUALITY OF NUMERICAL RESULTS

     Condition Number for the Information Matrix              0.229E-08
       (ratio of smallest to largest eigenvalue)


STANDARDIZED MODEL RESULTS


STDYX Standardization

                                                    Two-Tailed
                    Estimate       S.E.  Est./S.E.    P-Value

 MS_6M    BY
    NONDISTRES         0.996      0.017     60.025      0.000
    POSREGARD6         0.558      0.021     26.111      0.000
    INTRUSIVEN         0.666      0.017     38.301      0.000

 MS_15M   BY
    NONDISTRES         0.861      0.022     39.969      0.000
    POSREGARD1         0.548      0.025     22.192      0.000
    INTRUSIVEN         0.645      0.022     28.885      0.000

 MS_24M   BY
    NONDISTRES         0.893      0.018     48.362      0.000
    POSREGARD2         0.653      0.020     32.603      0.000
    INTRUSIVEN         0.587      0.024     24.101      0.000

 MS_36M   BY
    SPTSIO36           0.809      0.015     53.406      0.000
    RSCSIO36           0.814      0.015     52.560      0.000
    HOSTILITY3         0.636      0.024     26.202      0.000

 MS_54M   BY
    SPTSIO54           0.844      0.013     65.503      0.000
    RSCSIO54           0.844      0.016     52.155      0.000
    HOSTILITY5         0.725      0.021     35.207      0.000

 MS_G1    BY
    SPTSIO1S           0.824      0.016     50.612      0.000
    RSCSIO1S           0.852      0.017     50.336      0.000
    HOSTILITYG         0.674      0.023     28.703      0.000
```

```
MS_G3    BY
   SPTSIOG3           0.920      0.012     74.350      0.000
   RSCSIOG3           0.844      0.014     59.244      0.000
   HOSTILITYG         0.538      0.026     20.551      0.000

MS_G5    BY
   SPTSIOG5           0.952      0.009    106.773      0.000
   RSCSIOG5           0.838      0.013     66.027      0.000
   HOSTILITYG         0.706      0.020     35.078      0.000

MS_15    BY
   MRASIOX5           0.949      0.010     99.099      0.000
   MWRSIOX5           0.659      0.021     31.365      0.000
   MHISIOX5          -0.887      0.010    -84.990      0.000

SR       BY
   SLFR_O1S           0.212      0.027      7.953      0.000
   SLFR_OG3           0.246      0.030      8.297      0.000
   SLFR_OG5           0.261      0.033      7.982      0.000
   SLFRLCX5           0.567      0.065      8.693      0.000

SLFR_G1R BY
   SLFR_O1S           0.972      0.006    159.985      0.000

SLFR_G3R BY
   SLFR_OG3           0.965      0.008    124.189      0.000

SLFR_G5R BY
   SLFR_OG5           0.964      0.009    108.534      0.000

SLFR_15R BY
   SLFRLCX5           0.824      0.045     18.413      0.000

READ     BY
   WJPCSCG3           0.822      0.012     70.218      0.000
   WJPCSCG5           0.900      0.025     35.803      0.000
   WJPCSCX5           0.802      0.016     49.424      0.000

WJPCSCG3 BY
   WJPCSCG3           0.556      0.017     32.904      0.000

WJPCSCG5 BY
   WJPCSCG5           0.439      0.051      8.668      0.000

WJPCSCX5 BY
   WJPCSCX5           0.597      0.022     27.330      0.000

MATH     BY
   WJAPSC1S           0.662      0.026     25.137      0.000
   WJAPSCG3           0.758      0.025     30.472      0.000
   WJAPSCG5           0.839      0.028     30.253      0.000
   WJAPSCX5           0.814      0.027     30.227      0.000

WJAPSC1S BY
```

```
    WJAPSC1S           0.671      0.023     28.705      0.000

 WJAPSCG3 BY
    WJAPSCG3           0.625      0.026     24.456      0.000

 WJAPSCG5 BY
    WJAPSCG5           0.535      0.040     13.344      0.000

 WJAPSCX5 BY
    WJAPSCX5           0.578      0.036     15.889      0.000

 MS_RI    BY
    MS_6M              0.534      0.018     29.116      0.000
    MS_15M             0.658      0.022     29.913      0.000
    MS_24M             0.592      0.021     28.033      0.000
    MS_36M             0.668      0.019     35.835      0.000
    MS_54M             0.626      0.019     33.411      0.000
    MS_G1              0.608      0.020     30.612      0.000
    MS_G3              0.676      0.017     38.627      0.000
    MS_G5              0.706      0.017     41.728      0.000
    MS_15              0.577      0.017     33.662      0.000

 MS_6MR   BY
    MS_6M              0.845      0.012     72.834      0.000

 MS_15MR  BY
    MS_15M             0.753      0.019     39.200      0.000

 MS_24MR  BY
    MS_24M             0.806      0.015     52.047      0.000

 MS_36MR  BY
    MS_36M             0.745      0.017     44.591      0.000

 MS_54MR  BY
    MS_54M             0.780      0.015     51.957      0.000

 MS_G1R   BY
    MS_G1              0.756      0.016     46.987      0.000

 MS_G3R   BY
    MS_G3              0.730      0.016     46.715      0.000

 MS_G5R   BY
    MS_G5              0.707      0.017     42.148      0.000

 MS_15R   BY
    MS_15              0.817      0.012     67.460      0.000

 MS_15MR  ON
    MS_6MR             0.089      0.040      2.229      0.026

 MS_36MR  ON
    MS_24MR            0.228      0.049      4.675      0.000
```

```
MS_54MR  ON
    MS_36MR          0.306      0.052      5.826      0.000

MS_G1R   ON
    MS_54MR          0.237      0.060      3.920      0.000

MS_G3R   ON
    MS_G1R           0.172      0.051      3.368      0.001

SLFR_G3R ON
    SLFR_G1R         0.195      0.036      5.434      0.000
    WJAPSC1S_R       0.084      0.038      2.232      0.026

WJPCSCG3 ON
    WJAPSC1S_R       0.122      0.053      2.284      0.022

WJAPSCG3 ON
    WJAPSC1S_R       0.296      0.051      5.858      0.000

MS_G5R   ON
    MS_G3R           0.120      0.053      2.273      0.023

SLFR_G5R ON
    SLFR_G3R         0.265      0.035      7.617      0.000
    WJPCSCG3_R       0.015      0.040      0.374      0.708

WJPCSCG5 ON
    WJAPSCG3_R      -0.142      0.054     -2.643      0.008
    WJPCSCG3_R       0.118      0.078      1.515      0.130

WJAPSCG5 ON
    WJAPSCG3_R       0.281      0.064      4.400      0.000
    WJPCSCG3_R       0.031      0.063      0.493      0.622

MS_15R   ON
    MS_G5R           0.127      0.047      2.698      0.007

SLFR_15R ON
    SLFR_G5R        -0.123      0.059     -2.085      0.037
    WJAPSCG5_R      -0.119      0.053     -2.271      0.023

WJPCSCX5 ON
    SLFR_G5R         0.152      0.048      3.189      0.001
    WJPCSCG5_R      -0.316      0.151     -2.092      0.036
    WJAPSCG5_R      -0.062      0.086     -0.721      0.471

WJAPSCX5 ON
    SLFR_G5R         0.138      0.043      3.188      0.001
    WJPCSCG5_R      -0.324      0.087     -3.717      0.000
    WJAPSCG5_R       0.060      0.106      0.565      0.572

MS_RI    ON
    CSEX_M01         0.052      0.025      2.034      0.042
```

```
    CRACEM01           0.185      0.029      6.320      0.000
    MEDUCM01           0.484      0.027     17.992      0.000
    TEMP_M06          -0.060      0.026     -2.337      0.019
    MDI15O15           0.185      0.026      7.140      0.000
    INCNTM01           0.142      0.031      4.606      0.000
    MADEPM01          -0.124      0.028     -4.478      0.000

 SR       ON
    CSEX_M01           0.294      0.059      4.960      0.000
    CRACEM01          -0.042      0.050     -0.851      0.395
    MEDUCM01           0.267      0.058      4.587      0.000
    TEMP_M06          -0.070      0.053     -1.312      0.189
    MDI15O15           0.220      0.054      4.099      0.000
    INCNTM01           0.147      0.057      2.592      0.010
    MADEPM01          -0.045      0.055     -0.820      0.412

 MATH     ON
    CSEX_M01          -0.119      0.030     -3.974      0.000
    CRACEM01           0.086      0.035      2.437      0.015
    MEDUCM01           0.338      0.030     11.166      0.000
    TEMP_M06          -0.073      0.030     -2.443      0.015
    MDI15O15           0.269      0.031      8.709      0.000
    INCNTM01           0.093      0.035      2.705      0.007
    MADEPM01          -0.046      0.032     -1.450      0.147

 READ     ON
    CSEX_M01           0.036      0.028      1.273      0.203
    CRACEM01           0.102      0.029      3.474      0.001
    MEDUCM01           0.367      0.031     11.702      0.000
    TEMP_M06          -0.032      0.028     -1.146      0.252
    MDI15O15           0.243      0.029      8.300      0.000
    INCNTM01           0.075      0.031      2.391      0.017
    MADEPM01          -0.040      0.030     -1.349      0.177

 MS_G1R   ON
    PLSASC54           0.159      0.040      4.011      0.000

 SLFR_G1R ON
    CPOMSC54          -0.171      0.034     -4.951      0.000

 WJAPSC1S ON
    PLSASC54           0.204      0.046      4.411      0.000
    PLSESC54           0.102      0.049      2.098      0.036
    CPOMSC54          -0.121      0.033     -3.676      0.000
    WJMSSC54           0.095      0.039      2.441      0.015

 RVCSTM36 ON
    MS_24MR            0.129      0.027      4.721      0.000

 RELSTM36 ON
    MS_24MR            0.106      0.031      3.414      0.001

 PLSASC54 ON
    CSEX_M01           0.035      0.021      1.681      0.093
```

```
    CRACEM01           0.045      0.024      1.928      0.054
    MEDUCM01           0.098      0.026      3.796      0.000
    TEMP_M06           0.000      0.022      0.019      0.985
    MDI15O15           0.044      0.024      1.854      0.064
    INCNTM01           0.074      0.025      2.949      0.003
    MADEPM01          -0.023      0.020     -1.143      0.253
    RVCSTM36           0.592      0.028     21.089      0.000
    RELSTM36           0.059      0.026      2.218      0.027

 PLSESC54 ON
    CSEX_M01           0.011      0.022      0.500      0.617
    CRACEM01           0.057      0.026      2.150      0.032
    MEDUCM01           0.106      0.028      3.833      0.000
    TEMP_M06          -0.035      0.024     -1.439      0.150
    MDI15O15           0.089      0.025      3.485      0.000
    INCNTM01           0.026      0.024      1.097      0.273
    MADEPM01           0.004      0.024      0.144      0.886
    RVCSTM36           0.504      0.031     16.263      0.000
    RELSTM36           0.132      0.028      4.680      0.000

 CPINCC54 ON
    CSEX_M01          -0.193      0.025     -7.576      0.000
    CRACEM01          -0.074      0.031     -2.388      0.017
    MEDUCM01          -0.095      0.033     -2.840      0.005
    TEMP_M06           0.016      0.032      0.522      0.602
    MDI15O15          -0.070      0.038     -1.852      0.064
    INCNTM01          -0.005      0.034     -0.150      0.881
    MADEPM01           0.015      0.034      0.458      0.647
    RVCSTM36          -0.239      0.040     -5.987      0.000
    RELSTM36           0.074      0.039      1.911      0.056

 CPOMSC54 ON
    CSEX_M01          -0.011      0.030     -0.353      0.724
    CRACEM01          -0.019      0.033     -0.580      0.562
    MEDUCM01          -0.016      0.035     -0.458      0.647
    TEMP_M06          -0.009      0.032     -0.271      0.786
    MDI15O15          -0.059      0.035     -1.716      0.086
    INCNTM01           0.007      0.032      0.224      0.823
    MADEPM01           0.086      0.033      2.587      0.010
    RVCSTM36          -0.307      0.044     -7.021      0.000
    RELSTM36           0.000      0.038      0.001      0.999

 WJMSSC54 ON
    CSEX_M01          -0.051      0.026     -1.954      0.051
    CRACEM01          -0.016      0.025     -0.658      0.511
    MEDUCM01           0.032      0.031      1.032      0.302
    TEMP_M06           0.023      0.027      0.862      0.389
    MDI15O15           0.115      0.030      3.825      0.000
    INCNTM01           0.030      0.033      0.911      0.362
    MADEPM01          -0.055      0.028     -1.954      0.051
    RVCSTM36           0.454      0.036     12.735      0.000
    RELSTM36           0.061      0.034      1.778      0.075

 RVCSTM36 ON
```

```
   CSEX_M01          0.125      0.023      5.474      0.000
   CRACEM01          0.125      0.026      4.808      0.000
   MEDUCM01          0.316      0.025     12.541      0.000
   TEMP_M06         -0.039      0.025     -1.558      0.119
   MDI15O15          0.347      0.023     15.277      0.000
   INCNTM01          0.111      0.028      3.934      0.000
   MADEPM01         -0.039      0.025     -1.533      0.125

 RELSTM36 ON
   CSEX_M01          0.112      0.027      4.135      0.000
   CRACEM01          0.072      0.029      2.505      0.012
   MEDUCM01          0.246      0.032      7.716      0.000
   TEMP_M06         -0.021      0.028     -0.739      0.460
   MDI15O15          0.228      0.028      8.124      0.000
   INCNTM01          0.021      0.032      0.634      0.526
   MADEPM01         -0.032      0.028     -1.127      0.260

 MS_RI    WITH
   SR                0.266      0.093      2.870      0.004
   MATH              0.230      0.042      5.421      0.000
   READ              0.252      0.044      5.804      0.000

 SR       WITH
   MATH              0.566      0.116      4.875      0.000
   READ              0.441      0.095      4.644      0.000

 READ     WITH
   MATH              0.808      0.040     20.305      0.000

 MS_54MR  WITH
   PLSASC54          0.066      0.039      1.695      0.090
   PLSESC54          0.063      0.040      1.577      0.115
   CPINCC54         -0.117      0.050     -2.319      0.020
   CPOMSC54         -0.182      0.040     -4.493      0.000
   WJMSSC54         -0.022      0.039     -0.567      0.571

 MS_36MR  WITH
   RVCSTM36          0.213      0.036      5.951      0.000
   RELSTM36          0.100      0.041      2.472      0.013

 PLSASC54 WITH
   PLSESC54          0.365      0.028     13.057      0.000
   CPINCC54         -0.124      0.028     -4.450      0.000
   CPOMSC54         -0.056      0.032     -1.762      0.078
   WJMSSC54          0.170      0.030      5.603      0.000

 PLSESC54 WITH
   CPINCC54         -0.139      0.029     -4.739      0.000
   CPOMSC54         -0.109      0.034     -3.173      0.002
   WJMSSC54          0.235      0.031      7.505      0.000

 CPINCC54 WITH
   CPOMSC54          0.137      0.031      4.470      0.000
   WJMSSC54         -0.039      0.028     -1.403      0.161
```

CPOMSC54 WITH

WJMSSC54	-0.049	0.035	-1.397	0.162

RVCSTM36 WITH

RELSTM36	0.431	0.025	17.138	0.000

CSEX_M01 WITH

CRACEM01	0.002	0.027	0.066	0.948
MEDUCM01	0.036	0.027	1.340	0.180
TEMP_M06	0.045	0.028	1.602	0.109
MDI15O15	0.126	0.028	4.461	0.000
INCNTM01	0.011	0.029	0.395	0.692
MADEPM01	-0.044	0.028	-1.564	0.118

CRACEM01 WITH

MEDUCM01	0.065	0.027	2.408	0.016
TEMP_M06	-0.136	0.027	-5.017	0.000
MDI15O15	0.155	0.026	5.886	0.000
INCNTM01	0.160	0.027	5.856	0.000
MADEPM01	-0.068	0.033	-2.069	0.039

MEDUCM01 WITH

TEMP_M06	-0.141	0.028	-4.959	0.000
MDI15O15	0.145	0.028	5.094	0.000
INCNTM01	0.410	0.023	18.163	0.000
MADEPM01	-0.231	0.027	-8.500	0.000

TEMP_M06 WITH

MDI15O15	-0.071	0.028	-2.534	0.011
INCNTM01	-0.150	0.031	-4.817	0.000
MADEPM01	0.238	0.025	9.458	0.000

MDI15O15 WITH

INCNTM01	0.118	0.032	3.716	0.000
MADEPM01	-0.090	0.029	-3.079	0.002

INCNTM01 WITH

MADEPM01	-0.170	0.024	-7.054	0.000

Means

CSEX_M01	2.968	0.024	121.814	0.000
CRACEM01	7.632	0.309	24.685	0.000
MEDUCM01	5.667	0.108	52.424	0.000
TEMP_M06	7.867	0.177	44.346	0.000
MDI15O15	7.649	0.167	45.778	0.000
INCNTM01	1.061	0.040	26.623	0.000
MADEPM01	1.257	0.026	48.378	0.000

Intercepts

NONDISTRES	1.266	0.223	5.670	0.000
POSREGARD6	2.673	0.170	15.681	0.000
INTRUSIVEN	2.513	0.200	12.584	0.000
NONDISTRES	1.457	0.238	6.109	0.000

POSREGARD1	2.338	0.213	10.978	0.000
INTRUSIVEN	2.642	0.269	9.828	0.000
NONDISTRES	1.331	0.223	5.969	0.000
POSREGARD2	2.035	0.204	9.974	0.000
INTRUSIVEN	3.057	0.254	12.033	0.000
SPTSIO36	1.358	0.230	5.905	0.000
RSCSIO36	2.084	0.274	7.610	0.000
HOSTILITY3	6.025	0.523	11.526	0.000
SPTSIO54	1.227	0.225	5.462	0.000
RSCSIO54	1.979	0.257	7.704	0.000
HOSTILITY5	5.016	0.447	11.213	0.000
SPTSIO1S	0.663	0.251	2.648	0.008
RSCSIO1S	1.315	0.278	4.737	0.000
HOSTILITYG	4.452	0.392	11.346	0.000
SPTSIOG3	1.187	0.260	4.560	0.000
RSCSIOG3	1.608	0.270	5.950	0.000
HOSTILITYG	5.722	0.473	12.098	0.000
SPTSIOG5	1.479	0.285	5.181	0.000
RSCSIOG5	1.910	0.282	6.775	0.000
HOSTILITYG	4.463	0.378	11.795	0.000
MRASIOX5	1.161	0.233	4.977	0.000
MWRSIOX5	2.323	0.213	10.923	0.000
MHISIOX5	4.763	0.216	22.053	0.000
SLFR_O1S	3.742	0.188	19.899	0.000
SLFR_OG3	3.686	0.224	16.420	0.000
SLFR_OG5	4.376	0.234	18.711	0.000
SLFRLCX5	6.517	0.537	12.128	0.000
WJAPSC1S	2.751	0.342	8.040	0.000
WJAPSCG3	4.534	0.409	11.078	0.000
WJAPSCG5	4.898	0.452	10.835	0.000
WJAPSCX5	4.341	0.436	9.951	0.000
WJPCSCG3	3.904	0.398	9.815	0.000
WJPCSCG5	3.870	0.469	8.252	0.000
WJPCSCX5	3.572	0.378	9.461	0.000
RVCSTM36	0.595	0.361	1.648	0.099
RELSTM36	2.767	0.443	6.248	0.000
PLSASC54	-0.533	0.323	-1.649	0.099
PLSESC54	-0.492	0.360	-1.367	0.171
CPINCC54	3.740	0.429	8.720	0.000
CPOMSC54	3.795	0.455	8.336	0.000
WJMSSC54	0.786	0.402	1.957	0.050

Variances

CSEX_M01	1.000	0.000	999.000	999.000
CRACEM01	1.000	0.000	999.000	999.000
MEDUCM01	1.000	0.000	999.000	999.000
TEMP_M06	1.000	0.000	999.000	999.000
MDI15O15	1.000	0.000	999.000	999.000
INCNTM01	1.000	0.000	999.000	999.000
MADEPM01	1.000	0.000	999.000	999.000
MS_6MR	1.000	0.000	999.000	999.000
MS_24MR	1.000	0.000	999.000	999.000

Residual Variances

NONDISTRES	0.008	0.033	0.236	0.813
POSREGARD6	0.689	0.024	28.941	0.000
INTRUSIVEN	0.556	0.023	24.014	0.000
NONDISTRES	0.259	0.037	6.987	0.000
POSREGARD1	0.700	0.027	25.858	0.000
INTRUSIVEN	0.583	0.029	20.224	0.000
NONDISTRES	0.202	0.033	6.120	0.000
POSREGARD2	0.573	0.026	21.912	0.000
INTRUSIVEN	0.656	0.029	22.953	0.000
SPTSIO36	0.346	0.024	14.133	0.000
RSCSIO36	0.338	0.025	13.392	0.000
HOSTILITY3	0.596	0.031	19.337	0.000
SPTSIO54	0.288	0.022	13.235	0.000
RSCSIO54	0.288	0.027	10.558	0.000
HOSTILITY5	0.474	0.030	15.880	0.000
SPTSIO1S	0.321	0.027	11.951	0.000
RSCSIO1S	0.275	0.029	9.523	0.000
HOSTILITYG	0.546	0.032	17.269	0.000
SPTSIOG3	0.154	0.023	6.743	0.000
RSCSIOG3	0.288	0.024	12.009	0.000
HOSTILITYG	0.711	0.028	25.240	0.000
SPTSIOG5	0.094	0.017	5.555	0.000
RSCSIOG5	0.297	0.021	13.960	0.000
HOSTILITYG	0.502	0.028	17.661	0.000
MRASIOX5	0.099	0.018	5.424	0.000
MWRSIOX5	0.565	0.028	20.403	0.000
MHISIOX5	0.213	0.019	11.490	0.000
SLFR_O1S	0.000	999.000	999.000	999.000
SLFR_OG3	0.000	999.000	999.000	999.000
SLFR_OG5	0.000	999.000	999.000	999.000
SLFRLCX5	0.000	999.000	999.000	999.000
WJAPSC1S	0.000	999.000	999.000	999.000
WJAPSCG3	0.000	999.000	999.000	999.000
WJAPSCG5	0.000	999.000	999.000	999.000
WJAPSCX5	0.000	999.000	999.000	999.000
WJPCSCG3	0.000	999.000	999.000	999.000
WJPCSCG5	0.000	999.000	999.000	999.000
WJPCSCX5	0.000	999.000	999.000	999.000
RVCSTM36	0.591	0.023	25.414	0.000
RELSTM36	0.810	0.022	36.440	0.000
PLSASC54	0.438	0.019	22.759	0.000
PLSESC54	0.483	0.021	23.232	0.000
CPINCC54	0.840	0.019	44.165	0.000
CPOMSC54	0.860	0.020	42.591	0.000
WJMSSC54	0.668	0.025	26.676	0.000
MS_6M	0.000	999.000	999.000	999.000
MS_15M	0.000	999.000	999.000	999.000
MS_24M	0.000	999.000	999.000	999.000
MS_36M	0.000	999.000	999.000	999.000
MS_54M	0.000	999.000	999.000	999.000
MS_G1	0.000	999.000	999.000	999.000
MS_G3	0.000	999.000	999.000	999.000
MS_G5	0.000	999.000	999.000	999.000
MS_15	0.000	999.000	999.000	999.000

MS_RI	0.472	0.026	18.214	0.000
MS_15MR	0.992	0.007	140.898	0.000
MS_36MR	0.948	0.022	42.743	0.000
MS_54MR	0.907	0.032	28.298	0.000
MS_G1R	0.913	0.032	28.093	0.000
MS_G3R	0.970	0.018	55.149	0.000
MS_G5R	0.986	0.013	77.658	0.000
MS_15R	0.984	0.012	82.732	0.000
SR	0.669	0.080	8.351	0.000
SLFR_G1R	0.971	0.012	82.459	0.000
SLFR_G3R	0.953	0.016	61.448	0.000
SLFR_G5R	0.929	0.018	50.491	0.000
SLFR_15R	0.971	0.020	48.529	0.000
READ	0.699	0.029	24.106	0.000
WJPCSCG3_R	0.985	0.013	76.079	0.000
WJPCSCG5_R	0.967	0.023	42.000	0.000
WJPCSCX5_R	0.874	0.093	9.405	0.000
MATH	0.689	0.027	25.860	0.000
WJAPSC1S_R	0.836	0.026	31.775	0.000
WJAPSCG3_R	0.912	0.030	30.442	0.000
WJAPSCG5_R	0.919	0.037	24.873	0.000
WJAPSCX5_R	0.871	0.064	13.640	0.000

R-SQUARE

Observed Variable	Estimate	S.E.	Est./S.E.	Two-Tailed P-Value
NONDISTR	0.992	0.033	30.013	0.000
POSREGAR	0.311	0.024	13.056	0.000
INTRUSIV	0.444	0.023	19.150	0.000
NONDISTR	0.741	0.037	19.985	0.000
POSREGAR	0.300	0.027	11.096	0.000
INTRUSIV	0.417	0.029	14.443	0.000
NONDISTR	0.798	0.033	24.181	0.000
POSREGAR	0.427	0.026	16.301	0.000
INTRUSIV	0.344	0.029	12.050	0.000
SPTSIO36	0.654	0.024	26.703	0.000
RSCSIO36	0.662	0.025	26.280	0.000
HOSTILIT	0.404	0.031	13.101	0.000
SPTSIO54	0.712	0.022	32.751	0.000
RSCSIO54	0.712	0.027	26.077	0.000
HOSTILIT	0.526	0.030	17.604	0.000
SPTSIO1S	0.679	0.027	25.306	0.000
RSCSIO1S	0.725	0.029	25.168	0.000
HOSTILIT	0.454	0.032	14.352	0.000
SPTSIOG3	0.846	0.023	37.175	0.000
RSCSIOG3	0.712	0.024	29.622	0.000
HOSTILIT	0.289	0.028	10.276	0.000
SPTSIOG5	0.906	0.017	53.387	0.000
RSCSIOG5	0.703	0.021	33.013	0.000
HOSTILIT	0.498	0.028	17.539	0.000
MRASIOX5	0.901	0.018	49.549	0.000

MWRSIOX5	0.435	0.028	15.683	0.000
MHISIOX5	0.787	0.019	42.495	0.000
SLFR_O1S	1.000	999.000	999.000	999.000
SLFR_OG3	1.000	999.000	999.000	999.000
SLFR_OG5	1.000	999.000	999.000	999.000
SLFRLCX5	1.000	999.000	999.000	999.000
WJAPSC1S	1.000	999.000	999.000	999.000
WJAPSCG3	1.000	999.000	999.000	999.000
WJAPSCG5	1.000	999.000	999.000	999.000
WJAPSCX5	1.000	999.000	999.000	999.000
WJPCSCG3	1.000	999.000	999.000	999.000
WJPCSCG5	1.000	999.000	999.000	999.000
WJPCSCX5	1.000	999.000	999.000	999.000
RVCSTM36	0.409	0.023	17.574	0.000
RELSTM36	0.190	0.022	8.552	0.000
PLSASC54	0.562	0.019	29.154	0.000
PLSESC54	0.517	0.021	24.907	0.000
CPINCC54	0.160	0.019	8.409	0.000
CPOMSC54	0.140	0.020	6.923	0.000
WJMSSC54	0.332	0.025	13.261	0.000

Latent Variable	Estimate	S.E.	Est./S.E.	Two-Tailed P-Value
MS_6M	1.000	999.000	999.000	999.000
MS_15M	1.000	999.000	999.000	999.000
MS_24M	1.000	999.000	999.000	999.000
MS_36M	1.000	999.000	999.000	999.000
MS_54M	1.000	999.000	999.000	999.000
MS_G1	1.000	999.000	999.000	999.000
MS_G3	1.000	999.000	999.000	999.000
MS_G5	1.000	999.000	999.000	999.000
MS_15	1.000	999.000	999.000	999.000
MS_RI	0.528	0.026	20.340	0.000
MS_15MR	0.008	0.007	1.114	0.265
MS_36MR	0.052	0.022	2.337	0.019
MS_54MR	0.093	0.032	2.913	0.004
MS_G1R	0.087	0.032	2.678	0.007
MS_G3R	0.030	0.018	1.684	0.092
MS_G5R	0.014	0.013	1.136	0.256
MS_15R	0.016	0.012	1.349	0.177
SR	0.331	0.080	4.125	0.000
SLFR_G1R	0.029	0.012	2.475	0.013
SLFR_G3R	0.047	0.016	3.005	0.003
SLFR_G5R	0.071	0.018	3.837	0.000
SLFR_15R	0.029	0.020	1.469	0.142
READ	0.301	0.029	10.392	0.000
WJPCSCG3	0.015	0.013	1.142	0.254
WJPCSCG5	0.033	0.023	1.437	0.151
WJPCSCX5	0.126	0.093	1.350	0.177
MATH	0.311	0.027	11.683	0.000
WJAPSC1S	0.164	0.026	6.250	0.000
WJAPSCG3	0.088	0.030	2.929	0.003
WJAPSCG5	0.081	0.037	2.184	0.029

```
    WJAPSCX5           0.129      0.064      2.019      0.043


TOTAL, TOTAL INDIRECT, SPECIFIC INDIRECT, AND DIRECT EFFECTS


                                                    Two-Tailed
                    Estimate       S.E.  Est./S.E.    P-Value

Effects from MS_24MR to MS_15R

  Total                0.000      0.000      1.175      0.240
  Total indirect       0.000      0.000      1.175      0.240

  Specific indirect 1
    MS_15R
    MS_G5R
    MS_G3R
    MS_G1R
    PLSASC54
    RVCSTM36
    MS_24MR            0.000      0.000      1.182      0.237

  Specific indirect 2
    MS_15R
    MS_G5R
    MS_G3R
    MS_G1R
    PLSASC54
    RELSTM36
    MS_24MR            0.000      0.000      1.017      0.309

  Specific indirect 3
    MS_15R
    MS_G5R
    MS_G3R
    MS_G1R
    MS_54MR
    MS_36MR
    MS_24MR            0.000      0.000      1.070      0.284


Effects from MS_54MR to MS_15R

  Total                0.001      0.001      1.226      0.220
  Total indirect       0.001      0.001      1.226      0.220

  Specific indirect 1
    MS_15R
    MS_G5R
    MS_G3R
    MS_G1R
    MS_54MR            0.001      0.001      1.226      0.220
```

Effects from PLSASC54 to MS_15R

```
  Total                0.000      0.000      1.268      0.205
  Total indirect       0.000      0.000      1.268      0.205

  Specific indirect 1
    MS_15R
    MS_G5R
    MS_G3R
    MS_G1R
    PLSASC54           0.000      0.000      1.268      0.205
```

Effects from PLSESC54 to MS_15R

```
  Total                0.000      0.000     -2.976      0.003
  Total indirect       0.000      0.000     -2.976      0.003
```

Effects from CPINCC54 to MS_15R

```
  Total                0.000      0.000    999.000      0.000
  Total indirect       0.000      0.000    999.000      0.000
```

Effects from CPOMSC54 to MS_15R

```
  Total                0.000      0.000      2.978      0.003
  Total indirect       0.000      0.000      2.978      0.003
```

Effects from WJMSSC54 to MS_15R

```
  Total                0.000      0.000      0.000      1.000
  Total indirect       0.000      0.000      0.000      1.000
```

Effects from RVCSTM36 to MS_15R

```
  Total                0.000      0.000      1.269      0.204
  Total indirect       0.000      0.000      1.269      0.204

  Specific indirect 1
    MS_15R
    MS_G5R
    MS_G3R
    MS_G1R
    PLSASC54
    RVCSTM36           0.000      0.000      1.269      0.204
```

Effects from RELSTM36 to MS_15R

```
  Total               0.000      0.000      1.068      0.286
  Total indirect      0.000      0.000      1.068      0.286

  Specific indirect 1
    MS_15R
    MS_G5R
    MS_G3R
    MS_G1R
    PLSASC54
    RELSTM36          0.000      0.000      1.068      0.286


Effects from MS_24MR to SLFR_15R

  Total               0.000      0.000     -1.700      0.089
  Total indirect      0.000      0.000     -1.700      0.089

  Specific indirect 1
    SLFR_15R
    SLFR_G5R
    SLFR_G3R
    SLFR_G1R
    CPOMSC54
    RVCSTM36
    MS_24MR           0.000      0.000     -1.832      0.067

  Specific indirect 2
    SLFR_15R
    SLFR_G5R
    SLFR_G3R
    SLFR_G1R
    CPOMSC54
    RELSTM36
    MS_24MR           0.000      0.000      0.001      0.999

  Specific indirect 3
    SLFR_15R
    SLFR_G5R
    SLFR_G3R
    WJAPSC1S
    PLSASC54
    RVCSTM36
    MS_24MR           0.000      0.000     -1.369      0.171

  Specific indirect 4
    SLFR_15R
    SLFR_G5R
    SLFR_G3R
    WJAPSC1S
    PLSASC54
    RELSTM36
    MS_24MR           0.000      0.000     -1.161      0.246

  Specific indirect 5
```

```
  SLFR_15R
  SLFR_G5R
  SLFR_G3R
  WJAPSC1S
  PLSESC54
  RVCSTM36
  MS_24MR          0.000      0.000     -1.169      0.243

Specific indirect 6
  SLFR_15R
  SLFR_G5R
  SLFR_G3R
  WJAPSC1S
  PLSESC54
  RELSTM36
  MS_24MR          0.000      0.000     -1.112      0.266

Specific indirect 7
  SLFR_15R
  SLFR_G5R
  SLFR_G3R
  WJAPSC1S
  CPOMSC54
  RVCSTM36
  MS_24MR          0.000      0.000     -1.348      0.178

Specific indirect 8
  SLFR_15R
  SLFR_G5R
  SLFR_G3R
  WJAPSC1S
  CPOMSC54
  RELSTM36
  MS_24MR          0.000      0.000      0.001      0.999

Specific indirect 9
  SLFR_15R
  SLFR_G5R
  SLFR_G3R
  WJAPSC1S
  WJMSSC54
  RVCSTM36
  MS_24MR          0.000      0.000     -1.267      0.205

Specific indirect 10
  SLFR_15R
  SLFR_G5R
  SLFR_G3R
  WJAPSC1S
  WJMSSC54
  RELSTM36
  MS_24MR          0.000      0.000     -1.034      0.301

Specific indirect 11
```

SLFR_15R
SLFR_G5R
WJPCSCG3
WJAPSC1S
PLSASC54
RVCSTM36
MS_24MR 0.000 0.000 -0.347 0.729

Specific indirect 12
SLFR_15R
SLFR_G5R
WJPCSCG3
WJAPSC1S
PLSASC54
RELSTM36
MS_24MR 0.000 0.000 -0.340 0.734

Specific indirect 13
SLFR_15R
SLFR_G5R
WJPCSCG3
WJAPSC1S
PLSESC54
RVCSTM36
MS_24MR 0.000 0.000 -0.338 0.736

Specific indirect 14
SLFR_15R
SLFR_G5R
WJPCSCG3
WJAPSC1S
PLSESC54
RELSTM36
MS_24MR 0.000 0.000 -0.334 0.738

Specific indirect 15
SLFR_15R
SLFR_G5R
WJPCSCG3
WJAPSC1S
CPOMSC54
RVCSTM36
MS_24MR 0.000 0.000 -0.344 0.731

Specific indirect 16
SLFR_15R
SLFR_G5R
WJPCSCG3
WJAPSC1S
CPOMSC54
RELSTM36
MS_24MR 0.000 0.000 0.001 0.999

Specific indirect 17

SLFR_15R
SLFR_G5R
WJPCSCG3
WJAPSC1S
WJMSSC54
RVCSTM36
MS_24MR 0.000 0.000 -0.344 0.730

Specific indirect 18
SLFR_15R
SLFR_G5R
WJPCSCG3
WJAPSC1S
WJMSSC54
RELSTM36
MS_24MR 0.000 0.000 -0.335 0.738

Specific indirect 19
SLFR_15R
WJAPSCG5
WJPCSCG3
WJAPSC1S
PLSASC54
RVCSTM36
MS_24MR 0.000 0.000 -0.409 0.682

Specific indirect 20
SLFR_15R
WJAPSCG5
WJPCSCG3
WJAPSC1S
PLSASC54
RELSTM36
MS_24MR 0.000 0.000 -0.402 0.688

Specific indirect 21
SLFR_15R
WJAPSCG5
WJPCSCG3
WJAPSC1S
PLSESC54
RVCSTM36
MS_24MR 0.000 0.000 -0.393 0.694

Specific indirect 22
SLFR_15R
WJAPSCG5
WJPCSCG3
WJAPSC1S
PLSESC54
RELSTM36
MS_24MR 0.000 0.000 -0.391 0.696

Specific indirect 23

SLFR_15R
WJAPSCG5
WJPCSCG3
WJAPSC1S
CPOMSC54
RVCSTM36
MS_24MR 0.000 0.000 -0.411 0.681

Specific indirect 24
SLFR_15R
WJAPSCG5
WJPCSCG3
WJAPSC1S
CPOMSC54
RELSTM36
MS_24MR 0.000 0.000 0.001 0.999

Specific indirect 25
SLFR_15R
WJAPSCG5
WJPCSCG3
WJAPSC1S
WJMSSC54
RVCSTM36
MS_24MR 0.000 0.000 -0.399 0.690

Specific indirect 26
SLFR_15R
WJAPSCG5
WJPCSCG3
WJAPSC1S
WJMSSC54
RELSTM36
MS_24MR 0.000 0.000 -0.390 0.697

Specific indirect 27
SLFR_15R
WJAPSCG5
WJAPSCG3
WJAPSC1S
PLSASC54
RVCSTM36
MS_24MR 0.000 0.000 -1.318 0.187

Specific indirect 28
SLFR_15R
WJAPSCG5
WJAPSCG3
WJAPSC1S
PLSASC54
RELSTM36
MS_24MR 0.000 0.000 -1.140 0.254

Specific indirect 29

SLFR_15R
WJAPSCG5
WJAPSCG3
WJAPSC1S
PLSESC54
RVCSTM36
MS_24MR 0.000 0.000 -1.153 0.249

Specific indirect 30
SLFR_15R
WJAPSCG5
WJAPSCG3
WJAPSC1S
PLSESC54
RELSTM36
MS_24MR 0.000 0.000 -1.109 0.267

Specific indirect 31
SLFR_15R
WJAPSCG5
WJAPSCG3
WJAPSC1S
CPOMSC54
RVCSTM36
MS_24MR 0.000 0.000 -1.356 0.175

Specific indirect 32
SLFR_15R
WJAPSCG5
WJAPSCG3
WJAPSC1S
CPOMSC54
RELSTM36
MS_24MR 0.000 0.000 0.001 0.999

Specific indirect 33
SLFR_15R
WJAPSCG5
WJAPSCG3
WJAPSC1S
WJMSSC54
RVCSTM36
MS_24MR 0.000 0.000 -1.289 0.197

Specific indirect 34
SLFR_15R
WJAPSCG5
WJAPSCG3
WJAPSC1S
WJMSSC54
RELSTM36
MS_24MR 0.000 0.000 -1.021 0.307

Effects from MS_54MR to SLFR_15R

Total	0.000	0.000	999.000	0.000
Total indirect	0.000	0.000	999.000	0.000

Effects from PLSASC54 to SLFR_15R

Total	0.000	0.000	-1.592	0.111
Total indirect	0.000	0.000	-1.592	0.111

Specific indirect 1
SLFR_15R
SLFR_G5R
SLFR_G3R
WJAPSC1S
PLSASC54 0.000 0.000 -1.452 0.146

Specific indirect 2
SLFR_15R
SLFR_G5R
WJPCSCG3
WJAPSC1S
PLSASC54 0.000 0.000 -0.348 0.728

Specific indirect 3
SLFR_15R
WJAPSCG5
WJPCSCG3
WJAPSC1S
PLSASC54 0.000 0.000 -0.412 0.681

Specific indirect 4
SLFR_15R
WJAPSCG5
WJAPSCG3
WJAPSC1S
PLSASC54 0.000 0.000 -1.402 0.161

Effects from PLSESC54 to SLFR_15R

Total	0.000	0.000	-1.292	0.196
Total indirect	0.000	0.000	-1.292	0.196

Specific indirect 1
SLFR_15R
SLFR_G5R
SLFR_G3R
WJAPSC1S
PLSESC54 0.000 0.000 -1.211 0.226

Specific indirect 2
SLFR_15R

SLFR_G5R
WJPCSCG3
WJAPSC1S
PLSESC54 0.000 0.000 -0.339 0.735

Specific indirect 3
SLFR_15R
WJAPSCG5
WJPCSCG3
WJAPSC1S
PLSESC54 0.000 0.000 -0.395 0.693

Specific indirect 4
SLFR_15R
WJAPSCG5
WJAPSCG3
WJAPSC1S
PLSESC54 0.000 0.000 -1.202 0.230

Effects from CPINCC54 to SLFR_15R

Total 0.000 0.000 999.000 0.000
Total indirect 0.000 0.000 999.000 0.000

Effects from CPOMSC54 to SLFR_15R

Total 0.000 0.000 2.319 0.020
Total indirect 0.000 0.000 2.319 0.020

Specific indirect 1
SLFR_15R
SLFR_G5R
SLFR_G3R
SLFR_G1R
CPOMSC54 0.000 0.000 2.114 0.034

Specific indirect 2
SLFR_15R
SLFR_G5R
SLFR_G3R
WJAPSC1S
CPOMSC54 0.000 0.000 1.445 0.148

Specific indirect 3
SLFR_15R
SLFR_G5R
WJPCSCG3
WJAPSC1S
CPOMSC54 0.000 0.000 0.346 0.730

Specific indirect 4
SLFR_15R

WJAPSCG5
WJPCSCG3
WJAPSC1S
CPOMSC54 0.000 0.000 0.413 0.679

Specific indirect 5
SLFR_15R
WJAPSCG5
WJAPSCG3
WJAPSC1S
CPOMSC54 0.000 0.000 1.451 0.147

Effects from WJMSSC54 to SLFR_15R

Total 0.000 0.000 -1.501 0.133
Total indirect 0.000 0.000 -1.501 0.133

Specific indirect 1
SLFR_15R
SLFR_G5R
SLFR_G3R
WJAPSC1S
WJMSSC54 0.000 0.000 -1.336 0.182

Specific indirect 2
SLFR_15R
SLFR_G5R
WJPCSCG3
WJAPSC1S
WJMSSC54 0.000 0.000 -0.346 0.729

Specific indirect 3
SLFR_15R
WJAPSCG5
WJPCSCG3
WJAPSC1S
WJMSSC54 0.000 0.000 -0.402 0.688

Specific indirect 4
SLFR_15R
WJAPSCG5
WJAPSCG3
WJAPSC1S
WJMSSC54 0.000 0.000 -1.367 0.172

Effects from RVCSTM36 to SLFR_15R

Total 0.000 0.000 -1.863 0.062
Total indirect 0.000 0.000 -1.863 0.062

Specific indirect 1
SLFR_15R

SLFR_G5R
SLFR_G3R
SLFR_G1R
CPOMSC54
RVCSTM36 0.000 0.000 -2.019 0.044

Specific indirect 2
SLFR_15R
SLFR_G5R
SLFR_G3R
WJAPSC1S
PLSASC54
RVCSTM36 0.000 0.000 -1.449 0.147

Specific indirect 3
SLFR_15R
SLFR_G5R
SLFR_G3R
WJAPSC1S
PLSESC54
RVCSTM36 0.000 0.000 -1.206 0.228

Specific indirect 4
SLFR_15R
SLFR_G5R
SLFR_G3R
WJAPSC1S
CPOMSC54
RVCSTM36 0.000 0.000 -1.421 0.155

Specific indirect 5
SLFR_15R
SLFR_G5R
SLFR_G3R
WJAPSC1S
WJMSSC54
RVCSTM36 0.000 0.000 -1.320 0.187

Specific indirect 6
SLFR_15R
SLFR_G5R
WJPCSCG3
WJAPSC1S
PLSASC54
RVCSTM36 0.000 0.000 -0.348 0.728

Specific indirect 7
SLFR_15R
SLFR_G5R
WJPCSCG3
WJAPSC1S
PLSESC54
RVCSTM36 0.000 0.000 -0.339 0.735

Specific indirect 8
SLFR_15R
SLFR_G5R
WJPCSCG3
WJAPSC1S
CPOMSC54
RVCSTM36 0.000 0.000 -0.346 0.730

Specific indirect 9
SLFR_15R
SLFR_G5R
WJPCSCG3
WJAPSC1S
WJMSSC54
RVCSTM36 0.000 0.000 -0.346 0.730

Specific indirect 10
SLFR_15R
WJAPSCG5
WJPCSCG3
WJAPSC1S
PLSASC54
RVCSTM36 0.000 0.000 -0.412 0.681

Specific indirect 11
SLFR_15R
WJAPSCG5
WJPCSCG3
WJAPSC1S
PLSESC54
RVCSTM36 0.000 0.000 -0.395 0.693

Specific indirect 12
SLFR_15R
WJAPSCG5
WJPCSCG3
WJAPSC1S
CPOMSC54
RVCSTM36 0.000 0.000 -0.413 0.680

Specific indirect 13
SLFR_15R
WJAPSCG5
WJPCSCG3
WJAPSC1S
WJMSSC54
RVCSTM36 0.000 0.000 -0.401 0.689

Specific indirect 14
SLFR_15R
WJAPSCG5
WJAPSCG3
WJAPSC1S
PLSASC54

RVCSTM36 0.000 0.000 -1.394 0.163

Specific indirect 15
SLFR_15R
WJAPSCG5
WJAPSCG3
WJAPSC1S
PLSESC54
RVCSTM36 0.000 0.000 -1.191 0.234

Specific indirect 16
SLFR_15R
WJAPSCG5
WJAPSCG3
WJAPSC1S
CPOMSC54
RVCSTM36 0.000 0.000 -1.435 0.151

Specific indirect 17
SLFR_15R
WJAPSCG5
WJAPSCG3
WJAPSC1S
WJMSSC54
RVCSTM36 0.000 0.000 -1.349 0.177

Effects from RELSTM36 to SLFR_15R

Total 0.000 0.000 -1.365 0.172
Total indirect 0.000 0.000 -1.365 0.172

Specific indirect 1
SLFR_15R
SLFR_G5R
SLFR_G3R
SLFR_G1R
CPOMSC54
RELSTM36 0.000 0.000 0.001 0.999

Specific indirect 2
SLFR_15R
SLFR_G5R
SLFR_G3R
WJAPSC1S
PLSASC54
RELSTM36 0.000 0.000 -1.212 0.226

Specific indirect 3
SLFR_15R
SLFR_G5R
SLFR_G3R
WJAPSC1S
PLSESC54

```
    RELSTM36         0.000      0.000     -1.158      0.247

  Specific indirect 4
    SLFR_15R
    SLFR_G5R
    SLFR_G3R
    WJAPSC1S
    CPOMSC54
    RELSTM36         0.000      0.000      0.001      0.999

  Specific indirect 5
    SLFR_15R
    SLFR_G5R
    SLFR_G3R
    WJAPSC1S
    WJMSSC54
    RELSTM36         0.000      0.000     -1.078      0.281

  Specific indirect 6
    SLFR_15R
    SLFR_G5R
    WJPCSCG3
    WJAPSC1S
    PLSASC54
    RELSTM36         0.000      0.000     -0.342      0.732

  Specific indirect 7
    SLFR_15R
    SLFR_G5R
    WJPCSCG3
    WJAPSC1S
    PLSESC54
    RELSTM36         0.000      0.000     -0.336      0.737

  Specific indirect 8
    SLFR_15R
    SLFR_G5R
    WJPCSCG3
    WJAPSC1S
    CPOMSC54
    RELSTM36         0.000      0.000      0.001      0.999

  Specific indirect 9
    SLFR_15R
    SLFR_G5R
    WJPCSCG3
    WJAPSC1S
    WJMSSC54
    RELSTM36         0.000      0.000     -0.338      0.736

  Specific indirect 10
    SLFR_15R
    WJAPSCG5
    WJPCSCG3
```

WJAPSC1S
PLSASC54
RELSTM36 0.000 0.000 -0.405 0.686

Specific indirect 11
SLFR_15R
WJAPSCG5
WJPCSCG3
WJAPSC1S
PLSESC54
RELSTM36 0.000 0.000 -0.394 0.694

Specific indirect 12
SLFR_15R
WJAPSCG5
WJPCSCG3
WJAPSC1S
CPOMSC54
RELSTM36 0.000 0.000 0.001 0.999

Specific indirect 13
SLFR_15R
WJAPSCG5
WJPCSCG3
WJAPSC1S
WJMSSC54
RELSTM36 0.000 0.000 -0.393 0.694

Specific indirect 14
SLFR_15R
WJAPSCG5
WJAPSCG3
WJAPSC1S
PLSASC54
RELSTM36 0.000 0.000 -1.196 0.232

Specific indirect 15
SLFR_15R
WJAPSCG5
WJAPSCG3
WJAPSC1S
PLSESC54
RELSTM36 0.000 0.000 -1.161 0.245

Specific indirect 16
SLFR_15R
WJAPSCG5
WJAPSCG3
WJAPSC1S
CPOMSC54
RELSTM36 0.000 0.000 0.001 0.999

Specific indirect 17
SLFR_15R

WJAPSCG5
WJAPSCG3
WJAPSC1S
WJMSSC54
RELSTM36 0.000 0.000 -1.069 0.285

Effects from MS_24MR to WJPCSCX5

Total 0.003 0.005 0.592 0.554
Total indirect 0.003 0.005 0.592 0.554

Specific indirect 1
WJPCSCX5
SLFR_G5R
SLFR_G3R
SLFR_G1R
CPOMSC54
RVCSTM36
MS_24MR 0.001 0.000 1.825 0.068

Specific indirect 2
WJPCSCX5
SLFR_G5R
SLFR_G3R
SLFR_G1R
CPOMSC54
RELSTM36
MS_24MR 0.000 0.000 -0.001 0.999

Specific indirect 3
WJPCSCX5
SLFR_G5R
SLFR_G3R
WJAPSC1S
PLSASC54
RVCSTM36
MS_24MR 0.001 0.000 1.443 0.149

Specific indirect 4
WJPCSCX5
SLFR_G5R
SLFR_G3R
WJAPSC1S
PLSASC54
RELSTM36
MS_24MR 0.000 0.000 1.185 0.236

Specific indirect 5
WJPCSCX5
SLFR_G5R
SLFR_G3R
WJAPSC1S
PLSESC54

RVCSTM36
MS_24MR 0.000 0.000 1.219 0.223

Specific indirect 6
WJPCSCX5
SLFR_G5R
SLFR_G3R
WJAPSC1S
PLSESC54
RELSTM36
MS_24MR 0.000 0.000 1.144 0.253

Specific indirect 7
WJPCSCX5
SLFR_G5R
SLFR_G3R
WJAPSC1S
CPOMSC54
RVCSTM36
MS_24MR 0.000 0.000 1.414 0.157

Specific indirect 8
WJPCSCX5
SLFR_G5R
SLFR_G3R
WJAPSC1S
CPOMSC54
RELSTM36
MS_24MR 0.000 0.000 -0.001 0.999

Specific indirect 9
WJPCSCX5
SLFR_G5R
SLFR_G3R
WJAPSC1S
WJMSSC54
RVCSTM36
MS_24MR 0.000 0.000 1.313 0.189

Specific indirect 10
WJPCSCX5
SLFR_G5R
SLFR_G3R
WJAPSC1S
WJMSSC54
RELSTM36
MS_24MR 0.000 0.000 1.041 0.298

Specific indirect 11
WJPCSCX5
SLFR_G5R
WJPCSCG3
WJAPSC1S
PLSASC54

RVCSTM36
MS_24MR 0.000 0.000 0.346 0.729

Specific indirect 12
WJPCSCX5
SLFR_G5R
WJPCSCG3
WJAPSC1S
PLSASC54
RELSTM36
MS_24MR 0.000 0.000 0.338 0.735

Specific indirect 13
WJPCSCX5
SLFR_G5R
WJPCSCG3
WJAPSC1S
PLSESC54
RVCSTM36
MS_24MR 0.000 0.000 0.337 0.736

Specific indirect 14
WJPCSCX5
SLFR_G5R
WJPCSCG3
WJAPSC1S
PLSESC54
RELSTM36
MS_24MR 0.000 0.000 0.333 0.739

Specific indirect 15
WJPCSCX5
SLFR_G5R
WJPCSCG3
WJAPSC1S
CPOMSC54
RVCSTM36
MS_24MR 0.000 0.000 0.343 0.731

Specific indirect 16
WJPCSCX5
SLFR_G5R
WJPCSCG3
WJAPSC1S
CPOMSC54
RELSTM36
MS_24MR 0.000 0.000 -0.001 0.999

Specific indirect 17
WJPCSCX5
SLFR_G5R
WJPCSCG3
WJAPSC1S
WJMSSC54

RVCSTM36
MS_24MR 0.000 0.000 0.343 0.731

Specific indirect 18
WJPCSCX5
SLFR_G5R
WJPCSCG3
WJAPSC1S
WJMSSC54
RELSTM36
MS_24MR 0.000 0.000 0.333 0.739

Specific indirect 19
WJPCSCX5
WJPCSCG5
WJPCSCG3
WJAPSC1S
PLSASC54
RVCSTM36
MS_24MR -0.001 0.000 -1.558 0.119

Specific indirect 20
WJPCSCX5
WJPCSCG5
WJPCSCG3
WJAPSC1S
PLSASC54
RELSTM36
MS_24MR 0.000 0.000 -1.234 0.217

Specific indirect 21
WJPCSCX5
WJPCSCG5
WJPCSCG3
WJAPSC1S
PLSESC54
RVCSTM36
MS_24MR 0.000 0.000 -1.241 0.214

Specific indirect 22
WJPCSCX5
WJPCSCG5
WJPCSCG3
WJAPSC1S
PLSESC54
RELSTM36
MS_24MR 0.000 0.000 -1.178 0.239

Specific indirect 23
WJPCSCX5
WJPCSCG5
WJPCSCG3
WJAPSC1S
CPOMSC54

RVCSTM36
MS_24MR 0.000 0.000 -1.461 0.144

Specific indirect 24
WJPCSCX5
WJPCSCG5
WJPCSCG3
WJAPSC1S
CPOMSC54
RELSTM36
MS_24MR 0.000 0.000 0.001 0.999

Specific indirect 25
WJPCSCX5
WJPCSCG5
WJPCSCG3
WJAPSC1S
WJMSSC54
RVCSTM36
MS_24MR 0.000 0.000 -1.306 0.192

Specific indirect 26
WJPCSCX5
WJPCSCG5
WJPCSCG3
WJAPSC1S
WJMSSC54
RELSTM36
MS_24MR 0.000 0.000 -1.029 0.304

Specific indirect 27
WJPCSCX5
WJPCSCG5
WJAPSCG3
WJAPSC1S
PLSASC54
RVCSTM36
MS_24MR 0.002 0.002 1.206 0.228

Specific indirect 28
WJPCSCX5
WJPCSCG5
WJAPSCG3
WJAPSC1S
PLSASC54
RELSTM36
MS_24MR 0.000 0.000 1.057 0.290

Specific indirect 29
WJPCSCX5
WJPCSCG5
WJAPSCG3
WJAPSC1S
PLSESC54

RVCSTM36
MS_24MR 0.001 0.001 1.051 0.293

Specific indirect 30
WJPCSCX5
WJPCSCG5
WJAPSCG3
WJAPSC1S
PLSESC54
RELSTM36
MS_24MR 0.000 0.000 1.013 0.311

Specific indirect 31
WJPCSCX5
WJPCSCG5
WJAPSCG3
WJAPSC1S
CPOMSC54
RVCSTM36
MS_24MR 0.001 0.000 1.231 0.218

Specific indirect 32
WJPCSCX5
WJPCSCG5
WJAPSCG3
WJAPSC1S
CPOMSC54
RELSTM36
MS_24MR 0.000 0.000 -0.001 0.999

Specific indirect 33
WJPCSCX5
WJPCSCG5
WJAPSCG3
WJAPSC1S
WJMSSC54
RVCSTM36
MS_24MR 0.001 0.001 1.124 0.261

Specific indirect 34
WJPCSCX5
WJPCSCG5
WJAPSCG3
WJAPSC1S
WJMSSC54
RELSTM36
MS_24MR 0.000 0.000 0.947 0.344

Specific indirect 35
WJPCSCX5
WJAPSCG5
WJPCSCG3
WJAPSC1S
PLSASC54

RVCSTM36
MS_24MR 0.000 0.000 -0.511 0.609

Specific indirect 36
WJPCSCX5
WJAPSCG5
WJPCSCG3
WJAPSC1S
PLSASC54
RELSTM36
MS_24MR 0.000 0.000 -0.494 0.621

Specific indirect 37
WJPCSCX5
WJAPSCG5
WJPCSCG3
WJAPSC1S
PLSESC54
RVCSTM36
MS_24MR 0.000 0.000 -0.481 0.631

Specific indirect 38
WJPCSCX5
WJAPSCG5
WJPCSCG3
WJAPSC1S
PLSESC54
RELSTM36
MS_24MR 0.000 0.000 -0.476 0.634

Specific indirect 39
WJPCSCX5
WJAPSCG5
WJPCSCG3
WJAPSC1S
CPOMSC54
RVCSTM36
MS_24MR 0.000 0.000 -0.501 0.616

Specific indirect 40
WJPCSCX5
WJAPSCG5
WJPCSCG3
WJAPSC1S
CPOMSC54
RELSTM36
MS_24MR 0.000 0.000 0.001 0.999

Specific indirect 41
WJPCSCX5
WJAPSCG5
WJPCSCG3
WJAPSC1S
WJMSSC54

RVCSTM36
MS_24MR 0.000 0.000 -0.486 0.627

Specific indirect 42
WJPCSCX5
WJAPSCG5
WJPCSCG3
WJAPSC1S
WJMSSC54
RELSTM36
MS_24MR 0.000 0.000 -0.466 0.641

Specific indirect 43
WJPCSCX5
WJAPSCG5
WJAPSCG3
WJAPSC1S
PLSASC54
RVCSTM36
MS_24MR -0.001 0.001 -0.800 0.424

Specific indirect 44
WJPCSCX5
WJAPSCG5
WJAPSCG3
WJAPSC1S
PLSASC54
RELSTM36
MS_24MR 0.000 0.000 -0.747 0.455

Specific indirect 45
WJPCSCX5
WJAPSCG5
WJAPSCG3
WJAPSC1S
PLSESC54
RVCSTM36
MS_24MR 0.000 0.000 -0.758 0.448

Specific indirect 46
WJPCSCX5
WJAPSCG5
WJAPSCG3
WJAPSC1S
PLSESC54
RELSTM36
MS_24MR 0.000 0.000 -0.747 0.455

Specific indirect 47
WJPCSCX5
WJAPSCG5
WJAPSCG3
WJAPSC1S
CPOMSC54

RVCSTM36
MS_24MR 0.000 0.000 -0.759 0.448

Specific indirect 48
WJPCSCX5
WJAPSCG5
WJAPSCG3
WJAPSC1S
CPOMSC54
RELSTM36
MS_24MR 0.000 0.000 0.001 0.999

Specific indirect 49
WJPCSCX5
WJAPSCG5
WJAPSCG3
WJAPSC1S
WJMSSC54
RVCSTM36
MS_24MR 0.000 0.000 -0.773 0.440

Specific indirect 50
WJPCSCX5
WJAPSCG5
WJAPSCG3
WJAPSC1S
WJMSSC54
RELSTM36
MS_24MR 0.000 0.000 -0.690 0.490

Effects from MS_54MR to WJPCSCX5

Total 0.000 0.000 999.000 0.000
Total indirect 0.000 0.000 999.000 0.000

Effects from PLSASC54 to WJPCSCX5

Total 0.001 0.001 0.492 0.623
Total indirect 0.001 0.001 0.492 0.623

Specific indirect 1
WJPCSCX5
SLFR_G5R
SLFR_G3R
WJAPSC1S
PLSASC54 0.000 0.000 1.543 0.123

Specific indirect 2
WJPCSCX5
SLFR_G5R
WJPCSCG3
WJAPSC1S

PLSASC54 0.000 0.000 0.347 0.729

Specific indirect 3
WJPCSCX5
WJPCSCG5
WJPCSCG3
WJAPSC1S
PLSASC54 0.000 0.000 -1.677 0.094

Specific indirect 4
WJPCSCX5
WJPCSCG5
WJAPSCG3
WJAPSC1S
PLSASC54 0.001 0.001 1.257 0.209

Specific indirect 5
WJPCSCX5
WJAPSCG5
WJPCSCG3
WJAPSC1S
PLSASC54 0.000 0.000 -0.514 0.607

Specific indirect 6
WJPCSCX5
WJAPSCG5
WJAPSCG3
WJAPSC1S
PLSASC54 0.000 0.001 -0.811 0.418

Effects from PLSESC54 to WJPCSCX5

Total 0.000 0.001 0.479 0.632
Total indirect 0.000 0.001 0.479 0.632

Specific indirect 1
WJPCSCX5
SLFR_G5R
SLFR_G3R
WJAPSC1S
PLSESC54 0.000 0.000 1.270 0.204

Specific indirect 2
WJPCSCX5
SLFR_G5R
WJPCSCG3
WJAPSC1S
PLSESC54 0.000 0.000 0.338 0.736

Specific indirect 3
WJPCSCX5
WJPCSCG5
WJPCSCG3

WJAPSC1S
PLSESC54 0.000 0.000 -1.296 0.195

Specific indirect 4
WJPCSCX5
WJPCSCG5
WJAPSCG3
WJAPSC1S
PLSESC54 0.001 0.001 1.081 0.280

Specific indirect 5
WJPCSCX5
WJAPSCG5
WJPCSCG3
WJAPSC1S
PLSESC54 0.000 0.000 -0.483 0.629

Specific indirect 6
WJPCSCX5
WJAPSCG5
WJAPSCG3
WJAPSC1S
PLSESC54 0.000 0.000 -0.765 0.444

Effects from CPINCC54 to WJPCSCX5

Total 0.000 0.000 999.000 0.000
Total indirect 0.000 0.000 999.000 0.000

Effects from CPOMSC54 to WJPCSCX5

Total -0.003 0.002 -1.183 0.237
Total indirect -0.003 0.002 -1.183 0.237

Specific indirect 1
WJPCSCX5
SLFR_G5R
SLFR_G3R
SLFR_G1R
CPOMSC54 -0.002 0.001 -2.100 0.036

Specific indirect 2
WJPCSCX5
SLFR_G5R
SLFR_G3R
WJAPSC1S
CPOMSC54 0.000 0.000 -1.527 0.127

Specific indirect 3
WJPCSCX5
SLFR_G5R
WJPCSCG3

WJAPSC1S
CPOMSC54 0.000 0.000 -0.344 0.731

Specific indirect 4
WJPCSCX5
WJPCSCG5
WJPCSCG3
WJAPSC1S
CPOMSC54 0.001 0.000 1.582 0.114

Specific indirect 5
WJPCSCX5
WJPCSCG5
WJAPSCG3
WJAPSC1S
CPOMSC54 -0.002 0.001 -1.291 0.197

Specific indirect 6
WJPCSCX5
WJAPSCG5
WJPCSCG3
WJAPSC1S
CPOMSC54 0.000 0.000 0.504 0.614

Specific indirect 7
WJPCSCX5
WJAPSCG5
WJAPSCG3
WJAPSC1S
CPOMSC54 0.001 0.001 0.774 0.439

Effects from WJMSSC54 to WJPCSCX5

Total 0.000 0.001 0.491 0.624
Total indirect 0.000 0.001 0.491 0.624

Specific indirect 1
WJPCSCX5
SLFR_G5R
SLFR_G3R
WJAPSC1S
WJMSSC54 0.000 0.000 1.392 0.164

Specific indirect 2
WJPCSCX5
SLFR_G5R
WJPCSCG3
WJAPSC1S
WJMSSC54 0.000 0.000 0.345 0.730

Specific indirect 3
WJPCSCX5
WJPCSCG5

WJPCSCG3
WJAPSC1S
WJMSSC54 0.000 0.000 -1.375 0.169

Specific indirect 4
WJPCSCX5
WJPCSCG5
WJAPSCG3
WJAPSC1S
WJMSSC54 0.001 0.001 1.168 0.243

Specific indirect 5
WJPCSCX5
WJAPSCG5
WJPCSCG3
WJAPSC1S
WJMSSC54 0.000 0.000 -0.488 0.625

Specific indirect 6
WJPCSCX5
WJAPSCG5
WJAPSCG3
WJAPSC1S
WJMSSC54 0.000 0.000 -0.778 0.437

Effects from RVCSTM36 to WJPCSCX5

Total 0.001 0.002 0.608 0.543
Total indirect 0.001 0.002 0.608 0.543

Specific indirect 1
WJPCSCX5
SLFR_G5R
SLFR_G3R
SLFR_G1R
CPOMSC54
RVCSTM36 0.000 0.000 2.008 0.045

Specific indirect 2
WJPCSCX5
SLFR_G5R
SLFR_G3R
WJAPSC1S
PLSASC54
RVCSTM36 0.000 0.000 1.538 0.124

Specific indirect 3
WJPCSCX5
SLFR_G5R
SLFR_G3R
WJAPSC1S
PLSESC54
RVCSTM36 0.000 0.000 1.262 0.207

Specific indirect 4
WJPCSCX5
SLFR_G5R
SLFR_G3R
WJAPSC1S
CPOMSC54
RVCSTM36 0.000 0.000 1.500 0.134

Specific indirect 5
WJPCSCX5
SLFR_G5R
SLFR_G3R
WJAPSC1S
WJMSSC54
RVCSTM36 0.000 0.000 1.373 0.170

Specific indirect 6
WJPCSCX5
SLFR_G5R
WJPCSCG3
WJAPSC1S
PLSASC54
RVCSTM36 0.000 0.000 0.347 0.728

Specific indirect 7
WJPCSCX5
SLFR_G5R
WJPCSCG3
WJAPSC1S
PLSESC54
RVCSTM36 0.000 0.000 0.338 0.736

Specific indirect 8
WJPCSCX5
SLFR_G5R
WJPCSCG3
WJAPSC1S
CPOMSC54
RVCSTM36 0.000 0.000 0.345 0.730

Specific indirect 9
WJPCSCX5
SLFR_G5R
WJPCSCG3
WJAPSC1S
WJMSSC54
RVCSTM36 0.000 0.000 0.344 0.731

Specific indirect 10
WJPCSCX5
WJPCSCG5
WJPCSCG3
WJAPSC1S

PLSASC54
RVCSTM36 0.000 0.000 -1.674 0.094

Specific indirect 11
WJPCSCX5
WJPCSCG5
WJPCSCG3
WJAPSC1S
PLSESC54
RVCSTM36 0.000 0.000 -1.284 0.199

Specific indirect 12
WJPCSCX5
WJPCSCG5
WJPCSCG3
WJAPSC1S
CPOMSC54
RVCSTM36 0.000 0.000 -1.551 0.121

Specific indirect 13
WJPCSCX5
WJPCSCG5
WJPCSCG3
WJAPSC1S
WJMSSC54
RVCSTM36 0.000 0.000 -1.361 0.173

Specific indirect 14
WJPCSCX5
WJPCSCG5
WJAPSCG3
WJAPSC1S
PLSASC54
RVCSTM36 0.001 0.001 1.254 0.210

Specific indirect 15
WJPCSCX5
WJPCSCG5
WJAPSCG3
WJAPSC1S
PLSESC54
RVCSTM36 0.000 0.000 1.075 0.283

Specific indirect 16
WJPCSCX5
WJPCSCG5
WJAPSCG3
WJAPSC1S
CPOMSC54
RVCSTM36 0.000 0.000 1.280 0.201

Specific indirect 17
WJPCSCX5
WJPCSCG5

WJAPSCG3
WJAPSC1S
WJMSSC54
RVCSTM36 0.000 0.000 1.156 0.248

Specific indirect 18
WJPCSCX5
WJAPSCG5
WJPCSCG3
WJAPSC1S
PLSASC54
RVCSTM36 0.000 0.000 -0.514 0.607

Specific indirect 19
WJPCSCX5
WJAPSCG5
WJPCSCG3
WJAPSC1S
PLSESC54
RVCSTM36 0.000 0.000 -0.483 0.629

Specific indirect 20
WJPCSCX5
WJAPSCG5
WJPCSCG3
WJAPSC1S
CPOMSC54
RVCSTM36 0.000 0.000 -0.503 0.615

Specific indirect 21
WJPCSCX5
WJAPSCG5
WJPCSCG3
WJAPSC1S
WJMSSC54
RVCSTM36 0.000 0.000 -0.488 0.626

Specific indirect 22
WJPCSCX5
WJAPSCG5
WJAPSCG3
WJAPSC1S
PLSASC54
RVCSTM36 0.000 0.000 -0.811 0.417

Specific indirect 23
WJPCSCX5
WJAPSCG5
WJAPSCG3
WJAPSC1S
PLSESC54
RVCSTM36 0.000 0.000 -0.765 0.444

Specific indirect 24

WJPCSCX5
WJAPSCG5
WJAPSCG3
WJAPSC1S
CPOMSC54
RVCSTM36 0.000 0.000 -0.768 0.442

Specific indirect 25
WJPCSCX5
WJAPSCG5
WJAPSCG3
WJAPSC1S
WJMSSC54
RVCSTM36 0.000 0.000 -0.781 0.435

Effects from RELSTM36 to WJPCSCX5

Total 0.000 0.000 0.475 0.635
Total indirect 0.000 0.000 0.475 0.635

Specific indirect 1
WJPCSCX5
SLFR_G5R
SLFR_G3R
SLFR_G1R
CPOMSC54
RELSTM36 0.000 0.000 -0.001 0.999

Specific indirect 2
WJPCSCX5
SLFR_G5R
SLFR_G3R
WJAPSC1S
PLSASC54
RELSTM36 0.000 0.000 1.262 0.207

Specific indirect 3
WJPCSCX5
SLFR_G5R
SLFR_G3R
WJAPSC1S
PLSESC54
RELSTM36 0.000 0.000 1.213 0.225

Specific indirect 4
WJPCSCX5
SLFR_G5R
SLFR_G3R
WJAPSC1S
CPOMSC54
RELSTM36 0.000 0.000 -0.001 0.999

Specific indirect 5

WJPCSCX5				
SLFR_G5R				
SLFR_G3R				
WJAPSC1S				
WJMSSC54				
RELSTM36	0.000	0.000	1.101	0.271

Specific indirect 6

WJPCSCX5				
SLFR_G5R				
WJPCSCG3				
WJAPSC1S				
PLSASC54				
RELSTM36	0.000	0.000	0.341	0.733

Specific indirect 7

WJPCSCX5				
SLFR_G5R				
WJPCSCG3				
WJAPSC1S				
PLSESC54				
RELSTM36	0.000	0.000	0.336	0.737

Specific indirect 8

WJPCSCX5				
SLFR_G5R				
WJPCSCG3				
WJAPSC1S				
CPOMSC54				
RELSTM36	0.000	0.000	-0.001	0.999

Specific indirect 9

WJPCSCX5				
SLFR_G5R				
WJPCSCG3				
WJAPSC1S				
WJMSSC54				
RELSTM36	0.000	0.000	0.336	0.737

Specific indirect 10

WJPCSCX5				
WJPCSCG5				
WJPCSCG3				
WJAPSC1S				
PLSASC54				
RELSTM36	0.000	0.000	-1.313	0.189

Specific indirect 11

WJPCSCX5				
WJPCSCG5				
WJPCSCG3				
WJAPSC1S				
PLSESC54				
RELSTM36	0.000	0.000	-1.247	0.212

Specific indirect 12
WJPCSCX5
WJPCSCG5
WJPCSCG3
WJAPSC1S
CPOMSC54
RELSTM36 0.000 0.000 0.001 0.999

Specific indirect 13
WJPCSCX5
WJPCSCG5
WJPCSCG3
WJAPSC1S
WJMSSC54
RELSTM36 0.000 0.000 -1.082 0.279

Specific indirect 14
WJPCSCX5
WJPCSCG5
WJAPSCG3
WJAPSC1S
PLSASC54
RELSTM36 0.000 0.000 1.097 0.273

Specific indirect 15
WJPCSCX5
WJPCSCG5
WJAPSCG3
WJAPSC1S
PLSESC54
RELSTM36 0.000 0.000 1.049 0.294

Specific indirect 16
WJPCSCX5
WJPCSCG5
WJAPSCG3
WJAPSC1S
CPOMSC54
RELSTM36 0.000 0.000 -0.001 0.999

Specific indirect 17
WJPCSCX5
WJPCSCG5
WJAPSCG3
WJAPSC1S
WJMSSC54
RELSTM36 0.000 0.000 0.982 0.326

Specific indirect 18
WJPCSCX5
WJAPSCG5
WJPCSCG3
WJAPSC1S

```
  PLSASC54
  RELSTM36           0.000      0.000     -0.499      0.618

Specific indirect 19
  WJPCSCX5
  WJAPSCG5
  WJPCSCG3
  WJAPSC1S
  PLSESC54
  RELSTM36           0.000      0.000     -0.480      0.631

Specific indirect 20
  WJPCSCX5
  WJAPSCG5
  WJPCSCG3
  WJAPSC1S
  CPOMSC54
  RELSTM36           0.000      0.000      0.001      0.999

Specific indirect 21
  WJPCSCX5
  WJAPSCG5
  WJPCSCG3
  WJAPSC1S
  WJMSSC54
  RELSTM36           0.000      0.000     -0.471      0.638

Specific indirect 22
  WJPCSCX5
  WJAPSCG5
  WJAPSCG3
  WJAPSC1S
  PLSASC54
  RELSTM36           0.000      0.000     -0.758      0.449

Specific indirect 23
  WJPCSCX5
  WJAPSCG5
  WJAPSCG3
  WJAPSC1S
  PLSESC54
  RELSTM36           0.000      0.000     -0.758      0.448

Specific indirect 24
  WJPCSCX5
  WJAPSCG5
  WJAPSCG3
  WJAPSC1S
  CPOMSC54
  RELSTM36           0.000      0.000      0.001      0.999

Specific indirect 25
  WJPCSCX5
  WJAPSCG5
```

WJAPSCG3
WJAPSC1S
WJMSSC54
RELSTM36 0.000 0.000 -0.701 0.483

Effects from MS_24MR to WJAPSCX5

Total 0.006 0.006 0.973 0.331
Total indirect 0.006 0.006 0.973 0.331

Specific indirect 1
WJAPSCX5
SLFR_G5R
SLFR_G3R
SLFR_G1R
CPOMSC54
RVCSTM36
MS_24MR 0.000 0.000 1.891 0.059

Specific indirect 2
WJAPSCX5
SLFR_G5R
SLFR_G3R
SLFR_G1R
CPOMSC54
RELSTM36
MS_24MR 0.000 0.000 -0.001 0.999

Specific indirect 3
WJAPSCX5
SLFR_G5R
SLFR_G3R
WJAPSC1S
PLSASC54
RVCSTM36
MS_24MR 0.000 0.000 1.422 0.155

Specific indirect 4
WJAPSCX5
SLFR_G5R
SLFR_G3R
WJAPSC1S
PLSASC54
RELSTM36
MS_24MR 0.000 0.000 1.175 0.240

Specific indirect 5
WJAPSCX5
SLFR_G5R
SLFR_G3R
WJAPSC1S
PLSESC54
RVCSTM36

MS_24MR 0.000 0.000 1.196 0.232

Specific indirect 6
WJAPSCX5
SLFR_G5R
SLFR_G3R
WJAPSC1S
PLSESC54
RELSTM36
MS_24MR 0.000 0.000 1.128 0.260

Specific indirect 7
WJAPSCX5
SLFR_G5R
SLFR_G3R
WJAPSC1S
CPOMSC54
RVCSTM36
MS_24MR 0.000 0.000 1.400 0.161

Specific indirect 8
WJAPSCX5
SLFR_G5R
SLFR_G3R
WJAPSC1S
CPOMSC54
RELSTM36
MS_24MR 0.000 0.000 -0.001 0.999

Specific indirect 9
WJAPSCX5
SLFR_G5R
SLFR_G3R
WJAPSC1S
WJMSSC54
RVCSTM36
MS_24MR 0.000 0.000 1.297 0.195

Specific indirect 10
WJAPSCX5
SLFR_G5R
SLFR_G3R
WJAPSC1S
WJMSSC54
RELSTM36
MS_24MR 0.000 0.000 1.040 0.298

Specific indirect 11
WJAPSCX5
SLFR_G5R
WJPCSCG3
WJAPSC1S
PLSASC54
RVCSTM36

MS_24MR 0.000 0.000 0.351 0.726

Specific indirect 12
WJAPSCX5
SLFR_G5R
WJPCSCG3
WJAPSC1S
PLSASC54
RELSTM36
MS_24MR 0.000 0.000 0.343 0.732

Specific indirect 13
WJAPSCX5
SLFR_G5R
WJPCSCG3
WJAPSC1S
PLSESC54
RVCSTM36
MS_24MR 0.000 0.000 0.341 0.733

Specific indirect 14
WJAPSCX5
SLFR_G5R
WJPCSCG3
WJAPSC1S
PLSESC54
RELSTM36
MS_24MR 0.000 0.000 0.337 0.736

Specific indirect 15
WJAPSCX5
SLFR_G5R
WJPCSCG3
WJAPSC1S
CPOMSC54
RVCSTM36
MS_24MR 0.000 0.000 0.348 0.728

Specific indirect 16
WJAPSCX5
SLFR_G5R
WJPCSCG3
WJAPSC1S
CPOMSC54
RELSTM36
MS_24MR 0.000 0.000 -0.001 0.999

Specific indirect 17
WJAPSCX5
SLFR_G5R
WJPCSCG3
WJAPSC1S
WJMSSC54
RVCSTM36

MS_24MR 0.000 0.000 0.348 0.728

Specific indirect 18
WJAPSCX5
SLFR_G5R
WJPCSCG3
WJAPSC1S
WJMSSC54
RELSTM36
MS_24MR 0.000 0.000 0.338 0.736

Specific indirect 19
WJAPSCX5
WJPCSCG5
WJPCSCG3
WJAPSC1S
PLSASC54
RVCSTM36
MS_24MR -0.001 0.000 -1.573 0.116

Specific indirect 20
WJAPSCX5
WJPCSCG5
WJPCSCG3
WJAPSC1S
PLSASC54
RELSTM36
MS_24MR 0.000 0.000 -1.239 0.215

Specific indirect 21
WJAPSCX5
WJPCSCG5
WJPCSCG3
WJAPSC1S
PLSESC54
RVCSTM36
MS_24MR 0.000 0.000 -1.251 0.211

Specific indirect 22
WJAPSCX5
WJPCSCG5
WJPCSCG3
WJAPSC1S
PLSESC54
RELSTM36
MS_24MR 0.000 0.000 -1.187 0.235

Specific indirect 23
WJAPSCX5
WJPCSCG5
WJPCSCG3
WJAPSC1S
CPOMSC54
RVCSTM36

MS_24MR 0.000 0.000 -1.451 0.147

Specific indirect 24
WJAPSCX5
WJPCSCG5
WJPCSCG3
WJAPSC1S
CPOMSC54
RELSTM36
MS_24MR 0.000 0.000 0.001 0.999

Specific indirect 25
WJAPSCX5
WJPCSCG5
WJPCSCG3
WJAPSC1S
WJMSSC54
RVCSTM36
MS_24MR 0.000 0.000 -1.325 0.185

Specific indirect 26
WJAPSCX5
WJPCSCG5
WJPCSCG3
WJAPSC1S
WJMSSC54
RELSTM36
MS_24MR 0.000 0.000 -1.028 0.304

Specific indirect 27
WJAPSCX5
WJPCSCG5
WJAPSCG3
WJAPSC1S
PLSASC54
RVCSTM36
MS_24MR 0.002 0.001 1.328 0.184

Specific indirect 28
WJAPSCX5
WJPCSCG5
WJAPSCG3
WJAPSC1S
PLSASC54
RELSTM36
MS_24MR 0.000 0.000 1.135 0.256

Specific indirect 29
WJAPSCX5
WJPCSCG5
WJAPSCG3
WJAPSC1S
PLSESC54
RVCSTM36

MS_24MR 0.001 0.001 1.130 0.258

Specific indirect 30
WJAPSCX5
WJPCSCG5
WJAPSCG3
WJAPSC1S
PLSESC54
RELSTM36
MS_24MR 0.000 0.000 1.085 0.278

Specific indirect 31
WJAPSCX5
WJPCSCG5
WJAPSCG3
WJAPSC1S
CPOMSC54
RVCSTM36
MS_24MR 0.001 0.000 1.343 0.179

Specific indirect 32
WJAPSCX5
WJPCSCG5
WJAPSCG3
WJAPSC1S
CPOMSC54
RELSTM36
MS_24MR 0.000 0.000 -0.001 0.999

Specific indirect 33
WJAPSCX5
WJPCSCG5
WJAPSCG3
WJAPSC1S
WJMSSC54
RVCSTM36
MS_24MR 0.001 0.001 1.228 0.219

Specific indirect 34
WJAPSCX5
WJPCSCG5
WJAPSCG3
WJAPSC1S
WJMSSC54
RELSTM36
MS_24MR 0.000 0.000 0.998 0.318

Specific indirect 35
WJAPSCX5
WJAPSCG5
WJPCSCG3
WJAPSC1S
PLSASC54
RVCSTM36

MS_24MR 0.000 0.000 0.313 0.754

Specific indirect 36
WJAPSCX5
WJAPSCG5
WJPCSCG3
WJAPSC1S
PLSASC54
RELSTM36
MS_24MR 0.000 0.000 0.310 0.757

Specific indirect 37
WJAPSCX5
WJAPSCG5
WJPCSCG3
WJAPSC1S
PLSESC54
RVCSTM36
MS_24MR 0.000 0.000 0.307 0.759

Specific indirect 38
WJAPSCX5
WJAPSCG5
WJPCSCG3
WJAPSC1S
PLSESC54
RELSTM36
MS_24MR 0.000 0.000 0.306 0.760

Specific indirect 39
WJAPSCX5
WJAPSCG5
WJPCSCG3
WJAPSC1S
CPOMSC54
RVCSTM36
MS_24MR 0.000 0.000 0.318 0.751

Specific indirect 40
WJAPSCX5
WJAPSCG5
WJPCSCG3
WJAPSC1S
CPOMSC54
RELSTM36
MS_24MR 0.000 0.000 -0.001 0.999

Specific indirect 41
WJAPSCX5
WJAPSCG5
WJPCSCG3
WJAPSC1S
WJMSSC54
RVCSTM36

MS_24MR 0.000 0.000 0.312 0.755

Specific indirect 42
WJAPSCX5
WJAPSCG5
WJPCSCG3
WJAPSC1S
WJMSSC54
RELSTM36
MS_24MR 0.000 0.000 0.308 0.758

Specific indirect 43
WJAPSCX5
WJAPSCG5
WJAPSCG3
WJAPSC1S
PLSASC54
RVCSTM36
MS_24MR 0.001 0.001 0.461 0.645

Specific indirect 44
WJAPSCX5
WJAPSCG5
WJAPSCG3
WJAPSC1S
PLSASC54
RELSTM36
MS_24MR 0.000 0.000 0.452 0.652

Specific indirect 45
WJAPSCX5
WJAPSCG5
WJAPSCG3
WJAPSC1S
PLSESC54
RVCSTM36
MS_24MR 0.000 0.001 0.456 0.649

Specific indirect 46
WJAPSCX5
WJAPSCG5
WJAPSCG3
WJAPSC1S
PLSESC54
RELSTM36
MS_24MR 0.000 0.000 0.453 0.651

Specific indirect 47
WJAPSCX5
WJAPSCG5
WJAPSCG3
WJAPSC1S
CPOMSC54
RVCSTM36

MS_24MR 0.000 0.000 0.474 0.636

Specific indirect 48
WJAPSCX5
WJAPSCG5
WJAPSCG3
WJAPSC1S
CPOMSC54
RELSTM36
MS_24MR 0.000 0.000 -0.001 0.999

Specific indirect 49
WJAPSCX5
WJAPSCG5
WJAPSCG3
WJAPSC1S
WJMSSC54
RVCSTM36
MS_24MR 0.000 0.001 0.470 0.638

Specific indirect 50
WJAPSCX5
WJAPSCG5
WJAPSCG3
WJAPSC1S
WJMSSC54
RELSTM36
MS_24MR 0.000 0.000 0.455 0.649

Effects from MS_54MR to WJAPSCX5

Total 0.000 0.000 999.000 0.000
Total indirect 0.000 0.000 999.000 0.000

Effects from PLSASC54 to WJAPSCX5

Total 0.001 0.002 0.900 0.368
Total indirect 0.001 0.002 0.900 0.368

Specific indirect 1
WJAPSCX5
SLFR_G5R
SLFR_G3R
WJAPSC1S
PLSASC54 0.000 0.000 1.513 0.130

Specific indirect 2
WJAPSCX5
SLFR_G5R
WJPCSCG3
WJAPSC1S
PLSASC54 0.000 0.000 0.352 0.725

```
  Specific indirect 3
    WJAPSCX5
    WJPCSCG5
    WJPCSCG3
    WJAPSC1S
    PLSASC54            0.000      0.000     -1.713      0.087

  Specific indirect 4
    WJAPSCX5
    WJPCSCG5
    WJAPSCG3
    WJAPSC1S
    PLSASC54            0.001      0.001      1.407      0.159

  Specific indirect 5
    WJAPSCX5
    WJAPSCG5
    WJPCSCG3
    WJAPSC1S
    PLSASC54            0.000      0.000      0.314      0.753

  Specific indirect 6
    WJAPSCX5
    WJAPSCG5
    WJAPSCG3
    WJAPSC1S
    PLSASC54            0.000      0.001      0.464      0.642


Effects from PLSESC54 to WJAPSCX5

  Total                0.001      0.001      0.838      0.402
  Total indirect       0.001      0.001      0.838      0.402

  Specific indirect 1
    WJAPSCX5
    SLFR_G5R
    SLFR_G3R
    WJAPSC1S
    PLSESC54            0.000      0.000      1.240      0.215

  Specific indirect 2
    WJAPSCX5
    SLFR_G5R
    WJPCSCG3
    WJAPSC1S
    PLSESC54            0.000      0.000      0.342      0.732

  Specific indirect 3
    WJAPSCX5
    WJPCSCG5
    WJPCSCG3
    WJAPSC1S
```

PLSESC54 0.000 0.000 -1.314 0.189

Specific indirect 4
WJAPSCX5
WJPCSCG5
WJAPSCG3
WJAPSC1S
PLSESC54 0.001 0.000 1.173 0.241

Specific indirect 5
WJAPSCX5
WJAPSCG5
WJPCSCG3
WJAPSC1S
PLSESC54 0.000 0.000 0.308 0.758

Specific indirect 6
WJAPSCX5
WJAPSCG5
WJAPSCG3
WJAPSC1S
PLSESC54 0.000 0.000 0.459 0.646

Effects from CPINCC54 to WJAPSCX5

Total 0.000 0.000 999.000 0.000
Total indirect 0.000 0.000 999.000 0.000

Effects from CPOMSC54 to WJAPSCX5

Total -0.004 0.002 -1.412 0.158
Total indirect -0.004 0.002 -1.412 0.158

Specific indirect 1
WJAPSCX5
SLFR_G5R
SLFR_G3R
SLFR_G1R
CPOMSC54 -0.001 0.001 -2.197 0.028

Specific indirect 2
WJAPSCX5
SLFR_G5R
SLFR_G3R
WJAPSC1S
CPOMSC54 0.000 0.000 -1.507 0.132

Specific indirect 3
WJAPSCX5
SLFR_G5R
WJPCSCG3
WJAPSC1S

CPOMSC54 0.000 0.000 -0.349 0.727

Specific indirect 4
WJAPSCX5
WJPCSCG5
WJPCSCG3
WJAPSC1S
CPOMSC54 0.001 0.000 1.588 0.112

Specific indirect 5
WJAPSCX5
WJPCSCG5
WJAPSCG3
WJAPSC1S
CPOMSC54 -0.002 0.001 -1.437 0.151

Specific indirect 6
WJAPSCX5
WJAPSCG5
WJPCSCG3
WJAPSC1S
CPOMSC54 0.000 0.000 -0.318 0.750

Specific indirect 7
WJAPSCX5
WJAPSCG5
WJAPSCG3
WJAPSC1S
CPOMSC54 -0.001 0.001 -0.477 0.633

Effects from WJMSSC54 to WJAPSCX5

Total 0.001 0.001 0.904 0.366
Total indirect 0.001 0.001 0.904 0.366

Specific indirect 1
WJAPSCX5
SLFR_G5R
SLFR_G3R
WJAPSC1S
WJMSSC54 0.000 0.000 1.371 0.170

Specific indirect 2
WJAPSCX5
SLFR_G5R
WJPCSCG3
WJAPSC1S
WJMSSC54 0.000 0.000 0.350 0.727

Specific indirect 3
WJAPSCX5
WJPCSCG5
WJPCSCG3

WJAPSC1S
WJMSSC54 0.000 0.000 -1.404 0.160

Specific indirect 4
WJAPSCX5
WJPCSCG5
WJAPSCG3
WJAPSC1S
WJMSSC54 0.001 0.000 1.292 0.196

Specific indirect 5
WJAPSCX5
WJAPSCG5
WJPCSCG3
WJAPSC1S
WJMSSC54 0.000 0.000 0.313 0.754

Specific indirect 6
WJAPSCX5
WJAPSCG5
WJAPSCG3
WJAPSC1S
WJMSSC54 0.000 0.000 0.475 0.635

Effects from RVCSTM36 to WJAPSCX5

Total 0.002 0.002 1.006 0.315
Total indirect 0.002 0.002 1.006 0.315

Specific indirect 1
WJAPSCX5
SLFR_G5R
SLFR_G3R
SLFR_G1R
CPOMSC54
RVCSTM36 0.000 0.000 2.088 0.037

Specific indirect 2
WJAPSCX5
SLFR_G5R
SLFR_G3R
WJAPSC1S
PLSASC54
RVCSTM36 0.000 0.000 1.508 0.132

Specific indirect 3
WJAPSCX5
SLFR_G5R
SLFR_G3R
WJAPSC1S
PLSESC54
RVCSTM36 0.000 0.000 1.234 0.217

Specific indirect 4
WJAPSCX5
SLFR_G5R
SLFR_G3R
WJAPSC1S
CPOMSC54
RVCSTM36 0.000 0.000 1.480 0.139

Specific indirect 5
WJAPSCX5
SLFR_G5R
SLFR_G3R
WJAPSC1S
WJMSSC54
RVCSTM36 0.000 0.000 1.352 0.176

Specific indirect 6
WJAPSCX5
SLFR_G5R
WJPCSCG3
WJAPSC1S
PLSASC54
RVCSTM36 0.000 0.000 0.352 0.725

Specific indirect 7
WJAPSCX5
SLFR_G5R
WJPCSCG3
WJAPSC1S
PLSESC54
RVCSTM36 0.000 0.000 0.342 0.732

Specific indirect 8
WJAPSCX5
SLFR_G5R
WJPCSCG3
WJAPSC1S
CPOMSC54
RVCSTM36 0.000 0.000 0.349 0.727

Specific indirect 9
WJAPSCX5
SLFR_G5R
WJPCSCG3
WJAPSC1S
WJMSSC54
RVCSTM36 0.000 0.000 0.349 0.727

Specific indirect 10
WJAPSCX5
WJPCSCG5
WJPCSCG3
WJAPSC1S
PLSASC54

```
  RVCSTM36          0.000      0.000     -1.709      0.088

Specific indirect 11
  WJAPSCX5
  WJPCSCG5
  WJPCSCG3
  WJAPSC1S
  PLSESC54
  RVCSTM36          0.000      0.000     -1.301      0.193

Specific indirect 12
  WJAPSCX5
  WJPCSCG5
  WJPCSCG3
  WJAPSC1S
  CPOMSC54
  RVCSTM36          0.000      0.000     -1.551      0.121

Specific indirect 13
  WJAPSCX5
  WJPCSCG5
  WJPCSCG3
  WJAPSC1S
  WJMSSC54
  RVCSTM36          0.000      0.000     -1.391      0.164

Specific indirect 14
  WJAPSCX5
  WJPCSCG5
  WJAPSCG3
  WJAPSC1S
  PLSASC54
  RVCSTM36          0.001      0.001      1.402      0.161

Specific indirect 15
  WJAPSCX5
  WJPCSCG5
  WJAPSCG3
  WJAPSC1S
  PLSESC54
  RVCSTM36          0.000      0.000      1.165      0.244

Specific indirect 16
  WJAPSCX5
  WJPCSCG5
  WJAPSCG3
  WJAPSC1S
  CPOMSC54
  RVCSTM36          0.000      0.000      1.417      0.156

Specific indirect 17
  WJAPSCX5
  WJPCSCG5
  WJAPSCG3
```

WJAPSC1S
WJMSSC54
RVCSTM36 0.000 0.000 1.278 0.201

Specific indirect 18
WJAPSCX5
WJAPSCG5
WJPCSCG3
WJAPSC1S
PLSASC54
RVCSTM36 0.000 0.000 0.314 0.753

Specific indirect 19
WJAPSCX5
WJAPSCG5
WJPCSCG3
WJAPSC1S
PLSESC54
RVCSTM36 0.000 0.000 0.308 0.758

Specific indirect 20
WJAPSCX5
WJAPSCG5
WJPCSCG3
WJAPSC1S
CPOMSC54
RVCSTM36 0.000 0.000 0.319 0.750

Specific indirect 21
WJAPSCX5
WJAPSCG5
WJPCSCG3
WJAPSC1S
WJMSSC54
RVCSTM36 0.000 0.000 0.313 0.755

Specific indirect 22
WJAPSCX5
WJAPSCG5
WJAPSCG3
WJAPSC1S
PLSASC54
RVCSTM36 0.000 0.001 0.464 0.643

Specific indirect 23
WJAPSCX5
WJAPSCG5
WJAPSCG3
WJAPSC1S
PLSESC54
RVCSTM36 0.000 0.000 0.458 0.647

Specific indirect 24
WJAPSCX5

WJAPSCG5
WJAPSCG3
WJAPSC1S
CPOMSC54
RVCSTM36 0.000 0.000 0.477 0.633

Specific indirect 25
WJAPSCX5
WJAPSCG5
WJAPSCG3
WJAPSC1S
WJMSSC54
RVCSTM36 0.000 0.000 0.473 0.636

Effects from RELSTM36 to WJAPSCX5

Total 0.000 0.000 0.860 0.390
Total indirect 0.000 0.000 0.860 0.390

Specific indirect 1
WJAPSCX5
SLFR_G5R
SLFR_G3R
SLFR_G1R
CPOMSC54
RELSTM36 0.000 0.000 -0.001 0.999

Specific indirect 2
WJAPSCX5
SLFR_G5R
SLFR_G3R
WJAPSC1S
PLSASC54
RELSTM36 0.000 0.000 1.242 0.214

Specific indirect 3
WJAPSCX5
SLFR_G5R
SLFR_G3R
WJAPSC1S
PLSESC54
RELSTM36 0.000 0.000 1.188 0.235

Specific indirect 4
WJAPSCX5
SLFR_G5R
SLFR_G3R
WJAPSC1S
CPOMSC54
RELSTM36 0.000 0.000 -0.001 0.999

Specific indirect 5
WJAPSCX5

SLFR_G5R
SLFR_G3R
WJAPSC1S
WJMSSC54
RELSTM36 0.000 0.000 1.096 0.273

Specific indirect 6
WJAPSCX5
SLFR_G5R
WJPCSCG3
WJAPSC1S
PLSASC54
RELSTM36 0.000 0.000 0.346 0.730

Specific indirect 7
WJAPSCX5
SLFR_G5R
WJPCSCG3
WJAPSC1S
PLSESC54
RELSTM36 0.000 0.000 0.340 0.734

Specific indirect 8
WJAPSCX5
SLFR_G5R
WJPCSCG3
WJAPSC1S
CPOMSC54
RELSTM36 0.000 0.000 -0.001 0.999

Specific indirect 9
WJAPSCX5
SLFR_G5R
WJPCSCG3
WJAPSC1S
WJMSSC54
RELSTM36 0.000 0.000 0.341 0.733

Specific indirect 10
WJAPSCX5
WJPCSCG5
WJPCSCG3
WJAPSC1S
PLSASC54
RELSTM36 0.000 0.000 -1.328 0.184

Specific indirect 11
WJAPSCX5
WJPCSCG5
WJPCSCG3
WJAPSC1S
PLSESC54
RELSTM36 0.000 0.000 -1.266 0.206

Specific indirect 12
WJAPSCX5
WJPCSCG5
WJPCSCG3
WJAPSC1S
CPOMSC54
RELSTM36 0.000 0.000 0.001 0.999

Specific indirect 13
WJAPSCX5
WJPCSCG5
WJPCSCG3
WJAPSC1S
WJMSSC54
RELSTM36 0.000 0.000 -1.086 0.277

Specific indirect 14
WJAPSCX5
WJPCSCG5
WJAPSCG3
WJAPSC1S
PLSASC54
RELSTM36 0.000 0.000 1.191 0.234

Specific indirect 15
WJAPSCX5
WJPCSCG5
WJAPSCG3
WJAPSC1S
PLSESC54
RELSTM36 0.000 0.000 1.135 0.257

Specific indirect 16
WJAPSCX5
WJPCSCG5
WJAPSCG3
WJAPSC1S
CPOMSC54
RELSTM36 0.000 0.000 -0.001 0.999

Specific indirect 17
WJAPSCX5
WJPCSCG5
WJAPSCG3
WJAPSC1S
WJMSSC54
RELSTM36 0.000 0.000 1.044 0.297

Specific indirect 18
WJAPSCX5
WJAPSCG5
WJPCSCG3
WJAPSC1S
PLSASC54

RELSTM36 0.000 0.000 0.311 0.756

Specific indirect 19
WJAPSCX5
WJAPSCG5
WJPCSCG3
WJAPSC1S
PLSESC54
RELSTM36 0.000 0.000 0.307 0.759

Specific indirect 20
WJAPSCX5
WJAPSCG5
WJPCSCG3
WJAPSC1S
CPOMSC54
RELSTM36 0.000 0.000 -0.001 0.999

Specific indirect 21
WJAPSCX5
WJAPSCG5
WJPCSCG3
WJAPSC1S
WJMSSC54
RELSTM36 0.000 0.000 0.310 0.757

Specific indirect 22
WJAPSCX5
WJAPSCG5
WJAPSCG3
WJAPSC1S
PLSASC54
RELSTM36 0.000 0.000 0.455 0.649

Specific indirect 23
WJAPSCX5
WJAPSCG5
WJAPSCG3
WJAPSC1S
PLSESC54
RELSTM36 0.000 0.000 0.457 0.648

Specific indirect 24
WJAPSCX5
WJAPSCG5
WJAPSCG3
WJAPSC1S
CPOMSC54
RELSTM36 0.000 0.000 -0.001 0.999

Specific indirect 25
WJAPSCX5
WJAPSCG5
WJAPSCG3

WJAPSC1S
WJMSSC54
RELSTM36 0.000 0.000 0.460 0.646

Effects from MS_24MR to MS_G5R

Total 0.000 0.000 1.438 0.151
Total indirect 0.000 0.000 1.438 0.151

Specific indirect 1
MS_G5R
MS_G3R
MS_G1R
PLSASC54
RVCSTM36
MS_24MR 0.000 0.000 1.453 0.146

Specific indirect 2
MS_G5R
MS_G3R
MS_G1R
PLSASC54
RELSTM36
MS_24MR 0.000 0.000 1.183 0.237

Specific indirect 3
MS_G5R
MS_G3R
MS_G1R
MS_54MR
MS_36MR
MS_24MR 0.000 0.000 1.257 0.209

Effects from MS_54MR to MS_G5R

Total 0.004 0.003 1.521 0.128
Total indirect 0.004 0.003 1.521 0.128

Specific indirect 1
MS_G5R
MS_G3R
MS_G1R
MS_54MR 0.004 0.003 1.521 0.128

Effects from PLSASC54 to MS_G5R

Total 0.000 0.000 1.599 0.110
Total indirect 0.000 0.000 1.599 0.110

Specific indirect 1
MS_G5R

MS_G3R
MS_G1R
PLSASC54 0.000 0.000 1.599 0.110

Effects from PLSESC54 to MS_G5R

Total 0.000 0.000 0.000 1.000
Total indirect 0.000 0.000 0.000 1.000

Effects from CPINCC54 to MS_G5R

Total 0.000 0.000 999.000 0.000
Total indirect 0.000 0.000 999.000 0.000

Effects from CPOMSC54 to MS_G5R

Total 0.000 0.000 0.000 1.000
Total indirect 0.000 0.000 0.000 1.000

Effects from WJMSSC54 to MS_G5R

Total 0.000 0.000 0.000 1.000
Total indirect 0.000 0.000 0.000 1.000

Effects from RVCSTM36 to MS_G5R

Total 0.000 0.000 1.598 0.110
Total indirect 0.000 0.000 1.598 0.110

Specific indirect 1
MS_G5R
MS_G3R
MS_G1R
PLSASC54
RVCSTM36 0.000 0.000 1.598 0.110

Effects from RELSTM36 to MS_G5R

Total 0.000 0.000 1.266 0.206
Total indirect 0.000 0.000 1.266 0.206

Specific indirect 1
MS_G5R
MS_G3R
MS_G1R
PLSASC54
RELSTM36 0.000 0.000 1.266 0.206

Effects from MS_24MR to SLFR_G5R

```
  Total                0.001      0.001      2.202      0.028
  Total indirect       0.001      0.001      2.202      0.028

  Specific indirect 1
    SLFR_G5R
    SLFR_G3R
    SLFR_G1R
    CPOMSC54
    RVCSTM36
    MS_24MR            0.000      0.000      2.308      0.021

  Specific indirect 2
    SLFR_G5R
    SLFR_G3R
    SLFR_G1R
    CPOMSC54
    RELSTM36
    MS_24MR            0.000      0.000     -0.001      0.999

  Specific indirect 3
    SLFR_G5R
    SLFR_G3R
    WJAPSC1S
    PLSASC54
    RVCSTM36
    MS_24MR            0.000      0.000      1.682      0.093

  Specific indirect 4
    SLFR_G5R
    SLFR_G3R
    WJAPSC1S
    PLSASC54
    RELSTM36
    MS_24MR            0.000      0.000      1.305      0.192

  Specific indirect 5
    SLFR_G5R
    SLFR_G3R
    WJAPSC1S
    PLSESC54
    RVCSTM36
    MS_24MR            0.000      0.000      1.362      0.173

  Specific indirect 6
    SLFR_G5R
    SLFR_G3R
    WJAPSC1S
    PLSESC54
    RELSTM36
    MS_24MR            0.000      0.000      1.266      0.206
```

Specific indirect 7
SLFR_G5R
SLFR_G3R
WJAPSC1S
CPOMSC54
RVCSTM36

MS_24MR	0.000	0.000	1.654	0.098

Specific indirect 8
SLFR_G5R
SLFR_G3R
WJAPSC1S
CPOMSC54
RELSTM36

MS_24MR	0.000	0.000	-0.001	0.999

Specific indirect 9
SLFR_G5R
SLFR_G3R
WJAPSC1S
WJMSSC54
RVCSTM36

MS_24MR	0.000	0.000	1.473	0.141

Specific indirect 10
SLFR_G5R
SLFR_G3R
WJAPSC1S
WJMSSC54
RELSTM36

MS_24MR	0.000	0.000	1.117	0.264

Specific indirect 11
SLFR_G5R
WJPCSCG3
WJAPSC1S
PLSASC54
RVCSTM36

MS_24MR	0.000	0.000	0.361	0.718

Specific indirect 12
SLFR_G5R
WJPCSCG3
WJAPSC1S
PLSASC54
RELSTM36

MS_24MR	0.000	0.000	0.352	0.725

Specific indirect 13
SLFR_G5R
WJPCSCG3
WJAPSC1S
PLSESC54
RVCSTM36

MS_24MR 0.000 0.000 0.350 0.726

Specific indirect 14
SLFR_G5R
WJPCSCG3
WJAPSC1S
PLSESC54
RELSTM36
MS_24MR 0.000 0.000 0.346 0.729

Specific indirect 15
SLFR_G5R
WJPCSCG3
WJAPSC1S
CPOMSC54
RVCSTM36
MS_24MR 0.000 0.000 0.358 0.721

Specific indirect 16
SLFR_G5R
WJPCSCG3
WJAPSC1S
CPOMSC54
RELSTM36
MS_24MR 0.000 0.000 -0.001 0.999

Specific indirect 17
SLFR_G5R
WJPCSCG3
WJAPSC1S
WJMSSC54
RVCSTM36
MS_24MR 0.000 0.000 0.357 0.721

Specific indirect 18
SLFR_G5R
WJPCSCG3
WJAPSC1S
WJMSSC54
RELSTM36
MS_24MR 0.000 0.000 0.346 0.729

Effects from MS_54MR to SLFR_G5R

Total 0.000 0.000 999.000 0.000
Total indirect 0.000 0.000 999.000 0.000

Effects from PLSASC54 to SLFR_G5R

Total 0.000 0.000 1.786 0.074
Total indirect 0.000 0.000 1.786 0.074

Specific indirect 1
SLFR_G5R
SLFR_G3R
WJAPSC1S
PLSASC54 0.000 0.000 1.830 0.067

Specific indirect 2
SLFR_G5R
WJPCSCG3
WJAPSC1S
PLSASC54 0.000 0.000 0.362 0.718

Effects from PLSESC54 to SLFR_G5R

Total 0.000 0.000 1.378 0.168
Total indirect 0.000 0.000 1.378 0.168

Specific indirect 1
SLFR_G5R
SLFR_G3R
WJAPSC1S
PLSESC54 0.000 0.000 1.426 0.154

Specific indirect 2
SLFR_G5R
WJPCSCG3
WJAPSC1S
PLSESC54 0.000 0.000 0.351 0.725

Effects from CPINCC54 to SLFR_G5R

Total 0.000 0.000 999.000 0.000
Total indirect 0.000 0.000 999.000 0.000

Effects from CPOMSC54 to SLFR_G5R

Total -0.001 0.000 -3.259 0.001
Total indirect -0.001 0.000 -3.259 0.001

Specific indirect 1
SLFR_G5R
SLFR_G3R
SLFR_G1R
CPOMSC54 -0.001 0.000 -2.910 0.004

Specific indirect 2
SLFR_G5R
SLFR_G3R
WJAPSC1S
CPOMSC54 0.000 0.000 -1.830 0.067

Specific indirect 3
SLFR_G5R
WJPCSCG3
WJAPSC1S
CPOMSC54 0.000 0.000 -0.359 0.720

Effects from WJMSSC54 to SLFR_G5R

Total 0.000 0.000 1.545 0.122
Total indirect 0.000 0.000 1.545 0.122

Specific indirect 1
SLFR_G5R
SLFR_G3R
WJAPSC1S
WJMSSC54 0.000 0.000 1.574 0.115

Specific indirect 2
SLFR_G5R
WJPCSCG3
WJAPSC1S
WJMSSC54 0.000 0.000 0.359 0.720

Effects from RVCSTM36 to SLFR_G5R

Total 0.000 0.000 2.568 0.010
Total indirect 0.000 0.000 2.568 0.010

Specific indirect 1
SLFR_G5R
SLFR_G3R
SLFR_G1R
CPOMSC54
RVCSTM36 0.000 0.000 2.668 0.008

Specific indirect 2
SLFR_G5R
SLFR_G3R
WJAPSC1S
PLSASC54
RVCSTM36 0.000 0.000 1.821 0.069

Specific indirect 3
SLFR_G5R
SLFR_G3R
WJAPSC1S
PLSESC54
RVCSTM36 0.000 0.000 1.415 0.157

Specific indirect 4
SLFR_G5R
SLFR_G3R

WJAPSC1S
CPOMSC54
RVCSTM36 0.000 0.000 1.781 0.075

Specific indirect 5
SLFR_G5R
SLFR_G3R
WJAPSC1S
WJMSSC54
RVCSTM36 0.000 0.000 1.549 0.121

Specific indirect 6
SLFR_G5R
WJPCSCG3
WJAPSC1S
PLSASC54
RVCSTM36 0.000 0.000 0.362 0.717

Specific indirect 7
SLFR_G5R
WJPCSCG3
WJAPSC1S
PLSESC54
RVCSTM36 0.000 0.000 0.351 0.725

Specific indirect 8
SLFR_G5R
WJPCSCG3
WJAPSC1S
CPOMSC54
RVCSTM36 0.000 0.000 0.359 0.720

Specific indirect 9
SLFR_G5R
WJPCSCG3
WJAPSC1S
WJMSSC54
RVCSTM36 0.000 0.000 0.358 0.720

Effects from RELSTM36 to SLFR_G5R

Total 0.000 0.000 1.112 0.266
Total indirect 0.000 0.000 1.112 0.266

Specific indirect 1
SLFR_G5R
SLFR_G3R
SLFR_G1R
CPOMSC54
RELSTM36 0.000 0.000 -0.001 0.999

Specific indirect 2
SLFR_G5R

```
  SLFR_G3R
  WJAPSC1S
  PLSASC54
  RELSTM36             0.000      0.000      1.397      0.162

Specific indirect 3
  SLFR_G5R
  SLFR_G3R
  WJAPSC1S
  PLSESC54
  RELSTM36             0.000      0.000      1.350      0.177

Specific indirect 4
  SLFR_G5R
  SLFR_G3R
  WJAPSC1S
  CPOMSC54
  RELSTM36             0.000      0.000     -0.001      0.999

Specific indirect 5
  SLFR_G5R
  SLFR_G3R
  WJAPSC1S
  WJMSSC54
  RELSTM36             0.000      0.000      1.185      0.236

Specific indirect 6
  SLFR_G5R
  WJPCSCG3
  WJAPSC1S
  PLSASC54
  RELSTM36             0.000      0.000      0.355      0.723

Specific indirect 7
  SLFR_G5R
  WJPCSCG3
  WJAPSC1S
  PLSESC54
  RELSTM36             0.000      0.000      0.349      0.727

Specific indirect 8
  SLFR_G5R
  WJPCSCG3
  WJAPSC1S
  CPOMSC54
  RELSTM36             0.000      0.000     -0.001      0.999

Specific indirect 9
  SLFR_G5R
  WJPCSCG3
  WJAPSC1S
  WJMSSC54
  RELSTM36             0.000      0.000      0.349      0.727
```

Effects from MS_24MR to WJPCSCG5

```
  Total              -0.006      0.005     -1.283      0.199
  Total indirect     -0.006      0.005     -1.283      0.199

  Specific indirect 1
    WJPCSCG5
    WJPCSCG3
    WJAPSC1S
    PLSASC54
    RVCSTM36
    MS_24MR           0.001      0.001      1.503      0.133

  Specific indirect 2
    WJPCSCG5
    WJPCSCG3
    WJAPSC1S
    PLSASC54
    RELSTM36
    MS_24MR           0.000      0.000      1.198      0.231

  Specific indirect 3
    WJPCSCG5
    WJPCSCG3
    WJAPSC1S
    PLSESC54
    RVCSTM36
    MS_24MR           0.001      0.000      1.240      0.215

  Specific indirect 4
    WJPCSCG5
    WJPCSCG3
    WJAPSC1S
    PLSESC54
    RELSTM36
    MS_24MR           0.000      0.000      1.183      0.237

  Specific indirect 5
    WJPCSCG5
    WJPCSCG3
    WJAPSC1S
    CPOMSC54
    RVCSTM36
    MS_24MR           0.000      0.000      1.347      0.178

  Specific indirect 6
    WJPCSCG5
    WJPCSCG3
    WJAPSC1S
    CPOMSC54
    RELSTM36
    MS_24MR           0.000      0.000     -0.001      0.999
```

Specific indirect 7
WJPCSCG5
WJPCSCG3
WJAPSC1S
WJMSSC54
RVCSTM36
MS_24MR 0.001 0.000 1.295 0.195

Specific indirect 8
WJPCSCG5
WJPCSCG3
WJAPSC1S
WJMSSC54
RELSTM36
MS_24MR 0.000 0.000 1.012 0.311

Specific indirect 9
WJPCSCG5
WJAPSCG3
WJAPSC1S
PLSASC54
RVCSTM36
MS_24MR -0.004 0.002 -1.766 0.077

Specific indirect 10
WJPCSCG5
WJAPSCG3
WJAPSC1S
PLSASC54
RELSTM36
MS_24MR 0.000 0.000 -1.365 0.172

Specific indirect 11
WJPCSCG5
WJAPSCG3
WJAPSC1S
PLSESC54
RVCSTM36
MS_24MR -0.002 0.001 -1.403 0.161

Specific indirect 12
WJPCSCG5
WJAPSCG3
WJAPSC1S
PLSESC54
RELSTM36
MS_24MR 0.000 0.000 -1.326 0.185

Specific indirect 13
WJPCSCG5
WJAPSCG3
WJAPSC1S
CPOMSC54
RVCSTM36

MS_24MR -0.001 0.001 -1.703 0.089

Specific indirect 14
WJPCSCG5
WJAPSCG3
WJAPSC1S
CPOMSC54
RELSTM36
MS_24MR 0.000 0.000 0.001 0.999

Specific indirect 15
WJPCSCG5
WJAPSCG3
WJAPSC1S
WJMSSC54
RVCSTM36
MS_24MR -0.001 0.001 -1.575 0.115

Specific indirect 16
WJPCSCG5
WJAPSCG3
WJAPSC1S
WJMSSC54
RELSTM36
MS_24MR 0.000 0.000 -1.157 0.247

Effects from MS_54MR to WJPCSCG5

Total	0.000	0.000	999.000	0.000
Total indirect	0.000	0.000	999.000	0.000

Effects from PLSASC54 to WJPCSCG5

Total	-0.002	0.001	-1.272	0.203
Total indirect	-0.002	0.001	-1.272	0.203

Specific indirect 1
WJPCSCG5
WJPCSCG3
WJAPSC1S
PLSASC54 0.001 0.001 1.622 0.105

Specific indirect 2
WJPCSCG5
WJAPSCG3
WJAPSC1S
PLSASC54 -0.002 0.001 -1.966 0.049

Effects from PLSESC54 to WJPCSCG5

Total	-0.001	0.001	-1.116	0.264

```
  Total indirect      -0.001      0.001     -1.116      0.264

  Specific indirect 1
    WJPCSCG5
    WJPCSCG3
    WJAPSC1S
    PLSESC54           0.000      0.000      1.302      0.193

  Specific indirect 2
    WJPCSCG5
    WJAPSCG3
    WJAPSC1S
    PLSESC54          -0.001      0.001     -1.488      0.137


Effects from CPINCC54 to WJPCSCG5

  Total                0.000      0.000    999.000      0.000
  Total indirect       0.000      0.000    999.000      0.000


Effects from CPOMSC54 to WJPCSCG5

  Total                0.003      0.002      1.305      0.192
  Total indirect       0.003      0.002      1.305      0.192

  Specific indirect 1
    WJPCSCG5
    WJPCSCG3
    WJAPSC1S
    CPOMSC54          -0.001      0.001     -1.465      0.143

  Specific indirect 2
    WJPCSCG5
    WJAPSCG3
    WJAPSC1S
    CPOMSC54           0.004      0.002      1.933      0.053


Effects from WJMSSC54 to WJPCSCG5

  Total               -0.001      0.001     -1.228      0.220
  Total indirect      -0.001      0.001     -1.228      0.220

  Specific indirect 1
    WJPCSCG5
    WJPCSCG3
    WJAPSC1S
    WJMSSC54           0.000      0.000      1.366      0.172

  Specific indirect 2
    WJPCSCG5
    WJAPSCG3
    WJAPSC1S
```

WJMSSC54 -0.001 0.001 -1.711 0.087

Effects from RVCSTM36 to WJPCSCG5

Total -0.003 0.002 -1.339 0.180
Total indirect -0.003 0.002 -1.339 0.180

Specific indirect 1
WJPCSCG5
WJPCSCG3
WJAPSC1S
PLSASC54
RVCSTM36 0.001 0.000 1.621 0.105

Specific indirect 2
WJPCSCG5
WJPCSCG3
WJAPSC1S
PLSESC54
RVCSTM36 0.000 0.000 1.291 0.197

Specific indirect 3
WJPCSCG5
WJPCSCG3
WJAPSC1S
CPOMSC54
RVCSTM36 0.000 0.000 1.427 0.154

Specific indirect 4
WJPCSCG5
WJPCSCG3
WJAPSC1S
WJMSSC54
RVCSTM36 0.000 0.000 1.359 0.174

Specific indirect 5
WJPCSCG5
WJAPSCG3
WJAPSC1S
PLSASC54
RVCSTM36 -0.002 0.001 -1.955 0.051

Specific indirect 6
WJPCSCG5
WJAPSCG3
WJAPSC1S
PLSESC54
RVCSTM36 -0.001 0.001 -1.472 0.141

Specific indirect 7
WJPCSCG5
WJAPSCG3
WJAPSC1S

CPOMSC54
RVCSTM36 -0.001 0.000 -1.864 0.062

Specific indirect 8
WJPCSCG5
WJAPSCG3
WJAPSC1S
WJMSSC54
RVCSTM36 -0.001 0.000 -1.685 0.092

Effects from RELSTM36 to WJPCSCG5

Total 0.000 0.000 -1.202 0.230
Total indirect 0.000 0.000 -1.202 0.230

Specific indirect 1
WJPCSCG5
WJPCSCG3
WJAPSC1S
PLSASC54
RELSTM36 0.000 0.000 1.278 0.201

Specific indirect 2
WJPCSCG5
WJPCSCG3
WJAPSC1S
PLSESC54
RELSTM36 0.000 0.000 1.261 0.207

Specific indirect 3
WJPCSCG5
WJPCSCG3
WJAPSC1S
CPOMSC54
RELSTM36 0.000 0.000 -0.001 0.999

Specific indirect 4
WJPCSCG5
WJPCSCG3
WJAPSC1S
WJMSSC54
RELSTM36 0.000 0.000 1.068 0.286

Specific indirect 5
WJPCSCG5
WJAPSCG3
WJAPSC1S
PLSASC54
RELSTM36 0.000 0.000 -1.466 0.143

Specific indirect 6
WJPCSCG5
WJAPSCG3

WJAPSC1S
PLSESC54
RELSTM36 0.000 0.000 -1.420 0.156

Specific indirect 7
WJPCSCG5
WJAPSCG3
WJAPSC1S
CPOMSC54
RELSTM36 0.000 0.000 0.001 0.999

Specific indirect 8
WJPCSCG5
WJAPSCG3
WJAPSC1S
WJMSSC54
RELSTM36 0.000 0.000 -1.230 0.219

Effects from MS_24MR to WJAPSCG5

Total 0.024 0.013 1.861 0.063
Total indirect 0.024 0.013 1.861 0.063

Specific indirect 1
WJAPSCG5
WJPCSCG3
WJAPSC1S
PLSASC54
RVCSTM36
MS_24MR 0.000 0.001 0.421 0.674

Specific indirect 2
WJAPSCG5
WJPCSCG3
WJAPSC1S
PLSASC54
RELSTM36
MS_24MR 0.000 0.000 0.411 0.681

Specific indirect 3
WJAPSCG5
WJPCSCG3
WJAPSC1S
PLSESC54
RVCSTM36
MS_24MR 0.000 0.000 0.402 0.688

Specific indirect 4
WJAPSCG5
WJPCSCG3
WJAPSC1S
PLSESC54
RELSTM36

MS_24MR	0.000	0.000	0.399	0.690

Specific indirect 5

WJAPSCG5				
WJPCSCG3				
WJAPSC1S				
CPOMSC54				
RVCSTM36				
MS_24MR	0.000	0.000	0.421	0.674

Specific indirect 6

WJAPSCG5				
WJPCSCG3				
WJAPSC1S				
CPOMSC54				
RELSTM36				
MS_24MR	0.000	0.000	-0.001	0.999

Specific indirect 7

WJAPSCG5				
WJPCSCG3				
WJAPSC1S				
WJMSSC54				
RVCSTM36				
MS_24MR	0.000	0.000	0.407	0.684

Specific indirect 8

WJAPSCG5				
WJPCSCG3				
WJAPSC1S				
WJMSSC54				
RELSTM36				
MS_24MR	0.000	0.000	0.398	0.691

Specific indirect 9

WJAPSCG5				
WJAPSCG3				
WJAPSC1S				
PLSASC54				
RVCSTM36				
MS_24MR	0.010	0.006	1.759	0.079

Specific indirect 10

WJAPSCG5				
WJAPSCG3				
WJAPSC1S				
PLSASC54				
RELSTM36				
MS_24MR	0.001	0.001	1.361	0.173

Specific indirect 11

WJAPSCG5
WJAPSCG3
WJAPSC1S

PLSESC54
RVCSTM36
MS_24MR 0.004 0.003 1.361 0.173

Specific indirect 12
WJAPSCG5
WJAPSCG3
WJAPSC1S
PLSESC54
RELSTM36
MS_24MR 0.001 0.001 1.281 0.200

Specific indirect 13
WJAPSCG5
WJAPSCG3
WJAPSC1S
CPOMSC54
RVCSTM36
MS_24MR 0.003 0.002 1.767 0.077

Specific indirect 14
WJAPSCG5
WJAPSCG3
WJAPSC1S
CPOMSC54
RELSTM36
MS_24MR 0.000 0.000 -0.001 0.999

Specific indirect 15
WJAPSCG5
WJAPSCG3
WJAPSC1S
WJMSSC54
RVCSTM36
MS_24MR 0.004 0.002 1.566 0.117

Specific indirect 16
WJAPSCG5
WJAPSCG3
WJAPSC1S
WJMSSC54
RELSTM36
MS_24MR 0.000 0.000 1.153 0.249

Effects from MS_54MR to WJAPSCG5

Total 0.000 0.000 999.000 0.000
Total indirect 0.000 0.000 999.000 0.000

Effects from PLSASC54 to WJAPSCG5

Total 0.006 0.003 1.887 0.059

Total indirect 0.006 0.003 1.887 0.059

Specific indirect 1
WJAPSCG5
WJPCSCG3
WJAPSC1S
PLSASC54 0.000 0.001 0.422 0.673

Specific indirect 2
WJAPSCG5
WJAPSCG3
WJAPSC1S
PLSASC54 0.006 0.003 1.934 0.053

Effects from PLSESC54 to WJAPSCG5

Total 0.003 0.002 1.393 0.164
Total indirect 0.003 0.002 1.393 0.164

Specific indirect 1
WJAPSCG5
WJPCSCG3
WJAPSC1S
PLSESC54 0.000 0.000 0.404 0.686

Specific indirect 2
WJAPSCG5
WJAPSCG3
WJAPSC1S
PLSESC54 0.003 0.002 1.431 0.152

Effects from CPINCC54 to WJAPSCG5

Total 0.000 0.000 999.000 0.000
Total indirect 0.000 0.000 999.000 0.000

Effects from CPOMSC54 to WJAPSCG5

Total -0.010 0.005 -1.932 0.053
Total indirect -0.010 0.005 -1.932 0.053

Specific indirect 1
WJAPSCG5
WJPCSCG3
WJAPSC1S
CPOMSC54 0.000 0.001 -0.423 0.672

Specific indirect 2
WJAPSCG5
WJAPSCG3
WJAPSC1S

CPOMSC54 -0.010 0.005 -1.981 0.048

Effects from WJMSSC54 to WJAPSCG5

Total 0.003 0.002 1.632 0.103
Total indirect 0.003 0.002 1.632 0.103

Specific indirect 1
WJAPSCG5
WJPCSCG3
WJAPSC1S
WJMSSC54 0.000 0.000 0.410 0.682

Specific indirect 2
WJAPSCG5
WJAPSCG3
WJAPSC1S
WJMSSC54 0.003 0.002 1.690 0.091

Effects from RVCSTM36 to WJAPSCG5

Total 0.010 0.005 2.033 0.042
Total indirect 0.010 0.005 2.033 0.042

Specific indirect 1
WJAPSCG5
WJPCSCG3
WJAPSC1S
PLSASC54
RVCSTM36 0.000 0.000 0.422 0.673

Specific indirect 2
WJAPSCG5
WJPCSCG3
WJAPSC1S
PLSESC54
RVCSTM36 0.000 0.000 0.403 0.687

Specific indirect 3
WJAPSCG5
WJPCSCG3
WJAPSC1S
CPOMSC54
RVCSTM36 0.000 0.000 0.423 0.672

Specific indirect 4
WJAPSCG5
WJPCSCG3
WJAPSC1S
WJMSSC54
RVCSTM36 0.000 0.000 0.409 0.683

Specific indirect 5
WJAPSCG5
WJAPSCG3
WJAPSC1S
PLSASC54
RVCSTM36 0.005 0.002 1.919 0.055

Specific indirect 6
WJAPSCG5
WJAPSCG3
WJAPSC1S
PLSESC54
RVCSTM36 0.002 0.001 1.413 0.158

Specific indirect 7
WJAPSCG5
WJAPSCG3
WJAPSC1S
CPOMSC54
RVCSTM36 0.001 0.001 1.922 0.055

Specific indirect 8
WJAPSCG5
WJAPSCG3
WJAPSC1S
WJMSSC54
RVCSTM36 0.002 0.001 1.656 0.098

Effects from RELSTM36 to WJAPSCG5

Total 0.001 0.001 1.594 0.111
Total indirect 0.001 0.001 1.594 0.111

Specific indirect 1
WJAPSCG5
WJPCSCG3
WJAPSC1S
PLSASC54
RELSTM36 0.000 0.000 0.415 0.678

Specific indirect 2
WJAPSCG5
WJPCSCG3
WJAPSC1S
PLSESC54
RELSTM36 0.000 0.000 0.402 0.688

Specific indirect 3
WJAPSCG5
WJPCSCG3
WJAPSC1S
CPOMSC54
RELSTM36 0.000 0.000 -0.001 0.999

Specific indirect 4
WJAPSCG5
WJPCSCG3
WJAPSC1S
WJMSSC54
RELSTM36 0.000 0.000 0.401 0.688

Specific indirect 5
WJAPSCG5
WJAPSCG3
WJAPSC1S
PLSASC54
RELSTM36 0.000 0.000 1.460 0.144

Specific indirect 6
WJAPSCG5
WJAPSCG3
WJAPSC1S
PLSESC54
RELSTM36 0.001 0.000 1.363 0.173

Specific indirect 7
WJAPSCG5
WJAPSCG3
WJAPSC1S
CPOMSC54
RELSTM36 0.000 0.000 -0.001 0.999

Specific indirect 8
WJAPSCG5
WJAPSCG3
WJAPSC1S
WJMSSC54
RELSTM36 0.000 0.000 1.225 0.221

Effects from MS_24MR to MS_G3R

Total 0.004 0.002 2.128 0.033
Total indirect 0.004 0.002 2.128 0.033

Specific indirect 1
MS_G3R
MS_G1R
PLSASC54
RVCSTM36
MS_24MR 0.002 0.001 2.122 0.034

Specific indirect 2
MS_G3R
MS_G1R
PLSASC54
RELSTM36

MS_24MR 0.000 0.000 1.497 0.134

Specific indirect 3
MS_G3R
MS_G1R
MS_54MR
MS_36MR
MS_24MR 0.002 0.001 1.662 0.096

Effects from MS_54MR to MS_G3R

Total	0.035	0.015	2.338	0.019
Total indirect	0.035	0.015	2.338	0.019

Specific indirect 1
MS_G3R
MS_G1R
MS_54MR 0.035 0.015 2.338 0.019

Effects from PLSASC54 to MS_G3R

Total	0.001	0.000	2.569	0.010
Total indirect	0.001	0.000	2.569	0.010

Specific indirect 1
MS_G3R
MS_G1R
PLSASC54 0.001 0.000 2.569 0.010

Effects from PLSESC54 to MS_G3R

Total	0.000	0.000	0.000	1.000
Total indirect	0.000	0.000	0.000	1.000

Effects from CPINCC54 to MS_G3R

Total	0.000	0.000	999.000	0.000
Total indirect	0.000	0.000	999.000	0.000

Effects from CPOMSC54 to MS_G3R

Total	0.000	0.000	0.000	1.000
Total indirect	0.000	0.000	0.000	1.000

Effects from WJMSSC54 to MS_G3R

Total	0.000	0.000	-2.008	0.045
Total indirect	0.000	0.000	-2.008	0.045

Effects from RVCSTM36 to MS_G3R

Total	0.001	0.000	2.551	0.011
Total indirect	0.001	0.000	2.551	0.011

Specific indirect 1
MS_G3R
MS_G1R
PLSASC54
RVCSTM36 0.001 0.000 2.551 0.011

Effects from RELSTM36 to MS_G3R

Total	0.000	0.000	1.666	0.096
Total indirect	0.000	0.000	1.666	0.096

Specific indirect 1
MS_G3R
MS_G1R
PLSASC54
RELSTM36 0.000 0.000 1.666 0.096

Effects from MS_24MR to SLFR_G3R

Total	0.004	0.002	2.453	0.014
Total indirect	0.004	0.002	2.453	0.014

Specific indirect 1
SLFR_G3R
SLFR_G1R
CPOMSC54
RVCSTM36
MS_24MR 0.001 0.001 2.542 0.011

Specific indirect 2
SLFR_G3R
SLFR_G1R
CPOMSC54
RELSTM36
MS_24MR 0.000 0.000 -0.001 0.999

Specific indirect 3
SLFR_G3R
WJAPSC1S
PLSASC54
RVCSTM36
MS_24MR 0.001 0.001 1.741 0.082

Specific indirect 4
SLFR_G3R

WJAPSC1S
PLSASC54
RELSTM36
MS_24MR 0.000 0.000 1.334 0.182

Specific indirect 5
SLFR_G3R
WJAPSC1S
PLSESC54
RVCSTM36
MS_24MR 0.001 0.000 1.394 0.163

Specific indirect 6
SLFR_G3R
WJAPSC1S
PLSESC54
RELSTM36
MS_24MR 0.000 0.000 1.287 0.198

Specific indirect 7
SLFR_G3R
WJAPSC1S
CPOMSC54
RVCSTM36
MS_24MR 0.000 0.000 1.712 0.087

Specific indirect 8
SLFR_G3R
WJAPSC1S
CPOMSC54
RELSTM36
MS_24MR 0.000 0.000 -0.001 0.999

Specific indirect 9
SLFR_G3R
WJAPSC1S
WJMSSC54
RVCSTM36
MS_24MR 0.000 0.000 1.520 0.128

Specific indirect 10
SLFR_G3R
WJAPSC1S
WJMSSC54
RELSTM36
MS_24MR 0.000 0.000 1.139 0.255

Effects from MS_54MR to SLFR_G3R

Total 0.000 0.000 999.000 0.000
Total indirect 0.000 0.000 999.000 0.000

Effects from PLSASC54 to SLFR_G3R

```
  Total                0.001      0.000      1.906      0.057
  Total indirect       0.001      0.000      1.906      0.057

  Specific indirect 1
    SLFR_G3R
    WJAPSC1S
    PLSASC54           0.001      0.000      1.906      0.057
```

Effects from PLSESC54 to SLFR_G3R

```
  Total                0.000      0.000      1.462      0.144
  Total indirect       0.000      0.000      1.462      0.144

  Specific indirect 1
    SLFR_G3R
    WJAPSC1S
    PLSESC54           0.000      0.000      1.462      0.144
```

Effects from CPINCC54 to SLFR_G3R

```
  Total                0.000      0.000    999.000      0.000
  Total indirect       0.000      0.000    999.000      0.000
```

Effects from CPOMSC54 to SLFR_G3R

```
  Total               -0.005      0.001     -3.942      0.000
  Total indirect      -0.005      0.001     -3.942      0.000

  Specific indirect 1
    SLFR_G3R
    SLFR_G1R
    CPOMSC54          -0.004      0.001     -3.424      0.001

  Specific indirect 2
    SLFR_G3R
    WJAPSC1S
    CPOMSC54          -0.001      0.001     -1.910      0.056
```

Effects from WJMSSC54 to SLFR_G3R

```
  Total                0.000      0.000      1.631      0.103
  Total indirect       0.000      0.000      1.631      0.103

  Specific indirect 1
    SLFR_G3R
    WJAPSC1S
    WJMSSC54           0.000      0.000      1.631      0.103
```

Effects from RVCSTM36 to SLFR_G3R

Total	0.002	0.001	2.966	0.003
Total indirect	0.002	0.001	2.966	0.003

Specific indirect 1
SLFR_G3R
SLFR_G1R
CPOMSC54
RVCSTM36 0.001 0.000 3.049 0.002

Specific indirect 2
SLFR_G3R
WJAPSC1S
PLSASC54
RVCSTM36 0.001 0.000 1.897 0.058

Specific indirect 3
SLFR_G3R
WJAPSC1S
PLSESC54
RVCSTM36 0.000 0.000 1.451 0.147

Specific indirect 4
SLFR_G3R
WJAPSC1S
CPOMSC54
RVCSTM36 0.000 0.000 1.854 0.064

Specific indirect 5
SLFR_G3R
WJAPSC1S
WJMSSC54
RVCSTM36 0.000 0.000 1.604 0.109

Effects from RELSTM36 to SLFR_G3R

Total	0.000	0.000	1.124	0.261
Total indirect	0.000	0.000	1.124	0.261

Specific indirect 1
SLFR_G3R
SLFR_G1R
CPOMSC54
RELSTM36 0.000 0.000 -0.001 0.999

Specific indirect 2
SLFR_G3R
WJAPSC1S
PLSASC54
RELSTM36 0.000 0.000 1.436 0.151

Specific indirect 3
SLFR_G3R
WJAPSC1S
PLSESC54
RELSTM36 0.000 0.000 1.380 0.168

Specific indirect 4
SLFR_G3R
WJAPSC1S
CPOMSC54
RELSTM36 0.000 0.000 -0.001 0.999

Specific indirect 5
SLFR_G3R
WJAPSC1S
WJMSSC54
RELSTM36 0.000 0.000 1.213 0.225

Effects from MS_24MR to WJPCSCG3

Total 0.038 0.020 1.887 0.059
Total indirect 0.038 0.020 1.887 0.059

Specific indirect 1
WJPCSCG3
WJAPSC1S
PLSASC54
RVCSTM36
MS_24MR 0.016 0.009 1.806 0.071

Specific indirect 2
WJPCSCG3
WJAPSC1S
PLSASC54
RELSTM36
MS_24MR 0.001 0.001 1.356 0.175

Specific indirect 3
WJPCSCG3
WJAPSC1S
PLSESC54
RVCSTM36
MS_24MR 0.007 0.005 1.347 0.178

Specific indirect 4
WJPCSCG3
WJAPSC1S
PLSESC54
RELSTM36
MS_24MR 0.002 0.001 1.262 0.207

Specific indirect 5
WJPCSCG3

WJAPSC1S
CPOMSC54
RVCSTM36
MS_24MR 0.005 0.003 1.683 0.092

Specific indirect 6
WJPCSCG3
WJAPSC1S
CPOMSC54
RELSTM36
MS_24MR 0.000 0.001 -0.001 0.999

Specific indirect 7
WJPCSCG3
WJAPSC1S
WJMSSC54
RVCSTM36
MS_24MR 0.006 0.004 1.461 0.144

Specific indirect 8
WJPCSCG3
WJAPSC1S
WJMSSC54
RELSTM36
MS_24MR 0.001 0.001 1.106 0.269

Effects from MS_54MR to WJPCSCG3

Total 0.000 0.000 999.000 0.000
Total indirect 0.000 0.000 999.000 0.000

Effects from PLSASC54 to WJPCSCG3

Total 0.010 0.005 2.004 0.045
Total indirect 0.010 0.005 2.004 0.045

Specific indirect 1
WJPCSCG3
WJAPSC1S
PLSASC54 0.010 0.005 2.004 0.045

Effects from PLSESC54 to WJPCSCG3

Total 0.005 0.004 1.417 0.156
Total indirect 0.005 0.004 1.417 0.156

Specific indirect 1
WJPCSCG3
WJAPSC1S
PLSESC54 0.005 0.004 1.417 0.156

Effects from CPINCC54 to WJPCSCG3

Total 0.000 0.000 999.000 0.000
Total indirect 0.000 0.000 999.000 0.000

Effects from CPOMSC54 to WJPCSCG3

Total -0.015 0.008 -1.895 0.058
Total indirect -0.015 0.008 -1.895 0.058

Specific indirect 1
WJPCSCG3
WJAPSC1S
CPOMSC54 -0.015 0.008 -1.895 0.058

Effects from WJMSSC54 to WJPCSCG3

Total 0.005 0.003 1.563 0.118
Total indirect 0.005 0.003 1.563 0.118

Specific indirect 1
WJPCSCG3
WJAPSC1S
WJMSSC54 0.005 0.003 1.563 0.118

Effects from RVCSTM36 to WJPCSCG3

Total 0.015 0.007 2.091 0.037
Total indirect 0.015 0.007 2.091 0.037

Specific indirect 1
WJPCSCG3
WJAPSC1S
PLSASC54
RVCSTM36 0.007 0.004 1.998 0.046

Specific indirect 2
WJPCSCG3
WJAPSC1S
PLSESC54
RVCSTM36 0.003 0.002 1.404 0.160

Specific indirect 3
WJPCSCG3
WJAPSC1S
CPOMSC54
RVCSTM36 0.002 0.001 1.829 0.067

Specific indirect 4
WJPCSCG3

WJAPSC1S
WJMSSC54
RVCSTM36 0.003 0.002 1.542 0.123

Effects from RELSTM36 to WJPCSCG3

Total 0.002 0.001 1.611 0.107
Total indirect 0.002 0.001 1.611 0.107

Specific indirect 1
WJPCSCG3
WJAPSC1S
PLSASC54
RELSTM36 0.001 0.001 1.469 0.142

Specific indirect 2
WJPCSCG3
WJAPSC1S
PLSESC54
RELSTM36 0.001 0.001 1.353 0.176

Specific indirect 3
WJPCSCG3
WJAPSC1S
CPOMSC54
RELSTM36 0.000 0.000 -0.001 0.999

Specific indirect 4
WJPCSCG3
WJAPSC1S
WJMSSC54
RELSTM36 0.000 0.000 1.176 0.240

Effects from MS_24MR to WJAPSCG3

Total 0.107 0.035 3.034 0.002
Total indirect 0.107 0.035 3.034 0.002

Specific indirect 1
WJAPSCG3
WJAPSC1S
PLSASC54
RVCSTM36
MS_24MR 0.046 0.018 2.544 0.011

Specific indirect 2
WJAPSCG3
WJAPSC1S
PLSASC54
RELSTM36
MS_24MR 0.004 0.002 1.636 0.102

Specific indirect 3
WJAPSCG3
WJAPSC1S
PLSESC54
RVCSTM36
MS_24MR 0.020 0.012 1.662 0.097

Specific indirect 4
WJAPSCG3
WJAPSC1S
PLSESC54
RELSTM36
MS_24MR 0.004 0.003 1.517 0.129

Specific indirect 5
WJAPSCG3
WJAPSC1S
CPOMSC54
RVCSTM36
MS_24MR 0.014 0.006 2.341 0.019

Specific indirect 6
WJAPSCG3
WJAPSC1S
CPOMSC54
RELSTM36
MS_24MR 0.000 0.001 -0.001 0.999

Specific indirect 7
WJAPSCG3
WJAPSC1S
WJMSSC54
RVCSTM36
MS_24MR 0.017 0.009 1.920 0.055

Specific indirect 8
WJAPSCG3
WJAPSC1S
WJMSSC54
RELSTM36
MS_24MR 0.002 0.001 1.277 0.201

Effects from MS_54MR to WJAPSCG3

Total 0.000 0.000 999.000 0.000
Total indirect 0.000 0.000 999.000 0.000

Effects from PLSASC54 to WJAPSCG3

Total 0.028 0.009 3.168 0.002
Total indirect 0.028 0.009 3.168 0.002

```
  Specific indirect 1
    WJAPSCG3
    WJAPSC1S
    PLSASC54           0.028      0.009      3.168      0.002


Effects from PLSESC54 to WJAPSCG3

  Total                0.014      0.008      1.789      0.074
  Total indirect       0.014      0.008      1.789      0.074

  Specific indirect 1
    WJAPSCG3
    WJAPSC1S
    PLSESC54           0.014      0.008      1.789      0.074


Effects from CPINCC54 to WJAPSCG3

  Total                0.000      0.000    999.000      0.000
  Total indirect       0.000      0.000    999.000      0.000


Effects from CPOMSC54 to WJAPSCG3

  Total               -0.044      0.015     -2.990      0.003
  Total indirect      -0.044      0.015     -2.990      0.003

  Specific indirect 1
    WJAPSCG3
    WJAPSC1S
    CPOMSC54          -0.044      0.015     -2.990      0.003


Effects from WJMSSC54 to WJAPSCG3

  Total                0.014      0.007      2.151      0.031
  Total indirect       0.014      0.007      2.151      0.031

  Specific indirect 1
    WJAPSCG3
    WJAPSC1S
    WJMSSC54           0.014      0.007      2.151      0.031


Effects from RVCSTM36 to WJAPSCG3

  Total                0.044      0.011      3.981      0.000
  Total indirect       0.044      0.011      3.981      0.000

  Specific indirect 1
    WJAPSCG3
    WJAPSC1S
    PLSASC54
```

```
    RVCSTM36           0.021      0.007      3.118      0.002

  Specific indirect 2
    WJAPSCG3
    WJAPSC1S
    PLSESC54
    RVCSTM36           0.009      0.005      1.760      0.078

  Specific indirect 3
    WJAPSCG3
    WJAPSC1S
    CPOMSC54
    RVCSTM36           0.006      0.002      2.744      0.006

  Specific indirect 4
    WJAPSCG3
    WJAPSC1S
    WJMSSC54
    RVCSTM36           0.008      0.004      2.096      0.036


Effects from RELSTM36 to WJAPSCG3

  Total                0.006      0.003      2.241      0.025
  Total indirect       0.006      0.003      2.241      0.025

  Specific indirect 1
    WJAPSCG3
    WJAPSC1S
    PLSASC54
    RELSTM36           0.002      0.001      1.822      0.068

  Specific indirect 2
    WJAPSCG3
    WJAPSC1S
    PLSESC54
    RELSTM36           0.003      0.002      1.665      0.096

  Specific indirect 3
    WJAPSCG3
    WJAPSC1S
    CPOMSC54
    RELSTM36           0.000      0.001     -0.001      0.999

  Specific indirect 4
    WJAPSCG3
    WJAPSC1S
    WJMSSC54
    RELSTM36           0.001      0.001      1.379      0.168


Effects from MS_24MR to MS_G1R

  Total                0.027      0.009      3.018      0.003
```

```
  Total indirect       0.027      0.009      3.018      0.003

  Specific indirect 1
    MS_G1R
    PLSASC54
    RVCSTM36
    MS_24MR            0.011      0.004      2.827      0.005

  Specific indirect 2
    MS_G1R
    PLSASC54
    RELSTM36
    MS_24MR            0.001      0.001      1.679      0.093

  Specific indirect 3
    MS_G1R
    MS_54MR
    MS_36MR
    MS_24MR            0.015      0.007      2.044      0.041


Effects from MS_54MR to MS_G1R

  Total                0.236      0.062      3.780      0.000
  Total indirect       0.000      0.000    999.000      0.000

  Direct
    MS_G1R
    MS_54MR            0.236      0.062      3.780      0.000


Effects from PLSASC54 to MS_G1R

  Total                0.007      0.002      3.882      0.000
  Total indirect       0.000      0.000      0.000      1.000

  Direct
    MS_G1R
    PLSASC54           0.007      0.002      3.882      0.000


Effects from PLSESC54 to MS_G1R

  Total                0.000      0.000      0.000      1.000
  Total indirect       0.000      0.000      0.000      1.000


Effects from CPINCC54 to MS_G1R

  Total                0.000      0.000    999.000      0.000
  Total indirect       0.000      0.000    999.000      0.000


Effects from CPOMSC54 to MS_G1R
```

```
  Total                0.000      0.000      0.000      1.000
  Total indirect       0.000      0.000      0.000      1.000


Effects from WJMSSC54 to MS_G1R

  Total                0.000      0.000     -1.706      0.088
  Total indirect       0.000      0.000     -1.706      0.088


Effects from RVCSTM36 to MS_G1R

  Total                0.005      0.001      3.834      0.000
  Total indirect       0.005      0.001      3.834      0.000

  Specific indirect 1
    MS_G1R
    PLSASC54
    RVCSTM36           0.005      0.001      3.834      0.000


Effects from RELSTM36 to MS_G1R

  Total                0.001      0.000      1.891      0.059
  Total indirect       0.001      0.000      1.891      0.059

  Specific indirect 1
    MS_G1R
    PLSASC54
    RELSTM36           0.001      0.000      1.891      0.059


Effects from MS_24MR to SLFR_G1R

  Total                0.008      0.003      3.070      0.002
  Total indirect       0.008      0.003      3.070      0.002

  Specific indirect 1
    SLFR_G1R
    CPOMSC54
    RVCSTM36
    MS_24MR            0.008      0.003      3.006      0.003

  Specific indirect 2
    SLFR_G1R
    CPOMSC54
    RELSTM36
    MS_24MR            0.000      0.001     -0.001      0.999


Effects from MS_54MR to SLFR_G1R

  Total                0.000      0.000    999.000      0.000
```

Total indirect 0.000 0.000 999.000 0.000

Effects from PLSASC54 to SLFR_G1R

```
  Total               0.000      0.000     -3.861      0.000
  Total indirect      0.000      0.000     -3.861      0.000
```

Effects from PLSESC54 to SLFR_G1R

```
  Total               0.000      0.000     -2.221      0.026
  Total indirect      0.000      0.000     -2.221      0.026
```

Effects from CPINCC54 to SLFR_G1R

```
  Total               0.000      0.000    999.000      0.000
  Total indirect      0.000      0.000    999.000      0.000
```

Effects from CPOMSC54 to SLFR_G1R

```
  Total              -0.024      0.005     -4.888      0.000
  Total indirect      0.000      0.000    -19.391      0.000

  Direct
    SLFR_G1R
    CPOMSC54         -0.024      0.005     -4.888      0.000
```

Effects from WJMSSC54 to SLFR_G1R

```
  Total               0.000      0.000     -2.532      0.011
  Total indirect      0.000      0.000     -2.532      0.011
```

Effects from RVCSTM36 to SLFR_G1R

```
  Total               0.004      0.001      3.969      0.000
  Total indirect      0.004      0.001      3.969      0.000

  Specific indirect 1
    SLFR_G1R
    CPOMSC54
    RVCSTM36          0.004      0.001      3.969      0.000
```

Effects from RELSTM36 to SLFR_G1R

```
  Total               0.000      0.000     -0.001      0.999
  Total indirect      0.000      0.000     -0.001      0.999

  Specific indirect 1
```

SLFR_G1R
CPOMSC54
RELSTM36 0.000 0.000 -0.001 0.999

Effects from MS_24MR to WJAPSC1S

Total 0.444 0.106 4.178 0.000
Total indirect 0.444 0.106 4.178 0.000

Specific indirect 1
WJAPSC1S
PLSASC54
RVCSTM36
MS_24MR 0.192 0.064 2.995 0.003

Specific indirect 2
WJAPSC1S
PLSASC54
RELSTM36
MS_24MR 0.016 0.009 1.738 0.082

Specific indirect 3
WJAPSC1S
PLSESC54
RVCSTM36
MS_24MR 0.082 0.044 1.891 0.059

Specific indirect 4
WJAPSC1S
PLSESC54
RELSTM36
MS_24MR 0.018 0.011 1.683 0.092

Specific indirect 5
WJAPSC1S
CPOMSC54
RVCSTM36
MS_24MR 0.059 0.023 2.576 0.010

Specific indirect 6
WJAPSC1S
CPOMSC54
RELSTM36
MS_24MR 0.000 0.006 -0.001 0.999

Specific indirect 7
WJAPSC1S
WJMSSC54
RVCSTM36
MS_24MR 0.069 0.033 2.115 0.034

Specific indirect 8
WJAPSC1S

WJMSSC54
RELSTM36
MS_24MR 0.008 0.006 1.329 0.184

Effects from MS_54MR to WJAPSC1S

Total 0.000 0.000 999.000 0.000
Total indirect 0.000 0.000 999.000 0.000

Effects from PLSASC54 to WJAPSC1S

Total 0.117 0.028 4.197 0.000
Total indirect 0.000 0.000 -1.658 0.097

Direct
WJAPSC1S
PLSASC54 0.117 0.028 4.197 0.000

Effects from PLSESC54 to WJAPSC1S

Total 0.058 0.028 2.076 0.038
Total indirect 0.000 0.000 0.000 1.000

Direct
WJAPSC1S
PLSESC54 0.058 0.028 2.076 0.038

Effects from CPINCC54 to WJAPSC1S

Total 0.000 0.000 999.000 0.000
Total indirect 0.000 0.000 999.000 0.000

Effects from CPOMSC54 to WJAPSC1S

Total -0.182 0.051 -3.599 0.000
Total indirect 0.000 0.000 0.000 1.000

Direct
WJAPSC1S
CPOMSC54 -0.182 0.051 -3.599 0.000

Effects from WJMSSC54 to WJAPSC1S

Total 0.059 0.024 2.428 0.015
Total indirect 0.000 0.000 -2.445 0.014

Direct
WJAPSC1S

WJMSSC54 0.059 0.024 2.428 0.015

Effects from RVCSTM36 to WJAPSC1S

Total 0.182 0.020 8.966 0.000
Total indirect 0.182 0.020 8.966 0.000

Specific indirect 1
WJAPSC1S
PLSASC54
RVCSTM36 0.087 0.021 4.104 0.000

Specific indirect 2
WJAPSC1S
PLSESC54
RVCSTM36 0.037 0.018 2.041 0.041

Specific indirect 3
WJAPSC1S
CPOMSC54
RVCSTM36 0.027 0.009 3.152 0.002

Specific indirect 4
WJAPSC1S
WJMSSC54
RVCSTM36 0.031 0.013 2.360 0.018

Effects from RELSTM36 to WJAPSC1S

Total 0.024 0.009 2.715 0.007
Total indirect 0.024 0.009 2.715 0.007

Specific indirect 1
WJAPSC1S
PLSASC54
RELSTM36 0.009 0.005 1.971 0.049

Specific indirect 2
WJAPSC1S
PLSESC54
RELSTM36 0.011 0.006 1.897 0.058

Specific indirect 3
WJAPSC1S
CPOMSC54
RELSTM36 0.000 0.004 -0.001 0.999

Specific indirect 4
WJAPSC1S
WJMSSC54
RELSTM36 0.005 0.003 1.446 0.148

Effects from MS_24MR to MS_54MR

Total	0.064	0.021	3.009	0.003
Total indirect	0.064	0.021	3.009	0.003

Specific indirect 1
MS_54MR
MS_36MR

MS_24MR	0.064	0.021	3.009	0.003

Effects from MS_24MR to PLSASC54

Total	1.782	0.379	4.706	0.000
Total indirect	1.782	0.379	4.706	0.000

Specific indirect 1
PLSASC54
RVCSTM36

MS_24MR	1.648	0.360	4.583	0.000

Specific indirect 2
PLSASC54
RELSTM36

MS_24MR	0.134	0.070	1.902	0.057

Effects from MS_24MR to PLSESC54

Total	1.712	0.365	4.686	0.000
Total indirect	1.712	0.365	4.686	0.000

Specific indirect 1
PLSESC54
RVCSTM36

MS_24MR	1.409	0.315	4.470	0.000

Specific indirect 2
PLSESC54
RELSTM36

MS_24MR	0.303	0.111	2.727	0.006

Effects from MS_24MR to CPINCC54

Total	-0.529	0.186	-2.838	0.005
Total indirect	-0.529	0.186	-2.838	0.005

Specific indirect 1
CPINCC54
RVCSTM36

MS_24MR	-0.712	0.198	-3.602	0.000

```
  Specific indirect 2
    CPINCC54
    RELSTM36
    MS_24MR            0.183      0.107      1.711      0.087


 Effects from MS_24MR to CPOMSC54

  Total               -0.326      0.086     -3.801      0.000
  Total indirect      -0.326      0.086     -3.801      0.000

  Specific indirect 1
    CPOMSC54
    RVCSTM36
    MS_24MR           -0.326      0.087     -3.732      0.000

  Specific indirect 2
    CPOMSC54
    RELSTM36
    MS_24MR            0.000      0.033      0.001      0.999


 Effects from MS_24MR to WJMSSC54

  Total                1.305      0.283      4.615      0.000
  Total indirect       1.305      0.283      4.615      0.000

  Specific indirect 1
    WJMSSC54
    RVCSTM36
    MS_24MR            1.176      0.265      4.433      0.000

  Specific indirect 2
    WJMSSC54
    RELSTM36
    MS_24MR            0.129      0.082      1.580      0.114



STANDARDIZED TOTAL, TOTAL INDIRECT, SPECIFIC INDIRECT, AND DIRECT EFFECTS


STDYX Standardization

                                                    Two-Tailed
                    Estimate       S.E.  Est./S.E.    P-Value

 Effects from MS_24MR to MS_15R

  Total                0.000      0.000      1.183      0.237
  Total indirect       0.000      0.000      1.183      0.237

  Specific indirect 1
    MS_15R
```

MS_G5R
MS_G3R
MS_G1R
PLSASC54
RVCSTM36
MS_24MR 0.000 0.000 1.191 0.234

Specific indirect 2
MS_15R
MS_G5R
MS_G3R
MS_G1R
PLSASC54
RELSTM36
MS_24MR 0.000 0.000 1.022 0.307

Specific indirect 3
MS_15R
MS_G5R
MS_G3R
MS_G1R
MS_54MR
MS_36MR
MS_24MR 0.000 0.000 1.077 0.281

Effects from MS_54MR to MS_15R

Total 0.001 0.001 1.223 0.221
Total indirect 0.001 0.001 1.223 0.221

Specific indirect 1
MS_15R
MS_G5R
MS_G3R
MS_G1R
MS_54MR 0.001 0.001 1.223 0.221

Effects from PLSASC54 to MS_15R

Total 0.000 0.000 1.269 0.204
Total indirect 0.000 0.000 1.269 0.204

Specific indirect 1
MS_15R
MS_G5R
MS_G3R
MS_G1R
PLSASC54 0.000 0.000 1.269 0.204

Effects from PLSESC54 to MS_15R

```
  Total                0.000       0.000       0.000       1.000
  Total indirect       0.000       0.000       0.000       1.000


Effects from CPINCC54 to MS_15R

  Total                0.000       0.000       0.000       1.000
  Total indirect       0.000       0.000       0.000       1.000


Effects from CPOMSC54 to MS_15R

  Total                0.000       0.000       0.000       1.000
  Total indirect       0.000       0.000       0.000       1.000


Effects from WJMSSC54 to MS_15R

  Total                0.000       0.000       0.000       1.000
  Total indirect       0.000       0.000       0.000       1.000


Effects from RVCSTM36 to MS_15R

  Total                0.000       0.000       1.272       0.204
  Total indirect       0.000       0.000       1.272       0.204

  Specific indirect 1
    MS_15R
    MS_G5R
    MS_G3R
    MS_G1R
    PLSASC54
    RVCSTM36           0.000       0.000       1.272       0.204


Effects from RELSTM36 to MS_15R

  Total                0.000       0.000       1.069       0.285
  Total indirect       0.000       0.000       1.069       0.285

  Specific indirect 1
    MS_15R
    MS_G5R
    MS_G3R
    MS_G1R
    PLSASC54
    RELSTM36           0.000       0.000       1.069       0.285


Effects from MS_24MR to SLFR_15R

  Total               -0.001       0.000      -1.673       0.094
  Total indirect      -0.001       0.000      -1.673       0.094
```

Specific indirect 1
SLFR_15R
SLFR_G5R
SLFR_G3R
SLFR_G1R
CPOMSC54
RVCSTM36

MS_24MR	0.000	0.000	-1.761	0.078

Specific indirect 2
SLFR_15R
SLFR_G5R
SLFR_G3R
SLFR_G1R
CPOMSC54
RELSTM36

MS_24MR	0.000	0.000	0.001	0.999

Specific indirect 3
SLFR_15R
SLFR_G5R
SLFR_G3R
WJAPSC1S
PLSASC54
RVCSTM36

MS_24MR	0.000	0.000	-1.323	0.186

Specific indirect 4
SLFR_15R
SLFR_G5R
SLFR_G3R
WJAPSC1S
PLSASC54
RELSTM36

MS_24MR	0.000	0.000	-1.133	0.257

Specific indirect 5
SLFR_15R
SLFR_G5R
SLFR_G3R
WJAPSC1S
PLSESC54
RVCSTM36

MS_24MR	0.000	0.000	-1.139	0.255

Specific indirect 6
SLFR_15R
SLFR_G5R
SLFR_G3R
WJAPSC1S
PLSESC54
RELSTM36

MS_24MR	0.000	0.000	-1.086	0.277

Specific indirect 7
SLFR_15R
SLFR_G5R
SLFR_G3R
WJAPSC1S
CPOMSC54
RVCSTM36
MS_24MR 0.000 0.000 -1.303 0.193

Specific indirect 8
SLFR_15R
SLFR_G5R
SLFR_G3R
WJAPSC1S
CPOMSC54
RELSTM36
MS_24MR 0.000 0.000 0.001 0.999

Specific indirect 9
SLFR_15R
SLFR_G5R
SLFR_G3R
WJAPSC1S
WJMSSC54
RVCSTM36
MS_24MR 0.000 0.000 -1.229 0.219

Specific indirect 10
SLFR_15R
SLFR_G5R
SLFR_G3R
WJAPSC1S
WJMSSC54
RELSTM36
MS_24MR 0.000 0.000 -1.014 0.311

Specific indirect 11
SLFR_15R
SLFR_G5R
WJPCSCG3
WJAPSC1S
PLSASC54
RVCSTM36
MS_24MR 0.000 0.000 -0.345 0.730

Specific indirect 12
SLFR_15R
SLFR_G5R
WJPCSCG3
WJAPSC1S
PLSASC54
RELSTM36
MS_24MR 0.000 0.000 -0.338 0.735

Specific indirect 13
SLFR_15R
SLFR_G5R
WJPCSCG3
WJAPSC1S
PLSESC54
RVCSTM36
MS_24MR 0.000 0.000 -0.336 0.737

Specific indirect 14
SLFR_15R
SLFR_G5R
WJPCSCG3
WJAPSC1S
PLSESC54
RELSTM36
MS_24MR 0.000 0.000 -0.332 0.740

Specific indirect 15
SLFR_15R
SLFR_G5R
WJPCSCG3
WJAPSC1S
CPOMSC54
RVCSTM36
MS_24MR 0.000 0.000 -0.343 0.732

Specific indirect 16
SLFR_15R
SLFR_G5R
WJPCSCG3
WJAPSC1S
CPOMSC54
RELSTM36
MS_24MR 0.000 0.000 0.001 0.999

Specific indirect 17
SLFR_15R
SLFR_G5R
WJPCSCG3
WJAPSC1S
WJMSSC54
RVCSTM36
MS_24MR 0.000 0.000 -0.343 0.732

Specific indirect 18
SLFR_15R
SLFR_G5R
WJPCSCG3
WJAPSC1S
WJMSSC54
RELSTM36
MS_24MR 0.000 0.000 -0.333 0.739

Specific indirect 19
SLFR_15R
WJAPSCG5
WJPCSCG3
WJAPSC1S
PLSASC54
RVCSTM36
MS_24MR 0.000 0.000 -0.409 0.683

Specific indirect 20
SLFR_15R
WJAPSCG5
WJPCSCG3
WJAPSC1S
PLSASC54
RELSTM36
MS_24MR 0.000 0.000 -0.401 0.689

Specific indirect 21
SLFR_15R
WJAPSCG5
WJPCSCG3
WJAPSC1S
PLSESC54
RVCSTM36
MS_24MR 0.000 0.000 -0.393 0.695

Specific indirect 22
SLFR_15R
WJAPSCG5
WJPCSCG3
WJAPSC1S
PLSESC54
RELSTM36
MS_24MR 0.000 0.000 -0.390 0.697

Specific indirect 23
SLFR_15R
WJAPSCG5
WJPCSCG3
WJAPSC1S
CPOMSC54
RVCSTM36
MS_24MR 0.000 0.000 -0.410 0.682

Specific indirect 24
SLFR_15R
WJAPSCG5
WJPCSCG3
WJAPSC1S
CPOMSC54
RELSTM36
MS_24MR 0.000 0.000 0.001 0.999

Specific indirect 25
SLFR_15R
WJAPSCG5
WJPCSCG3
WJAPSC1S
WJMSSC54
RVCSTM36
MS_24MR 0.000 0.000 -0.398 0.690

Specific indirect 26
SLFR_15R
WJAPSCG5
WJPCSCG3
WJAPSC1S
WJMSSC54
RELSTM36
MS_24MR 0.000 0.000 -0.389 0.697

Specific indirect 27
SLFR_15R
WJAPSCG5
WJAPSCG3
WJAPSC1S
PLSASC54
RVCSTM36
MS_24MR 0.000 0.000 -1.319 0.187

Specific indirect 28
SLFR_15R
WJAPSCG5
WJAPSCG3
WJAPSC1S
PLSASC54
RELSTM36
MS_24MR 0.000 0.000 -1.141 0.254

Specific indirect 29
SLFR_15R
WJAPSCG5
WJAPSCG3
WJAPSC1S
PLSESC54
RVCSTM36
MS_24MR 0.000 0.000 -1.152 0.249

Specific indirect 30
SLFR_15R
WJAPSCG5
WJAPSCG3
WJAPSC1S
PLSESC54
RELSTM36
MS_24MR 0.000 0.000 -1.109 0.268

Specific indirect 31
SLFR_15R
WJAPSCG5
WJAPSCG3
WJAPSC1S
CPOMSC54
RVCSTM36
MS_24MR 0.000 0.000 -1.355 0.175

Specific indirect 32
SLFR_15R
WJAPSCG5
WJAPSCG3
WJAPSC1S
CPOMSC54
RELSTM36
MS_24MR 0.000 0.000 0.001 0.999

Specific indirect 33
SLFR_15R
WJAPSCG5
WJAPSCG3
WJAPSC1S
WJMSSC54
RVCSTM36
MS_24MR 0.000 0.000 -1.289 0.197

Specific indirect 34
SLFR_15R
WJAPSCG5
WJAPSCG3
WJAPSC1S
WJMSSC54
RELSTM36
MS_24MR 0.000 0.000 -1.021 0.307

Effects from MS_54MR to SLFR_15R

Total 0.000 0.000 0.000 1.000
Total indirect 0.000 0.000 0.000 1.000

Effects from PLSASC54 to SLFR_15R

Total -0.003 0.002 -1.567 0.117
Total indirect -0.003 0.002 -1.567 0.117

Specific indirect 1
SLFR_15R
SLFR_G5R
SLFR_G3R
WJAPSC1S

PLSASC54 -0.001 0.000 -1.396 0.163

Specific indirect 2
SLFR_15R
SLFR_G5R
WJPCSCG3
WJAPSC1S
PLSASC54 0.000 0.000 -0.346 0.729

Specific indirect 3
SLFR_15R
WJAPSCG5
WJPCSCG3
WJAPSC1S
PLSASC54 0.000 0.000 -0.411 0.681

Specific indirect 4
SLFR_15R
WJAPSCG5
WJAPSCG3
WJAPSC1S
PLSASC54 -0.002 0.001 -1.398 0.162

Effects from PLSESC54 to SLFR_15R

Total -0.001 0.001 -1.279 0.201
Total indirect -0.001 0.001 -1.279 0.201

Specific indirect 1
SLFR_15R
SLFR_G5R
SLFR_G3R
WJAPSC1S
PLSESC54 0.000 0.000 -1.178 0.239

Specific indirect 2
SLFR_15R
SLFR_G5R
WJPCSCG3
WJAPSC1S
PLSESC54 0.000 0.000 -0.337 0.736

Specific indirect 3
SLFR_15R
WJAPSCG5
WJPCSCG3
WJAPSC1S
PLSESC54 0.000 0.000 -0.395 0.693

Specific indirect 4
SLFR_15R
WJAPSCG5
WJAPSCG3

WJAPSC1S
PLSESC54 -0.001 0.001 -1.199 0.231

Effects from CPINCC54 to SLFR_15R

Total	0.000	0.000	0.000	1.000
Total indirect	0.000	0.000	0.000	1.000

Effects from CPOMSC54 to SLFR_15R

Total	0.003	0.001	2.217	0.027
Total indirect	0.003	0.001	2.217	0.027

Specific indirect 1
SLFR_15R
SLFR_G5R
SLFR_G3R
SLFR_G1R
CPOMSC54 0.001 0.001 1.995 0.046

Specific indirect 2
SLFR_15R
SLFR_G5R
SLFR_G3R
WJAPSC1S
CPOMSC54 0.000 0.000 1.392 0.164

Specific indirect 3
SLFR_15R
SLFR_G5R
WJPCSCG3
WJAPSC1S
CPOMSC54 0.000 0.000 0.344 0.731

Specific indirect 4
SLFR_15R
WJAPSCG5
WJPCSCG3
WJAPSC1S
CPOMSC54 0.000 0.000 0.413 0.680

Specific indirect 5
SLFR_15R
WJAPSCG5
WJAPSCG3
WJAPSC1S
CPOMSC54 0.001 0.001 1.449 0.147

Effects from WJMSSC54 to SLFR_15R

Total	-0.001	0.001	-1.485	0.138

```
  Total indirect      -0.001      0.001     -1.485      0.138

  Specific indirect 1
    SLFR_15R
    SLFR_G5R
    SLFR_G3R
    WJAPSC1S
    WJMSSC54           0.000      0.000     -1.295      0.195

  Specific indirect 2
    SLFR_15R
    SLFR_G5R
    WJPCSCG3
    WJAPSC1S
    WJMSSC54           0.000      0.000     -0.344      0.731

  Specific indirect 3
    SLFR_15R
    WJAPSCG5
    WJPCSCG3
    WJAPSC1S
    WJMSSC54           0.000      0.000     -0.401      0.688

  Specific indirect 4
    SLFR_15R
    WJAPSCG5
    WJAPSCG3
    WJAPSC1S
    WJMSSC54          -0.001      0.001     -1.367      0.172


Effects from RVCSTM36 to SLFR_15R

  Total               -0.004      0.002     -1.820      0.069
  Total indirect      -0.004      0.002     -1.820      0.069

  Specific indirect 1
    SLFR_15R
    SLFR_G5R
    SLFR_G3R
    SLFR_G1R
    CPOMSC54
    RVCSTM36           0.000      0.000     -1.914      0.056

  Specific indirect 2
    SLFR_15R
    SLFR_G5R
    SLFR_G3R
    WJAPSC1S
    PLSASC54
    RVCSTM36           0.000      0.000     -1.392      0.164

  Specific indirect 3
    SLFR_15R
```

SLFR_G5R
SLFR_G3R
WJAPSC1S
PLSESC54
RVCSTM36 0.000 0.000 -1.173 0.241

Specific indirect 4
SLFR_15R
SLFR_G5R
SLFR_G3R
WJAPSC1S
CPOMSC54
RVCSTM36 0.000 0.000 -1.368 0.171

Specific indirect 5
SLFR_15R
SLFR_G5R
SLFR_G3R
WJAPSC1S
WJMSSC54
RVCSTM36 0.000 0.000 -1.279 0.201

Specific indirect 6
SLFR_15R
SLFR_G5R
WJPCSCG3
WJAPSC1S
PLSASC54
RVCSTM36 0.000 0.000 -0.347 0.729

Specific indirect 7
SLFR_15R
SLFR_G5R
WJPCSCG3
WJAPSC1S
PLSESC54
RVCSTM36 0.000 0.000 -0.337 0.736

Specific indirect 8
SLFR_15R
SLFR_G5R
WJPCSCG3
WJAPSC1S
CPOMSC54
RVCSTM36 0.000 0.000 -0.344 0.731

Specific indirect 9
SLFR_15R
SLFR_G5R
WJPCSCG3
WJAPSC1S
WJMSSC54
RVCSTM36 0.000 0.000 -0.344 0.731

Specific indirect 10
SLFR_15R
WJAPSCG5
WJPCSCG3
WJAPSC1S
PLSASC54
RVCSTM36 0.000 0.000 -0.411 0.681

Specific indirect 11
SLFR_15R
WJAPSCG5
WJPCSCG3
WJAPSC1S
PLSESC54
RVCSTM36 0.000 0.000 -0.394 0.693

Specific indirect 12
SLFR_15R
WJAPSCG5
WJPCSCG3
WJAPSC1S
CPOMSC54
RVCSTM36 0.000 0.000 -0.412 0.680

Specific indirect 13
SLFR_15R
WJAPSCG5
WJPCSCG3
WJAPSC1S
WJMSSC54
RVCSTM36 0.000 0.000 -0.400 0.689

Specific indirect 14
SLFR_15R
WJAPSCG5
WJAPSCG3
WJAPSC1S
PLSASC54
RVCSTM36 -0.001 0.001 -1.389 0.165

Specific indirect 15
SLFR_15R
WJAPSCG5
WJAPSCG3
WJAPSC1S
PLSESC54
RVCSTM36 -0.001 0.000 -1.188 0.235

Specific indirect 16
SLFR_15R
WJAPSCG5
WJAPSCG3
WJAPSC1S
CPOMSC54

RVCSTM36 0.000 0.000 -1.430 0.153

Specific indirect 17
SLFR_15R
WJAPSCG5
WJAPSCG3
WJAPSC1S
WJMSSC54
RVCSTM36 0.000 0.000 -1.348 0.178

Effects from RELSTM36 to SLFR_15R

Total 0.000 0.000 -1.351 0.177
Total indirect 0.000 0.000 -1.351 0.177

Specific indirect 1
SLFR_15R
SLFR_G5R
SLFR_G3R
SLFR_G1R
CPOMSC54
RELSTM36 0.000 0.000 0.001 0.999

Specific indirect 2
SLFR_15R
SLFR_G5R
SLFR_G3R
WJAPSC1S
PLSASC54
RELSTM36 0.000 0.000 -1.179 0.239

Specific indirect 3
SLFR_15R
SLFR_G5R
SLFR_G3R
WJAPSC1S
PLSESC54
RELSTM36 0.000 0.000 -1.128 0.259

Specific indirect 4
SLFR_15R
SLFR_G5R
SLFR_G3R
WJAPSC1S
CPOMSC54
RELSTM36 0.000 0.000 0.001 0.999

Specific indirect 5
SLFR_15R
SLFR_G5R
SLFR_G3R
WJAPSC1S
WJMSSC54

```
  RELSTM36          0.000      0.000     -1.056      0.291

Specific indirect 6
  SLFR_15R
  SLFR_G5R
  WJPCSCG3
  WJAPSC1S
  PLSASC54
  RELSTM36          0.000      0.000     -0.341      0.733

Specific indirect 7
  SLFR_15R
  SLFR_G5R
  WJPCSCG3
  WJAPSC1S
  PLSESC54
  RELSTM36          0.000      0.000     -0.335      0.738

Specific indirect 8
  SLFR_15R
  SLFR_G5R
  WJPCSCG3
  WJAPSC1S
  CPOMSC54
  RELSTM36          0.000      0.000      0.001      0.999

Specific indirect 9
  SLFR_15R
  SLFR_G5R
  WJPCSCG3
  WJAPSC1S
  WJMSSC54
  RELSTM36          0.000      0.000     -0.336      0.737

Specific indirect 10
  SLFR_15R
  WJAPSCG5
  WJPCSCG3
  WJAPSC1S
  PLSASC54
  RELSTM36          0.000      0.000     -0.404      0.686

Specific indirect 11
  SLFR_15R
  WJAPSCG5
  WJPCSCG3
  WJAPSC1S
  PLSESC54
  RELSTM36          0.000      0.000     -0.393      0.694

Specific indirect 12
  SLFR_15R
  WJAPSCG5
  WJPCSCG3
```

WJAPSC1S
CPOMSC54
RELSTM36 0.000 0.000 0.001 0.999

Specific indirect 13
SLFR_15R
WJAPSCG5
WJPCSCG3
WJAPSC1S
WJMSSC54
RELSTM36 0.000 0.000 -0.393 0.695

Specific indirect 14
SLFR_15R
WJAPSCG5
WJAPSCG3
WJAPSC1S
PLSASC54
RELSTM36 0.000 0.000 -1.194 0.232

Specific indirect 15
SLFR_15R
WJAPSCG5
WJAPSCG3
WJAPSC1S
PLSESC54
RELSTM36 0.000 0.000 -1.159 0.246

Specific indirect 16
SLFR_15R
WJAPSCG5
WJAPSCG3
WJAPSC1S
CPOMSC54
RELSTM36 0.000 0.000 0.001 0.999

Specific indirect 17
SLFR_15R
WJAPSCG5
WJAPSCG3
WJAPSC1S
WJMSSC54
RELSTM36 0.000 0.000 -1.070 0.285

Effects from MS_24MR to WJPCSCX5

Total 0.000 0.001 0.592 0.554
Total indirect 0.000 0.001 0.592 0.554

Specific indirect 1
WJPCSCX5
SLFR_G5R
SLFR_G3R

SLFR_G1R
CPOMSC54
RVCSTM36
MS_24MR 0.000 0.000 1.833 0.067

Specific indirect 2
WJPCSCX5
SLFR_G5R
SLFR_G3R
SLFR_G1R
CPOMSC54
RELSTM36
MS_24MR 0.000 0.000 -0.001 0.999

Specific indirect 3
WJPCSCX5
SLFR_G5R
SLFR_G3R
WJAPSC1S
PLSASC54
RVCSTM36
MS_24MR 0.000 0.000 1.447 0.148

Specific indirect 4
WJPCSCX5
SLFR_G5R
SLFR_G3R
WJAPSC1S
PLSASC54
RELSTM36
MS_24MR 0.000 0.000 1.186 0.236

Specific indirect 5
WJPCSCX5
SLFR_G5R
SLFR_G3R
WJAPSC1S
PLSESC54
RVCSTM36
MS_24MR 0.000 0.000 1.217 0.224

Specific indirect 6
WJPCSCX5
SLFR_G5R
SLFR_G3R
WJAPSC1S
PLSESC54
RELSTM36
MS_24MR 0.000 0.000 1.141 0.254

Specific indirect 7
WJPCSCX5
SLFR_G5R
SLFR_G3R

WJAPSC1S
CPOMSC54
RVCSTM36
MS_24MR 0.000 0.000 1.416 0.157

Specific indirect 8
WJPCSCX5
SLFR_G5R
SLFR_G3R
WJAPSC1S
CPOMSC54
RELSTM36
MS_24MR 0.000 0.000 -0.001 0.999

Specific indirect 9
WJPCSCX5
SLFR_G5R
SLFR_G3R
WJAPSC1S
WJMSSC54
RVCSTM36
MS_24MR 0.000 0.000 1.311 0.190

Specific indirect 10
WJPCSCX5
SLFR_G5R
SLFR_G3R
WJAPSC1S
WJMSSC54
RELSTM36
MS_24MR 0.000 0.000 1.039 0.299

Specific indirect 11
WJPCSCX5
SLFR_G5R
WJPCSCG3
WJAPSC1S
PLSASC54
RVCSTM36
MS_24MR 0.000 0.000 0.346 0.730

Specific indirect 12
WJPCSCX5
SLFR_G5R
WJPCSCG3
WJAPSC1S
PLSASC54
RELSTM36
MS_24MR 0.000 0.000 0.338 0.735

Specific indirect 13
WJPCSCX5
SLFR_G5R
WJPCSCG3

WJAPSC1S
PLSESC54
RVCSTM36
MS_24MR 0.000 0.000 0.336 0.737

Specific indirect 14
WJPCSCX5
SLFR_G5R
WJPCSCG3
WJAPSC1S
PLSESC54
RELSTM36
MS_24MR 0.000 0.000 0.332 0.740

Specific indirect 15
WJPCSCX5
SLFR_G5R
WJPCSCG3
WJAPSC1S
CPOMSC54
RVCSTM36
MS_24MR 0.000 0.000 0.343 0.732

Specific indirect 16
WJPCSCX5
SLFR_G5R
WJPCSCG3
WJAPSC1S
CPOMSC54
RELSTM36
MS_24MR 0.000 0.000 -0.001 0.999

Specific indirect 17
WJPCSCX5
SLFR_G5R
WJPCSCG3
WJAPSC1S
WJMSSC54
RVCSTM36
MS_24MR 0.000 0.000 0.343 0.732

Specific indirect 18
WJPCSCX5
SLFR_G5R
WJPCSCG3
WJAPSC1S
WJMSSC54
RELSTM36
MS_24MR 0.000 0.000 0.333 0.739

Specific indirect 19
WJPCSCX5
WJPCSCG5
WJPCSCG3

WJAPSC1S
PLSASC54
RVCSTM36
MS_24MR 0.000 0.000 -1.532 0.125

Specific indirect 20
WJPCSCX5
WJPCSCG5
WJPCSCG3
WJAPSC1S
PLSASC54
RELSTM36
MS_24MR 0.000 0.000 -1.219 0.223

Specific indirect 21
WJPCSCX5
WJPCSCG5
WJPCSCG3
WJAPSC1S
PLSESC54
RVCSTM36
MS_24MR 0.000 0.000 -1.223 0.221

Specific indirect 22
WJPCSCX5
WJPCSCG5
WJPCSCG3
WJAPSC1S
PLSESC54
RELSTM36
MS_24MR 0.000 0.000 -1.161 0.245

Specific indirect 23
WJPCSCX5
WJPCSCG5
WJPCSCG3
WJAPSC1S
CPOMSC54
RVCSTM36
MS_24MR 0.000 0.000 -1.437 0.151

Specific indirect 24
WJPCSCX5
WJPCSCG5
WJPCSCG3
WJAPSC1S
CPOMSC54
RELSTM36
MS_24MR 0.000 0.000 0.001 0.999

Specific indirect 25
WJPCSCX5
WJPCSCG5
WJPCSCG3

WJAPSC1S
WJMSSC54
RVCSTM36
MS_24MR 0.000 0.000 -1.285 0.199

Specific indirect 26
WJPCSCX5
WJPCSCG5
WJPCSCG3
WJAPSC1S
WJMSSC54
RELSTM36
MS_24MR 0.000 0.000 -1.017 0.309

Specific indirect 27
WJPCSCX5
WJPCSCG5
WJAPSCG3
WJAPSC1S
PLSASC54
RVCSTM36
MS_24MR 0.000 0.000 1.202 0.229

Specific indirect 28
WJPCSCX5
WJPCSCG5
WJAPSCG3
WJAPSC1S
PLSASC54
RELSTM36
MS_24MR 0.000 0.000 1.054 0.292

Specific indirect 29
WJPCSCX5
WJPCSCG5
WJAPSCG3
WJAPSC1S
PLSESC54
RVCSTM36
MS_24MR 0.000 0.000 1.046 0.296

Specific indirect 30
WJPCSCX5
WJPCSCG5
WJAPSCG3
WJAPSC1S
PLSESC54
RELSTM36
MS_24MR 0.000 0.000 1.008 0.313

Specific indirect 31
WJPCSCX5
WJPCSCG5
WJAPSCG3

WJAPSC1S
CPOMSC54
RVCSTM36

MS_24MR	0.000	0.000	1.226	0.220

Specific indirect 32
WJPCSCX5
WJPCSCG5
WJAPSCG3
WJAPSC1S
CPOMSC54
RELSTM36

MS_24MR	0.000	0.000	-0.001	0.999

Specific indirect 33
WJPCSCX5
WJPCSCG5
WJAPSCG3
WJAPSC1S
WJMSSC54
RVCSTM36

MS_24MR	0.000	0.000	1.118	0.264

Specific indirect 34
WJPCSCX5
WJPCSCG5
WJAPSCG3
WJAPSC1S
WJMSSC54
RELSTM36

MS_24MR	0.000	0.000	0.942	0.346

Specific indirect 35
WJPCSCX5
WJAPSCG5
WJPCSCG3
WJAPSC1S
PLSASC54
RVCSTM36

MS_24MR	0.000	0.000	-0.510	0.610

Specific indirect 36
WJPCSCX5
WJAPSCG5
WJPCSCG3
WJAPSC1S
PLSASC54
RELSTM36

MS_24MR	0.000	0.000	-0.492	0.623

Specific indirect 37
WJPCSCX5
WJAPSCG5
WJPCSCG3

WJAPSC1S
PLSESC54
RVCSTM36
MS_24MR 0.000 0.000 -0.479 0.632

Specific indirect 38
WJPCSCX5
WJAPSCG5
WJPCSCG3
WJAPSC1S
PLSESC54
RELSTM36
MS_24MR 0.000 0.000 -0.474 0.635

Specific indirect 39
WJPCSCX5
WJAPSCG5
WJPCSCG3
WJAPSC1S
CPOMSC54
RVCSTM36
MS_24MR 0.000 0.000 -0.499 0.618

Specific indirect 40
WJPCSCX5
WJAPSCG5
WJPCSCG3
WJAPSC1S
CPOMSC54
RELSTM36
MS_24MR 0.000 0.000 0.001 0.999

Specific indirect 41
WJPCSCX5
WJAPSCG5
WJPCSCG3
WJAPSC1S
WJMSSC54
RVCSTM36
MS_24MR 0.000 0.000 -0.484 0.628

Specific indirect 42
WJPCSCX5
WJAPSCG5
WJPCSCG3
WJAPSC1S
WJMSSC54
RELSTM36
MS_24MR 0.000 0.000 -0.464 0.642

Specific indirect 43
WJPCSCX5
WJAPSCG5
WJAPSCG3

```
  WJAPSC1S
  PLSASC54
  RVCSTM36
  MS_24MR            0.000      0.000     -0.799      0.424

 Specific indirect 44
  WJPCSCX5
  WJAPSCG5
  WJAPSCG3
  WJAPSC1S
  PLSASC54
  RELSTM36
  MS_24MR            0.000      0.000     -0.745      0.456

 Specific indirect 45
  WJPCSCX5
  WJAPSCG5
  WJAPSCG3
  WJAPSC1S
  PLSESC54
  RVCSTM36
  MS_24MR            0.000      0.000     -0.756      0.450

 Specific indirect 46
  WJPCSCX5
  WJAPSCG5
  WJAPSCG3
  WJAPSC1S
  PLSESC54
  RELSTM36
  MS_24MR            0.000      0.000     -0.745      0.457

 Specific indirect 47
  WJPCSCX5
  WJAPSCG5
  WJAPSCG3
  WJAPSC1S
  CPOMSC54
  RVCSTM36
  MS_24MR            0.000      0.000     -0.758      0.449

 Specific indirect 48
  WJPCSCX5
  WJAPSCG5
  WJAPSCG3
  WJAPSC1S
  CPOMSC54
  RELSTM36
  MS_24MR            0.000      0.000      0.001      0.999

 Specific indirect 49
  WJPCSCX5
  WJAPSCG5
  WJAPSCG3
```

WJAPSC1S
WJMSSC54
RVCSTM36
MS_24MR 0.000 0.000 -0.770 0.441

Specific indirect 50
WJPCSCX5
WJAPSCG5
WJAPSCG3
WJAPSC1S
WJMSSC54
RELSTM36
MS_24MR 0.000 0.000 -0.688 0.492

Effects from MS_54MR to WJPCSCX5

Total	0.000	0.000	0.000	1.000
Total indirect	0.000	0.000	0.000	1.000

Effects from PLSASC54 to WJPCSCX5

Total	0.001	0.003	0.493	0.622
Total indirect	0.001	0.003	0.493	0.622

Specific indirect 1
WJPCSCX5
SLFR_G5R
SLFR_G3R
WJAPSC1S
PLSASC54 0.001 0.000 1.548 0.122

Specific indirect 2
WJPCSCX5
SLFR_G5R
WJPCSCG3
WJAPSC1S
PLSASC54 0.000 0.000 0.347 0.729

Specific indirect 3
WJPCSCX5
WJPCSCG5
WJPCSCG3
WJAPSC1S
PLSASC54 -0.001 0.001 -1.638 0.101

Specific indirect 4
WJPCSCX5
WJPCSCG5
WJAPSCG3
WJAPSC1S
PLSASC54 0.003 0.002 1.252 0.211

Specific indirect 5
WJPCSCX5
WJAPSCG5
WJPCSCG3
WJAPSC1S
PLSASC54 0.000 0.000 -0.512 0.609

Specific indirect 6
WJPCSCX5
WJAPSCG5
WJAPSCG3
WJAPSC1S
PLSASC54 -0.001 0.001 -0.808 0.419

Effects from PLSESC54 to WJPCSCX5

Total 0.001 0.002 0.479 0.632
Total indirect 0.001 0.002 0.479 0.632

Specific indirect 1
WJPCSCX5
SLFR_G5R
SLFR_G3R
WJAPSC1S
PLSESC54 0.000 0.000 1.269 0.205

Specific indirect 2
WJPCSCX5
SLFR_G5R
WJPCSCG3
WJAPSC1S
PLSESC54 0.000 0.000 0.338 0.736

Specific indirect 3
WJPCSCX5
WJPCSCG5
WJPCSCG3
WJAPSC1S
PLSESC54 0.000 0.000 -1.274 0.203

Specific indirect 4
WJPCSCX5
WJPCSCG5
WJAPSCG3
WJAPSC1S
PLSESC54 0.001 0.001 1.075 0.282

Specific indirect 5
WJPCSCX5
WJAPSCG5
WJPCSCG3
WJAPSC1S
PLSESC54 0.000 0.000 -0.481 0.630

Specific indirect 6
WJPCSCX5
WJAPSCG5
WJAPSCG3
WJAPSC1S
PLSESC54 -0.001 0.001 -0.762 0.446

Effects from CPINCC54 to WJPCSCX5

Total 0.000 0.000 0.000 1.000
Total indirect 0.000 0.000 0.000 1.000

Effects from CPOMSC54 to WJPCSCX5

Total -0.002 0.002 -1.189 0.235
Total indirect -0.002 0.002 -1.189 0.235

Specific indirect 1
WJPCSCX5
SLFR_G5R
SLFR_G3R
SLFR_G1R
CPOMSC54 -0.001 0.001 -2.107 0.035

Specific indirect 2
WJPCSCX5
SLFR_G5R
SLFR_G3R
WJAPSC1S
CPOMSC54 0.000 0.000 -1.534 0.125

Specific indirect 3
WJPCSCX5
SLFR_G5R
WJPCSCG3
WJAPSC1S
CPOMSC54 0.000 0.000 -0.344 0.731

Specific indirect 4
WJPCSCX5
WJPCSCG5
WJPCSCG3
WJAPSC1S
CPOMSC54 0.001 0.000 1.552 0.121

Specific indirect 5
WJPCSCX5
WJPCSCG5
WJAPSCG3
WJAPSC1S
CPOMSC54 -0.002 0.001 -1.287 0.198

Specific indirect 6
WJPCSCX5
WJAPSCG5
WJPCSCG3
WJAPSC1S
CPOMSC54 0.000 0.000 0.503 0.615

Specific indirect 7
WJPCSCX5
WJAPSCG5
WJAPSCG3
WJAPSC1S
CPOMSC54 0.001 0.001 0.771 0.441

Effects from WJMSSC54 to WJPCSCX5

Total 0.001 0.001 0.491 0.623
Total indirect 0.001 0.001 0.491 0.623

Specific indirect 1
WJPCSCX5
SLFR_G5R
SLFR_G3R
WJAPSC1S
WJMSSC54 0.000 0.000 1.393 0.164

Specific indirect 2
WJPCSCX5
SLFR_G5R
WJPCSCG3
WJAPSC1S
WJMSSC54 0.000 0.000 0.345 0.730

Specific indirect 3
WJPCSCX5
WJPCSCG5
WJPCSCG3
WJAPSC1S
WJMSSC54 0.000 0.000 -1.352 0.176

Specific indirect 4
WJPCSCX5
WJPCSCG5
WJAPSCG3
WJAPSC1S
WJMSSC54 0.001 0.001 1.163 0.245

Specific indirect 5
WJPCSCX5
WJAPSCG5
WJPCSCG3
WJAPSC1S

WJMSSC54 0.000 0.000 -0.487 0.627

Specific indirect 6
WJPCSCX5
WJAPSCG5
WJAPSCG3
WJAPSC1S
WJMSSC54 0.000 0.001 -0.775 0.438

Effects from RVCSTM36 to WJPCSCX5

Total 0.002 0.004 0.609 0.542
Total indirect 0.002 0.004 0.609 0.542

Specific indirect 1
WJPCSCX5
SLFR_G5R
SLFR_G3R
SLFR_G1R
CPOMSC54
RVCSTM36 0.000 0.000 2.013 0.044

Specific indirect 2
WJPCSCX5
SLFR_G5R
SLFR_G3R
WJAPSC1S
PLSASC54
RVCSTM36 0.000 0.000 1.542 0.123

Specific indirect 3
WJPCSCX5
SLFR_G5R
SLFR_G3R
WJAPSC1S
PLSESC54
RVCSTM36 0.000 0.000 1.260 0.208

Specific indirect 4
WJPCSCX5
SLFR_G5R
SLFR_G3R
WJAPSC1S
CPOMSC54
RVCSTM36 0.000 0.000 1.502 0.133

Specific indirect 5
WJPCSCX5
SLFR_G5R
SLFR_G3R
WJAPSC1S
WJMSSC54
RVCSTM36 0.000 0.000 1.373 0.170

Specific indirect 6
WJPCSCX5
SLFR_G5R
WJPCSCG3
WJAPSC1S
PLSASC54
RVCSTM36 0.000 0.000 0.347 0.729

Specific indirect 7
WJPCSCX5
SLFR_G5R
WJPCSCG3
WJAPSC1S
PLSESC54
RVCSTM36 0.000 0.000 0.337 0.736

Specific indirect 8
WJPCSCX5
SLFR_G5R
WJPCSCG3
WJAPSC1S
CPOMSC54
RVCSTM36 0.000 0.000 0.344 0.731

Specific indirect 9
WJPCSCX5
SLFR_G5R
WJPCSCG3
WJAPSC1S
WJMSSC54
RVCSTM36 0.000 0.000 0.344 0.731

Specific indirect 10
WJPCSCX5
WJPCSCG5
WJPCSCG3
WJAPSC1S
PLSASC54
RVCSTM36 -0.001 0.000 -1.635 0.102

Specific indirect 11
WJPCSCX5
WJPCSCG5
WJPCSCG3
WJAPSC1S
PLSESC54
RVCSTM36 0.000 0.000 -1.262 0.207

Specific indirect 12
WJPCSCX5
WJPCSCG5
WJPCSCG3
WJAPSC1S

CPOMSC54				
RVCSTM36	0.000	0.000	-1.519	0.129

Specific indirect 13

WJPCSCX5				
WJPCSCG5				
WJPCSCG3				
WJAPSC1S				
WJMSSC54				
RVCSTM36	0.000	0.000	-1.338	0.181

Specific indirect 14

WJPCSCX5				
WJPCSCG5				
WJAPSCG3				
WJAPSC1S				
PLSASC54				
RVCSTM36	0.002	0.001	1.248	0.212

Specific indirect 15

WJPCSCX5				
WJPCSCG5				
WJAPSCG3				
WJAPSC1S				
PLSESC54				
RVCSTM36	0.001	0.001	1.069	0.285

Specific indirect 16

WJPCSCX5				
WJPCSCG5				
WJAPSCG3				
WJAPSC1S				
CPOMSC54				
RVCSTM36	0.000	0.000	1.273	0.203

Specific indirect 17

WJPCSCX5				
WJPCSCG5				
WJAPSCG3				
WJAPSC1S				
WJMSSC54				
RVCSTM36	0.001	0.001	1.150	0.250

Specific indirect 18

WJPCSCX5				
WJAPSCG5				
WJPCSCG3				
WJAPSC1S				
PLSASC54				
RVCSTM36	0.000	0.000	-0.512	0.609

Specific indirect 19

WJPCSCX5
WJAPSCG5

WJPCSCG3
WJAPSC1S
PLSESC54
RVCSTM36 0.000 0.000 -0.481 0.631

Specific indirect 20
WJPCSCX5
WJAPSCG5
WJPCSCG3
WJAPSC1S
CPOMSC54
RVCSTM36 0.000 0.000 -0.501 0.616

Specific indirect 21
WJPCSCX5
WJAPSCG5
WJPCSCG3
WJAPSC1S
WJMSSC54
RVCSTM36 0.000 0.000 -0.486 0.627

Specific indirect 22
WJPCSCX5
WJAPSCG5
WJAPSCG3
WJAPSC1S
PLSASC54
RVCSTM36 -0.001 0.001 -0.808 0.419

Specific indirect 23
WJPCSCX5
WJAPSCG5
WJAPSCG3
WJAPSC1S
PLSESC54
RVCSTM36 0.000 0.000 -0.761 0.446

Specific indirect 24
WJPCSCX5
WJAPSCG5
WJAPSCG3
WJAPSC1S
CPOMSC54
RVCSTM36 0.000 0.000 -0.765 0.444

Specific indirect 25
WJPCSCX5
WJAPSCG5
WJAPSCG3
WJAPSC1S
WJMSSC54
RVCSTM36 0.000 0.000 -0.777 0.437

Effects from RELSTM36 to WJPCSCX5

```
 Total              0.000      0.000      0.476      0.634
 Total indirect       0.000      0.000      0.476      0.634

 Specific indirect 1
  WJPCSCX5
  SLFR_G5R
  SLFR_G3R
  SLFR_G1R
  CPOMSC54
  RELSTM36           0.000      0.000     -0.001      0.999

 Specific indirect 2
  WJPCSCX5
  SLFR_G5R
  SLFR_G3R
  WJAPSC1S
  PLSASC54
  RELSTM36           0.000      0.000      1.261      0.207

 Specific indirect 3
  WJPCSCX5
  SLFR_G5R
  SLFR_G3R
  WJAPSC1S
  PLSESC54
  RELSTM36           0.000      0.000      1.211      0.226

 Specific indirect 4
  WJPCSCX5
  SLFR_G5R
  SLFR_G3R
  WJAPSC1S
  CPOMSC54
  RELSTM36           0.000      0.000     -0.001      0.999

 Specific indirect 5
  WJPCSCX5
  SLFR_G5R
  SLFR_G3R
  WJAPSC1S
  WJMSSC54
  RELSTM36           0.000      0.000      1.100      0.271

 Specific indirect 6
  WJPCSCX5
  SLFR_G5R
  WJPCSCG3
  WJAPSC1S
  PLSASC54
  RELSTM36           0.000      0.000      0.341      0.733

 Specific indirect 7
```

WJPCSCX5
SLFR_G5R
WJPCSCG3
WJAPSC1S
PLSESC54
RELSTM36 0.000 0.000 0.335 0.737

Specific indirect 8
WJPCSCX5
SLFR_G5R
WJPCSCG3
WJAPSC1S
CPOMSC54
RELSTM36 0.000 0.000 -0.001 0.999

Specific indirect 9
WJPCSCX5
SLFR_G5R
WJPCSCG3
WJAPSC1S
WJMSSC54
RELSTM36 0.000 0.000 0.336 0.737

Specific indirect 10
WJPCSCX5
WJPCSCG5
WJPCSCG3
WJAPSC1S
PLSASC54
RELSTM36 0.000 0.000 -1.294 0.196

Specific indirect 11
WJPCSCX5
WJPCSCG5
WJPCSCG3
WJAPSC1S
PLSESC54
RELSTM36 0.000 0.000 -1.228 0.220

Specific indirect 12
WJPCSCX5
WJPCSCG5
WJPCSCG3
WJAPSC1S
CPOMSC54
RELSTM36 0.000 0.000 0.001 0.999

Specific indirect 13
WJPCSCX5
WJPCSCG5
WJPCSCG3
WJAPSC1S
WJMSSC54
RELSTM36 0.000 0.000 -1.070 0.285

Specific indirect 14
WJPCSCX5
WJPCSCG5
WJAPSCG3
WJAPSC1S
PLSASC54
RELSTM36 0.000 0.000 1.094 0.274

Specific indirect 15
WJPCSCX5
WJPCSCG5
WJAPSCG3
WJAPSC1S
PLSESC54
RELSTM36 0.000 0.000 1.045 0.296

Specific indirect 16
WJPCSCX5
WJPCSCG5
WJAPSCG3
WJAPSC1S
CPOMSC54
RELSTM36 0.000 0.000 -0.001 0.999

Specific indirect 17
WJPCSCX5
WJPCSCG5
WJAPSCG3
WJAPSC1S
WJMSSC54
RELSTM36 0.000 0.000 0.979 0.328

Specific indirect 18
WJPCSCX5
WJAPSCG5
WJPCSCG3
WJAPSC1S
PLSASC54
RELSTM36 0.000 0.000 -0.497 0.619

Specific indirect 19
WJPCSCX5
WJAPSCG5
WJPCSCG3
WJAPSC1S
PLSESC54
RELSTM36 0.000 0.000 -0.479 0.632

Specific indirect 20
WJPCSCX5
WJAPSCG5
WJPCSCG3
WJAPSC1S

CPOMSC54
RELSTM36 0.000 0.000 0.001 0.999

Specific indirect 21
WJPCSCX5
WJAPSCG5
WJPCSCG3
WJAPSC1S
WJMSSC54
RELSTM36 0.000 0.000 -0.469 0.639

Specific indirect 22
WJPCSCX5
WJAPSCG5
WJAPSCG3
WJAPSC1S
PLSASC54
RELSTM36 0.000 0.000 -0.754 0.451

Specific indirect 23
WJPCSCX5
WJAPSCG5
WJAPSCG3
WJAPSC1S
PLSESC54
RELSTM36 0.000 0.000 -0.754 0.451

Specific indirect 24
WJPCSCX5
WJAPSCG5
WJAPSCG3
WJAPSC1S
CPOMSC54
RELSTM36 0.000 0.000 0.001 0.999

Specific indirect 25
WJPCSCX5
WJAPSCG5
WJAPSCG3
WJAPSC1S
WJMSSC54
RELSTM36 0.000 0.000 -0.698 0.485

Effects from MS_24MR to WJAPSCX5

Total 0.001 0.001 1.013 0.311
Total indirect 0.001 0.001 1.013 0.311

Specific indirect 1
WJAPSCX5
SLFR_G5R
SLFR_G3R
SLFR_G1R

CPOMSC54
RVCSTM36
MS_24MR 0.000 0.000 1.890 0.059

Specific indirect 2
WJAPSCX5
SLFR_G5R
SLFR_G3R
SLFR_G1R
CPOMSC54
RELSTM36
MS_24MR 0.000 0.000 -0.001 0.999

Specific indirect 3
WJAPSCX5
SLFR_G5R
SLFR_G3R
WJAPSC1S
PLSASC54
RVCSTM36
MS_24MR 0.000 0.000 1.441 0.150

Specific indirect 4
WJAPSCX5
SLFR_G5R
SLFR_G3R
WJAPSC1S
PLSASC54
RELSTM36
MS_24MR 0.000 0.000 1.184 0.236

Specific indirect 5
WJAPSCX5
SLFR_G5R
SLFR_G3R
WJAPSC1S
PLSESC54
RVCSTM36
MS_24MR 0.000 0.000 1.202 0.229

Specific indirect 6
WJAPSCX5
SLFR_G5R
SLFR_G3R
WJAPSC1S
PLSESC54
RELSTM36
MS_24MR 0.000 0.000 1.131 0.258

Specific indirect 7
WJAPSCX5
SLFR_G5R
SLFR_G3R
WJAPSC1S

CPOMSC54
RVCSTM36
MS_24MR 0.000 0.000 1.405 0.160

Specific indirect 8
WJAPSCX5
SLFR_G5R
SLFR_G3R
WJAPSC1S
CPOMSC54
RELSTM36
MS_24MR 0.000 0.000 -0.001 0.999

Specific indirect 9
WJAPSCX5
SLFR_G5R
SLFR_G3R
WJAPSC1S
WJMSSC54
RVCSTM36
MS_24MR 0.000 0.000 1.298 0.194

Specific indirect 10
WJAPSCX5
SLFR_G5R
SLFR_G3R
WJAPSC1S
WJMSSC54
RELSTM36
MS_24MR 0.000 0.000 1.039 0.299

Specific indirect 11
WJAPSCX5
SLFR_G5R
WJPCSCG3
WJAPSC1S
PLSASC54
RVCSTM36
MS_24MR 0.000 0.000 0.350 0.726

Specific indirect 12
WJAPSCX5
SLFR_G5R
WJPCSCG3
WJAPSC1S
PLSASC54
RELSTM36
MS_24MR 0.000 0.000 0.343 0.732

Specific indirect 13
WJAPSCX5
SLFR_G5R
WJPCSCG3
WJAPSC1S

PLSESC54
RVCSTM36
MS_24MR 0.000 0.000 0.341 0.733

Specific indirect 14
WJAPSCX5
SLFR_G5R
WJPCSCG3
WJAPSC1S
PLSESC54
RELSTM36
MS_24MR 0.000 0.000 0.336 0.737

Specific indirect 15
WJAPSCX5
SLFR_G5R
WJPCSCG3
WJAPSC1S
CPOMSC54
RVCSTM36
MS_24MR 0.000 0.000 0.348 0.728

Specific indirect 16
WJAPSCX5
SLFR_G5R
WJPCSCG3
WJAPSC1S
CPOMSC54
RELSTM36
MS_24MR 0.000 0.000 -0.001 0.999

Specific indirect 17
WJAPSCX5
SLFR_G5R
WJPCSCG3
WJAPSC1S
WJMSSC54
RVCSTM36
MS_24MR 0.000 0.000 0.347 0.728

Specific indirect 18
WJAPSCX5
SLFR_G5R
WJPCSCG3
WJAPSC1S
WJMSSC54
RELSTM36
MS_24MR 0.000 0.000 0.337 0.736

Specific indirect 19
WJAPSCX5
WJPCSCG5
WJPCSCG3
WJAPSC1S

PLSASC54
RVCSTM36
MS_24MR 0.000 0.000 -1.577 0.115

Specific indirect 20
WJAPSCX5
WJPCSCG5
WJPCSCG3
WJAPSC1S
PLSASC54
RELSTM36
MS_24MR 0.000 0.000 -1.239 0.215

Specific indirect 21
WJAPSCX5
WJPCSCG5
WJPCSCG3
WJAPSC1S
PLSESC54
RVCSTM36
MS_24MR 0.000 0.000 -1.247 0.212

Specific indirect 22
WJAPSCX5
WJPCSCG5
WJPCSCG3
WJAPSC1S
PLSESC54
RELSTM36
MS_24MR 0.000 0.000 -1.183 0.237

Specific indirect 23
WJAPSCX5
WJPCSCG5
WJPCSCG3
WJAPSC1S
CPOMSC54
RVCSTM36
MS_24MR 0.000 0.000 -1.439 0.150

Specific indirect 24
WJAPSCX5
WJPCSCG5
WJPCSCG3
WJAPSC1S
CPOMSC54
RELSTM36
MS_24MR 0.000 0.000 0.001 0.999

Specific indirect 25
WJAPSCX5
WJPCSCG5
WJPCSCG3
WJAPSC1S

WJMSSC54
RVCSTM36
MS_24MR 0.000 0.000 -1.313 0.189

Specific indirect 26
WJAPSCX5
WJPCSCG5
WJPCSCG3
WJAPSC1S
WJMSSC54
RELSTM36
MS_24MR 0.000 0.000 -1.021 0.307

Specific indirect 27
WJAPSCX5
WJPCSCG5
WJAPSCG3
WJAPSC1S
PLSASC54
RVCSTM36
MS_24MR 0.000 0.000 1.384 0.166

Specific indirect 28
WJAPSCX5
WJPCSCG5
WJAPSCG3
WJAPSC1S
PLSASC54
RELSTM36
MS_24MR 0.000 0.000 1.168 0.243

Specific indirect 29
WJAPSCX5
WJPCSCG5
WJAPSCG3
WJAPSC1S
PLSESC54
RVCSTM36
MS_24MR 0.000 0.000 1.160 0.246

Specific indirect 30
WJAPSCX5
WJPCSCG5
WJAPSCG3
WJAPSC1S
PLSESC54
RELSTM36
MS_24MR 0.000 0.000 1.110 0.267

Specific indirect 31
WJAPSCX5
WJPCSCG5
WJAPSCG3
WJAPSC1S

CPOMSC54
RVCSTM36
MS_24MR 0.000 0.000 1.388 0.165

Specific indirect 32
WJAPSCX5
WJPCSCG5
WJAPSCG3
WJAPSC1S
CPOMSC54
RELSTM36
MS_24MR 0.000 0.000 -0.001 0.999

Specific indirect 33
WJAPSCX5
WJPCSCG5
WJAPSCG3
WJAPSC1S
WJMSSC54
RVCSTM36
MS_24MR 0.000 0.000 1.260 0.208

Specific indirect 34
WJAPSCX5
WJPCSCG5
WJAPSCG3
WJAPSC1S
WJMSSC54
RELSTM36
MS_24MR 0.000 0.000 1.013 0.311

Specific indirect 35
WJAPSCX5
WJAPSCG5
WJPCSCG3
WJAPSC1S
PLSASC54
RVCSTM36
MS_24MR 0.000 0.000 0.316 0.752

Specific indirect 36
WJAPSCX5
WJAPSCG5
WJPCSCG3
WJAPSC1S
PLSASC54
RELSTM36
MS_24MR 0.000 0.000 0.312 0.755

Specific indirect 37
WJAPSCX5
WJAPSCG5
WJPCSCG3
WJAPSC1S

PLSESC54
RVCSTM36
MS_24MR 0.000 0.000 0.310 0.757

Specific indirect 38
WJAPSCX5
WJAPSCG5
WJPCSCG3
WJAPSC1S
PLSESC54
RELSTM36
MS_24MR 0.000 0.000 0.308 0.758

Specific indirect 39
WJAPSCX5
WJAPSCG5
WJPCSCG3
WJAPSC1S
CPOMSC54
RVCSTM36
MS_24MR 0.000 0.000 0.320 0.749

Specific indirect 40
WJAPSCX5
WJAPSCG5
WJPCSCG3
WJAPSC1S
CPOMSC54
RELSTM36
MS_24MR 0.000 0.000 -0.001 0.999

Specific indirect 41
WJAPSCX5
WJAPSCG5
WJPCSCG3
WJAPSC1S
WJMSSC54
RVCSTM36
MS_24MR 0.000 0.000 0.314 0.753

Specific indirect 42
WJAPSCX5
WJAPSCG5
WJPCSCG3
WJAPSC1S
WJMSSC54
RELSTM36
MS_24MR 0.000 0.000 0.311 0.756

Specific indirect 43
WJAPSCX5
WJAPSCG5
WJAPSCG3
WJAPSC1S

PLSASC54
RVCSTM36
MS_24MR 0.000 0.000 0.470 0.638

Specific indirect 44
WJAPSCX5
WJAPSCG5
WJAPSCG3
WJAPSC1S
PLSASC54
RELSTM36
MS_24MR 0.000 0.000 0.461 0.645

Specific indirect 45
WJAPSCX5
WJAPSCG5
WJAPSCG3
WJAPSC1S
PLSESC54
RVCSTM36
MS_24MR 0.000 0.000 0.465 0.642

Specific indirect 46
WJAPSCX5
WJAPSCG5
WJAPSCG3
WJAPSC1S
PLSESC54
RELSTM36
MS_24MR 0.000 0.000 0.462 0.644

Specific indirect 47
WJAPSCX5
WJAPSCG5
WJAPSCG3
WJAPSC1S
CPOMSC54
RVCSTM36
MS_24MR 0.000 0.000 0.484 0.629

Specific indirect 48
WJAPSCX5
WJAPSCG5
WJAPSCG3
WJAPSC1S
CPOMSC54
RELSTM36
MS_24MR 0.000 0.000 -0.001 0.999

Specific indirect 49
WJAPSCX5
WJAPSCG5
WJAPSCG3
WJAPSC1S

WJMSSC54
RVCSTM36
MS_24MR 0.000 0.000 0.480 0.631

Specific indirect 50
WJAPSCX5
WJAPSCG5
WJAPSCG3
WJAPSC1S
WJMSSC54
RELSTM36
MS_24MR 0.000 0.000 0.464 0.643

Effects from MS_54MR to WJAPSCX5

Total 0.000 0.000 0.000 1.000
Total indirect 0.000 0.000 0.000 1.000

Effects from PLSASC54 to WJAPSCX5

Total 0.004 0.004 0.935 0.350
Total indirect 0.004 0.004 0.935 0.350

Specific indirect 1
WJAPSCX5
SLFR_G5R
SLFR_G3R
WJAPSC1S
PLSASC54 0.001 0.000 1.538 0.124

Specific indirect 2
WJAPSCX5
SLFR_G5R
WJPCSCG3
WJAPSC1S
PLSASC54 0.000 0.000 0.352 0.725

Specific indirect 3
WJAPSCX5
WJPCSCG5
WJPCSCG3
WJAPSC1S
PLSASC54 -0.001 0.001 -1.709 0.088

Specific indirect 4
WJAPSCX5
WJPCSCG5
WJAPSCG3
WJAPSC1S
PLSASC54 0.003 0.002 1.471 0.141

Specific indirect 5

WJAPSCX5
WJAPSCG5
WJPCSCG3
WJAPSC1S
PLSASC54 0.000 0.000 0.317 0.751

Specific indirect 6
WJAPSCX5
WJAPSCG5
WJAPSCG3
WJAPSC1S
PLSASC54 0.001 0.002 0.474 0.635

Effects from PLSESC54 to WJAPSCX5

Total 0.002 0.002 0.865 0.387
Total indirect 0.002 0.002 0.865 0.387

Specific indirect 1
WJAPSCX5
SLFR_G5R
SLFR_G3R
WJAPSC1S
PLSESC54 0.000 0.000 1.250 0.211

Specific indirect 2
WJAPSCX5
SLFR_G5R
WJPCSCG3
WJAPSC1S
PLSESC54 0.000 0.000 0.342 0.733

Specific indirect 3
WJAPSCX5
WJPCSCG5
WJPCSCG3
WJAPSC1S
PLSESC54 0.000 0.000 -1.307 0.191

Specific indirect 4
WJAPSCX5
WJPCSCG5
WJAPSCG3
WJAPSC1S
PLSESC54 0.001 0.001 1.205 0.228

Specific indirect 5
WJAPSCX5
WJAPSCG5
WJPCSCG3
WJAPSC1S
PLSESC54 0.000 0.000 0.311 0.756

Specific indirect 6
WJAPSCX5
WJAPSCG5
WJAPSCG3
WJAPSC1S
PLSESC54 0.001 0.001 0.468 0.639

Effects from CPINCC54 to WJAPSCX5

Total	0.000	0.000	0.000	1.000
Total indirect	0.000	0.000	0.000	1.000

Effects from CPOMSC54 to WJAPSCX5

Total	-0.003	0.002	-1.496	0.135
Total indirect	-0.003	0.002	-1.496	0.135

Specific indirect 1
WJAPSCX5
SLFR_G5R
SLFR_G3R
SLFR_G1R
CPOMSC54 -0.001 0.001 -2.196 0.028

Specific indirect 2
WJAPSCX5
SLFR_G5R
SLFR_G3R
WJAPSC1S
CPOMSC54 0.000 0.000 -1.521 0.128

Specific indirect 3
WJAPSCX5
SLFR_G5R
WJPCSCG3
WJAPSC1S
CPOMSC54 0.000 0.000 -0.349 0.727

Specific indirect 4
WJAPSCX5
WJPCSCG5
WJPCSCG3
WJAPSC1S
CPOMSC54 0.001 0.000 1.573 0.116

Specific indirect 5
WJAPSCX5
WJPCSCG5
WJAPSCG3
WJAPSC1S
CPOMSC54 -0.002 0.001 -1.495 0.135

Specific indirect 6
WJAPSCX5
WJAPSCG5
WJPCSCG3
WJAPSC1S
CPOMSC54 0.000 0.000 -0.321 0.748

Specific indirect 7
WJAPSCX5
WJAPSCG5
WJAPSCG3
WJAPSC1S
CPOMSC54 -0.001 0.001 -0.487 0.626

Effects from WJMSSC54 to WJAPSCX5

Total 0.002 0.002 0.936 0.349
Total indirect 0.002 0.002 0.936 0.349

Specific indirect 1
WJAPSCX5
SLFR_G5R
SLFR_G3R
WJAPSC1S
WJMSSC54 0.000 0.000 1.376 0.169

Specific indirect 2
WJAPSCX5
SLFR_G5R
WJPCSCG3
WJAPSC1S
WJMSSC54 0.000 0.000 0.349 0.727

Specific indirect 3
WJAPSCX5
WJPCSCG5
WJPCSCG3
WJAPSC1S
WJMSSC54 0.000 0.000 -1.390 0.164

Specific indirect 4
WJAPSCX5
WJPCSCG5
WJAPSCG3
WJAPSC1S
WJMSSC54 0.001 0.001 1.331 0.183

Specific indirect 5
WJAPSCX5
WJAPSCG5
WJPCSCG3
WJAPSC1S
WJMSSC54 0.000 0.000 0.316 0.752

Specific indirect 6
WJAPSCX5
WJAPSCG5
WJAPSCG3
WJAPSC1S
WJMSSC54 0.000 0.001 0.485 0.628

Effects from RVCSTM36 to WJAPSCX5

Total 0.005 0.005 1.049 0.294
Total indirect 0.005 0.005 1.049 0.294

Specific indirect 1
WJAPSCX5
SLFR_G5R
SLFR_G3R
SLFR_G1R
CPOMSC54
RVCSTM36 0.000 0.000 2.083 0.037

Specific indirect 2
WJAPSCX5
SLFR_G5R
SLFR_G3R
WJAPSC1S
PLSASC54
RVCSTM36 0.000 0.000 1.532 0.126

Specific indirect 3
WJAPSCX5
SLFR_G5R
SLFR_G3R
WJAPSC1S
PLSESC54
RVCSTM36 0.000 0.000 1.242 0.214

Specific indirect 4
WJAPSCX5
SLFR_G5R
SLFR_G3R
WJAPSC1S
CPOMSC54
RVCSTM36 0.000 0.000 1.487 0.137

Specific indirect 5
WJAPSCX5
SLFR_G5R
SLFR_G3R
WJAPSC1S
WJMSSC54
RVCSTM36 0.000 0.000 1.357 0.175

Specific indirect 6
WJAPSCX5
SLFR_G5R
WJPCSCG3
WJAPSC1S
PLSASC54
RVCSTM36 0.000 0.000 0.352 0.725

Specific indirect 7
WJAPSCX5
SLFR_G5R
WJPCSCG3
WJAPSC1S
PLSESC54
RVCSTM36 0.000 0.000 0.342 0.733

Specific indirect 8
WJAPSCX5
SLFR_G5R
WJPCSCG3
WJAPSC1S
CPOMSC54
RVCSTM36 0.000 0.000 0.349 0.727

Specific indirect 9
WJAPSCX5
SLFR_G5R
WJPCSCG3
WJAPSC1S
WJMSSC54
RVCSTM36 0.000 0.000 0.349 0.727

Specific indirect 10
WJAPSCX5
WJPCSCG5
WJPCSCG3
WJAPSC1S
PLSASC54
RVCSTM36 -0.001 0.000 -1.705 0.088

Specific indirect 11
WJAPSCX5
WJPCSCG5
WJPCSCG3
WJAPSC1S
PLSESC54
RVCSTM36 0.000 0.000 -1.294 0.195

Specific indirect 12
WJAPSCX5
WJPCSCG5
WJPCSCG3
WJAPSC1S
CPOMSC54

RVCSTM36	0.000	0.000	-1.532	0.126

Specific indirect 13
WJAPSCX5
WJPCSCG5
WJPCSCG3
WJAPSC1S
WJMSSC54

RVCSTM36	0.000	0.000	-1.377	0.169

Specific indirect 14
WJAPSCX5
WJPCSCG5
WJAPSCG3
WJAPSC1S
PLSASC54

RVCSTM36	0.002	0.001	1.464	0.143

Specific indirect 15
WJAPSCX5
WJPCSCG5
WJAPSCG3
WJAPSC1S
PLSESC54

RVCSTM36	0.001	0.001	1.196	0.232

Specific indirect 16
WJAPSCX5
WJPCSCG5
WJAPSCG3
WJAPSC1S
CPOMSC54

RVCSTM36	0.001	0.000	1.468	0.142

Specific indirect 17
WJAPSCX5
WJPCSCG5
WJAPSCG3
WJAPSC1S
WJMSSC54

RVCSTM36	0.001	0.000	1.314	0.189

Specific indirect 18
WJAPSCX5
WJAPSCG5
WJPCSCG3
WJAPSC1S
PLSASC54

RVCSTM36	0.000	0.000	0.317	0.751

Specific indirect 19
WJAPSCX5
WJAPSCG5
WJPCSCG3

WJAPSC1S
PLSESC54
RVCSTM36 0.000 0.000 0.310 0.756

Specific indirect 20
WJAPSCX5
WJAPSCG5
WJPCSCG3
WJAPSC1S
CPOMSC54
RVCSTM36 0.000 0.000 0.321 0.748

Specific indirect 21
WJAPSCX5
WJAPSCG5
WJPCSCG3
WJAPSC1S
WJMSSC54
RVCSTM36 0.000 0.000 0.315 0.753

Specific indirect 22
WJAPSCX5
WJAPSCG5
WJAPSCG3
WJAPSC1S
PLSASC54
RVCSTM36 0.001 0.001 0.474 0.636

Specific indirect 23
WJAPSCX5
WJAPSCG5
WJAPSCG3
WJAPSC1S
PLSESC54
RVCSTM36 0.000 0.001 0.467 0.640

Specific indirect 24
WJAPSCX5
WJAPSCG5
WJAPSCG3
WJAPSC1S
CPOMSC54
RVCSTM36 0.000 0.000 0.487 0.626

Specific indirect 25
WJAPSCX5
WJAPSCG5
WJAPSCG3
WJAPSC1S
WJMSSC54
RVCSTM36 0.000 0.000 0.483 0.629

Effects from RELSTM36 to WJAPSCX5

Total 0.001 0.001 0.890 0.374
Total indirect 0.001 0.001 0.890 0.374

Specific indirect 1
WJAPSCX5
SLFR_G5R
SLFR_G3R
SLFR_G1R
CPOMSC54
RELSTM36 0.000 0.000 -0.001 0.999

Specific indirect 2
WJAPSCX5
SLFR_G5R
SLFR_G3R
WJAPSC1S
PLSASC54
RELSTM36 0.000 0.000 1.255 0.209

Specific indirect 3
WJAPSCX5
SLFR_G5R
SLFR_G3R
WJAPSC1S
PLSESC54
RELSTM36 0.000 0.000 1.195 0.232

Specific indirect 4
WJAPSCX5
SLFR_G5R
SLFR_G3R
WJAPSC1S
CPOMSC54
RELSTM36 0.000 0.000 -0.001 0.999

Specific indirect 5
WJAPSCX5
SLFR_G5R
SLFR_G3R
WJAPSC1S
WJMSSC54
RELSTM36 0.000 0.000 1.098 0.272

Specific indirect 6
WJAPSCX5
SLFR_G5R
WJPCSCG3
WJAPSC1S
PLSASC54
RELSTM36 0.000 0.000 0.346 0.730

Specific indirect 7
WJAPSCX5

SLFR_G5R
WJPCSCG3
WJAPSC1S
PLSESC54

RELSTM36	0.000	0.000	0.339	0.734

Specific indirect 8
WJAPSCX5
SLFR_G5R
WJPCSCG3
WJAPSC1S
CPOMSC54

RELSTM36	0.000	0.000	-0.001	0.999

Specific indirect 9
WJAPSCX5
SLFR_G5R
WJPCSCG3
WJAPSC1S
WJMSSC54

RELSTM36	0.000	0.000	0.340	0.734

Specific indirect 10
WJAPSCX5
WJPCSCG5
WJPCSCG3
WJAPSC1S
PLSASC54

RELSTM36	0.000	0.000	-1.327	0.185

Specific indirect 11
WJAPSCX5
WJPCSCG5
WJPCSCG3
WJAPSC1S
PLSESC54

RELSTM36	0.000	0.000	-1.261	0.207

Specific indirect 12
WJAPSCX5
WJPCSCG5
WJPCSCG3
WJAPSC1S
CPOMSC54

RELSTM36	0.000	0.000	0.001	0.999

Specific indirect 13
WJAPSCX5
WJPCSCG5
WJPCSCG3
WJAPSC1S
WJMSSC54

RELSTM36	0.000	0.000	-1.080	0.280

Specific indirect 14
WJAPSCX5
WJPCSCG5
WJAPSCG3
WJAPSC1S
PLSASC54
RELSTM36 0.000 0.000 1.230 0.219

Specific indirect 15
WJAPSCX5
WJPCSCG5
WJAPSCG3
WJAPSC1S
PLSESC54
RELSTM36 0.000 0.000 1.166 0.244

Specific indirect 16
WJAPSCX5
WJPCSCG5
WJAPSCG3
WJAPSC1S
CPOMSC54
RELSTM36 0.000 0.000 -0.001 0.999

Specific indirect 17
WJAPSCX5
WJPCSCG5
WJAPSCG3
WJAPSC1S
WJMSSC54
RELSTM36 0.000 0.000 1.064 0.287

Specific indirect 18
WJAPSCX5
WJAPSCG5
WJPCSCG3
WJAPSC1S
PLSASC54
RELSTM36 0.000 0.000 0.314 0.753

Specific indirect 19
WJAPSCX5
WJAPSCG5
WJPCSCG3
WJAPSC1S
PLSESC54
RELSTM36 0.000 0.000 0.310 0.757

Specific indirect 20
WJAPSCX5
WJAPSCG5
WJPCSCG3
WJAPSC1S
CPOMSC54

RELSTM36 0.000 0.000 -0.001 0.999

Specific indirect 21
WJAPSCX5
WJAPSCG5
WJPCSCG3
WJAPSC1S
WJMSSC54
RELSTM36 0.000 0.000 0.313 0.755

Specific indirect 22
WJAPSCX5
WJAPSCG5
WJAPSCG3
WJAPSC1S
PLSASC54
RELSTM36 0.000 0.000 0.464 0.642

Specific indirect 23
WJAPSCX5
WJAPSCG5
WJAPSCG3
WJAPSC1S
PLSESC54
RELSTM36 0.000 0.000 0.466 0.641

Specific indirect 24
WJAPSCX5
WJAPSCG5
WJAPSCG3
WJAPSC1S
CPOMSC54
RELSTM36 0.000 0.000 -0.001 0.999

Specific indirect 25
WJAPSCX5
WJAPSCG5
WJAPSCG3
WJAPSC1S
WJMSSC54
RELSTM36 0.000 0.000 0.468 0.639

Effects from MS_24MR to MS_G5R

Total 0.001 0.000 1.457 0.145
Total indirect 0.001 0.000 1.457 0.145

Specific indirect 1
MS_G5R
MS_G3R
MS_G1R
PLSASC54
RVCSTM36

MS_24MR 0.000 0.000 1.473 0.141

Specific indirect 2
MS_G5R
MS_G3R
MS_G1R
PLSASC54
RELSTM36
MS_24MR 0.000 0.000 1.192 0.233

Specific indirect 3
MS_G5R
MS_G3R
MS_G1R
MS_54MR
MS_36MR
MS_24MR 0.000 0.000 1.269 0.204

Effects from MS_54MR to MS_G5R

Total	0.005	0.003	1.526	0.127
Total indirect	0.005	0.003	1.526	0.127

Specific indirect 1
MS_G5R
MS_G3R
MS_G1R
MS_54MR 0.005 0.003 1.526 0.127

Effects from PLSASC54 to MS_G5R

Total	0.003	0.002	1.613	0.107
Total indirect	0.003	0.002	1.613	0.107

Specific indirect 1
MS_G5R
MS_G3R
MS_G1R
PLSASC54 0.003 0.002 1.613 0.107

Effects from PLSESC54 to MS_G5R

Total	0.000	0.000	0.000	1.000
Total indirect	0.000	0.000	0.000	1.000

Effects from CPINCC54 to MS_G5R

Total	0.000	0.000	0.000	1.000
Total indirect	0.000	0.000	0.000	1.000

Effects from CPOMSC54 to MS_G5R

```
  Total                0.000      0.000      0.000      1.000
  Total indirect       0.000      0.000      0.000      1.000
```

Effects from WJMSSC54 to MS_G5R

```
  Total                0.000      0.000      0.000      1.000
  Total indirect       0.000      0.000      0.000      1.000
```

Effects from RVCSTM36 to MS_G5R

```
  Total                0.002      0.001      1.615      0.106
  Total indirect       0.002      0.001      1.615      0.106

  Specific indirect 1
    MS_G5R
    MS_G3R
    MS_G1R
    PLSASC54
    RVCSTM36           0.002      0.001      1.615      0.106
```

Effects from RELSTM36 to MS_G5R

```
  Total                0.000      0.000      1.272      0.203
  Total indirect       0.000      0.000      1.272      0.203

  Specific indirect 1
    MS_G5R
    MS_G3R
    MS_G1R
    PLSASC54
    RELSTM36           0.000      0.000      1.272      0.203
```

Effects from MS_24MR to SLFR_G5R

```
  Total                0.001      0.001      2.221      0.026
  Total indirect       0.001      0.001      2.221      0.026

  Specific indirect 1
    SLFR_G5R
    SLFR_G3R
    SLFR_G1R
    CPOMSC54
    RVCSTM36
    MS_24MR            0.000      0.000      2.353      0.019

  Specific indirect 2
    SLFR_G5R
```

SLFR_G3R
SLFR_G1R
CPOMSC54
RELSTM36
MS_24MR 0.000 0.000 -0.001 0.999

Specific indirect 3
SLFR_G5R
SLFR_G3R
WJAPSC1S
PLSASC54
RVCSTM36
MS_24MR 0.000 0.000 1.691 0.091

Specific indirect 4
SLFR_G5R
SLFR_G3R
WJAPSC1S
PLSASC54
RELSTM36
MS_24MR 0.000 0.000 1.310 0.190

Specific indirect 5
SLFR_G5R
SLFR_G3R
WJAPSC1S
PLSESC54
RVCSTM36
MS_24MR 0.000 0.000 1.364 0.173

Specific indirect 6
SLFR_G5R
SLFR_G3R
WJAPSC1S
PLSESC54
RELSTM36
MS_24MR 0.000 0.000 1.267 0.205

Specific indirect 7
SLFR_G5R
SLFR_G3R
WJAPSC1S
CPOMSC54
RVCSTM36
MS_24MR 0.000 0.000 1.658 0.097

Specific indirect 8
SLFR_G5R
SLFR_G3R
WJAPSC1S
CPOMSC54
RELSTM36
MS_24MR 0.000 0.000 -0.001 0.999

Specific indirect 9
SLFR_G5R
SLFR_G3R
WJAPSC1S
WJMSSC54
RVCSTM36
MS_24MR 0.000 0.000 1.475 0.140

Specific indirect 10
SLFR_G5R
SLFR_G3R
WJAPSC1S
WJMSSC54
RELSTM36
MS_24MR 0.000 0.000 1.119 0.263

Specific indirect 11
SLFR_G5R
WJPCSCG3
WJAPSC1S
PLSASC54
RVCSTM36
MS_24MR 0.000 0.000 0.360 0.719

Specific indirect 12
SLFR_G5R
WJPCSCG3
WJAPSC1S
PLSASC54
RELSTM36
MS_24MR 0.000 0.000 0.352 0.725

Specific indirect 13
SLFR_G5R
WJPCSCG3
WJAPSC1S
PLSESC54
RVCSTM36
MS_24MR 0.000 0.000 0.350 0.726

Specific indirect 14
SLFR_G5R
WJPCSCG3
WJAPSC1S
PLSESC54
RELSTM36
MS_24MR 0.000 0.000 0.346 0.730

Specific indirect 15
SLFR_G5R
WJPCSCG3
WJAPSC1S
CPOMSC54
RVCSTM36

MS_24MR 0.000 0.000 0.358 0.721

Specific indirect 16
SLFR_G5R
WJPCSCG3
WJAPSC1S
CPOMSC54
RELSTM36
MS_24MR 0.000 0.000 -0.001 0.999

Specific indirect 17
SLFR_G5R
WJPCSCG3
WJAPSC1S
WJMSSC54
RVCSTM36
MS_24MR 0.000 0.000 0.357 0.721

Specific indirect 18
SLFR_G5R
WJPCSCG3
WJAPSC1S
WJMSSC54
RELSTM36
MS_24MR 0.000 0.000 0.346 0.730

Effects from MS_54MR to SLFR_G5R

Total 0.000 0.000 0.000 1.000
Total indirect 0.000 0.000 0.000 1.000

Effects from PLSASC54 to SLFR_G5R

Total 0.005 0.003 1.795 0.073
Total indirect 0.005 0.003 1.795 0.073

Specific indirect 1
SLFR_G5R
SLFR_G3R
WJAPSC1S
PLSASC54 0.005 0.002 1.840 0.066

Specific indirect 2
SLFR_G5R
WJPCSCG3
WJAPSC1S
PLSASC54 0.000 0.001 0.362 0.718

Effects from PLSESC54 to SLFR_G5R

Total 0.002 0.002 1.382 0.167

Total indirect 0.002 0.002 1.382 0.167

Specific indirect 1
SLFR_G5R
SLFR_G3R
WJAPSC1S
PLSESC54 0.002 0.002 1.431 0.153

Specific indirect 2
SLFR_G5R
WJPCSCG3
WJAPSC1S
PLSESC54 0.000 0.001 0.351 0.725

Effects from CPINCC54 to SLFR_G5R

Total 0.000 0.000 0.000 1.000
Total indirect 0.000 0.000 0.000 1.000

Effects from CPOMSC54 to SLFR_G5R

Total -0.012 0.004 -3.348 0.001
Total indirect -0.012 0.004 -3.348 0.001

Specific indirect 1
SLFR_G5R
SLFR_G3R
SLFR_G1R
CPOMSC54 -0.009 0.003 -2.977 0.003

Specific indirect 2
SLFR_G5R
SLFR_G3R
WJAPSC1S
CPOMSC54 -0.003 0.001 -1.843 0.065

Specific indirect 3
SLFR_G5R
WJPCSCG3
WJAPSC1S
CPOMSC54 0.000 0.001 -0.359 0.720

Effects from WJMSSC54 to SLFR_G5R

Total 0.002 0.001 1.551 0.121
Total indirect 0.002 0.001 1.551 0.121

Specific indirect 1
SLFR_G5R
SLFR_G3R
WJAPSC1S

```
    WJMSSC54          0.002      0.001      1.583      0.113

  Specific indirect 2
    SLFR_G5R
    WJPCSCG3
    WJAPSC1S
    WJMSSC54          0.000      0.000      0.359      0.720


Effects from RVCSTM36 to SLFR_G5R

  Total                0.009      0.003      2.594      0.009
  Total indirect       0.009      0.003      2.594      0.009

  Specific indirect 1
    SLFR_G5R
    SLFR_G3R
    SLFR_G1R
    CPOMSC54
    RVCSTM36          0.003      0.001      2.718      0.007

  Specific indirect 2
    SLFR_G5R
    SLFR_G3R
    WJAPSC1S
    PLSASC54
    RVCSTM36          0.003      0.001      1.829      0.067

  Specific indirect 3
    SLFR_G5R
    SLFR_G3R
    WJAPSC1S
    PLSESC54
    RVCSTM36          0.001      0.001      1.417      0.156

  Specific indirect 4
    SLFR_G5R
    SLFR_G3R
    WJAPSC1S
    CPOMSC54
    RVCSTM36          0.001      0.000      1.786      0.074

  Specific indirect 5
    SLFR_G5R
    SLFR_G3R
    WJAPSC1S
    WJMSSC54
    RVCSTM36          0.001      0.001      1.554      0.120

  Specific indirect 6
    SLFR_G5R
    WJPCSCG3
    WJAPSC1S
    PLSASC54
```

RVCSTM36 0.000 0.001 0.362 0.717

Specific indirect 7
SLFR_G5R
WJPCSCG3
WJAPSC1S
PLSESC54
RVCSTM36 0.000 0.000 0.351 0.725

Specific indirect 8
SLFR_G5R
WJPCSCG3
WJAPSC1S
CPOMSC54
RVCSTM36 0.000 0.000 0.359 0.720

Specific indirect 9
SLFR_G5R
WJPCSCG3
WJAPSC1S
WJMSSC54
RVCSTM36 0.000 0.000 0.358 0.720

Effects from RELSTM36 to SLFR_G5R

Total 0.001 0.001 1.115 0.265
Total indirect 0.001 0.001 1.115 0.265

Specific indirect 1
SLFR_G5R
SLFR_G3R
SLFR_G1R
CPOMSC54
RELSTM36 0.000 0.000 -0.001 0.999

Specific indirect 2
SLFR_G5R
SLFR_G3R
WJAPSC1S
PLSASC54
RELSTM36 0.000 0.000 1.403 0.161

Specific indirect 3
SLFR_G5R
SLFR_G3R
WJAPSC1S
PLSESC54
RELSTM36 0.000 0.000 1.353 0.176

Specific indirect 4
SLFR_G5R
SLFR_G3R
WJAPSC1S

CPOMSC54
RELSTM36 0.000 0.000 -0.001 0.999

Specific indirect 5
SLFR_G5R
SLFR_G3R
WJAPSC1S
WJMSSC54
RELSTM36 0.000 0.000 1.190 0.234

Specific indirect 6
SLFR_G5R
WJPCSCG3
WJAPSC1S
PLSASC54
RELSTM36 0.000 0.000 0.355 0.723

Specific indirect 7
SLFR_G5R
WJPCSCG3
WJAPSC1S
PLSESC54
RELSTM36 0.000 0.000 0.349 0.727

Specific indirect 8
SLFR_G5R
WJPCSCG3
WJAPSC1S
CPOMSC54
RELSTM36 0.000 0.000 -0.001 0.999

Specific indirect 9
SLFR_G5R
WJPCSCG3
WJAPSC1S
WJMSSC54
RELSTM36 0.000 0.000 0.349 0.727

Effects from MS_24MR to WJPCSCG5

Total -0.001 0.001 -1.200 0.230
Total indirect -0.001 0.001 -1.200 0.230

Specific indirect 1
WJPCSCG5
WJPCSCG3
WJAPSC1S
PLSASC54
RVCSTM36
MS_24MR 0.000 0.000 1.660 0.097

Specific indirect 2
WJPCSCG5

WJPCSCG3
WJAPSC1S
PLSASC54
RELSTM36
MS_24MR 0.000 0.000 1.274 0.203

Specific indirect 3
WJPCSCG5
WJPCSCG3
WJAPSC1S
PLSESC54
RVCSTM36
MS_24MR 0.000 0.000 1.315 0.188

Specific indirect 4
WJPCSCG5
WJPCSCG3
WJAPSC1S
PLSESC54
RELSTM36
MS_24MR 0.000 0.000 1.245 0.213

Specific indirect 5
WJPCSCG5
WJPCSCG3
WJAPSC1S
CPOMSC54
RVCSTM36
MS_24MR 0.000 0.000 1.475 0.140

Specific indirect 6
WJPCSCG5
WJPCSCG3
WJAPSC1S
CPOMSC54
RELSTM36
MS_24MR 0.000 0.000 -0.001 0.999

Specific indirect 7
WJPCSCG5
WJPCSCG3
WJAPSC1S
WJMSSC54
RVCSTM36
MS_24MR 0.000 0.000 1.385 0.166

Specific indirect 8
WJPCSCG5
WJPCSCG3
WJAPSC1S
WJMSSC54
RELSTM36
MS_24MR 0.000 0.000 1.057 0.291

Specific indirect 9
WJPCSCG5
WJAPSCG3
WJAPSC1S
PLSASC54
RVCSTM36

MS_24MR	-0.001	0.000	-1.691	0.091

Specific indirect 10
WJPCSCG5
WJAPSCG3
WJAPSC1S
PLSASC54
RELSTM36

MS_24MR	0.000	0.000	-1.330	0.184

Specific indirect 11
WJPCSCG5
WJAPSCG3
WJAPSC1S
PLSESC54
RVCSTM36

MS_24MR	0.000	0.000	-1.354	0.176

Specific indirect 12
WJPCSCG5
WJAPSCG3
WJAPSC1S
PLSESC54
RELSTM36

MS_24MR	0.000	0.000	-1.281	0.200

Specific indirect 13
WJPCSCG5
WJAPSCG3
WJAPSC1S
CPOMSC54
RVCSTM36

MS_24MR	0.000	0.000	-1.661	0.097

Specific indirect 14
WJPCSCG5
WJAPSCG3
WJAPSC1S
CPOMSC54
RELSTM36

MS_24MR	0.000	0.000	0.001	0.999

Specific indirect 15
WJPCSCG5
WJAPSCG3
WJAPSC1S
WJMSSC54
RVCSTM36

MS_24MR 0.000 0.000 -1.510 0.131

Specific indirect 16
WJPCSCG5
WJAPSCG3
WJAPSC1S
WJMSSC54
RELSTM36
MS_24MR 0.000 0.000 -1.135 0.256

Effects from MS_54MR to WJPCSCG5

Total	0.000	0.000	0.000	1.000
Total indirect	0.000	0.000	0.000	1.000

Effects from PLSASC54 to WJPCSCG5

Total	-0.006	0.005	-1.190	0.234
Total indirect	-0.006	0.005	-1.190	0.234

Specific indirect 1
WJPCSCG5
WJPCSCG3
WJAPSC1S
PLSASC54 0.003 0.002 1.811 0.070

Specific indirect 2
WJPCSCG5
WJAPSCG3
WJAPSC1S
PLSASC54 -0.009 0.005 -1.853 0.064

Effects from PLSESC54 to WJPCSCG5

Total	-0.003	0.003	-1.055	0.291
Total indirect	-0.003	0.003	-1.055	0.291

Specific indirect 1
WJPCSCG5
WJPCSCG3
WJAPSC1S
PLSESC54 0.001 0.001 1.386 0.166

Specific indirect 2
WJPCSCG5
WJAPSCG3
WJAPSC1S
PLSESC54 -0.004 0.003 -1.427 0.154

Effects from CPINCC54 to WJPCSCG5

```
  Total               0.000      0.000      0.000      1.000
  Total indirect      0.000      0.000      0.000      1.000


Effects from CPOMSC54 to WJPCSCG5

  Total               0.003      0.003      1.228      0.220
  Total indirect      0.003      0.003      1.228      0.220

  Specific indirect 1
    WJPCSCG5
    WJPCSCG3
    WJAPSC1S
    CPOMSC54         -0.002      0.001     -1.627      0.104

  Specific indirect 2
    WJPCSCG5
    WJAPSCG3
    WJAPSC1S
    CPOMSC54          0.005      0.003      1.864      0.062


Effects from WJMSSC54 to WJPCSCG5

  Total              -0.003      0.002     -1.153      0.249
  Total indirect     -0.003      0.002     -1.153      0.249

  Specific indirect 1
    WJPCSCG5
    WJPCSCG3
    WJAPSC1S
    WJMSSC54          0.001      0.001      1.473      0.141

  Specific indirect 2
    WJPCSCG5
    WJAPSCG3
    WJAPSC1S
    WJMSSC54         -0.004      0.002     -1.633      0.102


Effects from RVCSTM36 to WJPCSCG5

  Total              -0.007      0.006     -1.243      0.214
  Total indirect     -0.007      0.006     -1.243      0.214

  Specific indirect 1
    WJPCSCG5
    WJPCSCG3
    WJAPSC1S
    PLSASC54
    RVCSTM36          0.002      0.001      1.809      0.070

  Specific indirect 2
```

WJPCSCG5
WJPCSCG3
WJAPSC1S
PLSESC54
RVCSTM36 0.001 0.001 1.371 0.170

Specific indirect 3
WJPCSCG5
WJPCSCG3
WJAPSC1S
CPOMSC54
RVCSTM36 0.001 0.000 1.574 0.115

Specific indirect 4
WJPCSCG5
WJPCSCG3
WJAPSC1S
WJMSSC54
RVCSTM36 0.001 0.000 1.459 0.145

Specific indirect 5
WJPCSCG5
WJAPSCG3
WJAPSC1S
PLSASC54
RVCSTM36 -0.005 0.003 -1.842 0.066

Specific indirect 6
WJPCSCG5
WJAPSCG3
WJAPSC1S
PLSESC54
RVCSTM36 -0.002 0.002 -1.412 0.158

Specific indirect 7
WJPCSCG5
WJAPSCG3
WJAPSC1S
CPOMSC54
RVCSTM36 -0.002 0.001 -1.801 0.072

Specific indirect 8
WJPCSCG5
WJAPSCG3
WJAPSC1S
WJMSSC54
RVCSTM36 -0.002 0.001 -1.604 0.109

Effects from RELSTM36 to WJPCSCG5

Total -0.001 0.001 -1.129 0.259
Total indirect -0.001 0.001 -1.129 0.259

Specific indirect 1
WJPCSCG5
WJPCSCG3
WJAPSC1S
PLSASC54
RELSTM36 0.000 0.000 1.367 0.172

Specific indirect 2
WJPCSCG5
WJPCSCG3
WJAPSC1S
PLSESC54
RELSTM36 0.000 0.000 1.335 0.182

Specific indirect 3
WJPCSCG5
WJPCSCG3
WJAPSC1S
CPOMSC54
RELSTM36 0.000 0.000 -0.001 0.999

Specific indirect 4
WJPCSCG5
WJPCSCG3
WJAPSC1S
WJMSSC54
RELSTM36 0.000 0.000 1.120 0.263

Specific indirect 5
WJPCSCG5
WJAPSCG3
WJAPSC1S
PLSASC54
RELSTM36 -0.001 0.000 -1.422 0.155

Specific indirect 6
WJPCSCG5
WJAPSCG3
WJAPSC1S
PLSESC54
RELSTM36 -0.001 0.000 -1.367 0.172

Specific indirect 7
WJPCSCG5
WJAPSCG3
WJAPSC1S
CPOMSC54
RELSTM36 0.000 0.000 0.001 0.999

Specific indirect 8
WJPCSCG5
WJAPSCG3
WJAPSC1S
WJMSSC54

RELSTM36 0.000 0.000 -1.206 0.228

Effects from MS_24MR to WJAPSCG5

Total 0.003 0.002 2.053 0.040
Total indirect 0.003 0.002 2.053 0.040

Specific indirect 1
WJAPSCG5
WJPCSCG3
WJAPSC1S
PLSASC54
RVCSTM36
MS_24MR 0.000 0.000 0.423 0.672

Specific indirect 2
WJAPSCG5
WJPCSCG3
WJAPSC1S
PLSASC54
RELSTM36
MS_24MR 0.000 0.000 0.413 0.679

Specific indirect 3
WJAPSCG5
WJPCSCG3
WJAPSC1S
PLSESC54
RVCSTM36
MS_24MR 0.000 0.000 0.404 0.686

Specific indirect 4
WJAPSCG5
WJPCSCG3
WJAPSC1S
PLSESC54
RELSTM36
MS_24MR 0.000 0.000 0.400 0.689

Specific indirect 5
WJAPSCG5
WJPCSCG3
WJAPSC1S
CPOMSC54
RVCSTM36
MS_24MR 0.000 0.000 0.423 0.672

Specific indirect 6
WJAPSCG5
WJPCSCG3
WJAPSC1S
CPOMSC54
RELSTM36

MS_24MR 0.000 0.000 -0.001 0.999

Specific indirect 7
WJAPSCG5
WJPCSCG3
WJAPSC1S
WJMSSC54
RVCSTM36
MS_24MR 0.000 0.000 0.409 0.683

Specific indirect 8
WJAPSCG5
WJPCSCG3
WJAPSC1S
WJMSSC54
RELSTM36
MS_24MR 0.000 0.000 0.399 0.690

Specific indirect 9
WJAPSCG5
WJAPSCG3
WJAPSC1S
PLSASC54
RVCSTM36
MS_24MR 0.001 0.001 1.933 0.053

Specific indirect 10
WJAPSCG5
WJAPSCG3
WJAPSC1S
PLSASC54
RELSTM36
MS_24MR 0.000 0.000 1.434 0.152

Specific indirect 11
WJAPSCG5
WJAPSCG3
WJAPSC1S
PLSESC54
RVCSTM36
MS_24MR 0.001 0.000 1.434 0.152

Specific indirect 12
WJAPSCG5
WJAPSCG3
WJAPSC1S
PLSESC54
RELSTM36
MS_24MR 0.000 0.000 1.340 0.180

Specific indirect 13
WJAPSCG5
WJAPSCG3
WJAPSC1S

CPOMSC54
RVCSTM36
MS_24MR 0.000 0.000 1.904 0.057

Specific indirect 14
WJAPSCG5
WJAPSCG3
WJAPSC1S
CPOMSC54
RELSTM36
MS_24MR 0.000 0.000 -0.001 0.999

Specific indirect 15
WJAPSCG5
WJAPSCG3
WJAPSC1S
WJMSSC54
RVCSTM36
MS_24MR 0.000 0.000 1.656 0.098

Specific indirect 16
WJAPSCG5
WJAPSCG3
WJAPSC1S
WJMSSC54
RELSTM36
MS_24MR 0.000 0.000 1.187 0.235

Effects from MS_54MR to WJAPSCG5

Total 0.000 0.000 0.000 1.000
Total indirect 0.000 0.000 0.000 1.000

Effects from PLSASC54 to WJAPSCG5

Total 0.018 0.008 2.095 0.036
Total indirect 0.018 0.008 2.095 0.036

Specific indirect 1
WJAPSCG5
WJPCSCG3
WJAPSC1S
PLSASC54 0.001 0.002 0.425 0.671

Specific indirect 2
WJAPSCG5
WJAPSCG3
WJAPSC1S
PLSASC54 0.017 0.008 2.158 0.031

Effects from PLSESC54 to WJAPSCG5

Total 0.009 0.006 1.468 0.142
Total indirect 0.009 0.006 1.468 0.142

Specific indirect 1
WJAPSCG5
WJPCSCG3
WJAPSC1S
PLSESC54 0.000 0.001 0.406 0.685

Specific indirect 2
WJAPSCG5
WJAPSCG3
WJAPSC1S
PLSESC54 0.009 0.006 1.513 0.130

Effects from CPINCC54 to WJAPSCG5

Total 0.000 0.000 0.000 1.000
Total indirect 0.000 0.000 0.000 1.000

Effects from CPOMSC54 to WJAPSCG5

Total -0.011 0.005 -2.120 0.034
Total indirect -0.011 0.005 -2.120 0.034

Specific indirect 1
WJAPSCG5
WJPCSCG3
WJAPSC1S
CPOMSC54 0.000 0.001 -0.425 0.671

Specific indirect 2
WJAPSCG5
WJAPSCG3
WJAPSC1S
CPOMSC54 -0.010 0.005 -2.184 0.029

Effects from WJMSSC54 to WJAPSCG5

Total 0.008 0.005 1.738 0.082
Total indirect 0.008 0.005 1.738 0.082

Specific indirect 1
WJAPSCG5
WJPCSCG3
WJAPSC1S
WJMSSC54 0.000 0.001 0.411 0.681

Specific indirect 2
WJAPSCG5

WJAPSCG3
WJAPSC1S
WJMSSC54 0.008 0.004 1.807 0.071

Effects from RVCSTM36 to WJAPSCG5

Total 0.022 0.010 2.279 0.023
Total indirect 0.022 0.010 2.279 0.023

Specific indirect 1
WJAPSCG5
WJPCSCG3
WJAPSC1S
PLSASC54
RVCSTM36 0.000 0.001 0.425 0.671

Specific indirect 2
WJAPSCG5
WJPCSCG3
WJAPSC1S
PLSESC54
RVCSTM36 0.000 0.000 0.405 0.685

Specific indirect 3
WJAPSCG5
WJPCSCG3
WJAPSC1S
CPOMSC54
RVCSTM36 0.000 0.000 0.425 0.671

Specific indirect 4
WJAPSCG5
WJPCSCG3
WJAPSC1S
WJMSSC54
RVCSTM36 0.000 0.000 0.410 0.682

Specific indirect 5
WJAPSCG5
WJAPSCG3
WJAPSC1S
PLSASC54
RVCSTM36 0.010 0.005 2.136 0.033

Specific indirect 6
WJAPSCG5
WJAPSCG3
WJAPSC1S
PLSESC54
RVCSTM36 0.004 0.003 1.492 0.136

Specific indirect 7
WJAPSCG5

WJAPSCG3
WJAPSC1S
CPOMSC54
RVCSTM36 0.003 0.001 2.092 0.036

Specific indirect 8
WJAPSCG5
WJAPSCG3
WJAPSC1S
WJMSSC54
RVCSTM36 0.004 0.002 1.764 0.078

Effects from RELSTM36 to WJAPSCG5

Total 0.003 0.002 1.712 0.087
Total indirect 0.003 0.002 1.712 0.087

Specific indirect 1
WJAPSCG5
WJPCSCG3
WJAPSC1S
PLSASC54
RELSTM36 0.000 0.000 0.417 0.677

Specific indirect 2
WJAPSCG5
WJPCSCG3
WJAPSC1S
PLSESC54
RELSTM36 0.000 0.000 0.404 0.686

Specific indirect 3
WJAPSCG5
WJPCSCG3
WJAPSC1S
CPOMSC54
RELSTM36 0.000 0.000 -0.001 0.999

Specific indirect 4
WJAPSCG5
WJPCSCG3
WJAPSC1S
WJMSSC54
RELSTM36 0.000 0.000 0.403 0.687

Specific indirect 5
WJAPSCG5
WJAPSCG3
WJAPSC1S
PLSASC54
RELSTM36 0.001 0.001 1.548 0.122

Specific indirect 6

WJAPSCG5
WJAPSCG3
WJAPSC1S
PLSESC54
RELSTM36 0.001 0.001 1.436 0.151

Specific indirect 7
WJAPSCG5
WJAPSCG3
WJAPSC1S
CPOMSC54
RELSTM36 0.000 0.000 -0.001 0.999

Specific indirect 8
WJAPSCG5
WJAPSCG3
WJAPSC1S
WJMSSC54
RELSTM36 0.000 0.000 1.268 0.205

Effects from MS_24MR to MS_G3R

Total 0.005 0.002 2.191 0.028
Total indirect 0.005 0.002 2.191 0.028

Specific indirect 1
MS_G3R
MS_G1R
PLSASC54
RVCSTM36
MS_24MR 0.002 0.001 2.180 0.029

Specific indirect 2
MS_G3R
MS_G1R
PLSASC54
RELSTM36
MS_24MR 0.000 0.000 1.517 0.129

Specific indirect 3
MS_G3R
MS_G1R
MS_54MR
MS_36MR
MS_24MR 0.003 0.002 1.693 0.090

Effects from MS_54MR to MS_G3R

Total 0.041 0.017 2.377 0.017
Total indirect 0.041 0.017 2.377 0.017

Specific indirect 1

MS_G3R
MS_G1R
MS_54MR 0.041 0.017 2.377 0.017

Effects from PLSASC54 to MS_G3R

Total 0.027 0.010 2.631 0.009
Total indirect 0.027 0.010 2.631 0.009

Specific indirect 1
MS_G3R
MS_G1R
PLSASC54 0.027 0.010 2.631 0.009

Effects from PLSESC54 to MS_G3R

Total 0.000 0.000 0.000 1.000
Total indirect 0.000 0.000 0.000 1.000

Effects from CPINCC54 to MS_G3R

Total 0.000 0.000 0.000 1.000
Total indirect 0.000 0.000 0.000 1.000

Effects from CPOMSC54 to MS_G3R

Total 0.000 0.000 0.000 1.000
Total indirect 0.000 0.000 0.000 1.000

Effects from WJMSSC54 to MS_G3R

Total 0.000 0.000 0.000 1.000
Total indirect 0.000 0.000 0.000 1.000

Effects from RVCSTM36 to MS_G3R

Total 0.016 0.006 2.620 0.009
Total indirect 0.016 0.006 2.620 0.009

Specific indirect 1
MS_G3R
MS_G1R
PLSASC54
RVCSTM36 0.016 0.006 2.620 0.009

Effects from RELSTM36 to MS_G3R

Total 0.002 0.001 1.685 0.092
Total indirect 0.002 0.001 1.685 0.092

Specific indirect 1
MS_G3R
MS_G1R
PLSASC54
RELSTM36 0.002 0.001 1.685 0.092

Effects from MS_24MR to SLFR_G3R

Total 0.004 0.002 2.466 0.014
Total indirect 0.004 0.002 2.466 0.014

Specific indirect 1
SLFR_G3R
SLFR_G1R
CPOMSC54
RVCSTM36
MS_24MR 0.001 0.001 2.572 0.010

Specific indirect 2
SLFR_G3R
SLFR_G1R
CPOMSC54
RELSTM36
MS_24MR 0.000 0.000 -0.001 0.999

Specific indirect 3
SLFR_G3R
WJAPSC1S
PLSASC54
RVCSTM36
MS_24MR 0.001 0.001 1.746 0.081

Specific indirect 4
SLFR_G3R
WJAPSC1S
PLSASC54
RELSTM36
MS_24MR 0.000 0.000 1.336 0.181

Specific indirect 5
SLFR_G3R
WJAPSC1S
PLSESC54
RVCSTM36
MS_24MR 0.001 0.000 1.395 0.163

Specific indirect 6
SLFR_G3R
WJAPSC1S
PLSESC54

RELSTM36
MS_24MR 0.000 0.000 1.287 0.198

Specific indirect 7
SLFR_G3R
WJAPSC1S
CPOMSC54
RVCSTM36
MS_24MR 0.000 0.000 1.713 0.087

Specific indirect 8
SLFR_G3R
WJAPSC1S
CPOMSC54
RELSTM36
MS_24MR 0.000 0.000 -0.001 0.999

Specific indirect 9
SLFR_G3R
WJAPSC1S
WJMSSC54
RVCSTM36
MS_24MR 0.000 0.000 1.517 0.129

Specific indirect 10
SLFR_G3R
WJAPSC1S
WJMSSC54
RELSTM36
MS_24MR 0.000 0.000 1.137 0.256

Effects from MS_54MR to SLFR_G3R

Total	0.000	0.000	0.000	1.000
Total indirect	0.000	0.000	0.000	1.000

Effects from PLSASC54 to SLFR_G3R

Total	0.017	0.009	1.913	0.056
Total indirect	0.017	0.009	1.913	0.056

Specific indirect 1
SLFR_G3R
WJAPSC1S
PLSASC54 0.017 0.009 1.913 0.056

Effects from PLSESC54 to SLFR_G3R

Total	0.009	0.006	1.466	0.143
Total indirect	0.009	0.006	1.466	0.143

Specific indirect 1
SLFR_G3R
WJAPSC1S
PLSESC54 0.009 0.006 1.466 0.143

Effects from CPINCC54 to SLFR_G3R

Total 0.000 0.000 0.000 1.000
Total indirect 0.000 0.000 0.000 1.000

Effects from CPOMSC54 to SLFR_G3R

Total -0.044 0.011 -4.014 0.000
Total indirect -0.044 0.011 -4.014 0.000

Specific indirect 1
SLFR_G3R
SLFR_G1R
CPOMSC54 -0.033 0.010 -3.466 0.001

Specific indirect 2
SLFR_G3R
WJAPSC1S
CPOMSC54 -0.010 0.005 -1.920 0.055

Effects from WJMSSC54 to SLFR_G3R

Total 0.008 0.005 1.635 0.102
Total indirect 0.008 0.005 1.635 0.102

Specific indirect 1
SLFR_G3R
WJAPSC1S
WJMSSC54 0.008 0.005 1.635 0.102

Effects from RVCSTM36 to SLFR_G3R

Total 0.032 0.011 2.982 0.003
Total indirect 0.032 0.011 2.982 0.003

Specific indirect 1
SLFR_G3R
SLFR_G1R
CPOMSC54
RVCSTM36 0.010 0.003 3.078 0.002

Specific indirect 2
SLFR_G3R
WJAPSC1S
PLSASC54

RVCSTM36 0.010 0.005 1.902 0.057

Specific indirect 3
SLFR_G3R
WJAPSC1S
PLSESC54
RVCSTM36 0.004 0.003 1.452 0.146

Specific indirect 4
SLFR_G3R
WJAPSC1S
CPOMSC54
RVCSTM36 0.003 0.002 1.855 0.064

Specific indirect 5
SLFR_G3R
WJAPSC1S
WJMSSC54
RVCSTM36 0.004 0.002 1.605 0.109

Effects from RELSTM36 to SLFR_G3R

Total 0.003 0.002 1.127 0.260
Total indirect 0.003 0.002 1.127 0.260

Specific indirect 1
SLFR_G3R
SLFR_G1R
CPOMSC54
RELSTM36 0.000 0.001 -0.001 0.999

Specific indirect 2
SLFR_G3R
WJAPSC1S
PLSASC54
RELSTM36 0.001 0.001 1.438 0.150

Specific indirect 3
SLFR_G3R
WJAPSC1S
PLSESC54
RELSTM36 0.001 0.001 1.381 0.167

Specific indirect 4
SLFR_G3R
WJAPSC1S
CPOMSC54
RELSTM36 0.000 0.000 -0.001 0.999

Specific indirect 5
SLFR_G3R
WJAPSC1S
WJMSSC54

RELSTM36 0.000 0.000 1.213 0.225

Effects from MS_24MR to WJPCSCG3

Total 0.004 0.002 1.914 0.056
Total indirect 0.004 0.002 1.914 0.056

Specific indirect 1
WJPCSCG3
WJAPSC1S
PLSASC54
RVCSTM36
MS_24MR 0.002 0.001 1.834 0.067

Specific indirect 2
WJPCSCG3
WJAPSC1S
PLSASC54
RELSTM36
MS_24MR 0.000 0.000 1.368 0.171

Specific indirect 3
WJPCSCG3
WJAPSC1S
PLSESC54
RVCSTM36
MS_24MR 0.001 0.001 1.354 0.176

Specific indirect 4
WJPCSCG3
WJAPSC1S
PLSESC54
RELSTM36
MS_24MR 0.000 0.000 1.267 0.205

Specific indirect 5
WJPCSCG3
WJAPSC1S
CPOMSC54
RVCSTM36
MS_24MR 0.001 0.000 1.700 0.089

Specific indirect 6
WJPCSCG3
WJAPSC1S
CPOMSC54
RELSTM36
MS_24MR 0.000 0.000 -0.001 0.999

Specific indirect 7
WJPCSCG3
WJAPSC1S
WJMSSC54

RVCSTM36
MS_24MR 0.001 0.000 1.472 0.141

Specific indirect 8
WJPCSCG3
WJAPSC1S
WJMSSC54
RELSTM36
MS_24MR 0.000 0.000 1.112 0.266

Effects from MS_54MR to WJPCSCG3

Total	0.000	0.000	0.000	1.000
Total indirect	0.000	0.000	0.000	1.000

Effects from PLSASC54 to WJPCSCG3

Total	0.025	0.012	2.032	0.042
Total indirect	0.025	0.012	2.032	0.042

Specific indirect 1
WJPCSCG3
WJAPSC1S
PLSASC54 0.025 0.012 2.032 0.042

Effects from PLSESC54 to WJPCSCG3

Total	0.012	0.009	1.424	0.154
Total indirect	0.012	0.009	1.424	0.154

Specific indirect 1
WJPCSCG3
WJAPSC1S
PLSESC54 0.012 0.009 1.424 0.154

Effects from CPINCC54 to WJPCSCG3

Total	0.000	0.000	0.000	1.000
Total indirect	0.000	0.000	0.000	1.000

Effects from CPOMSC54 to WJPCSCG3

Total	-0.015	0.008	-1.918	0.055
Total indirect	-0.015	0.008	-1.918	0.055

Specific indirect 1
WJPCSCG3
WJAPSC1S
CPOMSC54 -0.015 0.008 -1.918 0.055

Effects from WJMSSC54 to WJPCSCG3

```
  Total                0.012      0.007      1.580      0.114
  Total indirect       0.012      0.007      1.580      0.114

  Specific indirect 1
    WJPCSCG3
    WJAPSC1S
    WJMSSC54           0.012      0.007      1.580      0.114
```

Effects from RVCSTM36 to WJPCSCG3

```
  Total                0.031      0.015      2.115      0.034
  Total indirect       0.031      0.015      2.115      0.034

  Specific indirect 1
    WJPCSCG3
    WJAPSC1S
    PLSASC54
    RVCSTM36           0.015      0.007      2.021      0.043

  Specific indirect 2
    WJPCSCG3
    WJAPSC1S
    PLSESC54
    RVCSTM36           0.006      0.004      1.408      0.159

  Specific indirect 3
    WJPCSCG3
    WJAPSC1S
    CPOMSC54
    RVCSTM36           0.005      0.002      1.843      0.065

  Specific indirect 4
    WJPCSCG3
    WJAPSC1S
    WJMSSC54
    RVCSTM36           0.005      0.003      1.554      0.120
```

Effects from RELSTM36 to WJPCSCG3

```
  Total                0.004      0.002      1.630      0.103
  Total indirect       0.004      0.002      1.630      0.103

  Specific indirect 1
    WJPCSCG3
    WJAPSC1S
    PLSASC54
    RELSTM36           0.001      0.001      1.483      0.138
```

Specific indirect 2
WJPCSCG3
WJAPSC1S
PLSESC54
RELSTM36 0.002 0.001 1.360 0.174

Specific indirect 3
WJPCSCG3
WJAPSC1S
CPOMSC54
RELSTM36 0.000 0.001 -0.001 0.999

Specific indirect 4
WJPCSCG3
WJAPSC1S
WJMSSC54
RELSTM36 0.001 0.001 1.186 0.236

Effects from MS_24MR to WJAPSCG3

Total 0.011 0.003 3.253 0.001
Total indirect 0.011 0.003 3.253 0.001

Specific indirect 1
WJAPSCG3
WJAPSC1S
PLSASC54
RVCSTM36
MS_24MR 0.005 0.002 2.676 0.007

Specific indirect 2
WJAPSCG3
WJAPSC1S
PLSASC54
RELSTM36
MS_24MR 0.000 0.000 1.669 0.095

Specific indirect 3
WJAPSCG3
WJAPSC1S
PLSESC54
RVCSTM36
MS_24MR 0.002 0.001 1.702 0.089

Specific indirect 4
WJAPSCG3
WJAPSC1S
PLSESC54
RELSTM36
MS_24MR 0.000 0.000 1.547 0.122

Specific indirect 5
WJAPSCG3

WJAPSC1S
CPOMSC54
RVCSTM36
MS_24MR 0.001 0.001 2.408 0.016

Specific indirect 6
WJAPSCG3
WJAPSC1S
CPOMSC54
RELSTM36
MS_24MR 0.000 0.000 -0.001 0.999

Specific indirect 7
WJAPSCG3
WJAPSC1S
WJMSSC54
RVCSTM36
MS_24MR 0.002 0.001 1.962 0.050

Specific indirect 8
WJAPSCG3
WJAPSC1S
WJMSSC54
RELSTM36
MS_24MR 0.000 0.000 1.289 0.197

Effects from MS_54MR to WJAPSCG3

Total 0.000 0.000 0.000 1.000
Total indirect 0.000 0.000 0.000 1.000

Effects from PLSASC54 to WJAPSCG3

Total 0.060 0.018 3.379 0.001
Total indirect 0.060 0.018 3.379 0.001

Specific indirect 1
WJAPSCG3
WJAPSC1S
PLSASC54 0.060 0.018 3.379 0.001

Effects from PLSESC54 to WJAPSCG3

Total 0.030 0.017 1.833 0.067
Total indirect 0.030 0.017 1.833 0.067

Specific indirect 1
WJAPSCG3
WJAPSC1S
PLSESC54 0.030 0.017 1.833 0.067

Effects from CPINCC54 to WJAPSCG3

```
  Total                0.000      0.000      0.000      1.000
  Total indirect       0.000      0.000      0.000      1.000
```

Effects from CPOMSC54 to WJAPSCG3

```
  Total               -0.036      0.011     -3.134      0.002
  Total indirect      -0.036      0.011     -3.134      0.002

  Specific indirect 1
    WJAPSCG3
    WJAPSC1S
    CPOMSC54          -0.036      0.011     -3.134      0.002
```

Effects from WJMSSC54 to WJAPSCG3

```
  Total                0.028      0.013      2.216      0.027
  Total indirect       0.028      0.013      2.216      0.027

  Specific indirect 1
    WJAPSCG3
    WJAPSC1S
    WJMSSC54           0.028      0.013      2.216      0.027
```

Effects from RVCSTM36 to WJAPSCG3

```
  Total                0.075      0.017      4.401      0.000
  Total indirect       0.075      0.017      4.401      0.000

  Specific indirect 1
    WJAPSCG3
    WJAPSC1S
    PLSASC54
    RVCSTM36           0.036      0.011      3.310      0.001

  Specific indirect 2
    WJAPSCG3
    WJAPSC1S
    PLSESC54
    RVCSTM36           0.015      0.008      1.800      0.072

  Specific indirect 3
    WJAPSCG3
    WJAPSC1S
    CPOMSC54
    RVCSTM36           0.011      0.004      2.826      0.005

  Specific indirect 4
    WJAPSCG3
```

WJAPSC1S
WJMSSC54
RVCSTM36 0.013 0.006 2.148 0.032

Effects from RELSTM36 to WJAPSCG3

Total 0.009 0.004 2.332 0.020
Total indirect 0.009 0.004 2.332 0.020

Specific indirect 1
WJAPSCG3
WJAPSC1S
PLSASC54
RELSTM36 0.004 0.002 1.862 0.063

Specific indirect 2
WJAPSCG3
WJAPSC1S
PLSESC54
RELSTM36 0.004 0.002 1.704 0.088

Specific indirect 3
WJAPSCG3
WJAPSC1S
CPOMSC54
RELSTM36 0.000 0.001 -0.001 0.999

Specific indirect 4
WJAPSCG3
WJAPSC1S
WJMSSC54
RELSTM36 0.002 0.001 1.396 0.163

Effects from MS_24MR to MS_G1R

Total 0.030 0.009 3.190 0.001
Total indirect 0.030 0.009 3.190 0.001

Specific indirect 1
MS_G1R
PLSASC54
RVCSTM36
MS_24MR 0.012 0.004 2.916 0.004

Specific indirect 2
MS_G1R
PLSASC54
RELSTM36
MS_24MR 0.001 0.001 1.689 0.091

Specific indirect 3
MS_G1R

MS_54MR
MS_36MR
MS_24MR 0.016 0.008 2.111 0.035

Effects from MS_54MR to MS_G1R

Total	0.237	0.060	3.920	0.000
Total indirect	0.000	0.000	0.000	1.000

Direct
MS_G1R
MS_54MR 0.237 0.060 3.920 0.000

Effects from PLSASC54 to MS_G1R

Total	0.159	0.040	4.011	0.000
Total indirect	0.000	0.000	0.000	1.000

Direct
MS_G1R
PLSASC54 0.159 0.040 4.011 0.000

Effects from PLSESC54 to MS_G1R

Total	0.000	0.000	0.000	1.000
Total indirect	0.000	0.000	0.000	1.000

Effects from CPINCC54 to MS_G1R

Total	0.000	0.000	0.000	1.000
Total indirect	0.000	0.000	0.000	1.000

Effects from CPOMSC54 to MS_G1R

Total	0.000	0.000	0.000	1.000
Total indirect	0.000	0.000	0.000	1.000

Effects from WJMSSC54 to MS_G1R

Total	0.000	0.000	0.000	1.000
Total indirect	0.000	0.000	0.000	1.000

Effects from RVCSTM36 to MS_G1R

Total	0.094	0.024	3.973	0.000
Total indirect	0.094	0.024	3.973	0.000

Specific indirect 1
MS_G1R
PLSASC54
RVCSTM36 0.094 0.024 3.973 0.000

Effects from RELSTM36 to MS_G1R

Total 0.009 0.005 1.903 0.057
Total indirect 0.009 0.005 1.903 0.057

Specific indirect 1
MS_G1R
PLSASC54
RELSTM36 0.009 0.005 1.903 0.057

Effects from MS_24MR to SLFR_G1R

Total 0.007 0.002 3.111 0.002
Total indirect 0.007 0.002 3.111 0.002

Specific indirect 1
SLFR_G1R
CPOMSC54
RVCSTM36
MS_24MR 0.007 0.002 3.044 0.002

Specific indirect 2
SLFR_G1R
CPOMSC54
RELSTM36
MS_24MR 0.000 0.001 -0.001 0.999

Effects from MS_54MR to SLFR_G1R

Total 0.000 0.000 0.000 1.000
Total indirect 0.000 0.000 0.000 1.000

Effects from PLSASC54 to SLFR_G1R

Total 0.000 0.000 0.000 1.000
Total indirect 0.000 0.000 0.000 1.000

Effects from PLSESC54 to SLFR_G1R

Total 0.000 0.000 0.000 1.000
Total indirect 0.000 0.000 0.000 1.000

Effects from CPINCC54 to SLFR_G1R

Total	0.000	0.000	0.000	1.000
Total indirect	0.000	0.000	0.000	1.000

Effects from CPOMSC54 to SLFR_G1R

Total	-0.171	0.034	-4.951	0.000
Total indirect	0.000	0.000	0.000	1.000

Direct
SLFR_G1R

CPOMSC54	-0.171	0.034	-4.951	0.000

Effects from WJMSSC54 to SLFR_G1R

Total	0.000	0.000	0.000	1.000
Total indirect	0.000	0.000	0.000	1.000

Effects from RVCSTM36 to SLFR_G1R

Total	0.052	0.013	4.002	0.000
Total indirect	0.052	0.013	4.002	0.000

Specific indirect 1
SLFR_G1R
CPOMSC54

RVCSTM36	0.052	0.013	4.002	0.000

Effects from RELSTM36 to SLFR_G1R

Total	0.000	0.006	-0.001	0.999
Total indirect	0.000	0.006	-0.001	0.999

Specific indirect 1
SLFR_G1R
CPOMSC54

RELSTM36	0.000	0.006	-0.001	0.999

Effects from MS_24MR to WJAPSC1S

Total	0.036	0.008	4.422	0.000
Total indirect	0.036	0.008	4.422	0.000

Specific indirect 1
WJAPSC1S
PLSASC54
RVCSTM36

MS_24MR	0.015	0.005	3.118	0.002

Specific indirect 2
WJAPSC1S
PLSASC54
RELSTM36
MS_24MR 0.001 0.001 1.757 0.079

Specific indirect 3
WJAPSC1S
PLSESC54
RVCSTM36
MS_24MR 0.007 0.003 1.913 0.056

Specific indirect 4
WJAPSC1S
PLSESC54
RELSTM36
MS_24MR 0.001 0.001 1.696 0.090

Specific indirect 5
WJAPSC1S
CPOMSC54
RVCSTM36
MS_24MR 0.005 0.002 2.611 0.009

Specific indirect 6
WJAPSC1S
CPOMSC54
RELSTM36
MS_24MR 0.000 0.000 -0.001 0.999

Specific indirect 7
WJAPSC1S
WJMSSC54
RVCSTM36
MS_24MR 0.006 0.003 2.121 0.034

Specific indirect 8
WJAPSC1S
WJMSSC54
RELSTM36
MS_24MR 0.001 0.000 1.330 0.184

Effects from MS_54MR to WJAPSC1S

Total 0.000 0.000 0.000 1.000
Total indirect 0.000 0.000 0.000 1.000

Effects from PLSASC54 to WJAPSC1S

Total 0.204 0.046 4.411 0.000
Total indirect 0.000 0.000 0.000 1.000

Direct
WJAPSC1S
PLSASC54 0.204 0.046 4.411 0.000

Effects from PLSESC54 to WJAPSC1S

Total 0.102 0.049 2.098 0.036
Total indirect 0.000 0.000 0.000 1.000

Direct
WJAPSC1S
PLSESC54 0.102 0.049 2.098 0.036

Effects from CPINCC54 to WJAPSC1S

Total 0.000 0.000 0.000 1.000
Total indirect 0.000 0.000 0.000 1.000

Effects from CPOMSC54 to WJAPSC1S

Total -0.121 0.033 -3.676 0.000
Total indirect 0.000 0.000 0.000 1.000

Direct
WJAPSC1S
CPOMSC54 -0.121 0.033 -3.676 0.000

Effects from WJMSSC54 to WJAPSC1S

Total 0.095 0.039 2.441 0.015
Total indirect 0.000 0.000 0.000 1.000

Direct
WJAPSC1S
WJMSSC54 0.095 0.039 2.441 0.015

Effects from RVCSTM36 to WJAPSC1S

Total 0.253 0.024 10.574 0.000
Total indirect 0.253 0.024 10.574 0.000

Specific indirect 1
WJAPSC1S
PLSASC54
RVCSTM36 0.121 0.028 4.291 0.000

Specific indirect 2
WJAPSC1S
PLSESC54

RVCSTM36 0.052 0.025 2.058 0.040

Specific indirect 3
WJAPSC1S
CPOMSC54
RVCSTM36 0.037 0.012 3.174 0.002

Specific indirect 4
WJAPSC1S
WJMSSC54
RVCSTM36 0.043 0.018 2.364 0.018

Effects from RELSTM36 to WJAPSC1S

Total 0.031 0.011 2.772 0.006
Total indirect 0.031 0.011 2.772 0.006

Specific indirect 1
WJAPSC1S
PLSASC54
RELSTM36 0.012 0.006 1.993 0.046

Specific indirect 2
WJAPSC1S
PLSESC54
RELSTM36 0.013 0.007 1.915 0.056

Specific indirect 3
WJAPSC1S
CPOMSC54
RELSTM36 0.000 0.005 -0.001 0.999

Specific indirect 4
WJAPSC1S
WJMSSC54
RELSTM36 0.006 0.004 1.451 0.147

Effects from MS_24MR to MS_54MR

Total 0.070 0.022 3.148 0.002
Total indirect 0.070 0.022 3.148 0.002

Specific indirect 1
MS_54MR
MS_36MR
MS_24MR 0.070 0.022 3.148 0.002

Effects from MS_24MR to PLSASC54

Total 0.082 0.017 4.803 0.000
Total indirect 0.082 0.017 4.803 0.000

Specific indirect 1
PLSASC54
RVCSTM36
MS_24MR 0.076 0.016 4.670 0.000

Specific indirect 2
PLSASC54
RELSTM36
MS_24MR 0.006 0.003 1.910 0.056

Effects from MS_24MR to PLSESC54

Total 0.079 0.016 4.791 0.000
Total indirect 0.079 0.016 4.791 0.000

Specific indirect 1
PLSESC54
RVCSTM36
MS_24MR 0.065 0.014 4.558 0.000

Specific indirect 2
PLSESC54
RELSTM36
MS_24MR 0.014 0.005 2.749 0.006

Effects from MS_24MR to CPINCC54

Total -0.023 0.008 -2.890 0.004
Total indirect -0.023 0.008 -2.890 0.004

Specific indirect 1
CPINCC54
RVCSTM36
MS_24MR -0.031 0.008 -3.722 0.000

Specific indirect 2
CPINCC54
RELSTM36
MS_24MR 0.008 0.005 1.727 0.084

Effects from MS_24MR to CPOMSC54

Total -0.039 0.010 -3.945 0.000
Total indirect -0.039 0.010 -3.945 0.000

Specific indirect 1
CPOMSC54
RVCSTM36
MS_24MR -0.039 0.010 -3.853 0.000

Specific indirect 2
CPOMSC54
RELSTM36
MS_24MR 0.000 0.004 0.001 0.999

Effects from MS_24MR to WJMSSC54

Total	0.065	0.014	4.685	0.000
Total indirect	0.065	0.014	4.685	0.000

Specific indirect 1
WJMSSC54
RVCSTM36
MS_24MR 0.058 0.013 4.501 0.000

Specific indirect 2
WJMSSC54
RELSTM36
MS_24MR 0.006 0.004 1.581 0.114

MODEL MODIFICATION INDICES

NOTE: Modification indices for direct effects of observed dependent variables regressed on covariates may not be included. To include these, request MODINDICES (ALL).

Minimum M.I. value for printing the modification index 25.000

	M.I.	E.P.C.	Std E.P.C.	StdYX E.P.C.
**BY Statements**				
MS_15M BY INTRUSIV	31.825	0.225	0.231	0.183
MS_36M BY POSREGAR	37.904	0.218	0.221	0.188
MS_36M BY INTRUSIV	26.022	0.241	0.244	0.194
MS_36M BY SLFRLCX5	26.735	-0.094	-0.095	-0.232
MS_G5 BY MWRSIOX5	32.748	0.599	0.574	0.190
MS_RI BY POSREGAR	30.174	0.345	0.233	0.199
MS_RI BY INTRUSIV	30.978	0.430	0.290	0.231
MS_RI BY MWRSIOX5	29.316	0.920	0.622	0.206
MS_RI BY SLFRLCX5	49.216	-0.273	-0.184	-0.450
MS_6MR BY NONDISTR	55.771	0.705	0.754	0.594
MS_6MR BY POSREGAR	30.174	-0.345	-0.369	-0.314
MS_15MR BY NONDISTR	27.972	0.650	0.503	0.422
MS_24MR BY NONDISTR	40.229	0.648	0.596	0.466
MS_24MR BY INTRUSIV	30.978	-0.430	-0.396	-0.314
MS_15R BY MWRSIOX5	29.316	-0.920	-0.880	-0.291
SR BY SLFRLCX5	60.502	-1.143	-0.266	-0.648
READ BY SLFRLCX5	34.585	-0.013	-0.151	-0.368
READ BY WJPCSCX5	40.976	0.265	3.135	0.213

WJPCSCG3 BY WJAPSC1S	41.569	-0.637	-5.090	-0.299
WJPCSCG3 BY WJAPSCG3	67.034	0.398	3.184	0.214
WJPCSCG3 BY WJPCSCG5	41.079	-0.874	-6.986	-0.532
WJPCSCG5 BY WJAPSCG3	58.070	1.570	9.045	0.608
WJPCSCX5 BY WJAPSCX5	54.075	0.556	4.890	0.353
WJPCSCX5 BY WJPCSCG5	28.706	0.639	5.619	0.428
MATH BY SLFRLCX5	42.001	-0.015	-0.170	-0.415
MATH BY WJPCSCX5	39.090	0.249	2.811	0.191
WJAPSC1S BY WJAPSCG3	30.723	-0.662	-7.568	-0.509
WJAPSCG3 BY WJAPSC1S	26.437	-0.524	-4.876	-0.286
WJAPSCG3 BY WJPCSCG3	67.173	0.318	2.958	0.206
WJAPSCG5 BY WJPCSCG3	41.608	1.032	7.413	0.516
WJAPSCX5 BY WJPCSCG5	38.799	0.795	6.364	0.485
WJAPSCX5 BY WJPCSCX5	54.076	0.673	5.390	0.366

ON/BY Statements

RVCSTM36 ON MS_24M /				
MS_24M BY RVCSTM36	32.675	5.478	6.255	0.394
RVCSTM36 ON MS_36M /				
MS_36M BY RVCSTM36	32.675	5.478	5.544	0.350
RVCSTM36 ON MS_RI /				
MS_RI BY RVCSTM36	32.675	5.478	3.701	0.233
RVCSTM36 ON SR /				
SR BY RVCSTM36	67.992	22.730	5.283	0.333
RVCSTM36 ON READ /				
READ BY RVCSTM36	70.463	0.315	3.720	0.235
RVCSTM36 ON MATH /				
MATH BY RVCSTM36	84.725	0.374	4.215	0.266
PLSASC54 ON READ /				
READ BY PLSASC54	26.928	0.221	2.607	0.131
PLSASC54 ON MATH /				
MATH BY PLSASC54	30.226	0.256	2.881	0.144
MS_15 ON MS_15M /				
MS_15M BY MS_15	26.191	-0.199	-0.175	-0.175
MS_RI ON MS_G1 /				
MS_G1 BY MS_RI	27.537	0.167	0.275	0.275
MS_RI ON MS_G1R /				
MS_G1R BY MS_RI	27.536	0.167	0.208	0.208
MS_15R ON MS_15M /				
MS_15M BY MS_15R	26.191	-0.199	-0.214	-0.214
SLFR_G3R ON MS_RI /				
MS_RI BY SLFR_G3R	25.157	0.251	0.186	0.186
SLFR_G3R ON SR /				
SR BY SLFR_G3R	26.422	0.918	0.234	0.234
SLFR_G3R ON READ /				
READ BY SLFR_G3R	34.224	0.016	0.208	0.208
SLFR_G3R ON MATH /				
MATH BY SLFR_G3R	31.166	0.017	0.206	0.206
SLFR_15R ON MS_36M /				
MS_36M BY SLFR_15R	26.735	-0.094	-0.281	-0.281
SLFR_15R ON MS_RI /				
MS_RI BY SLFR_15R	49.216	-0.273	-0.546	-0.546
SLFR_15R ON SR /				

SR BY SLFR_15R 60.502 -1.143 -0.786 -0.786
SLFR_15R ON READ /
READ BY SLFR_15R 34.585 -0.013 -0.446 -0.446
SLFR_15R ON MATH /
MATH BY SLFR_15R 42.001 -0.015 -0.503 -0.503
READ ON WJPCSCG3 /
WJPCSCG3 BY READ 32.213 -0.559 -0.378 -0.378
WJPCSCG3 ON WJAPSCG3 /
WJAPSCG3 BY WJPCSCG3 67.035 0.318 0.370 0.370
WJPCSCG3 ON WJAPSCG5 /
WJAPSCG5 BY WJPCSCG3 37.608 1.057 0.950 0.950
WJPCSCG5 ON MS_RI /
MS_RI BY WJPCSCG5 26.817 -1.973 -0.231 -0.231
WJPCSCG5 ON READ /
READ BY WJPCSCG5 38.589 -0.182 -0.373 -0.373
WJPCSCG5 ON MATH /
MATH BY WJPCSCG5 34.100 -0.159 -0.311 -0.311
WJPCSCX5 ON READ /
READ BY WJPCSCX5 40.976 0.265 0.357 0.357
WJPCSCX5 ON MATH /
MATH BY WJPCSCX5 39.090 0.249 0.320 0.320
WJPCSCX5 ON WJAPSCX5 /
WJAPSCX5 BY WJPCSCX5 54.076 0.673 0.613 0.613
MATH ON WJAPSC1S /
WJAPSC1S BY MATH 37.352 0.277 0.281 0.281
WJAPSCG3 ON WJPCSCG3 /
WJPCSCG3 BY WJAPSCG3 67.034 0.398 0.342 0.342
WJAPSCG3 ON WJPCSCG5 /
WJPCSCG5 BY WJAPSCG3 37.207 1.361 0.843 0.843
WJAPSCG5 ON WJPCSCX5 /
WJPCSCX5 BY WJAPSCG5 31.759 0.585 0.716 0.716
WJAPSCG5 ON WJAPSCX5 /
WJAPSCX5 BY WJAPSCG5 27.591 0.673 0.750 0.750
WJAPSCX5 ON WJPCSCX5 /
WJPCSCX5 BY WJAPSCX5 54.075 0.556 0.611 0.611

ON Statements

SLFR_15R ON MEDUCM01 28.345 -0.051 -0.150 -0.376
READ ON RELSTM36 28.573 0.107 0.009 0.132
READ ON PLSASC54 28.861 0.088 0.007 0.149
READ ON PLSESC54 31.021 0.090 0.008 0.152
READ ON WJMSSC54 45.516 0.109 0.009 0.171
WJPCSCX5 ON PLSESC54 27.034 0.093 0.011 0.212
MATH ON PLSASC54 30.607 0.093 0.008 0.164

WITH Statements

INTRUSIV WITH NONDISTR 32.149 0.544 0.544 4.784
INTRUSIV WITH POSREGAR 58.686 -0.299 -0.299 -0.302
POSREGAR WITH POSREGAR 27.610 0.150 0.150 0.160
INTRUSIV WITH INTRUSIV 51.932 0.223 0.223 0.226
INTRUSIV WITH POSREGAR 31.051 -0.200 -0.200 -0.212
POSREGAR WITH POSREGAR 34.466 0.172 0.172 0.193

POSREGAR WITH NONDISTR	41.643	0.407	0.407	0.773
INTRUSIV WITH INTRUSIV	49.740	0.233	0.233	0.235
INTRUSIV WITH POSREGAR	49.955	-0.266	-0.266	-0.285
SPTSIO54 WITH INTRUSIV	30.909	-0.156	-0.156	-0.224
SPTSIO1S WITH SPTSIO54	29.053	0.134	0.134	0.255
HOSTILIT WITH HOSTILIT	31.496	0.086	0.086	0.204
HOSTILIT WITH HOSTILIT	46.212	0.105	0.105	0.255
HOSTILIT WITH HOSTILIT	26.058	0.084	0.084	0.187
MHISIOX5 WITH MRASIOX5	34.169	-1.137	-1.137	-2.133
WJAPSCG3 WITH WJAPSC1S	40.153	-41.332	-41.332	999.000
WJAPSCX5 WITH SLFR_OG5	25.995	-4.499	-4.499	999.000
WJPCSCG3 WITH WJAPSC1S	35.899	-35.511	-35.511	999.000
WJPCSCG3 WITH WJAPSCG3	68.376	25.301	25.301	999.000
WJPCSCG5 WITH WJAPSCX5	45.300	51.974	51.974	999.000
WJPCSCX5 WITH WJAPSCX5	54.076	37.592	37.592	999.000
WJPCSCX5 WITH WJPCSCG5	26.875	52.660	52.660	999.000
WJPCSCG3 WITH READ	36.029	-41.830	-0.534	-0.534
WJAPSCG3 WITH WJPCSCG3	67.034	25.078	0.356	0.356
WJAPSCG3 WITH MATH	26.866	-19.305	-0.232	-0.232
WJAPSCX5 WITH WJPCSCX5	54.076	37.592	0.612	0.612

Variances/Residual Variances

WJAPSC1S	41.435	192.105	192.105	0.662
WJAPSCG3	39.720	236.022	236.022	1.066
WJPCSCG5	39.603	121.436	121.436	0.705

SAMPLE STATISTICS FOR ESTIMATED FACTOR SCORES

SAMPLE STATISTICS

Means

MS_6M	MS_6M_SE	MS_15M	MS_15M_S	MS_24M
3.545	0.181	3.545	0.476	3.545

Means

MS_24M_S	MS_36M	MS_36M_S	MS_54M	MS_54M_S
0.519	3.545	0.464	3.545	0.506

Means

MS_G1	MS_G1_SE	MS_G3	MS_G3_SE	MS_G5
4.195	0.537	3.642	0.453	3.556

Means

MS_G5_SE MS_15 MS_15_SE MS_RI MS_RI_SE

0.412	3.547	0.546	3.545	0.301

Means

MS_6MR	MS_6MR_S	MS_15MR	MS_15MR_	MS_24MR
0.000	0.361	0.000	0.501	0.000

Means

MS_24MR_	MS_36MR	MS_36MR_	MS_54MR	MS_54MR_
0.552	0.000	0.495	0.000	0.541

Means

MS_G1R	MS_G1R_S	MS_G3R	MS_G3R_S	MS_G5R
0.650	0.561	0.097	0.484	0.011

Means

MS_G5R_S	MS_15R	MS_15R_S	SR	SR_SE
0.451	0.002	0.585	0.767	0.152

Means

SLFR_G1R	SLFR_G1R	SLFR_G3R	SLFR_G3R	SLFR_G5R
-0.226	0.410	0.102	0.361	0.028

Means

SLFR_G5R	SLFR_15R	SLFR_15R	READ	READ_SE
0.354	-0.008	0.199	54.315	4.774

Means

WJPCSCG3	WJPCSCG3	WJPCSCG5	WJPCSCG5	WJPCSCX5
1.768	4.626	-0.291	3.947	0.099

Means

WJPCSCX5	MATH	MATH_SE	WJAPSC1S	WJAPSC1S
5.130	42.052	4.950	20.808	5.639

Means

WJAPSCG3 WJAPSCG3 WJAPSCG5 WJAPSCG5 WJAPSCX5

5.012	5.238	1.139	4.676	0.244

Means

WJAPSCX5
5.140

Covariances

	MS_6M	MS_6M_SE	MS_15M	MS_15M_S	MS_24M
MS_6M	1.513				
MS_6M_SE	-0.018	0.068			
MS_15M	0.528	-0.022	0.823		
MS_15M_S	-0.008	0.021	-0.009	0.017	
MS_24M	0.472	-0.025	0.488	-0.010	1.054
MS_24M_S	-0.012	0.025	-0.014	0.018	-0.013
MS_36M	0.535	-0.022	0.501	-0.013	0.637
MS_36M_S	-0.009	0.020	-0.016	0.015	-0.011
MS_54M	0.492	-0.026	0.468	-0.010	0.573
MS_54M_S	-0.016	0.022	-0.018	0.017	-0.016
MS_G1	0.522	-0.022	0.522	-0.011	0.589
MS_G1_SE	-0.021	0.023	-0.022	0.015	-0.024
MS_G3	0.448	-0.017	0.442	-0.010	0.452
MS_G3_SE	-0.024	0.019	-0.019	0.013	-0.024
MS_G5	0.403	-0.017	0.399	-0.010	0.424
MS_G5_SE	-0.037	0.019	-0.024	0.013	-0.027
MS_15	0.358	-0.013	0.341	-0.007	0.385
MS_15_SE	-0.037	0.023	-0.037	0.016	-0.043
MS_RI	0.451	-0.017	0.423	-0.009	0.448
MS_RI_SE	-0.006	0.009	-0.006	0.006	-0.006
MS_6MR	1.062	-0.001	0.105	0.002	0.024
MS_6MR_S	-0.017	0.050	-0.019	0.017	-0.022
MS_15MR	0.077	-0.004	0.400	0.000	0.040
MS_15MR_	-0.006	0.014	-0.007	0.011	-0.007
MS_24MR	0.021	-0.007	0.065	0.000	0.606
MS_24MR_	-0.010	0.018	-0.011	0.013	-0.011
MS_36MR	0.084	-0.005	0.078	-0.003	0.189
MS_36MR_	-0.007	0.012	-0.011	0.009	-0.008
MS_54MR	0.041	-0.009	0.045	-0.001	0.125
MS_54MR_	-0.012	0.015	-0.013	0.011	-0.012
MS_G1R	0.071	-0.004	0.099	-0.001	0.141
MS_G1R_S	-0.016	0.015	-0.017	0.010	-0.018
MS_G3R	-0.003	0.000	0.019	-0.001	0.004
MS_G3R_S	-0.016	0.011	-0.013	0.008	-0.016
MS_G5R	-0.048	0.001	-0.023	0.000	-0.024
MS_G5R_S	-0.024	0.011	-0.015	0.007	-0.017
MS_15R	-0.093	0.005	-0.081	0.003	-0.063
MS_15R_S	-0.029	0.017	-0.029	0.011	-0.034
SR	0.073	-0.002	0.074	-0.002	0.083
SR_SE	-0.002	0.002	-0.002	0.002	-0.002
SLFR_G1R	0.087	-0.001	0.071	0.000	0.076

SLFR_G1R	-0.053	0.034	-0.033	0.023	-0.033
SLFR_G3R	0.063	-0.005	0.087	-0.002	0.071
SLFR_G3R	-0.026	0.026	-0.026	0.018	-0.027
SLFR_G5R	0.080	-0.006	0.108	-0.002	0.087
SLFR_G5R	-0.029	0.023	-0.026	0.016	-0.030
SLFR_15R	-0.028	0.001	-0.025	0.000	-0.026
SLFR_15R	-0.009	0.006	-0.008	0.004	-0.009
READ	4.235	-0.122	4.095	-0.109	4.712
READ_SE	-0.300	0.269	-0.222	0.194	-0.279
WJPCSCG3	-0.055	-0.005	0.210	-0.009	0.416
WJPCSCG3	-0.255	0.182	-0.185	0.132	-0.214
WJPCSCG5	-0.305	-0.019	-0.449	-0.002	-0.340
WJPCSCG5	-0.132	0.095	-0.082	0.071	-0.107
WJPCSCX5	0.838	0.006	0.818	0.009	0.690
WJPCSCX5	-0.226	0.187	-0.179	0.131	-0.216
MATH	3.764	-0.095	3.770	-0.099	4.239
MATH_SE	-0.255	0.236	-0.192	0.168	-0.241
WJAPSC1S	1.818	-0.063	1.589	-0.056	1.901
WJAPSC1S	-0.303	0.291	-0.283	0.203	-0.325
WJAPSCG3	0.122	-0.020	0.386	-0.010	0.617
WJAPSCG3	-0.288	0.218	-0.208	0.157	-0.240
WJAPSCG5	-0.281	-0.004	0.270	-0.003	0.378
WJAPSCG5	-0.181	0.131	-0.109	0.098	-0.145
WJAPSCX5	0.501	0.059	0.428	0.000	0.242
WJAPSCX5	-0.167	0.142	-0.137	0.100	-0.164

Covariances

	MS_24M_S	MS_36M	MS_36M_S	MS_54M	MS_54M_S
MS_24M_S	0.038				
MS_36M	-0.013	0.877			
MS_36M_S	0.025	-0.012	0.025		
MS_54M	-0.014	0.659	-0.013	0.916	
MS_54M_S	0.028	-0.019	0.026	-0.018	0.048
MS_G1	-0.013	0.630	-0.015	0.657	-0.019
MS_G1_SE	0.026	-0.028	0.023	-0.027	0.040
MS_G3	-0.011	0.483	-0.012	0.476	-0.017
MS_G3_SE	0.020	-0.028	0.019	-0.026	0.032
MS_G5	-0.014	0.454	-0.013	0.439	-0.020
MS_G5_SE	0.020	-0.030	0.018	-0.030	0.030
MS_15	-0.012	0.428	-0.011	0.412	-0.014
MS_15_SE	0.026	-0.043	0.023	-0.041	0.039
MS_RI	-0.013	0.463	-0.012	0.442	-0.017
MS_RI_SE	0.009	-0.007	0.007	-0.006	0.010
MS_6MR	0.001	0.072	0.003	0.051	0.001
MS_6MR_S	0.022	-0.020	0.018	-0.023	0.021
MS_15MR	-0.001	0.037	-0.004	0.026	-0.001
MS_15MR_	0.012	-0.010	0.010	-0.008	0.012
MS_24MR	0.000	0.174	0.001	0.131	0.000
MS_24MR_	0.029	-0.011	0.018	-0.011	0.021
MS_36MR	0.000	0.413	0.000	0.217	-0.003
MS_36MR_	0.016	-0.009	0.017	-0.010	0.017
MS_54MR	0.000	0.196	-0.001	0.474	-0.001

MS_54MR_	0.019	-0.015	0.018	-0.014	0.035
MS_G1R	0.001	0.167	-0.003	0.215	-0.002
MS_G1R_S	0.017	-0.021	0.016	-0.020	0.028
MS_G3R	0.002	0.020	0.000	0.035	0.000
MS_G3R_S	0.012	-0.019	0.011	-0.018	0.020
MS_G5R	0.000	-0.009	0.000	-0.003	-0.003
MS_G5R_S	0.012	-0.019	0.010	-0.020	0.018
MS_15R	0.002	-0.036	0.002	-0.030	0.003
MS_15R_S	0.019	-0.034	0.017	-0.032	0.030
SR	-0.003	0.085	-0.003	0.077	-0.005
SR_SE	0.002	-0.002	0.002	-0.002	0.003
SLFR_G1R	-0.007	0.082	-0.002	0.096	-0.001
SLFR_G1R	0.039	-0.049	0.036	-0.041	0.065
SLFR_G3R	-0.004	0.094	-0.003	0.104	-0.006
SLFR_G3R	0.028	-0.031	0.025	-0.020	0.045
SLFR_G5R	-0.003	0.103	-0.004	0.099	-0.003
SLFR_G5R	0.025	-0.034	0.022	-0.028	0.040
SLFR_15R	-0.001	-0.030	-0.001	-0.029	0.000
SLFR_15R	0.007	-0.010	0.006	-0.011	0.011
READ	-0.156	4.823	-0.149	4.542	-0.239
READ_SE	0.293	-0.317	0.257	-0.300	0.425
WJPCSCG3	0.013	0.214	0.022	0.224	0.012
WJPCSCG3	0.200	-0.261	0.172	-0.231	0.307
WJPCSCG5	-0.002	-0.506	-0.001	-0.460	0.008
WJPCSCG5	0.106	-0.126	0.092	-0.123	0.158
WJPCSCX5	-0.012	0.935	-0.025	0.839	-0.029
WJPCSCX5	0.214	-0.270	0.182	-0.268	0.325
MATH	-0.146	4.439	-0.136	4.266	-0.208
MATH_SE	0.257	-0.271	0.226	-0.262	0.373
WJAPSC1S	-0.060	2.112	-0.063	2.026	-0.112
WJAPSC1S	0.349	-0.382	0.310	-0.352	0.540
WJAPSCG3	-0.015	0.951	-0.027	0.628	-0.052
WJAPSCG3	0.238	-0.298	0.206	-0.263	0.363
WJAPSCG5	-0.006	0.280	0.004	0.391	0.002
WJAPSCG5	0.145	-0.166	0.126	-0.160	0.215
WJAPSCX5	0.017	0.523	0.009	0.501	-0.013
WJAPSCX5	0.166	-0.195	0.141	-0.202	0.255

Covariances

	MS_G1	MS_G1_SE	MS_G3	MS_G3_SE	MS_G5
MS_G1	0.969				
MS_G1_SE	-0.025	0.048			
MS_G3	0.573	-0.018	0.735		
MS_G3_SE	-0.029	0.035	-0.021	0.050	
MS_G5	0.485	-0.021	0.438	-0.018	0.635
MS_G5_SE	-0.035	0.033	-0.028	0.039	-0.025
MS_15	0.449	-0.017	0.395	-0.018	0.399
MS_15_SE	-0.040	0.043	-0.040	0.050	-0.031
MS_RI	0.482	-0.020	0.411	-0.020	0.383
MS_RI_SE	-0.007	0.010	-0.006	0.010	-0.006
MS_6MR	0.040	-0.001	0.037	-0.004	0.020
MS_6MR_S	-0.020	0.022	-0.016	0.019	-0.016

MS_15MR	0.040	-0.002	0.031	0.001	0.016
MS_15MR_	-0.008	0.011	-0.008	0.010	-0.007
MS_24MR	0.107	-0.003	0.041	-0.004	0.041
MS_24MR_	-0.010	0.020	-0.009	0.016	-0.011
MS_36MR	0.148	-0.008	0.072	-0.008	0.071
MS_36MR_	-0.011	0.015	-0.009	0.013	-0.009
MS_54MR	0.175	-0.007	0.065	-0.006	0.056
MS_54MR_	-0.014	0.029	-0.013	0.023	-0.015
MS_G1R	0.487	-0.005	0.162	-0.009	0.102
MS_G1R_S	-0.019	0.035	-0.013	0.024	-0.016
MS_G3R	0.091	0.003	0.324	-0.001	0.055
MS_G3R_S	-0.020	0.022	-0.014	0.034	-0.012
MS_G5R	0.003	-0.001	0.027	0.002	0.252
MS_G5R_S	-0.023	0.020	-0.018	0.024	-0.016
MS_15R	-0.033	0.003	-0.016	0.002	0.016
MS_15R_S	-0.031	0.033	-0.031	0.038	-0.025
SR	0.084	-0.005	0.081	-0.004	0.071
SR_SE	-0.003	0.003	-0.002	0.004	-0.002
SLFR_G1R	0.102	-0.002	0.111	-0.004	0.067
SLFR_G1R	-0.043	0.072	-0.034	0.057	-0.038
SLFR_G3R	0.105	-0.006	0.116	-0.002	0.092
SLFR_G3R	-0.029	0.049	-0.028	0.055	-0.024
SLFR_G5R	0.103	-0.004	0.080	-0.003	0.069
SLFR_G5R	-0.034	0.042	-0.030	0.048	-0.026
SLFR_15R	-0.019	-0.001	-0.012	0.000	-0.018
SLFR_15R	-0.011	0.012	-0.009	0.014	-0.009
READ	4.981	-0.262	4.276	-0.237	4.008
READ_SE	-0.318	0.456	-0.245	0.493	-0.254
WJPCSCG3	0.228	-0.010	0.402	-0.023	0.203
WJPCSCG3	-0.237	0.325	-0.191	0.374	-0.195
WJPCSCG5	-0.438	-0.007	-0.376	0.002	-0.338
WJPCSCG5	-0.140	0.170	-0.102	0.188	-0.099
WJPCSCX5	0.857	0.007	0.617	0.010	0.641
WJPCSCX5	-0.294	0.348	-0.242	0.387	-0.231
MATH	4.664	-0.232	3.836	-0.209	3.591
MATH_SE	-0.271	0.402	-0.210	0.419	-0.223
WJAPSC1S	1.841	-0.152	1.364	-0.120	1.256
WJAPSC1S	-0.353	0.628	-0.266	0.515	-0.298
WJAPSCG3	0.796	-0.082	0.527	-0.043	0.377
WJAPSCG3	-0.265	0.386	-0.213	0.439	-0.224
WJAPSCG5	0.319	-0.020	0.197	-0.031	0.240
WJAPSCG5	-0.187	0.232	-0.130	0.256	-0.132
WJAPSCX5	0.427	0.023	0.423	0.006	0.465
WJAPSCX5	-0.227	0.272	-0.183	0.300	-0.190

Covariances

	MS_G5_SE	MS_15	MS_15_SE	MS_RI	MS_RI_SE
MS_G5_SE	0.058				
MS_15	-0.025	0.851			
MS_15_SE	0.055	-0.033	0.115		
MS_RI	-0.024	0.356	-0.033	0.362	
MS_RI_SE	0.010	-0.005	0.013	-0.005	0.003

MS_6MR	-0.013	0.002	-0.004	0.089	0.000
MS_6MR_S	0.020	-0.012	0.024	-0.016	0.008
MS_15MR	0.000	-0.015	-0.003	0.060	0.000
MS_15MR_	0.010	-0.005	0.012	-0.007	0.004
MS_24MR	-0.003	0.029	-0.010	0.085	-0.001
MS_24MR_	0.016	-0.009	0.020	-0.011	0.006
MS_36MR	-0.006	0.071	-0.010	0.101	-0.001
MS_36MR_	0.012	-0.008	0.016	-0.009	0.005
MS_54MR	-0.006	0.056	-0.008	0.079	-0.001
MS_54MR_	0.022	-0.011	0.029	-0.013	0.007
MS_G1R	-0.011	0.093	-0.007	0.120	-0.001
MS_G1R_S	0.023	-0.012	0.031	-0.015	0.007
MS_G3R	-0.003	0.039	-0.006	0.049	0.000
MS_G3R_S	0.026	-0.012	0.033	-0.013	0.006
MS_G5R	0.000	0.043	0.002	0.021	0.000
MS_G5R_S	0.037	-0.016	0.034	-0.015	0.006
MS_15R	0.000	0.495	0.000	-0.006	0.000
MS_15R_S	0.042	-0.026	0.090	-0.026	0.010
SR	-0.006	0.061	-0.007	0.070	-0.001
SR_SE	0.004	-0.002	0.006	-0.002	0.001
SLFR_G1R	0.000	0.028	0.000	0.068	-0.001
SLFR_G1R	0.053	-0.030	0.071	-0.034	0.015
SLFR_G3R	-0.003	0.078	-0.007	0.083	-0.001
SLFR_G3R	0.052	-0.022	0.069	-0.024	0.013
SLFR_G5R	-0.003	0.033	0.004	0.076	-0.001
SLFR_G5R	0.054	-0.034	0.071	-0.026	0.012
SLFR_15R	-0.001	-0.011	0.001	-0.017	0.000
SLFR_15R	0.016	-0.008	0.026	-0.008	0.003
READ	-0.291	3.353	-0.368	4.027	-0.066
READ_SE	0.530	-0.227	0.700	-0.242	0.129
WJPCSCG3	-0.040	0.028	-0.053	0.185	-0.003
WJPCSCG3	0.377	-0.145	0.490	-0.186	0.088
WJPCSCG5	-0.009	-0.272	0.015	-0.325	0.000
WJPCSCG5	0.223	-0.087	0.299	-0.095	0.048
WJPCSCX5	0.014	0.406	0.001	0.668	-0.001
WJPCSCX5	0.433	-0.184	0.720	-0.208	0.097
MATH	-0.235	3.061	-0.290	3.678	-0.057
MATH_SE	0.443	-0.199	0.587	-0.210	0.112
WJAPSC1S	-0.115	0.977	-0.151	1.386	-0.029
WJAPSC1S	0.512	-0.226	0.686	-0.272	0.137
WJAPSCG3	-0.039	0.201	0.041	0.433	-0.008
WJAPSCG3	0.430	-0.180	0.552	-0.212	0.104
WJAPSCG5	-0.008	0.151	-0.028	0.204	-0.002
WJAPSCG5	0.308	-0.119	0.393	-0.126	0.066
WJAPSCX5	0.004	0.411	-0.026	0.379	0.001
WJAPSCX5	0.335	-0.145	0.558	-0.159	0.075

Covariances

	MS_6MR	MS_6MR_S	MS_15MR	MS_15MR_	MS_24MR
MS_6MR	0.974				
MS_6MR_S	-0.001	0.037			
MS_15MR	0.016	-0.003	0.340		

MS_15MR_	0.001	0.011	0.000	0.007	
MS_24MR	-0.065	-0.006	-0.020	0.000	0.521
MS_24MR_	0.001	0.016	-0.001	0.009	0.000
MS_36MR	-0.017	-0.004	-0.023	-0.003	0.088
MS_36MR_	0.002	0.011	-0.003	0.006	0.001
MS_54MR	-0.038	-0.007	-0.034	-0.001	0.045
MS_54MR_	0.000	0.014	-0.001	0.008	0.000
MS_G1R	-0.048	-0.004	-0.021	-0.001	0.021
MS_G1R_S	-0.001	0.014	-0.002	0.007	-0.003
MS_G3R	-0.051	0.000	-0.029	-0.001	-0.044
MS_G3R_S	-0.003	0.012	0.000	0.006	-0.003
MS_G5R	-0.068	0.001	-0.044	0.000	-0.045
MS_G5R_S	-0.009	0.011	0.000	0.006	-0.002
MS_15R	-0.087	0.004	-0.075	0.002	-0.057
MS_15R_S	-0.003	0.018	-0.003	0.009	-0.008
SR	0.002	-0.002	0.004	-0.002	0.012
SR_SE	0.000	0.002	0.000	0.001	0.000
SLFR_G1R	0.019	-0.001	0.003	0.000	0.007
SLFR_G1R	-0.019	0.034	0.001	0.017	0.000
SLFR_G3R	-0.020	-0.004	0.004	-0.001	-0.011
SLFR_G3R	-0.002	0.026	-0.002	0.013	-0.003
SLFR_G5R	0.005	-0.005	0.033	-0.001	0.011
SLFR_G5R	-0.003	0.023	0.000	0.012	-0.004
SLFR_15R	-0.011	0.001	-0.008	0.000	-0.009
SLFR_15R	-0.001	0.007	0.001	0.003	-0.001
READ	0.208	-0.127	0.068	-0.082	0.685
READ_SE	-0.058	0.266	0.020	0.141	-0.037
WJPCSCG3	-0.240	-0.005	0.025	-0.007	0.231
WJPCSCG3	-0.069	0.183	0.001	0.098	-0.028
WJPCSCG5	0.020	-0.015	-0.123	-0.001	-0.015
WJPCSCG5	-0.037	0.097	0.013	0.053	-0.012
WJPCSCX5	0.170	0.004	0.151	0.007	0.023
WJPCSCX5	-0.018	0.193	0.028	0.098	-0.008
MATH	0.086	-0.100	0.092	-0.073	0.561
MATH_SE	-0.045	0.231	0.018	0.121	-0.031
WJAPSC1S	0.432	-0.063	0.204	-0.043	0.516
WJAPSC1S	-0.030	0.289	-0.011	0.149	-0.053
WJAPSCG3	-0.311	-0.020	-0.046	-0.008	0.184
WJAPSCG3	-0.076	0.218	0.004	0.116	-0.028
WJAPSCG5	-0.485	-0.005	0.066	-0.003	0.174
WJAPSCG5	-0.055	0.133	0.017	0.073	-0.019
WJAPSCX5	0.122	0.045	0.049	-0.001	-0.137
WJAPSCX5	-0.008	0.147	0.022	0.075	-0.006

Covariances

	MS_24MR_	MS_36MR	MS_36MR_	MS_54MR	MS_54MR_
MS_24MR_	0.022				
MS_36MR	0.000	0.312			
MS_36MR_	0.012	0.000	0.011		
MS_54MR	-0.001	0.116	-0.001	0.395	
MS_54MR_	0.015	-0.002	0.012	-0.001	0.026
MS_G1R	0.000	0.047	-0.002	0.096	-0.002

MS_G1R_S	0.014	-0.006	0.011	-0.006	0.021
MS_G3R	0.002	-0.029	0.000	-0.014	0.000
MS_G3R_S	0.010	-0.005	0.008	-0.004	0.015
MS_G5R	0.000	-0.030	0.000	-0.024	-0.003
MS_G5R_S	0.009	-0.004	0.007	-0.004	0.013
MS_15R	0.001	-0.030	0.001	-0.023	0.002
MS_15R_S	0.015	-0.008	0.012	-0.006	0.022
SR	-0.002	0.015	-0.002	0.007	-0.004
SR_SE	0.002	0.000	0.001	0.000	0.002
SLFR_G1R	-0.006	0.014	-0.001	0.028	0.000
SLFR_G1R	0.030	-0.015	0.024	-0.007	0.047
SLFR_G3R	-0.003	0.012	-0.002	0.021	-0.004
SLFR_G3R	0.022	-0.007	0.017	0.004	0.033
SLFR_G5R	-0.002	0.027	-0.003	0.023	-0.002
SLFR_G5R	0.020	-0.007	0.015	-0.001	0.029
SLFR_15R	0.000	-0.013	0.000	-0.012	0.000
SLFR_15R	0.006	-0.002	0.004	-0.003	0.008
READ	-0.123	0.796	-0.104	0.515	-0.179
READ_SE	0.228	-0.075	0.172	-0.058	0.308
WJPCSCG3	0.010	0.030	0.016	0.040	0.008
WJPCSCG3	0.158	-0.075	0.118	-0.045	0.227
WJPCSCG5	-0.002	-0.181	-0.001	-0.134	0.006
WJPCSCG5	0.084	-0.031	0.063	-0.027	0.117
WJPCSCX5	-0.008	0.268	-0.018	0.172	-0.022
WJPCSCX5	0.171	-0.063	0.125	-0.060	0.242
MATH	-0.115	0.761	-0.094	0.589	-0.155
MATH_SE	0.199	-0.061	0.151	-0.052	0.269
WJAPSC1S	-0.049	0.727	-0.045	0.641	-0.084
WJAPSC1S	0.273	-0.110	0.210	-0.080	0.397
WJAPSCG3	-0.012	0.518	-0.020	0.195	-0.041
WJAPSCG3	0.188	-0.086	0.140	-0.051	0.268
WJAPSCG5	-0.006	0.076	0.003	0.187	0.001
WJAPSCG5	0.115	-0.040	0.087	-0.035	0.159
WJAPSCX5	0.014	0.144	0.007	0.122	-0.011
WJAPSCX5	0.132	-0.036	0.097	-0.043	0.189

Covariances

	MS_G1R	MS_G1R_S	MS_G3R	MS_G3R_S	MS_G5R
MS_G1R	0.367				
MS_G1R_S	-0.004	0.026			
MS_G3R	0.042	0.002	0.275		
MS_G3R_S	-0.006	0.016	-0.001	0.023	
MS_G5R	-0.018	-0.001	0.006	0.001	0.231
MS_G5R_S	-0.007	0.014	-0.002	0.016	0.000
MS_15R	-0.026	0.003	-0.010	0.002	0.022
MS_15R_S	-0.005	0.024	-0.005	0.025	0.002
SR	0.014	-0.004	0.011	-0.003	0.001
SR_SE	-0.001	0.002	0.000	0.002	0.000
SLFR_G1R	0.034	-0.001	0.043	-0.003	-0.001
SLFR_G1R	-0.009	0.052	-0.001	0.037	-0.004
SLFR_G3R	0.022	-0.004	0.033	-0.002	0.009
SLFR_G3R	-0.005	0.035	-0.004	0.036	0.001

SLFR_G5R	0.027	-0.003	0.005	-0.002	-0.007
SLFR_G5R	-0.008	0.030	-0.004	0.032	0.001
SLFR_15R	-0.002	0.000	0.005	0.000	-0.001
SLFR_15R	-0.003	0.009	-0.001	0.009	-0.001
READ	0.954	-0.191	0.249	-0.158	-0.019
READ_SE	-0.076	0.324	-0.003	0.324	-0.012
WJPCSCG3	0.044	-0.009	0.217	-0.017	0.018
WJPCSCG3	-0.051	0.235	-0.005	0.251	-0.009
WJPCSCG5	-0.112	-0.006	-0.050	0.001	-0.013
WJPCSCG5	-0.045	0.123	-0.007	0.126	-0.004
WJPCSCX5	0.190	0.007	-0.050	0.008	-0.027
WJPCSCX5	-0.086	0.252	-0.035	0.259	-0.023
MATH	0.986	-0.169	0.159	-0.138	-0.087
MATH_SE	-0.061	0.285	-0.001	0.273	-0.013
WJAPSC1S	0.455	-0.113	-0.021	-0.081	-0.129
WJAPSC1S	-0.081	0.460	0.007	0.341	-0.026
WJAPSCG3	0.363	-0.064	0.094	-0.030	-0.055
WJAPSCG3	-0.053	0.278	-0.001	0.294	-0.012
WJAPSCG5	0.115	-0.016	-0.007	-0.023	0.036
WJAPSCG5	-0.061	0.167	-0.005	0.171	-0.006
WJAPSCX5	0.048	0.019	0.044	0.005	0.087
WJAPSCX5	-0.068	0.197	-0.024	0.201	-0.031

Covariances

	MS_G5R_S	MS_15R	MS_15R_S	SR	SR_SE
MS_G5R_S	0.023				
MS_15R	0.000	0.501			
MS_15R_S	0.026	0.001	0.071		
SR	-0.004	-0.009	-0.005	0.030	
SR_SE	0.002	0.000	0.004	0.000	0.000
SLFR_G1R	0.000	-0.041	0.000	0.035	0.000
SLFR_G1R	0.033	0.004	0.054	-0.007	0.006
SLFR_G3R	-0.002	-0.005	-0.006	0.039	0.000
SLFR_G3R	0.032	0.003	0.053	-0.007	0.005
SLFR_G5R	-0.002	-0.043	0.003	0.034	0.000
SLFR_G5R	0.033	-0.007	0.055	-0.006	0.005
SLFR_15R	-0.001	0.006	0.001	0.008	0.000
SLFR_15R	0.010	0.000	0.020	-0.002	0.002
READ	-0.183	-0.674	-0.287	1.375	-0.023
READ_SE	0.326	0.015	0.536	-0.055	0.052
WJPCSCG3	-0.027	-0.156	-0.043	0.050	-0.002
WJPCSCG3	0.236	0.041	0.379	-0.044	0.036
WJPCSCG5	-0.007	0.053	0.011	-0.066	0.001
WJPCSCG5	0.141	0.009	0.233	-0.020	0.021
WJPCSCX5	0.010	-0.261	0.000	0.173	-0.001
WJPCSCX5	0.272	0.024	0.564	-0.044	0.044
MATH	-0.145	-0.617	-0.224	1.284	-0.019
MATH_SE	0.271	0.011	0.449	-0.048	0.045
WJAPSC1S	-0.072	-0.409	-0.118	0.515	-0.010
WJAPSC1S	0.317	0.046	0.530	-0.070	0.052
WJAPSCG3	-0.026	-0.232	0.034	0.249	-0.002
WJAPSCG3	0.268	0.032	0.426	-0.052	0.042

WJAPSCG5	-0.005	-0.053	-0.023	0.174	0.000
WJAPSCG5	0.194	0.006	0.305	-0.027	0.028
WJAPSCX5	0.004	0.032	-0.020	0.140	-0.001
WJAPSCX5	0.210	0.014	0.437	-0.034	0.035

Covariances

	SLFR_G1R	SLFR_G1R	SLFR_G3R	SLFR_G3R	SLFR_G5R
SLFR_G1R	0.796				
SLFR_G1R	-0.004	0.172			
SLFR_G3R	0.143	-0.006	0.583		
SLFR_G3R	-0.008	0.097	-0.003	0.117	
SLFR_G5R	0.073	0.001	0.147	-0.004	0.507
SLFR_G5R	-0.004	0.080	-0.007	0.080	-0.003
SLFR_15R	-0.019	0.001	-0.026	0.000	-0.042
SLFR_15R	0.000	0.020	-0.002	0.020	-0.001
READ	0.621	-0.343	1.567	-0.305	1.309
READ_SE	-0.004	0.750	-0.018	0.688	-0.020
WJPCSCG3	0.211	-0.004	0.194	-0.047	0.099
WJPCSCG3	-0.014	0.555	-0.029	0.527	-0.046
WJPCSCG5	-0.346	0.040	-0.258	0.009	0.059
WJPCSCG5	0.015	0.278	-0.005	0.263	-0.020
WJPCSCX5	0.091	-0.010	0.517	0.014	0.775
WJPCSCX5	0.008	0.567	-0.011	0.534	0.013
MATH	0.961	-0.327	1.544	-0.261	1.299
MATH_SE	-0.013	0.659	-0.019	0.585	-0.015
WJAPSC1S	1.151	-0.279	0.820	-0.151	0.486
WJAPSC1S	0.005	1.020	-0.058	0.733	-0.029
WJAPSCG3	0.373	-0.073	0.434	-0.046	-0.038
WJAPSCG3	-0.027	0.660	-0.024	0.619	-0.045
WJAPSCG5	0.232	0.014	0.125	-0.017	0.240
WJAPSCG5	0.023	0.381	-0.001	0.357	-0.030
WJAPSCX5	0.283	-0.061	0.549	-0.021	0.621
WJAPSCX5	-0.001	0.444	0.002	0.416	0.018

Covariances

	SLFR_G5R	SLFR_15R	SLFR_15R	READ	READ_SE
SLFR_G5R	0.105				
SLFR_15R	0.001	0.067			
SLFR_15R	0.021	0.000	0.008		
READ	-0.308	-0.284	-0.082	109.668	
READ_SE	0.672	0.004	0.201	-2.856	7.111
WJPCSCG3	0.029	-0.124	-0.013	7.673	-0.247
WJPCSCG3	0.488	0.014	0.144	-2.154	5.007
WJPCSCG5	0.012	0.080	0.004	1.205	0.004
WJPCSCG5	0.269	0.007	0.087	-1.085	2.850
WJPCSCX5	-0.050	-0.068	-0.004	18.637	-0.021
WJPCSCX5	0.543	0.012	0.211	-2.541	5.827
MATH	-0.244	-0.220	-0.064	95.490	-2.286
MATH_SE	0.565	0.001	0.168	-2.520	5.994
WJAPSC1S	-0.135	-0.217	-0.034	26.408	-1.249

WJAPSC1S	0.662	0.001	0.198	-3.665	6.978
WJAPSCG3	-0.051	-0.139	-0.001	12.433	-0.311
WJAPSCG3	0.564	0.012	0.163	-2.439	5.812
WJAPSCG5	0.042	-0.306	-0.002	3.983	-0.146
WJAPSCG5	0.369	0.007	0.114	-1.377	3.885
WJAPSCX5	-0.038	-0.290	-0.004	8.382	0.034
WJAPSCX5	0.424	0.004	0.164	-1.917	4.513

Covariances

	WJPCSCG3	WJPCSCG3	WJPCSCG5	WJPCSCG5	WJPCSCX5
WJPCSCG3	37.998				
WJPCSCG3	-0.234	4.498			
WJPCSCG5	-3.832	0.157	16.140		
WJPCSCG5	-0.215	2.159	-0.009	1.341	
WJPCSCX5	-7.521	-0.200	-16.420	-0.028	43.606
WJPCSCX5	-0.399	4.385	0.008	2.733	0.049
MATH	2.012	-1.769	-4.349	-0.748	13.717
MATH_SE	-0.175	4.139	0.008	2.335	-0.013
WJAPSC1S	6.504	-1.252	-6.172	-0.289	3.816
WJAPSC1S	-0.131	5.156	-0.191	2.775	0.252
WJAPSCG3	9.669	-0.455	-6.279	-0.106	-2.693
WJAPSCG3	-0.227	5.074	0.179	2.399	-0.187
WJAPSCG5	2.094	-0.356	-4.325	-0.101	-1.971
WJAPSCG5	-0.251	2.894	-0.026	1.804	-0.024
WJAPSCX5	-5.671	-0.034	-11.408	0.044	8.372
WJAPSCX5	-0.217	3.377	0.040	2.088	0.018

Covariances

	WJPCSCX5	MATH	MATH_SE	WJAPSC1S	WJAPSC1S
WJPCSCX5	6.993				
MATH	-1.851	97.425			
MATH_SE	4.804	-2.094	5.109		
WJAPSC1S	-0.582	35.890	-1.149	89.865	
WJAPSC1S	5.815	-3.136	6.011	-2.061	9.477
WJAPSCG3	-0.279	17.636	-0.280	15.111	-1.237
WJAPSCG3	4.829	-2.043	4.835	-1.536	6.043
WJAPSCG5	-0.404	12.108	-0.128	2.129	-0.430
WJAPSCG5	3.480	-0.956	3.188	-0.414	3.791
WJAPSCX5	0.052	15.096	0.019	-3.450	0.363
WJAPSCX5	5.328	-1.495	3.744	-0.516	4.544

Covariances

	WJAPSCG3	WJAPSCG3	WJAPSCG5	WJAPSCG5	WJAPSCX5
WJAPSCG3	53.178				
WJAPSCG3	-0.497	5.872			
WJAPSCG5	5.203	-0.425	27.413		
WJAPSCG5	-0.136	3.240	-0.109	2.475	
WJAPSCX5	-5.850	-0.025	-3.671	0.076	33.520

WJAPSCX5	-0.174	3.742	-0.396	2.673	0.028

Covariances

	WJAPSCX5
WJAPSCX5	4.175

Correlations

	MS_6M	MS_6M_SE	MS_15M	MS_15M_S	MS_24M
MS_6M	1.000				
MS_6M_SE	-0.056	1.000			
MS_15M	0.473	-0.092	1.000		
MS_15M_S	-0.048	0.632	-0.080	1.000	
MS_24M	0.374	-0.093	0.524	-0.072	1.000
MS_24M_S	-0.049	0.488	-0.080	0.727	-0.065
MS_36M	0.465	-0.090	0.590	-0.105	0.663
MS_36M_S	-0.046	0.480	-0.109	0.716	-0.067
MS_54M	0.418	-0.104	0.539	-0.084	0.583
MS_54M_S	-0.058	0.392	-0.091	0.583	-0.073
MS_G1	0.431	-0.085	0.584	-0.082	0.583
MS_G1_SE	-0.079	0.396	-0.113	0.539	-0.106
MS_G3	0.425	-0.076	0.568	-0.093	0.514
MS_G3_SE	-0.086	0.324	-0.094	0.453	-0.103
MS_G5	0.411	-0.080	0.552	-0.094	0.518
MS_G5_SE	-0.125	0.309	-0.110	0.431	-0.111
MS_15	0.316	-0.052	0.408	-0.058	0.406
MS_15_SE	-0.090	0.262	-0.119	0.357	-0.124
MS_RI	0.609	-0.111	0.774	-0.121	0.725
MS_RI_SE	-0.083	0.620	-0.111	0.815	-0.103
MS_6MR	0.875	-0.002	0.117	0.014	0.024
MS_6MR_S	-0.071	0.982	-0.111	0.689	-0.111
MS_15MR	0.107	-0.029	0.757	0.001	0.067
MS_15MR_	-0.058	0.604	-0.091	0.992	-0.083
MS_24MR	0.023	-0.039	0.100	-0.002	0.818
MS_24MR_	-0.055	0.466	-0.086	0.698	-0.070
MS_36MR	0.123	-0.031	0.154	-0.046	0.330
MS_36MR_	-0.052	0.443	-0.118	0.670	-0.073
MS_54MR	0.053	-0.052	0.079	-0.012	0.193
MS_54MR_	-0.061	0.349	-0.091	0.529	-0.075
MS_G1R	0.096	-0.028	0.180	-0.013	0.226
MS_G1R_S	-0.081	0.352	-0.114	0.482	-0.108
MS_G3R	-0.004	0.003	0.040	-0.013	0.008
MS_G3R_S	-0.088	0.280	-0.095	0.398	-0.103
MS_G5R	-0.081	0.005	-0.054	-0.004	-0.048
MS_G5R_S	-0.128	0.264	-0.109	0.376	-0.111
MS_15R	-0.107	0.026	-0.126	0.027	-0.086
MS_15R_S	-0.090	0.238	-0.120	0.326	-0.124
SR	0.339	-0.050	0.470	-0.086	0.461
SR_SE	-0.093	0.418	-0.102	0.580	-0.110
SLFR_G1R	0.079	-0.006	0.087	-0.003	0.082
SLFR_G1R	-0.103	0.318	-0.087	0.430	-0.079

SLFR_G3R	0.067	-0.024	0.126	-0.017	0.091
SLFR_G3R	-0.061	0.286	-0.085	0.397	-0.077
SLFR_G5R	0.092	-0.032	0.168	-0.019	0.118
SLFR_G5R	-0.074	0.271	-0.088	0.389	-0.090
SLFR_15R	-0.088	0.011	-0.105	0.004	-0.098
SLFR_15R	-0.081	0.270	-0.093	0.372	-0.100
READ	0.329	-0.045	0.431	-0.080	0.438
READ_SE	-0.091	0.386	-0.092	0.559	-0.102
WJPCSCG3	-0.007	-0.003	0.038	-0.011	0.066
WJPCSCG3	-0.098	0.328	-0.096	0.477	-0.098
WJPCSCG5	-0.062	-0.018	-0.123	-0.004	-0.082
WJPCSCG5	-0.093	0.315	-0.079	0.471	-0.090
WJPCSCX5	0.103	0.004	0.137	0.010	0.102
WJPCSCX5	-0.069	0.271	-0.075	0.382	-0.079
MATH	0.310	-0.037	0.421	-0.077	0.418
MATH_SE	-0.092	0.400	-0.094	0.572	-0.104
WJAPSC1S	0.156	-0.025	0.185	-0.045	0.195
WJAPSC1S	-0.080	0.362	-0.102	0.508	-0.103
WJAPSCG3	0.014	-0.010	0.058	-0.011	0.082
WJAPSCG3	-0.097	0.344	-0.095	0.498	-0.097
WJAPSCG5	-0.044	-0.003	0.057	-0.004	0.070
WJAPSCG5	-0.094	0.319	-0.077	0.480	-0.090
WJAPSCX5	0.070	0.039	0.081	0.000	0.041
WJAPSCX5	-0.066	0.266	-0.074	0.378	-0.078

Correlations

	MS_24M_S	MS_36M	MS_36M_S	MS_54M	MS_54M_S
MS_24M_S	1.000				
MS_36M	-0.070	1.000			
MS_36M_S	0.804	-0.083	1.000		
MS_54M	-0.073	0.736	-0.087	1.000	
MS_54M_S	0.650	-0.094	0.734	-0.085	1.000
MS_G1	-0.066	0.684	-0.096	0.697	-0.087
MS_G1_SE	0.604	-0.140	0.663	-0.131	0.830
MS_G3	-0.065	0.602	-0.091	0.581	-0.089
MS_G3_SE	0.467	-0.132	0.522	-0.121	0.646
MS_G5	-0.089	0.608	-0.100	0.576	-0.116
MS_G5_SE	0.434	-0.133	0.470	-0.133	0.568
MS_15	-0.064	0.495	-0.073	0.467	-0.071
MS_15_SE	0.389	-0.136	0.430	-0.127	0.532
MS_RI	-0.113	0.822	-0.127	0.767	-0.127
MS_RI_SE	0.781	-0.124	0.823	-0.117	0.799
MS_6MR	0.008	0.078	0.021	0.053	0.005
MS_6MR_S	0.572	-0.112	0.571	-0.127	0.501
MS_15MR	-0.008	0.069	-0.038	0.047	-0.010
MS_15MR_	0.739	-0.122	0.730	-0.098	0.623
MS_24MR	0.002	0.257	0.011	0.189	0.002
MS_24MR_	0.996	-0.078	0.787	-0.080	0.658
MS_36MR	0.004	0.790	-0.002	0.407	-0.021
MS_36MR_	0.773	-0.094	0.993	-0.097	0.730
MS_54MR	-0.003	0.333	-0.010	0.788	-0.008
MS_54MR_	0.605	-0.098	0.686	-0.088	0.995

MS_G1R	0.006	0.293	-0.029	0.371	-0.015
MS_G1R_S	0.560	-0.142	0.616	-0.134	0.804
MS_G3R	0.024	0.040	-0.002	0.069	0.000
MS_G3R_S	0.422	-0.134	0.477	-0.123	0.614
MS_G5R	-0.005	-0.021	-0.007	-0.006	-0.033
MS_G5R_S	0.390	-0.134	0.423	-0.135	0.534
MS_15R	0.012	-0.054	0.014	-0.044	0.016
MS_15R_S	0.365	-0.137	0.405	-0.127	0.513
SR	-0.085	0.523	-0.095	0.463	-0.121
SR_SE	0.583	-0.131	0.629	-0.128	0.739
SLFR_G1R	-0.043	0.099	-0.012	0.113	-0.004
SLFR_G1R	0.481	-0.126	0.549	-0.103	0.715
SLFR_G3R	-0.028	0.132	-0.021	0.142	-0.033
SLFR_G3R	0.417	-0.097	0.465	-0.062	0.598
SLFR_G5R	-0.018	0.154	-0.033	0.146	-0.019
SLFR_G5R	0.404	-0.111	0.429	-0.089	0.567
SLFR_15R	-0.011	-0.122	-0.015	-0.119	0.003
SLFR_15R	0.401	-0.116	0.438	-0.124	0.567
READ	-0.076	0.492	-0.089	0.453	-0.105
READ_SE	0.563	-0.127	0.604	-0.118	0.730
WJPCSCG3	0.010	0.037	0.023	0.038	0.009
WJPCSCG3	0.483	-0.131	0.509	-0.114	0.663
WJPCSCG5	-0.002	-0.135	-0.002	-0.120	0.009
WJPCSCG5	0.470	-0.117	0.499	-0.111	0.624
WJPCSCX5	-0.009	0.151	-0.024	0.133	-0.020
WJPCSCX5	0.415	-0.109	0.431	-0.106	0.563
MATH	-0.076	0.480	-0.086	0.452	-0.097
MATH_SE	0.582	-0.128	0.628	-0.121	0.755
WJAPSC1S	-0.032	0.238	-0.042	0.223	-0.054
WJAPSC1S	0.581	-0.133	0.632	-0.119	0.804
WJAPSCG3	-0.010	0.139	-0.023	0.090	-0.033
WJAPSCG3	0.504	-0.132	0.533	-0.114	0.686
WJAPSCG5	-0.006	0.057	0.005	0.078	0.002
WJAPSCG5	0.472	-0.113	0.505	-0.107	0.626
WJAPSCX5	0.015	0.096	0.010	0.090	-0.010
WJAPSCX5	0.416	-0.102	0.434	-0.104	0.571

Correlations

	MS_G1	MS_G1_SE	MS_G3	MS_G3_SE	MS_G5
MS_G1	1.000				
MS_G1_SE	-0.117	1.000			
MS_G3	0.679	-0.094	1.000		
MS_G3_SE	-0.132	0.708	-0.110	1.000	
MS_G5	0.618	-0.122	0.641	-0.103	1.000
MS_G5_SE	-0.149	0.624	-0.134	0.727	-0.129
MS_15	0.495	-0.085	0.499	-0.086	0.542
MS_15_SE	-0.120	0.583	-0.136	0.657	-0.117
MS_RI	0.813	-0.155	0.796	-0.149	0.799
MS_RI_SE	-0.122	0.798	-0.116	0.777	-0.122
MS_6MR	0.042	-0.004	0.044	-0.017	0.026
MS_6MR_S	-0.107	0.511	-0.096	0.440	-0.101
MS_15MR	0.069	-0.016	0.062	0.007	0.034

MS_15MR_	-0.095	0.584	-0.107	0.496	-0.106
MS_24MR	0.150	-0.022	0.066	-0.022	0.071
MS_24MR_	-0.071	0.619	-0.069	0.482	-0.094
MS_36MR	0.269	-0.067	0.151	-0.061	0.159
MS_36MR_	-0.107	0.669	-0.100	0.532	-0.108
MS_54MR	0.283	-0.051	0.121	-0.043	0.111
MS_54MR_	-0.089	0.818	-0.092	0.638	-0.119
MS_G1R	0.816	-0.037	0.311	-0.067	0.211
MS_G1R_S	-0.118	0.995	-0.093	0.685	-0.122
MS_G3R	0.176	0.024	0.720	-0.010	0.131
MS_G3R_S	-0.134	0.681	-0.110	0.995	-0.101
MS_G5R	0.007	-0.008	0.066	0.015	0.657
MS_G5R_S	-0.150	0.594	-0.134	0.697	-0.128
MS_15R	-0.047	0.021	-0.027	0.014	0.028
MS_15R_S	-0.120	0.566	-0.137	0.639	-0.116
SR	0.489	-0.137	0.543	-0.115	0.512
SR_SE	-0.128	0.792	-0.122	0.819	-0.131
SLFR_G1R	0.116	-0.012	0.145	-0.021	0.094
SLFR_G1R	-0.105	0.798	-0.097	0.610	-0.114
SLFR_G3R	0.139	-0.033	0.177	-0.012	0.151
SLFR_G3R	-0.086	0.650	-0.095	0.718	-0.086
SLFR_G5R	0.147	-0.029	0.132	-0.020	0.122
SLFR_G5R	-0.107	0.592	-0.108	0.668	-0.099
SLFR_15R	-0.074	-0.011	-0.052	0.005	-0.088
SLFR_15R	-0.122	0.621	-0.121	0.697	-0.126
READ	0.483	-0.115	0.476	-0.101	0.480
READ_SE	-0.121	0.785	-0.107	0.827	-0.120
WJPCSCG3	0.038	-0.008	0.076	-0.017	0.041
WJPCSCG3	-0.114	0.704	-0.105	0.789	-0.115
WJPCSCG5	-0.111	-0.008	-0.109	0.002	-0.106
WJPCSCG5	-0.123	0.672	-0.103	0.727	-0.107
WJPCSCX5	0.132	0.005	0.109	0.007	0.122
WJPCSCX5	-0.113	0.604	-0.107	0.654	-0.110
MATH	0.480	-0.108	0.453	-0.094	0.456
MATH_SE	-0.122	0.815	-0.109	0.828	-0.124
WJAPSC1S	0.197	-0.073	0.168	-0.057	0.166
WJAPSC1S	-0.117	0.936	-0.101	0.748	-0.121
WJAPSCG3	0.111	-0.052	0.084	-0.026	0.065
WJAPSCG3	-0.111	0.732	-0.103	0.810	-0.116
WJAPSCG5	0.062	-0.018	0.044	-0.027	0.058
WJAPSCG5	-0.121	0.676	-0.097	0.728	-0.105
WJAPSCX5	0.075	0.018	0.085	0.004	0.101
WJAPSCX5	-0.113	0.610	-0.105	0.657	-0.117

Correlations

	MS_G5_SE	MS_15	MS_15_SE	MS_RI	MS_RI_SE
MS_G5_SE	1.000				
MS_15	-0.112	1.000			
MS_15_SE	0.679	-0.106	1.000		
MS_RI	-0.168	0.641	-0.164	1.000	
MS_RI_SE	0.751	-0.097	0.670	-0.160	1.000
MS_6MR	-0.053	0.002	-0.012	0.149	-0.006

MS_6MR_S	0.424	-0.067	0.367	-0.138	0.720
MS_15MR	0.003	-0.027	-0.016	0.172	-0.007
MS_15MR_	0.477	-0.064	0.397	-0.137	0.828
MS_24MR	-0.018	0.043	-0.040	0.197	-0.014
MS_24MR_	0.452	-0.068	0.407	-0.120	0.770
MS_36MR	-0.043	0.138	-0.052	0.300	-0.035
MS_36MR_	0.483	-0.078	0.446	-0.138	0.796
MS_54MR	-0.042	0.097	-0.036	0.210	-0.025
MS_54MR_	0.564	-0.071	0.531	-0.129	0.756
MS_G1R	-0.075	0.166	-0.032	0.328	-0.039
MS_G1R_S	0.608	-0.083	0.572	-0.155	0.746
MS_G3R	-0.027	0.080	-0.035	0.154	-0.006
MS_G3R_S	0.710	-0.084	0.645	-0.148	0.725
MS_G5R	-0.004	0.096	0.012	0.072	-0.002
MS_G5R_S	0.995	-0.110	0.658	-0.166	0.695
MS_15R	-0.003	0.758	0.001	-0.015	0.010
MS_15R_S	0.662	-0.105	0.998	-0.164	0.637
SR	-0.134	0.382	-0.115	0.670	-0.121
SR_SE	0.809	-0.107	0.814	-0.164	0.884
SLFR_G1R	0.001	0.033	0.000	0.127	-0.013
SLFR_G1R	0.537	-0.078	0.502	-0.135	0.660
SLFR_G3R	-0.018	0.110	-0.028	0.180	-0.022
SLFR_G3R	0.630	-0.068	0.596	-0.118	0.667
SLFR_G5R	-0.017	0.050	0.015	0.177	-0.020
SLFR_G5R	0.696	-0.113	0.650	-0.135	0.660
SLFR_15R	-0.012	-0.048	0.009	-0.109	-0.003
SLFR_15R	0.722	-0.099	0.841	-0.149	0.684
READ	-0.116	0.347	-0.104	0.639	-0.111
READ_SE	0.829	-0.092	0.774	-0.151	0.856
WJPCSCG3	-0.027	0.005	-0.025	0.050	-0.009
WJPCSCG3	0.741	-0.074	0.681	-0.146	0.734
WJPCSCG5	-0.009	-0.073	0.011	-0.135	-0.001
WJPCSCG5	0.804	-0.081	0.763	-0.137	0.733
WJPCSCX5	0.009	0.067	0.000	0.168	-0.003
WJPCSCX5	0.683	-0.075	0.804	-0.130	0.649
MATH	-0.099	0.336	-0.087	0.619	-0.103
MATH_SE	0.817	-0.096	0.767	-0.154	0.875
WJAPSC1S	-0.050	0.112	-0.047	0.243	-0.054
WJAPSC1S	0.693	-0.080	0.658	-0.147	0.788
WJAPSCG3	-0.022	0.030	0.017	0.099	-0.019
WJAPSCG3	0.740	-0.081	0.673	-0.145	0.757
WJAPSCG5	-0.007	0.031	-0.016	0.065	-0.008
WJAPSCG5	0.817	-0.082	0.738	-0.133	0.740
WJAPSCX5	0.003	0.077	-0.013	0.109	0.003
WJAPSCX5	0.684	-0.077	0.806	-0.129	0.653

Correlations

	MS_6MR	MS_6MR_S	MS_15MR	MS_15MR_	MS_24MR
MS_6MR	1.000				
MS_6MR_S	-0.004	1.000			
MS_15MR	0.028	-0.029	1.000		
MS_15MR_	0.012	0.676	0.000	1.000	

MS_24MR	-0.091	-0.042	-0.047	-0.003	1.000
MS_24MR_	0.005	0.556	-0.009	0.717	0.000
MS_36MR	-0.030	-0.039	-0.070	-0.057	0.219
MS_36MR_	0.019	0.540	-0.041	0.694	0.011
MS_54MR	-0.061	-0.060	-0.094	-0.018	0.100
MS_54MR_	0.003	0.460	-0.008	0.574	0.001
MS_G1R	-0.080	-0.036	-0.058	-0.019	0.049
MS_G1R_S	-0.006	0.468	-0.018	0.532	-0.024
MS_G3R	-0.099	0.001	-0.096	-0.017	-0.117
MS_G3R_S	-0.019	0.397	0.005	0.447	-0.023
MS_G5R	-0.144	0.006	-0.158	-0.004	-0.129
MS_G5R_S	-0.058	0.379	0.003	0.428	-0.019
MS_15R	-0.124	0.030	-0.182	0.033	-0.111
MS_15R_S	-0.012	0.343	-0.018	0.370	-0.041
SR	0.013	-0.070	0.040	-0.102	0.097
SR_SE	-0.016	0.541	0.010	0.621	-0.020
SLFR_G1R	0.022	-0.007	0.005	-0.005	0.012
SLFR_G1R	-0.047	0.419	0.003	0.473	0.000
SLFR_G3R	-0.027	-0.028	0.009	-0.021	-0.021
SLFR_G3R	-0.005	0.389	-0.011	0.438	-0.011
SLFR_G5R	0.007	-0.037	0.079	-0.023	0.021
SLFR_G5R	-0.010	0.373	0.002	0.431	-0.016
SLFR_15R	-0.044	0.011	-0.051	0.005	-0.049
SLFR_15R	-0.011	0.380	0.010	0.417	-0.018
READ	0.020	-0.063	0.011	-0.090	0.091
READ_SE	-0.022	0.515	0.013	0.612	-0.019
WJPCSCG3	-0.039	-0.004	0.007	-0.013	0.052
WJPCSCG3	-0.033	0.447	0.001	0.536	-0.019
WJPCSCG5	0.005	-0.019	-0.053	-0.004	-0.005
WJPCSCG5	-0.033	0.434	0.019	0.531	-0.014
WJPCSCX5	0.026	0.003	0.039	0.012	0.005
WJPCSCX5	-0.007	0.378	0.018	0.432	-0.004
MATH	0.009	-0.052	0.016	-0.086	0.079
MATH_SE	-0.020	0.529	0.014	0.622	-0.019
WJAPSC1S	0.046	-0.035	0.037	-0.053	0.075
WJAPSC1S	-0.010	0.485	-0.006	0.562	-0.024
WJAPSCG3	-0.043	-0.014	-0.011	-0.013	0.035
WJAPSCG3	-0.032	0.465	0.003	0.557	-0.016
WJAPSCG5	-0.094	-0.005	0.021	-0.006	0.046
WJAPSCG5	-0.036	0.439	0.018	0.539	-0.017
WJAPSCX5	0.021	0.040	0.014	-0.001	-0.033
WJAPSCX5	-0.004	0.373	0.018	0.427	-0.004

Correlations

	MS_24MR_	MS_36MR	MS_36MR_	MS_54MR	MS_54MR_
MS_24MR_	1.000				
MS_36MR	-0.001	1.000			
MS_36MR_	0.764	-0.008	1.000		
MS_54MR	-0.006	0.332	-0.016	1.000	
MS_54MR_	0.618	-0.025	0.689	-0.011	1.000
MS_G1R	0.004	0.139	-0.037	0.251	-0.017
MS_G1R_S	0.580	-0.072	0.630	-0.055	0.799

MS_G3R	0.025	-0.098	-0.005	-0.043	-0.001
MS_G3R_S	0.443	-0.065	0.494	-0.046	0.614
MS_G5R	-0.006	-0.112	-0.007	-0.078	-0.036
MS_G5R_S	0.413	-0.045	0.444	-0.046	0.537
MS_15R	0.014	-0.075	0.015	-0.053	0.018
MS_15R_S	0.386	-0.054	0.424	-0.037	0.515
SR	-0.091	0.155	-0.103	0.064	-0.125
SR_SE	0.593	-0.043	0.630	-0.039	0.722
SLFR_G1R	-0.046	0.029	-0.012	0.050	-0.003
SLFR_G1R	0.496	-0.066	0.559	-0.028	0.711
SLFR_G3R	-0.030	0.027	-0.023	0.043	-0.034
SLFR_G3R	0.432	-0.036	0.474	0.018	0.593
SLFR_G5R	-0.019	0.067	-0.037	0.053	-0.018
SLFR_G5R	0.422	-0.040	0.439	-0.007	0.564
SLFR_15R	-0.012	-0.088	-0.017	-0.076	0.004
SLFR_15R	0.421	-0.034	0.454	-0.047	0.567
READ	-0.080	0.136	-0.095	0.078	-0.106
READ_SE	0.580	-0.050	0.613	-0.035	0.719
WJPCSCG3	0.011	0.009	0.025	0.010	0.008
WJPCSCG3	0.507	-0.063	0.528	-0.034	0.665
WJPCSCG5	-0.003	-0.081	-0.002	-0.053	0.010
WJPCSCG5	0.495	-0.048	0.519	-0.038	0.626
WJPCSCX5	-0.009	0.073	-0.025	0.041	-0.021
WJPCSCX5	0.438	-0.043	0.450	-0.036	0.568
MATH	-0.079	0.138	-0.091	0.095	-0.097
MATH_SE	0.597	-0.048	0.633	-0.037	0.740
WJAPSC1S	-0.035	0.137	-0.045	0.108	-0.055
WJAPSC1S	0.604	-0.064	0.649	-0.041	0.801
WJAPSCG3	-0.011	0.127	-0.026	0.043	-0.035
WJAPSCG3	0.527	-0.064	0.550	-0.034	0.687
WJAPSCG5	-0.007	0.026	0.005	0.057	0.001
WJAPSCG5	0.496	-0.046	0.524	-0.035	0.627
WJAPSCX5	0.016	0.045	0.012	0.034	-0.011
WJAPSCX5	0.440	-0.032	0.452	-0.034	0.576

Correlations

	MS_G1R	MS_G1R_S	MS_G3R	MS_G3R_S	MS_G5R
MS_G1R	1.000				
MS_G1R_S	-0.039	1.000			
MS_G3R	0.132	0.026	1.000		
MS_G3R_S	-0.070	0.664	-0.010	1.000	
MS_G5R	-0.060	-0.008	0.025	0.017	1.000
MS_G5R_S	-0.079	0.586	-0.029	0.687	-0.005
MS_15R	-0.062	0.023	-0.027	0.016	0.064
MS_15R_S	-0.032	0.559	-0.037	0.631	0.012
SR	0.129	-0.140	0.118	-0.114	0.010
SR_SE	-0.045	0.762	-0.011	0.786	-0.013
SLFR_G1R	0.063	-0.010	0.092	-0.022	-0.003
SLFR_G1R	-0.037	0.792	-0.004	0.591	-0.021
SLFR_G3R	0.047	-0.034	0.082	-0.014	0.025
SLFR_G3R	-0.023	0.633	-0.020	0.702	0.004
SLFR_G5R	0.064	-0.030	0.012	-0.021	-0.019

SLFR_G5R	-0.039	0.575	-0.022	0.652	0.004
SLFR_15R	-0.011	-0.011	0.039	0.006	-0.010
SLFR_15R	-0.051	0.609	-0.027	0.684	-0.023
READ	0.150	-0.114	0.045	-0.099	-0.004
READ_SE	-0.047	0.761	-0.002	0.802	-0.010
WJPCSCG3	0.012	-0.009	0.067	-0.018	0.006
WJPCSCG3	-0.040	0.692	-0.005	0.782	-0.009
WJPCSCG5	-0.046	-0.010	-0.024	0.001	-0.007
WJPCSCG5	-0.064	0.662	-0.012	0.717	-0.007
WJPCSCX5	0.047	0.007	-0.015	0.008	-0.008
WJPCSCX5	-0.054	0.597	-0.025	0.647	-0.018
MATH	0.165	-0.107	0.031	-0.092	-0.018
MATH_SE	-0.045	0.789	0.000	0.798	-0.012
WJAPSC1S	0.079	-0.075	-0.004	-0.057	-0.028
WJAPSC1S	-0.043	0.935	0.004	0.731	-0.017
WJAPSCG3	0.082	-0.055	0.024	-0.027	-0.016
WJAPSCG3	-0.036	0.718	-0.001	0.801	-0.011
WJAPSCG5	0.036	-0.020	-0.003	-0.028	0.014
WJAPSCG5	-0.064	0.665	-0.006	0.717	-0.008
WJAPSCX5	0.014	0.020	0.015	0.005	0.031
WJAPSCX5	-0.055	0.604	-0.023	0.649	-0.032

Correlations

	MS_G5R_S	MS_15R	MS_15R_S	SR	SR_SE
MS_G5R_S	1.000				
MS_15R	-0.002	1.000			
MS_15R_S	0.646	0.003	1.000		
SR	-0.134	-0.071	-0.116	1.000	
SR_SE	0.769	0.000	0.791	-0.122	1.000
SLFR_G1R	0.002	-0.064	0.000	0.227	-0.006
SLFR_G1R	0.515	0.013	0.489	-0.094	0.694
SLFR_G3R	-0.019	-0.010	-0.029	0.289	-0.020
SLFR_G3R	0.604	0.011	0.580	-0.110	0.761
SLFR_G5R	-0.018	-0.085	0.015	0.276	-0.013
SLFR_G5R	0.674	-0.033	0.634	-0.106	0.797
SLFR_15R	-0.013	0.030	0.008	0.174	0.002
SLFR_15R	0.702	-0.002	0.831	-0.105	0.911
READ	-0.114	-0.091	-0.103	0.754	-0.108
READ_SE	0.797	0.008	0.754	-0.119	0.968
WJPCSCG3	-0.028	-0.036	-0.026	0.046	-0.015
WJPCSCG3	0.726	0.027	0.671	-0.119	0.840
WJPCSCG5	-0.011	0.019	0.011	-0.094	0.006
WJPCSCG5	0.792	0.011	0.754	-0.100	0.875
WJPCSCX5	0.010	-0.056	0.000	0.150	-0.006
WJPCSCX5	0.671	0.013	0.801	-0.095	0.831
MATH	-0.096	-0.088	-0.085	0.747	-0.095
MATH_SE	0.781	0.007	0.744	-0.122	0.975
WJAPSC1S	-0.050	-0.061	-0.047	0.312	-0.054
WJAPSC1S	0.672	0.021	0.645	-0.130	0.841
WJAPSCG3	-0.024	-0.045	0.018	0.196	-0.013
WJAPSCG3	0.721	0.019	0.660	-0.124	0.853
WJAPSCG5	-0.007	-0.014	-0.017	0.190	-0.004

WJAPSCG5	0.805	0.006	0.727	-0.097	0.870
WJAPSCX5	0.004	0.008	-0.013	0.138	-0.004
WJAPSCX5	0.671	0.010	0.802	-0.094	0.838

Correlations

	SLFR_G1R	SLFR_G1R	SLFR_G3R	SLFR_G3R	SLFR_G5R
SLFR_G1R	1.000				
SLFR_G1R	-0.010	1.000			
SLFR_G3R	0.210	-0.020	1.000		
SLFR_G3R	-0.025	0.685	-0.013	1.000	
SLFR_G5R	0.114	0.005	0.270	-0.017	1.000
SLFR_G5R	-0.014	0.596	-0.027	0.726	-0.013
SLFR_15R	-0.081	0.007	-0.130	0.000	-0.226
SLFR_15R	0.002	0.535	-0.027	0.636	-0.016
READ	0.066	-0.079	0.196	-0.085	0.175
READ_SE	-0.002	0.679	-0.009	0.753	-0.011
WJPCSCG3	0.038	-0.001	0.041	-0.022	0.023
WJPCSCG3	-0.007	0.631	-0.018	0.726	-0.030
WJPCSCG5	-0.097	0.024	-0.084	0.007	0.021
WJPCSCG5	0.014	0.580	-0.006	0.662	-0.024
WJPCSCX5	0.015	-0.004	0.103	0.006	0.165
WJPCSCX5	0.003	0.517	-0.005	0.589	0.007
MATH	0.109	-0.080	0.205	-0.077	0.185
MATH_SE	-0.006	0.704	-0.011	0.756	-0.009
WJAPSC1S	0.136	-0.071	0.113	-0.047	0.072
WJAPSC1S	0.002	0.800	-0.025	0.695	-0.013
WJAPSCG3	0.057	-0.024	0.078	-0.019	-0.007
WJAPSCG3	-0.013	0.657	-0.013	0.745	-0.026
WJAPSCG5	0.050	0.006	0.031	-0.009	0.064
WJAPSCG5	0.016	0.584	-0.001	0.662	-0.027
WJAPSCX5	0.055	-0.025	0.124	-0.011	0.151
WJAPSCX5	0.000	0.525	0.002	0.595	0.012

Correlations

	SLFR_G5R	SLFR_15R	SLFR_15R	READ	READ_SE
SLFR_G5R	1.000				
SLFR_15R	0.009	1.000			
SLFR_15R	0.709	0.003	1.000		
READ	-0.091	-0.105	-0.087	1.000	
READ_SE	0.779	0.006	0.839	-0.102	1.000
WJPCSCG3	0.015	-0.078	-0.023	0.119	-0.015
WJPCSCG3	0.711	0.027	0.758	-0.097	0.885
WJPCSCG5	0.009	0.077	0.011	0.029	0.000
WJPCSCG5	0.718	0.022	0.839	-0.089	0.923
WJPCSCX5	-0.024	-0.040	-0.007	0.270	-0.001
WJPCSCX5	0.635	0.018	0.888	-0.092	0.826
MATH	-0.077	-0.086	-0.072	0.924	-0.087
MATH_SE	0.773	0.002	0.829	-0.106	0.994
WJAPSC1S	-0.044	-0.089	-0.040	0.266	-0.049
WJAPSC1S	0.665	0.001	0.714	-0.114	0.850

WJAPSCG3	-0.022	-0.074	-0.001	0.163	-0.016
WJAPSCG3	0.720	0.020	0.746	-0.096	0.899
WJAPSCG5	0.025	-0.227	-0.004	0.073	-0.010
WJAPSCG5	0.725	0.018	0.810	-0.084	0.926
WJAPSCX5	-0.020	-0.195	-0.007	0.138	0.002
WJAPSCX5	0.641	0.008	0.894	-0.090	0.828

Correlations

	WJPCSCG3	WJPCSCG3	WJPCSCG5	WJPCSCG5	WJPCSCX5
WJPCSCG3	1.000				
WJPCSCG3	-0.018	1.000			
WJPCSCG5	-0.155	0.018	1.000		
WJPCSCG5	-0.030	0.879	-0.002	1.000	
WJPCSCX5	-0.185	-0.014	-0.619	-0.004	1.000
WJPCSCX5	-0.024	0.782	0.001	0.892	0.003
MATH	0.033	-0.085	-0.110	-0.065	0.210
MATH_SE	-0.013	0.863	0.001	0.892	-0.001
WJAPSC1S	0.111	-0.062	-0.162	-0.026	0.061
WJAPSC1S	-0.007	0.790	-0.015	0.779	0.012
WJAPSCG3	0.215	-0.029	-0.214	-0.013	-0.056
WJAPSCG3	-0.015	0.987	0.018	0.855	-0.012
WJAPSCG5	0.065	-0.032	-0.206	-0.017	-0.057
WJAPSCG5	-0.026	0.867	-0.004	0.990	-0.002
WJAPSCX5	-0.159	-0.003	-0.490	0.007	0.219
WJAPSCX5	-0.017	0.779	0.005	0.882	0.001

Correlations

	WJPCSCX5	MATH	MATH_SE	WJAPSC1S	WJAPSC1S
WJPCSCX5	1.000				
MATH	-0.071	1.000			
MATH_SE	0.804	-0.094	1.000		
WJAPSC1S	-0.023	0.384	-0.054	1.000	
WJAPSC1S	0.714	-0.103	0.864	-0.071	1.000
WJAPSCG3	-0.014	0.245	-0.017	0.219	-0.055
WJAPSCG3	0.754	-0.085	0.883	-0.067	0.810
WJAPSCG5	-0.029	0.234	-0.011	0.043	-0.027
WJAPSCG5	0.836	-0.062	0.897	-0.028	0.783
WJAPSCX5	0.003	0.264	0.001	-0.063	0.020
WJAPSCX5	0.986	-0.074	0.811	-0.027	0.722

Correlations

	WJAPSCG3	WJAPSCG3	WJAPSCG5	WJAPSCG5	WJAPSCX5
WJAPSCG3	1.000				
WJAPSCG3	-0.028	1.000			
WJAPSCG5	0.136	-0.034	1.000		
WJAPSCG5	-0.012	0.850	-0.013	1.000	
WJAPSCX5	-0.139	-0.002	-0.121	0.008	1.000
WJAPSCX5	-0.012	0.756	-0.037	0.832	0.002

```
          Correlations
              WJAPSCX5
              ________
 WJAPSCX5       1.000
```

PLOT INFORMATION

The following plots are available:

  Histograms (sample values, estimated factor scores)
  Scatterplots (sample values, estimated factor scores)
  Latent variable distribution plots

** No plots of individual estimated values are available due to computational
** errors in one or more groups.

DIAGRAM INFORMATION

  Use View Diagram under the Diagram menu in the Mplus Editor to view the diagram.
  If running Mplus from the Mplus Diagrammer, the diagram opens automatically.

  Diagram output
    c:\users\tuf22063\onedrive - temple university\foley & weinraub revision\r2\models\r2.6.1. ds - replication test - r

     Beginning Time: 18:44:03
        Ending Time: 19:06:13
       Elapsed Time: 00:22:10

MUTHEN & MUTHEN
3463 Stoner Ave.
Los Angeles, CA 90066

Tel: (310) 391-9971
Fax: (310) 391-8971
Web: www.StatModel.com
Support: Support@StatModel.com

Copyright (c) 1998-2023 Muthen & Muthen
