# Supplementary material for: On the Broader Significance of Maternal Sensitivity: Mothers’ Early and Later Sensitive Parenting Matter to Children's Language, Executive Function, Academics, and Self‐Reliance
Source: Dev Sci. 2024 Dec 16;28(1):e13594. doi: 10.1111/desc.13594 (PMC11647561; doi:10.1111/desc.13594)
Supplement: Supplementary file 2 — Supporting information [file DESC-28-e13594-s002.pdf]

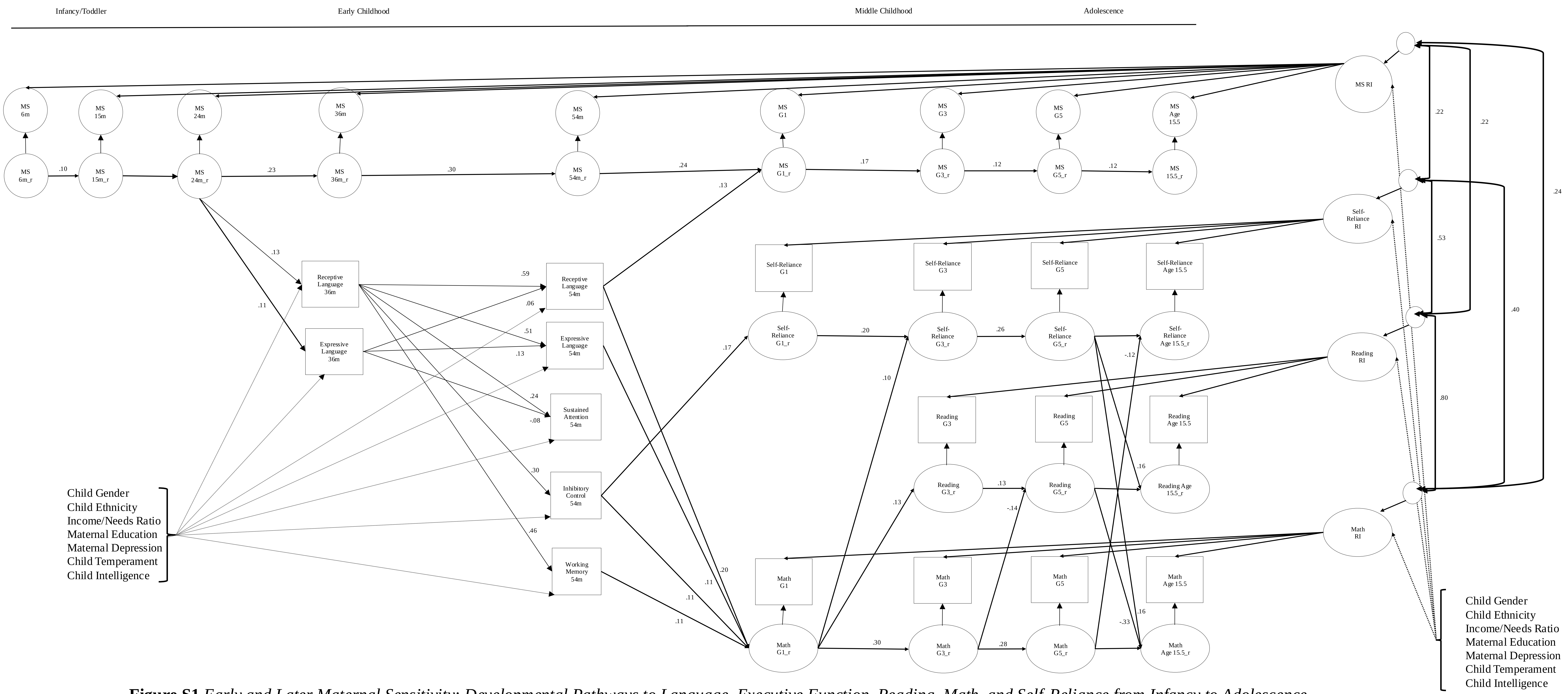

Figure S1 *Early and Later Maternal Sensitivity: Developmental Pathways to Language, Executive Function, Reading, Math, and Self-Reliance from Infancy to Adolescence*
Note. Standardized beta coefficients shown. $p < .05$.
